# Ranking Mammal Species for Conservation and the Loss of Both Phylogenetic and Trait Diversity

**DOI:** 10.1371/journal.pone.0141435

**Published:** 2015-12-02

**Authors:** David W. Redding, Arne O. Mooers

**Affiliations:** 1 BISC, Simon Fraser University, Burnaby, Canada; 2 IRMACS, Simon Fraser University, Burnaby, Canada; 3 CBER, Department of Genetics, Evolution and Environment, University College London, London, United Kingdom; BiK-F Biodiversity and Climate Research Center, GERMANY

## Abstract

The 'edge of existence' (EDGE) prioritisation scheme is a new approach to rank species for conservation attention that aims to identify species that are both isolated on the tree of life and at imminent risk of extinction as defined by the World Conservation Union (IUCN). The self-stated benefit of the EDGE system is that it effectively captures unusual 'unique' species, and doing so will preserve the total evolutionary history of a group into the future. Given the EDGE metric was not designed to capture total evolutionary history, we tested this claim. Our analyses show that the total evolutionary history of mammals preserved is indeed much higher if EDGE species are protected than if at-risk species are chosen randomly. More of the total tree is also protected by EDGE species than if solely threat status or solely evolutionary distinctiveness were used for prioritisation. When considering how much trait diversity is captured by IUCN and EDGE prioritisation rankings, interestingly, preserving the highest-ranked EDGE species, or indeed just the most threatened species, captures more total trait diversity compared to sets of randomly-selected at-risk species. These results suggest that, as advertised, EDGE mammal species contribute evolutionary history to the evolutionary tree of mammals non-randomly, and EDGE-style rankings among endangered species can also capture important trait diversity. If this pattern holds for other groups, the EDGE prioritisation scheme has greater potential to be an efficient method to allocate scarce conservation effort.

## Introduction

Much of the effort in global conservation is directed by the aim of conserving the most threatened species first [1]. This threat-led approach is likely not adequate in the long term, as there are not enough resources, or social will, to conserve all threatened species, leaving difficult decisions about which threatened species to concentrate on first [2]. Given the funding shortfall, and the urgency with which decisions need to be made [3,4], focussing on the most critically endangered species first may not be the optimum use of resources to conserve biodiversity [5] and, therefore, it becomes important to consider what other methods can be used alongside threat.

The ‘Evolutionarily Distinctive and Globally Endangered’ (EDGE) project is a first-of-its-kind international, independently-funded conservation programme that has aims to set priorities for specific target clades of species and, having achieved that, provide resources to protect priority species. It focuses on those species that are both threatened with imminent extinction and that also have few close relatives from an evolutionary perspective [6, 7]. Integral to the programme, the EDGE metric used to set conservation priorities consists of the sum of a species’ threat status (probability of extinction) and a measure of its evolutionary isolation [8]. Using this compound approach, the programme hopes that it will help preserve evolutionary history [9] and prevent the extinction of unique species that are “like no other on the planet” [7].

The EDGE approach is increasingly used in academic conservation analyses as a credible method to set global species priorities [10, 11]. As a result of the initial mammal list, and follow up EDGE lists for the world’s amphibians [12] and birds [13], there are now several active EDGE-based conservation programmes for a variety of species, and the programme is expanding to prioritise corals [14] and other groups. In this context, great emphasis is currently placed on the valuable attributes of the individual high-ranking species in the respective EDGE lists, but little is known about what EDGE species, as a group, add to our overall aim of biodiversity conservation.

As argued by Faith [15] there is a strong connection between the EDGE scoring method, made up as it is of a measure of phylogenetic position and an estimated extinction probability, and the concepts of phylogenetic diversity and future evolutionary history. Phylogenetic diversity [16] is measured as the sum of all branch lengths on a phylogenetic tree, and future evolutionary history is measured as the sum of all branch lengths weighted by the probability that each branch persists [17]. However, there are several problems with using current implementations of phylogenetic diversity for choosing those sets of species that maximise the protection of evolutionary history, problems that have prevented it from being used in an active conservation programme. Most obviously it produces many possible sets of species that capture maximum PD (phylogenetic diversity) and thus does not transparently set actionable targets. Here, we examine the viability of using a species-specific conservation index (the EDGE score) in a real-world example to quantify its ability to capture additional PD.

Maximising PD is expected to maximise total "feature" diversity [16], measured as the sum of the branch lengths under particular models of evolution. In support of the link between PD value and features, recent ecological work has shown positive relationships between local PD values and aspects of ecosystem functioning independent of species richness [18–21]. The theory here is that increased PD also increases complementary functions. Such a general relationship would synthesise evolutionary and ecological conservation priorities [22]. Due to the relatively low demands for data needed to calculate phylogenies (i.e. genetic sampling is increasingly cheap and produces large quantities of data), phylogenetic diversity could, potentially, act as a useful surrogate for feature diversity and, if the link is established, increase ecological diversity. Thus, when used to set conservation priorities, PD could be a convenient and data cheap way to ensure the protection of several different aspects of biodiversity [23].

Critically, however, the evolutionary isolation metric used to produce EDGE scores was not designed to identify any set of *n* species that would collectively represent the maximum possible amount of phylogenetic diversity, either in the present time [24, 25] or into the future following extinctions. It is indeed easy to devise cartoon scenarios where conserving the top-ranking EDGE species will not conserve the maximum expected PD ([26]).

**Fig 1:**
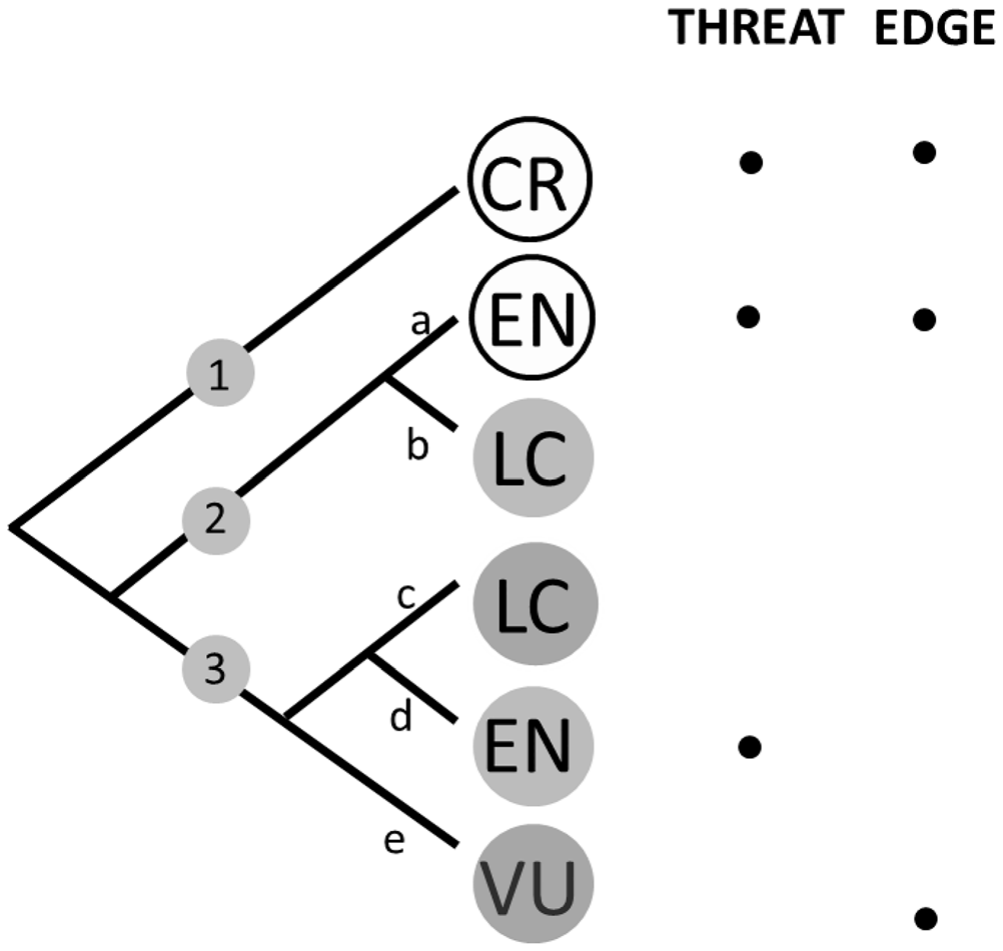
Cartoon tree containing six species, highlighting (with dots) the species that would be preferentially chosen based on threat alone or EDGE scores. Here, species selected using EDGE scores capture more Phylogenetic diversity and more Trait diversity than choosing the most threatened species. Threat categories of the species are represented in the circles at the tips. Column “THREAT” indicates the species’ tips chosen when choosing the most threatened species and “EDGE” when choosing highest EDGE scoring species. Solid circles at tips representing species with ecology type 1 and black bordered circles those with ecology type 2. Main branches on the tree are labelled 1–3 and minor branches a-e.

That the EDGE metric was not designed to explicitly capture present or future evolutionary history does not mean it must perform poorly at this task. Evolutionarily distinct species represent the deep (major) lineages of the tree of life rather than shallow (minor) ones (Fig 1: consider when branch 1 is represented at the expense of branch d). This may ensure that large sections of the tree of life are not lost through accident or neglect. Subsets of species that maximise PD are maximally dispersed across the tree and, therefore, have a representative from major lineage [25]. Simulation studies have shown that subsets of high ED (highly distinctive species) species also capture more PD than random sets of species on random trees [25]. However, and critically for this study, these results are not directly relevant to conservation: much of the evolutionary history of any clade (i.e. those evolutionary changes common to, say, all birds or mammals) will be represented by any number of least concern species that will persist regardless of specific conservation management plans. Therefore, we must consider the *additional* history or diversity one set of species may offer if chosen for active conservation over another.

Here, we use the class Mammalia to test whether protecting EDGE species protects additional mammalian evolutionary history compared to other prioritisation schemes, i.e. threat only, ED only and, for comparisons sake, random selection. Given that extinction is inherently probabilistic, we considered a medium-term time-frame (~100 years) when looking to conserve phylogenetic or trait diversity [9, 23].

In addition to this straightforward test, we also attempt to connect PD to feature diversity directly, comparing random choice to EDGE rankings of species on trait distance trees [27]. To facilitate this, mammals are the only major group of vertebrates for which there is a relatively complete dated phylogenetic tree [28] (called the Fritz phylogeny here) alongside a large public database of traits (Pantheria [29]).

## Methods

### Mammal evolutionary and trait data

Threat status for each mammal species was taken from the 2009 IUCN red list [30]. We used the 2009 IUCN red list because its taxonomy is closer to the Fritz phylogeny. Trait information was downloaded from the online database Pantheria [29]. The species names from the Pantheria database, the Fritz phylogeny [28] and IUCN red list were matched using Mammals of the World (MSW3) [31] as a taxonomic reference. Species in MSW3 represented by two named tips on the Fritz phylogeny but with only one threat category were lumped together. Species with more than one threat category associated with a single tip were given a threat calculated from the mean threat category rounded to the nearest integer. Species that were data-deficient were not included in the analysis but were left in the tree as removing them would affect other species’ ED scores. This left 4920 species with both a threat score and a score of evolutionary isolation. The trait database was then collapsed so that there was only one line of data for any species on the final tree, representing the average (mean for continuous or modal for discrete) trait value.

Though there are a number of traits to choose from in the Pantheria database, many are correlated. We therefore chose the 10 traits (data in Appendix One) that were the most orthogonal with respect to each other for input to the distance-based trait tree. We only kept those species that had at least 4 of the 10 variables present and the final trait dataset was roughly 75% complete and contained approximately 80% of all 4920 mammal species (S1 Table). Alternative datasets with more and less completeness showed no qualitative differences in results (S1 Fig). From our set of ten (orthogonal) trait variables we created a pairwise distance matrix for all species using the Gower approach [32], as used by previous studies [27, 33] (calculated using R package ape [34]). Missing data were dealt with on a pair-wise basis, with distances between species only being calculated using non-missing data; species without complete pairs of data were awarded the average of distance between all species pairs with complete pair data. We then created an unrooted tree representing the trait diversity of the World’s mammals by calculating, from the distance matrix, an average-weighted neighbour-joining tree (function nj; package ape [34]).

We then produced two further trees on which we based our extinction analyses: one of future evolutionary history [26] based on the Fritz phylogeny, and one of future trait diversity (or TD), based on the trait diversity distance tree created above. We term these two future predictions our ‘baseline trees’ as they represent the baseline amount of diversity expected to be present in 100 years, given no change to current rates of threat. Following the methodology of Witting & Loeschcke [17, 35], we produced the two baseline trees by weighting each branch length of the respective input trees by their probability of persistence (1-p(extinction)), with p(extinction) values following Redding & Mooers [8]. Critically Endangered (CR) species were assigned a p(extinction) of 0.999, Endangered (EN) species a value of 0.786, Vulnerable (VU) species a value of 0.1, Near Threatened (NT) were given p(ext) = 0.01 and Least Concern (LC) a value of 0.001. These values were calculated by extrapolating from the criterion E section from the IUCN threat listing process, verified against a survey of quantitative threat analyses from the literature [8]. Given we are forecasting into the future, we refer to CR, EN, VU and NT species as being “threatened” below. Using this approach, tip branches that lead to species that are in the IUCN red list category ‘Least Concern’ are transformed very little, and tip branches that connected to species in the ‘Critically Endangered’ category were reduced to almost zero (as they have low probability of contributing to the future tree). Internal branches were transformed using the combined probabilities of persistence of all connected daughter branches (two in the case of a bifurcating tree) i.e. In the case where we have two tips, one that is Critically Endangered p(extinction) = 0.999 and the other Endangered p(extinction) = 0.786, the probability that the interior branch persists is 1 minus the probability that both tip-ward branches go extinct: 1-(0.786*0.999) = 0.215. The internal branch, therefore, has a probability of persistence of 0.215.

### Analysis

Our overall scheme for investigating the gain of phylogenetic and trait diversity under different conservation schemes was divided into three steps:

First, we awarded a set of 100 species a zero threat score (thereby designating them as successfully protected) five times, each time choosing the sets of species using a different method:

1. 100 random species drawn from the 1350 threatened species—chosen to create a more realistic and conservative null comparison compare to unrestricted random selection
2. the 100 top-ranked ED species
3. the 100 most threatened species (with random sampling for within-category ties)
4. the 100 top-ranked EDGE species (i.e. a combination of (a) and (b))–EDGE score calculated using the original Isaac et al. [6] formula.
5. 100 group of species capturing the maximum amount of PD or TD (i.e. one of many possible solutions for optimal choice for capturing PD or TD obtained using a greedy algorithm [14])

Second, within each of the five threat category lists resulting from step one we simulated on-going threat changes across all mammals by altering the threat statuses of a further 1500 species randomly, with the category change taken from normal distribution of mean -1 and standard deviation of 1 and rounded to the nearest integer. These parameter values approximate a general pattern of threat increase seen in empirical studies [36], which, over a 100 year period, predict roughly 1300 shifts to a more-threatened IUCN category in mammals.

Third, using the five future threat category listing scenarios, the branches of six separate Fritz phylogenies were re-weighted using the same approach used to create the baseline future PD tree (see above).

The whole process was then repeated substituting the raw trait diversity tree for the phylogeny in step 3. These twelve altered trees represent six possible future alternatives for both the phylogenetic and trait diversity of the world’s mammals, each with a different schemes of protection. The difference between the summed total branch lengths of the baseline PD or TD trees and the summed total branch lengths of the altered trees represents the conservation gain in PD or TD for each of the prioritisation methods. We compared these conservation gains for the different methods of species prioritisation over 1000 simulations, a limit set as a compromise between sensitivity testing and computational cost.

The random choice of (threat-listed) species was considered the baseline protection scenario. We chose to use random choice as a null comparison because species that receive high levels of conservation funding have tended to be focussed in very narrow taxonomic groups e.g. Raptors and Carnivores [37] and random choice, therefore, represents a conservative null hypothesis—phylogenetic clumping of conservation under the status quo may make phylogenetic and trait diversity loss more extreme.

## Results

### Phylogenetic diversity

**Fig 2:**
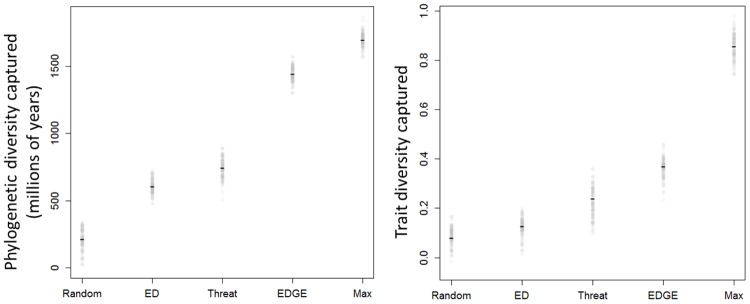
Extra phylogenetic diversity protected (panel a) and extra trait diversity protected (panel b) over no conservation effort, achieved when protecting 100 threatened mammal species from going extinct. Black bar represents mean value and grey circles results from 1000 simulations with random resolution of tied species. The *x*-axis represents five different methods of choosing species to protect: “RANDOM” represents random choice among threatened species; ED” the most evolutionary distinctive species; “Threat” represents choosing the most threatened first; “EDGE” the highest EDGE scoring species; and “MAX” is one of the optimal sets of *n* species for capturing total diversity, calculated using a greedy algorithm.

Using the EDGE rankings to identify 100 extra species to conserve, saved more additional phylogenetic diversity of mammals (ANOVA, n = 1000, p<0.001) than using threat status, using just ED score, or using random choice of threatened species: in the last comparison, the EDGE approach saved around 1435 million more years of evolution history (Fig 2a) than when randomly conserving 100 extra species, which in turn conserved just 205 million years more on average than doing nothing. Importantly, the EDGE scoring system captured approximately 87.5% of the maximum possible future PD. Prioritising by threat (ANOVA, n = 1000, p<0.001) and ED (ANOVA, n = 1000, p<0.001) also conserved more of the evolutionary tree of mammals than by randomly choosing threatened species but captured just 40% of the maximum amount of PD (Fig 2a). Replications of the analysis with 50 & 150 extra species conserved showed very similar patterns of results (S2 Fig).

### Trait diversity

Although it captured more trait diversity in all three cases, using EDGE ranking did not significantly protect more trait diversity than when using threat status alone (ANOVA, n = 1000, p~0.50). Prioritising species by either their threat status or EDGE score protected more additional trait diversity of mammals (ANOVA, n = 1000, p<0.05), capturing approximately two and three times more trait diversity than random choice (Fig 2b).

## Discussion

Choosing species for conservation attention using EDGE scores protected the tree of mammals (measured using PD) more effectively than all other methods used here. Considering that our analyses are based on expected PD projections based on current threat status, it is unsurprising that choosing to conserve the most threatened species first also provides a reasonable solution to protect future PD: choosing imminently threatened species will prioritise the PD that is most likely to be lost in the near future. This observation links the EDGE approach directly to the extensive theoretical literature on expected PD [15, 38]. Using Evolutionary Distinctiveness alone to choose species is also effective, as it seems to protect those species that, while they may not be severely threatened still represent large amounts of unique (species-specific) PD, thereby protecting greater amounts of PD under reasonable scenarios for future loss. The EDGE approach seems to combine information from both ED and threat such that it is more effective at protecting additional phylogenetic diversity than, for instance, threat alone. This pattern supports recent work for birds [13], where ranking threatened species by their ED scores also led to near-optimal PD gains.

Using the EDGE rankings also preserves more trait diversity than random choice, supporting the claim that conserving EDGE species achieves the stated aim of conserving remarkable feature diversity, at least for our data. The expected role of threat and evolutionary distinctness in choosing species with wide sets of features is unclear, but, of the two, under a simple model of divergent evolution high ED species might be expected to have traits different to other species-rich parts of the evolutionary tree. Importantly, however, our finding is driven mainly due to a pattern whereby the threat component of the metric is conserving threatened TD, as high ED species do not appear to contribute a large amount of features to this set over random choice. The modest increases in trait diversity seen when prioritising using EDGE scores over threat scores may result from a limited number of high scoring ED species tending to have unusual characteristics [23]. Previous analyses from an eclectic set of taxa—rockfish (genus *Sebastes* [39]), passerine birds (Order Passerina [40]), cone Shells (genus *Conus* [41]), and mammals [9] have documented that species with few close relatives are somewhat morphologically distinctive. How these unusual features are related to each other and to non-threatened biodiversity is not known, and more work with better characterised species needs to be undertaken to fully understand these relationships. There has also been some further work linking the aim of capturing PD with an ability to capture a greater diversity of functionally distinct species, and possibly an ability to capture ecosystem function in a general sense [21, 33]. Both the shape of a phylogeny and the pattern of trait evolution likely play significant role in how well ED species can represent these components; an investigation into alternative ways to represent trait diversity may yield important insights here.

Repeating this work with other taxonomic groups and much larger sets of traits are important next steps. Plant groups may offer good data. Some groups of species, however, may be less well-studied and different methods will needed to be employed to account for the missing threat data, poor phylogenetic information, and poor coverage of trait data. For instance, it might be that, within groups, species with missing data are disproportionately found in less well-studied environments, such as rainforests, and share particular sets of traits as a result. Quantifying such biases will aid the application of analyses such as ours to other taxonomic groups. Furthermore, it may be that the way the EDGE score are implemented for other taxonomic groups needs to be expanded: In this case the standard formulation [6] of multiplying the GE part of the score by log(2) produced near optimal PD capture by the resulting EDGE scores (S3 Fig), but for other taxonomic groups it could be advisable to adapt this weighting to maximise PD and trait diversity.

These results regarding phylogenetic and feature diversity should also be considered from the point of view of costs. With the funding shortfall within conservation, using an EDGE type approach moves focus away from the most critically endangered, and potentially most expensive [5], species. We can start to consider cost in the context of our analysis in the following crude (partly because it ignores geographical distribution [13]) way. Consider the number of threat categories a species needs to traverse before it is not considered under threat (i.e. 4 for a critically endangered species (CR -> VU -> EN -> NT -> LC) versus 2 for an endangered species (EN -> NT -> LC) as an index of increasing cost. Under this metric, the EDGE approach to prioritization is more efficient at conserving PD than using threat status alone, with species needing to be declassified by 65 fewer summed threat categories compared to the solely threat-led approach (535 vs 600). We note that when conserving TD, although the amount of overall TD captured is roughly the same using the threat and EDGE approaches, EDGE is a more efficient (again by 65 few category changes) and so *potentially* a less costly method also to conserve trait diversity.

In this context, an important future development would be to model the extinction trajectories of all species to better account for background extinction rather than using the random approach applied here. This is clearly a labour intensive problem where we could use intrinsic (e.g. body size, specialisation, phylogenetic patterns) and extrinsic (e.g. range size, range habitat quality) factors to predict future threat changes (see, e.g. [42]). This would allow us to more precisely test the impact of saving those particular species highlighted by an EDGE type approach, in order to to test whether the expected benefits predicted here are likely to be seen in reality. Another future development would be to formally examine cost alongside other important factors such as political governance in target conservation areas [43].

Pragmatic conservation prioritisation is necessary [2], and prioritizing based on threat does seem prudent (Fig 2). The EDGE programme is one of few prioritization programmes that have fully implemented a quantitative approach to valuing species on threat and other axes. Given the current state of the literature, it seems to be effective in achieving its aims. Though more work on a wider range of taxa is sorely needed [44], if similar patterns hold for other groups, and the benefits of using phylogenetic information in conservation further consolidated, then ranking species using the EDGE process should be seriously considered in any species-based conservation prioritisation scheme.

## Supporting Information

S1 Fig Extra trait diversity protected, compared to no extra conservation effort, when protecting 100 threatened mammal species from going extinct based on trait-based distance trees built using (a) large incomplete (b) medium complete (c) small complete trait data set. Dataset (a) contained all those species that had at least 3 of the 10 variables present and contained roughly 75% complete data and approximately 80% of all 4920 mammal species. Dataset (b) contained all those species that had at least 4 of the 10 variables present and contained roughly 80% complete data and approximately 70% of all mammal species. Dataset (c) contained all those species that had at least 5 of the 10 variables present and contained roughly 85% complete data and approximately 60% of all mammal species. Black bar represents mean value and grey circles results from 1000 simulations. The *x*-axis represents six different methods of choosing species to conserve in perpetuity: “GE” represents choosing the most threatened first; “EDGE” the highest EDGE scoring species; “ED” the most evolutionary distinctive species; “ThreatED” the most distinctive species calculated on a phylogeny where branch lengths have been resized proportional to the threat of loss of that branch; “RANDOM” represents random choice of the threatened species; and “MAX” is one of the optimal sets of *n* species for capturing total diversity, calculated using a greedy algorithm.
(TIF)

S2 Fig Total extra phylogenetic diversity (PD) conserved, compared to no extra conservation effort, achieved when protecting (a) 50, (b) 100, (c) 150 threatened mammal species from going extinct. Black bar represents mean value and grey circles results from 1000 simulations. The *x*-axis represents six different methods of choosing species to conserve in perpetuity: “GE” represents choosing the most threatened first; “EDGE” the highest EDGE scoring species; “ED” the most evolutionary distinctive species; “ThreatED” the most distinctive species calculated on a phylogeny where branch lengths have been resized proportional to the threat of loss of that branch; “RANDOM” represents random choice of the threatened species; and “MAX” is one of the optimal sets of *n* species for capturing total diversity, calculated using a greedy algorithm.
(TIF)

S3 Fig Different relative weighting of the two components of EDGE scores (Evolutionary Distinctiveness and Global Endangerment) result in different amounts of extra PD (solid line) and TD (dashed line) being captured when choosing to protect 150 top scoring species from going extinct over the next 100 years, compared to randomly choosing 150 threatened species from going extinct. New EDGE ranking lists were created by substituting the term *ln(2)* for the values 0.05, 0.25, 0.5, 0.75, 1, 1.25, 1.5, and 2 (x-axis) in the original (Isaac et al. 2007) formula: EDGE = ln(1+ED) +GE*ln(2)
(TIF)

S1 Table Raw trait data used in the analysis. (DOCX)
