# Supplementary figures and images for: Ranking Mammal Species for Conservation and the Loss of Both Phylogenetic and Trait Diversity
Source: PLoS One. 2015 Dec 2;10(12):e0141435. doi: 10.1371/journal.pone.0141435 (PMC4668038; doi:10.1371/journal.pone.0141435)

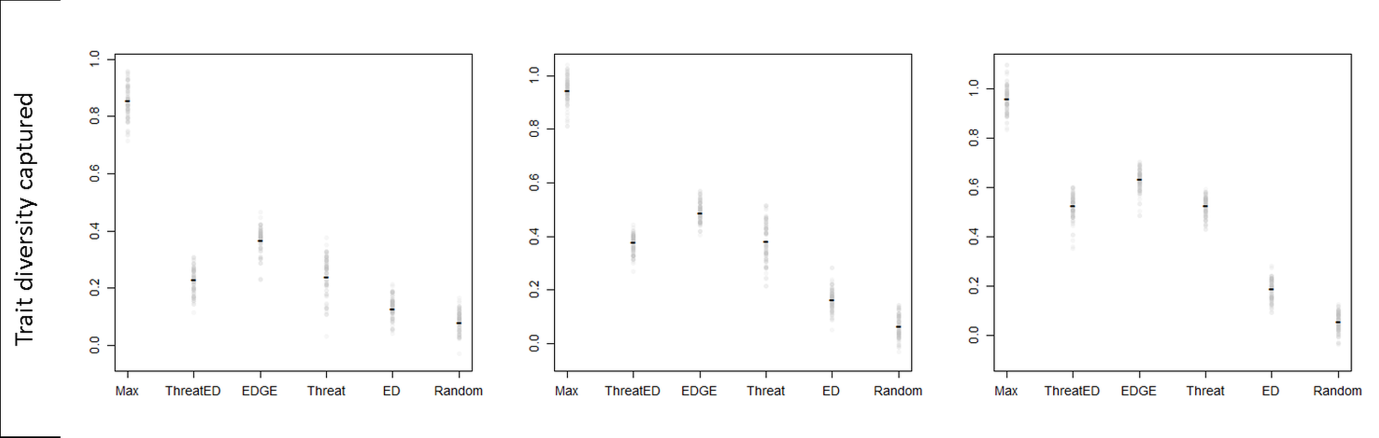

Supplement: S1 Fig — Dataset (a) contained all those species that had at least 3 of the 10 variables present and contained roughly 75% complete data and approximately 80% of all 4920 mammal species. Dataset (b) contained all those species that had at least 4 of the 10 variables present and contained roughly 80% complete data and approximately 70% of all mammal species. Dataset (c) contained all those species that had at least 5 of the 10 variables present and contained roughly 85% complete data and approximately 60% of all mammal species. Black bar represents mean value and grey circles results from 1000 simulations. The x-axis represents six different methods of choosing species to conserve in perpetuity: “GE” represents choosing the most threatened first; “EDGE” the highest EDGE scoring species; “ED” the most evolutionary distinctive species; “ThreatED” the most distinctive species calculated on a phylogeny where branch lengths have been resized proportional to the threat of loss of that branch; “RANDOM” represents random choice of the threatened species; and “MAX” is one of the optimal sets of n species for capturing total diversity, calculated using a greedy algorithm. (TIF) [file pone.0141435.s001.tif]

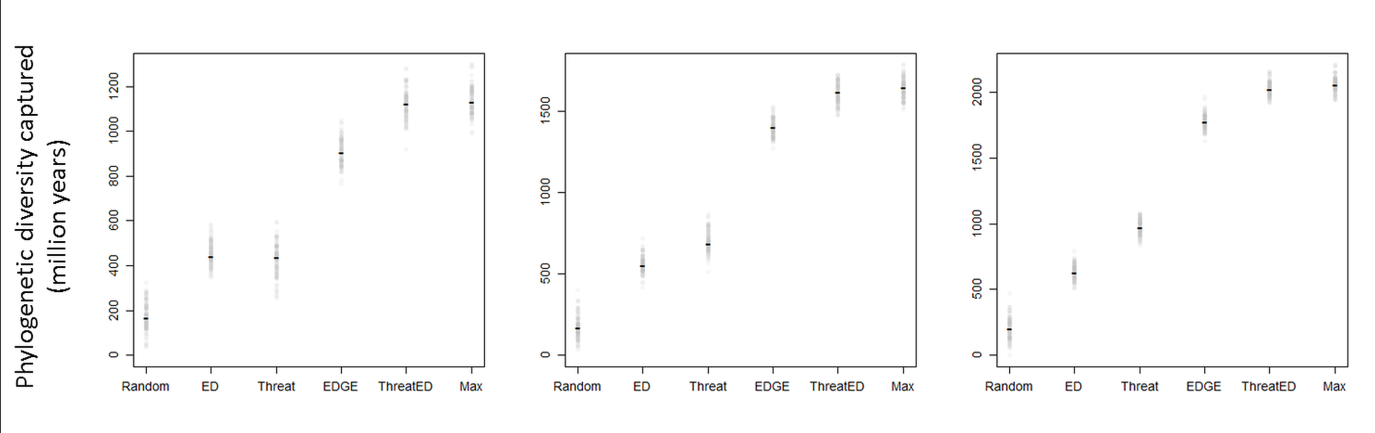

Supplement: S2 Fig — Black bar represents mean value and grey circles results from 1000 simulations. The x-axis represents six different methods of choosing species to conserve in perpetuity: “GE” represents choosing the most threatened first; “EDGE” the highest EDGE scoring species; “ED” the most evolutionary distinctive species; “ThreatED” the most distinctive species calculated on a phylogeny where branch lengths have been resized proportional to the threat of loss of that branch; “RANDOM” represents random choice of the threatened species; and “MAX” is one of the optimal sets of n species for capturing total diversity, calculated using a greedy algorithm. (TIF) [file pone.0141435.s002.tif]

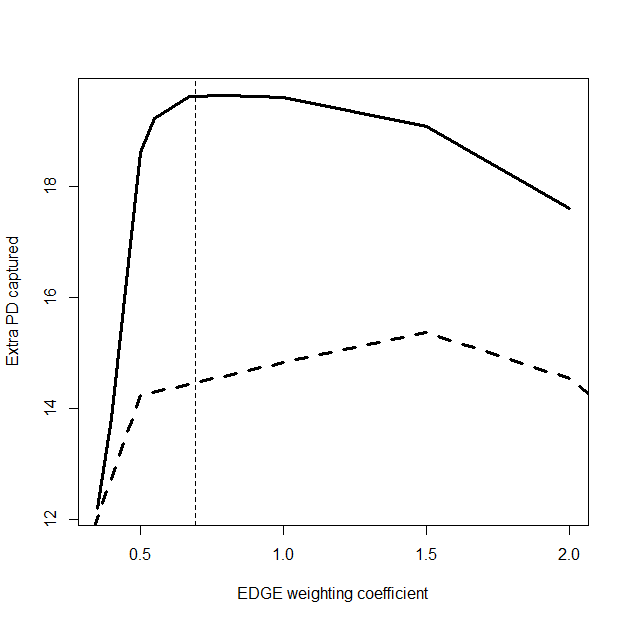

Supplement: S3 Fig — New EDGE ranking lists were created by substituting the term ln(2) for the values 0.05, 0.25, 0.5, 0.75, 1, 1.25, 1.5, and 2 (x-axis) in the original (Isaac et al. 2007) formula: EDGE = ln(1+ED) +GE*ln(2) (TIF) [file pone.0141435.s003.tif]
