# Supplementary material for: Ranking Mammal Species for Conservation and the Loss of Both Phylogenetic and Trait Diversity
Source: PLoS One. 2015 Dec 2;10(12):e0141435. doi: 10.1371/journal.pone.0141435 (PMC4668038; doi:10.1371/journal.pone.0141435)
Supplement: S1 Table — (DOCX) [file pone.0141435.s004.docx]

Table S1. Raw trait data used in the analysis.

| MSW05_Order | MSW05_Family | MSW05_Genus | MSW05_Species | MSW05_Binomial | AdultBodyMass_g | LitterSize | GR_Area_km2 | Precip_Mean_mm | Temp_Mean_degC | ActivityCycle | DietBreadth | HabitatBreadth | Terrestriality | TrophicLevel | EDGE_SCORE |
| --- | --- | --- | --- | --- | --- | --- | --- | --- | --- | --- | --- | --- | --- | --- | --- |
| Rodentia | Muridae | Melomys | rubicola | Melomys rubicola | 100 | NA | 1.87E-04 | NA | NA | NA | NA | NA | NA | NA | 1.922328 |
| Rodentia | Cricetidae | Peromyscus | dickeyi | Peromyscus dickeyi | 28.6 | NA | 7.25E-03 | NA | NA | 1 | NA | NA | NA | NA | 1.693332 |
| Soricomorpha | Talpidae | Mogera | uchidai | Mogera uchidai | NA | NA | 0.03 | NA | NA | 2 | 1 | 1 | 1 | 3 | 0.36334 |
| Chiroptera | Pteropodidae | Pteropus | pelewensis | Pteropus pelewensis | NA | NA | 0.06 | NA | NA | NA | NA | NA | NA | NA | 0.145922 |
| Rodentia | Capromyidae | Mysateles | garridoi | Mysateles garridoi | NA | NA | 0.49 | NA | NA | NA | 3 | 1 | 2 | 2 | 1.661717 |
| Rodentia | Cricetidae | Neotoma | anthonyi | Neotoma anthonyi | 195.4 | NA | 0.66 | NA | NA | NA | NA | NA | NA | NA | 1.563717 |
| Rodentia | Cricetidae | Microtus | breweri | Microtus breweri | NA | 4.5 | 1.85 | NA | NA | NA | 1 | 2 | 1 | 1 | 0.718228 |
| Chiroptera | Vespertilionidae | Hypsugo | lophurus | Hypsugo lophurus | NA | NA | 2.7 | 290 | 24.6 | NA | NA | 1 | 2 | NA | 0.289402 |
| Rodentia | Cricetidae | Neotoma | martinensis | Neotoma martinensis | 240.16 | NA | 2.8 | NA | NA | NA | NA | NA | NA | NA | 1.563717 |
| Chiroptera | Pteropodidae | Pteropus | howensis | Pteropus howensis | 232.92 | 0.98 | 3.16 | NA | NA | NA | NA | 1 | 2 | NA | 0.294088 |
| Soricomorpha | Soricidae | Crocidura | dhofarensis | Crocidura dhofarensis | NA | NA | 3.52 | 7 | 21.1 | NA | NA | NA | NA | NA | 0.286234 |
| Rodentia | Capromyidae | Mesocapromys | angelcabrerai | Mesocapromys angelcabrerai | NA | NA | 3.55 | 111 | 24.6 | NA | 3 | 1 | 2 | 2 | 1.35101 |
| Lagomorpha | Ochotonidae | Ochotona | gaoligongensis | Ochotona gaoligongensis | NA | NA | 4.89 | 195 | 11.3 | NA | NA | 1 | 1 | NA | 0.289957 |
| Chiroptera | Vespertilionidae | Myotis | abei | Myotis abei | NA | NA | 5.11 | 52 | -0.9 | NA | NA | 1 | 2 | NA | 0.276485 |
| Rodentia | Muridae | Rhynchomys | isarogensis | Rhynchomys isarogensis | 122.29 | NA | 5.28 | 331 | 25.2 | 2 | 1 | NA | NA | 3 | 0.616545 |
| Rodentia | Calomyscidae | Calomyscus | tsolovi | Calomyscus tsolovi | NA | NA | 6.78 | 18 | 17.1 | NA | NA | NA | NA | NA | 0.30668 |
| Didelphimorphia | Didelphidae | Monodelphis | unistriata | Monodelphis unistriata | 55.3 | NA | 6.86 | 107 | 17.9 | 1 | 1 | 1 | 1 | 3 | 0.386822 |
| Rodentia | Capromyidae | Geocapromys | ingrahami | Geocapromys ingrahami | 733.52 | 1.04 | 8.36 | NA | NA | 1 | 2 | 2 | 2 | 1 | 0.788262 |
| Chiroptera | Vespertilionidae | Hesperoptenus | gaskelli | Hesperoptenus gaskelli | NA | NA | 8.89 | 237 | 20.6 | NA | NA | 1 | 2 | NA | 0.302833 |
| Rodentia | Cricetidae | Neotoma | bunkeri | Neotoma bunkeri | 375 | NA | 9.15 | NA | NA | NA | NA | NA | NA | NA | 1.563717 |
| Rodentia | Cricetidae | Peromyscus | pseudocrinitus | Peromyscus pseudocrinitus | NA | NA | 9.15 | NA | NA | 1 | NA | NA | NA | NA | 1.693332 |
| Rodentia | Cricetidae | Peromyscus | sejugis | Peromyscus sejugis | 21.5 | NA | 13.64 | NA | NA | 1 | NA | NA | NA | NA | 1.336841 |
| Chiroptera | Hipposideridae | Paracoelops | megalotis | Paracoelops megalotis | NA | NA | 17.09 | 141 | 23.7 | NA | 1 | 1 | 2 | 3 | 0.344303 |
| Chiroptera | Rhinolophidae | Rhinolophus | montanus | Rhinolophus montanus | NA | NA | 17.24 | 155 | 24.7 | NA | NA | NA | NA | NA | 0.158335 |
| Rodentia | Cricetidae | Peromyscus | caniceps | Peromyscus caniceps | NA | NA | 18.17 | 9 | 20.2 | 1 | NA | NA | NA | NA | 1.693332 |
| Rodentia | Muridae | Gerbillus | floweri | Gerbillus floweri | NA | NA | 18.22 | NA | NA | NA | NA | NA | NA | NA | 0.022232 |
| Chiroptera | Vespertilionidae | Eptesicus | dimissus | Eptesicus dimissus | 13.03 | NA | 18.82 | 154 | 26.9 | NA | NA | 1 | 2 | NA | 0.268274 |
| Chiroptera | Vespertilionidae | Nyctalus | furvus | Nyctalus furvus | NA | NA | 18.98 | 98 | 5.7 | NA | NA | NA | NA | NA | 0.786431 |
| Rodentia | Sciuridae | Sundasciurus | davensis | Sundasciurus davensis | NA | NA | 19.48 | 166.99 | 25.45 | NA | NA | NA | NA | NA | 0.330157 |
| Rodentia | Dipodidae | Sicista | armenica | Sicista armenica | NA | NA | 21.68 | 41 | 0.75 | NA | NA | NA | NA | NA | 1.757216 |
| Rodentia | Cricetidae | Oryzomys | galapagoensis | Oryzomys galapagoensis | 63.51 | NA | 23.33 | NA | NA | NA | NA | NA | NA | NA | 0.760988 |
| Chiroptera | Vespertilionidae | Arielulus | cuprosus | Arielulus cuprosus | NA | NA | 25.19 | 265 | 26.7 | NA | NA | 1 | 2 | NA | 0.302108 |
| Chiroptera | Hipposideridae | Triaenops | auritus | Triaenops auritus | NA | NA | 25.57 | 81 | 26.5 | NA | NA | NA | NA | NA | 0.982971 |
| Rodentia | Cricetidae | Peromyscus | slevini | Peromyscus slevini | NA | NA | 27.84 | NA | NA | 1 | NA | NA | NA | NA | 1.693332 |
| Diprotodontia | Phalangeridae | Spilocuscus | kraemeri | Spilocuscus kraemeri | NA | 1.02 | 29.6 | NA | NA | 1 | NA | 1 | 2 | NA | 0.177411 |
| Rodentia | Cricetidae | Reithrodontomys | raviventris | Reithrodontomys raviventris | 10.95 | 3.88 | 32.04 | 46.75 | 13.125 | 1 | 2 | 2 | 2 | 1 | 1.38209 |
| Chiroptera | Vespertilionidae | Pipistrellus | permixtus | Pipistrellus permixtus | NA | NA | 35.66 | 99 | 25.2 | NA | NA | 1 | 2 | NA | 0.298002 |
| Chiroptera | Pteropodidae | Otopteropus | cartilagonodus | Otopteropus cartilagonodus | 16.92 | 0.99 | 36.68 | 269 | 22.6 | NA | NA | 1 | 2 | NA | 0.017247 |
| Soricomorpha | Soricidae | Crocidura | macowi | Crocidura macowi | NA | NA | 36.99 | 54 | 24.6 | NA | NA | NA | NA | NA | 0.286234 |
| Rodentia | Gliridae | Chaetocauda | sichuanensis | Chaetocauda sichuanensis | 31.51 | NA | 37.63 | 69 | 13.2 | 1 | NA | 1 | 2 | NA | 0.366986 |
| Soricomorpha | Soricidae | Crocidura | macmillani | Crocidura macmillani | NA | NA | 38.85 | 64.5 | 18.45 | NA | NA | NA | NA | NA | 0.89448 |
| Rodentia | Cricetidae | Peromyscus | interparietalis | Peromyscus interparietalis | NA | 2.39 | 40.64 | NA | NA | 1 | NA | NA | NA | NA | 1.693332 |
| Rodentia | Cricetidae | Peromyscus | stephani | Peromyscus stephani | NA | NA | 41.35 | NA | NA | 1 | NA | NA | NA | NA | 1.693332 |
| Chiroptera | Vespertilionidae | Pipistrellus | minahassae | Pipistrellus minahassae | NA | NA | 42.25 | 225 | 23.1 | NA | NA | 1 | 2 | NA | 0.273138 |
| Rodentia | Muridae | Leporillus | conditor | Leporillus conditor | 328.82 | 2.95 | 43.48 | 24.94 | 18.646 | NA | 2 | NA | NA | 1 | 0.90762 |
| Rodentia | Muridae | Pseudomys | fieldi | Pseudomys fieldi | 42.35 | 3.49 | 45.74 | NA | NA | NA | 2 | NA | NA | 1 | 1.082473 |
| Afrosoricida | Chrysochloridae | Calcochloris | tytonis | Calcochloris tytonis | NA | NA | 47.65 | 35.5 | 26.8 | NA | 1 | NA | NA | 3 | 0.384834 |
| Rodentia | Muridae | Rattus | hainaldi | Rattus hainaldi | NA | NA | 49.95 | 225.5 | 22.7 | NA | NA | NA | NA | NA | 1.244493 |
| Rodentia | Muridae | Rattus | feliceus | Rattus feliceus | 306 | NA | 50.4 | 277 | 24.3 | NA | NA | NA | NA | NA | 0.165889 |
| Rodentia | Geomyidae | Orthogeomys | thaeleri | Orthogeomys thaeleri | NA | NA | 50.46 | 461 | 24.5 | NA | 3 | 2 | 1 | 1 | 0.015066 |
| Chiroptera | Nycteridae | Nycteris | vinsoni | Nycteris vinsoni | NA | NA | 52.88 | 75 | 23.8 | NA | NA | NA | NA | NA | 0.28518 |
| Rodentia | Cricetidae | Phaenomys | ferrugineus | Phaenomys ferrugineus | 93.8 | NA | 53.88 | 135 | 21.2 | NA | NA | 1 | 2 | NA | 0.990736 |
| Rodentia | Sciuridae | Prosciurillus | abstrusus | Prosciurillus abstrusus | NA | NA | 62.43 | 239 | 19.6 | NA | NA | NA | NA | NA | 0.307928 |
| Rodentia | Dipodidae | Sicista | kazbegica | Sicista kazbegica | NA | NA | 64.07 | 66 | -5.2 | NA | NA | NA | NA | NA | 1.757216 |
| Chiroptera | Molossidae | Otomops | wroughtoni | Otomops wroughtoni | NA | 1 | 64.59 | 185 | 27.1 | NA | 1 | 1 | 2 | 3 | 0.294193 |
| Rodentia | Muridae | Tarsomys | echinatus | Tarsomys echinatus | NA | NA | 68.88 | 86 | 23 | NA | NA | NA | NA | NA | 0.878526 |
| Chiroptera | Hipposideridae | Hipposideros | nequam | Hipposideros nequam | NA | NA | 68.95 | 167 | 26.7 | NA | NA | 1 | 2 | NA | 0.391718 |
| Chiroptera | Vespertilionidae | Glauconycteris | kenyacola | Glauconycteris kenyacola | 7.17 | NA | 70.18 | 66 | 27.2 | NA | NA | 1 | 2 | NA | 0.250694 |
| Rodentia | Muridae | Pseudomys | patrius | Pseudomys patrius | NA | NA | 70.99 | 89.5 | 22.2 | NA | NA | NA | NA | NA | 0.021649 |
| Chiroptera | Pteropodidae | Pteropus | pselaphon | Pteropus pselaphon | NA | 0.98 | 72.37 | NA | NA | NA | NA | 1 | 2 | NA | 1.441621 |
| Chiroptera | Pteropodidae | Pteropus | insularis | Pteropus insularis | NA | NA | 72.88 | NA | NA | NA | 1 | 1 | 2 | 1 | 0.242799 |
| Erinaceomorpha | Erinaceidae | Hylomys | parvus | Hylomys parvus | NA | NA | 73.08 | 343 | 17.4 | NA | NA | NA | NA | NA | 1.165428 |
| Soricomorpha | Soricidae | Crocidura | susiana | Crocidura susiana | NA | NA | 73.72 | 31 | 22.9 | NA | NA | NA | NA | NA | 0.286234 |
| Rodentia | Muridae | Crateromys | paulus | Crateromys paulus | NA | NA | 75.23 | 183 | 25.8 | NA | NA | NA | NA | NA | 0.283032 |
| Soricomorpha | Soricidae | Crocidura | polia | Crocidura polia | NA | NA | 77.81 | 151 | 24 | NA | NA | NA | NA | NA | 0.286234 |
| Soricomorpha | Soricidae | Crocidura | raineyi | Crocidura raineyi | 14.49 | NA | 78.45 | 72.99 | 21.549 | NA | NA | NA | NA | NA | 0.286234 |
| Rodentia | Cricetidae | Microtus | mujanensis | Microtus mujanensis | NA | NA | 80.08 | 30 | -9.2 | NA | NA | NA | NA | NA | 0.211513 |
| Lagomorpha | Leporidae | Lepus | insularis | Lepus insularis | NA | NA | 82.65 | 18 | 22.5 | NA | NA | NA | NA | NA | 0.119848 |
| Rodentia | Capromyidae | Mesocapromys | auritus | Mesocapromys auritus | NA | NA | 83.4 | 123 | 25 | NA | 3 | 1 | 2 | 2 | 1.35101 |
| Rodentia | Muridae | Pelomys | isseli | Pelomys isseli | 59.99 | NA | 85.9 | NA | NA | NA | NA | NA | NA | NA | 0.347679 |
| Chiroptera | Pteropodidae | Acerodon | humilis | Acerodon humilis | NA | NA | 87.45 | NA | NA | NA | NA | 1 | 2 | NA | 1.146688 |
| Afrosoricida | Tenrecidae | Microgale | dryas | Microgale dryas | 40 | NA | 87.5 | 201.95 | 19.6 | NA | NA | NA | NA | NA | 1.050671 |
| Afrosoricida | Chrysochloridae | Neamblysomus | gunningi | Neamblysomus gunningi | NA | NA | 87.66 | 67.99 | 22.1 | NA | 1 | 1 | 1 | 3 | 1.652785 |
| Diprotodontia | Macropodidae | Lagorchestes | hirsutus | Lagorchestes hirsutus | 1407.14 | 1 | 89.5 | NA | NA | 1 | 3 | 1 | 1 | 1 | 0.740072 |
| Peramelemorphia | Peramelidae | Perameles | bougainville | Perameles bougainville | 230.8 | 2 | 89.5 | NA | NA | 2 | 3 | 2 | 1 | 2 | 1.484951 |
| Soricomorpha | Soricidae | Crocidura | ichnusae | Crocidura ichnusae | NA | NA | 93.15 | NA | NA | NA | NA | NA | NA | NA | 0.01789 |
| Chiroptera | Rhinolophidae | Rhinolophus | mitratus | Rhinolophus mitratus | NA | NA | 95.38 | 111 | 24.549 | NA | NA | 1 | 2 | NA | 0.195316 |
| Rodentia | Muridae | Rattus | bontanus | Rattus bontanus | NA | NA | 96.13 | 236 | 21.5 | NA | NA | NA | NA | NA | 0.265644 |
| Diprotodontia | Pseudocheiridae | Pseudochirops | coronatus | Pseudochirops coronatus | 1395.85 | NA | 97 | 207 | 11.8 | 2 | 2 | 1 | 2 | 1 | 0.991358 |
| Chiroptera | Pteropodidae | Pteropus | yapensis | Pteropus yapensis | NA | NA | 98.55 | NA | NA | NA | NA | NA | NA | NA | 0.72961 |
| Chiroptera | Pteropodidae | Pteropus | ualanus | Pteropus ualanus | NA | NA | 103.24 | NA | NA | NA | NA | NA | NA | NA | 0.72961 |
| Primates | Lemuridae | Eulemur | cinereiceps | Eulemur cinereiceps | NA | NA | 103.56 | 114 | 24.1 | NA | NA | NA | NA | NA | 1.29028 |
| Rodentia | Cricetidae | Tylomys | fulviventer | Tylomys fulviventer | NA | NA | 105.37 | 226 | 25.3 | NA | NA | NA | NA | NA | 0.313437 |
| Chiroptera | Pteropodidae | Pteropus | rodricensis | Pteropus rodricensis | 256.17 | 1 | 111.5 | NA | NA | NA | 1 | 1 | 2 | 1 | 1.394496 |
| Primates | Tarsiidae | Tarsius | pumilus | Tarsius pumilus | NA | 1.01 | 111.58 | 222.32 | 20.198 | NA | NA | NA | NA | NA | 0.345452 |
| Diprotodontia | Petauridae | Petaurus | abidi | Petaurus abidi | 278 | 1.02 | 114.2 | 217.5 | 29.85 | 1 | 5 | 1 | 2 | 2 | 1.61797 |
| Soricomorpha | Soricidae | Crocidura | phaeura | Crocidura phaeura | NA | NA | 114.4 | 65 | 15.75 | NA | NA | NA | NA | NA | 1.34172 |
| Rodentia | Nesomyidae | Eliurus | penicillatus | Eliurus penicillatus | 100 | NA | 118.48 | 188.97 | 17.049 | NA | NA | NA | NA | NA | 1.294246 |
| Chiroptera | Pteropodidae | Pteropus | aldabrensis | Pteropus aldabrensis | 310.29 | NA | 123.35 | NA | NA | NA | NA | 1 | 2 | NA | 0.748106 |
| Rodentia | Sciuridae | Hylopetes | winstoni | Hylopetes winstoni | NA | NA | 123.43 | 141 | 22.4 | NA | NA | NA | NA | NA | 0.223809 |
| Soricomorpha | Soricidae | Myosorex | eisentrauti | Myosorex eisentrauti | NA | NA | 125.66 | NA | NA | NA | NA | NA | NA | NA | 1.928109 |
| Rodentia | Muridae | Rattus | palmarum | Rattus palmarum | NA | NA | 126.12 | NA | NA | NA | NA | NA | NA | NA | 0.698143 |
| Chiroptera | Vespertilionidae | Myotis | hermani | Myotis hermani | NA | NA | 126.25 | NA | NA | NA | NA | NA | NA | NA | 0.20388 |
| Soricomorpha | Soricidae | Crocidura | harenna | Crocidura harenna | NA | NA | 133.85 | 71 | -8.5 | NA | NA | NA | NA | NA | 1.699512 |
| Afrosoricida | Chrysochloridae | Chrysochloris | visagiei | Chrysochloris visagiei | NA | NA | 137.09 | 12.49 | 16.201 | NA | 1 | NA | NA | 3 | 0.363152 |
| Soricomorpha | Soricidae | Crocidura | trichura | Crocidura trichura | NA | NA | 138.04 | NA | NA | NA | NA | NA | NA | NA | 1.476138 |
| Chiroptera | Emballonuridae | Coleura | seychellensis | Coleura seychellensis | 10.64 | NA | 151.19 | NA | NA | 1 | 1 | 1 | 2 | 3 | 2.031605 |
| Soricomorpha | Soricidae | Crocidura | wimmeri | Crocidura wimmeri | 23.5 | NA | 152.56 | 167 | 26.2 | NA | NA | NA | NA | NA | 1.699512 |
| Rodentia | Cricetidae | Peromyscus | mayensis | Peromyscus mayensis | NA | NA | 153.21 | 81.5 | 19.25 | 1 | NA | NA | NA | NA | 1.693332 |
| Rodentia | Muridae | Taeromys | punicans | Taeromys punicans | NA | NA | 153.67 | 216 | 15.6 | NA | NA | NA | NA | NA | 0.31657 |
| Diprotodontia | Macropodidae | Dendrolagus | scottae | Dendrolagus scottae | 9958.58 | 1.01 | 153.85 | 232.49 | 29.825 | 2 | 2 | 2 | 2 | 1 | 1.304949 |
| Soricomorpha | Soricidae | Myosorex | schalleri | Myosorex schalleri | NA | NA | 153.9 | 93 | 16.65 | NA | NA | NA | NA | NA | 0.324734 |
| Rodentia | Dasyproctidae | Dasyprocta | ruatanica | Dasyprocta ruatanica | NA | NA | 157.49 | NA | NA | 3 | 3 | 1 | 1 | 1 | 1.366237 |
| Rodentia | Muridae | Rattus | baluensis | Rattus baluensis | 107.5 | NA | 158.06 | 227.5 | 23.65 | NA | 4 | NA | NA | 2 | 0.013963 |
| Soricomorpha | Soricidae | Crocidura | grandis | Crocidura grandis | NA | NA | 159.87 | 203 | 24.9 | NA | NA | NA | NA | NA | 0.286234 |
| Rodentia | Muridae | Rattus | mollicomulus | Rattus mollicomulus | NA | NA | 160.31 | 236 | 21.5 | NA | NA | NA | NA | NA | 0.698143 |
| Chiroptera | Pteropodidae | Alionycteris | paucidentata | Alionycteris paucidentata | 16.24 | NA | 168.3 | 187 | 20 | NA | NA | 1 | 2 | NA | 0.018086 |
| Lagomorpha | Leporidae | Sylvilagus | mansuetus | Sylvilagus mansuetus | NA | NA | 170.04 | 13 | 20.6 | NA | NA | 1 | 1 | NA | 0.18414 |
| Rodentia | Muridae | Rattus | vandeuseni | Rattus vandeuseni | 67.24 | 2.15 | 170.49 | 209 | 19.4 | NA | 3 | 1 | 1 | 2 | 1.516252 |
| Artiodactyla | Cervidae | Muntiacus | gongshanensis | Muntiacus gongshanensis | 18590.01 | NA | 170.54 | 188.44 | -2.162 | NA | 4 | NA | NA | 2 | 0.241029 |
| Rodentia | Muridae | Gerbillus | occiduus | Gerbillus occiduus | NA | NA | 177.99 | 10 | 17.7 | NA | NA | NA | NA | NA | 0.35571 |
| Chiroptera | Pteropodidae | Pteropus | tuberculatus | Pteropus tuberculatus | NA | NA | 182.24 | NA | NA | NA | NA | 1 | 2 | NA | 1.441621 |
| Rodentia | Muridae | Taeromys | taerae | Taeromys taerae | NA | 4.37 | 191.74 | 225 | 23.1 | NA | NA | NA | NA | NA | 0.31657 |
| Diprotodontia | Macropodidae | Dorcopsis | atrata | Dorcopsis atrata | 6198.87 | 1.01 | 193.45 | NA | NA | 2 | 5 | 1 | 1 | 2 | 1.50715 |
| Rodentia | Geomyidae | Orthogeomys | lanius | Orthogeomys lanius | 499.99 | NA | 197.18 | 135 | 10.4 | 1 | 3 | 2 | 1 | 1 | 1.431223 |
| Artiodactyla | Cervidae | Axis | kuhlii | Axis kuhlii | 54999.99 | 1.22 | 197.86 | NA | NA | 3 | 2 | NA | NA | 1 | 1.522304 |
| Rodentia | Dipodidae | Allactaga | firouzi | Allactaga firouzi | NA | NA | 200.77 | 12 | 9.5 | NA | NA | NA | NA | NA | 0.369988 |
| Soricomorpha | Soricidae | Crocidura | nimbae | Crocidura nimbae | NA | NA | 200.78 | 159 | 23.7 | NA | NA | NA | NA | NA | 0.178896 |
| Rodentia | Cricetidae | Tylomys | tumbalensis | Tylomys tumbalensis | 280 | NA | 208.64 | 107 | 23.3 | NA | NA | NA | NA | NA | 1.861034 |
| Rodentia | Muridae | Komodomys | rintjanus | Komodomys rintjanus | NA | NA | 210.95 | NA | NA | NA | NA | NA | NA | NA | 1.007063 |
| Carnivora | Mustelidae | Melogale | everetti | Melogale everetti | NA | 2 | 213.93 | 227.5 | 23.65 | 2 | 6 | 1 | 1 | 2 | 0.30684 |
| Rodentia | Muridae | Tateomys | macrocercus | Tateomys macrocercus | NA | NA | 214.08 | 209 | 16.4 | NA | NA | NA | NA | NA | 0.26777 |
| Primates | Lemuridae | Hapalemur | alaotrensis | Hapalemur alaotrensis | 1616.83 | NA | 214.53 | 207.35 | 19.165 | NA | NA | NA | NA | NA | 1.894996 |
| Rodentia | Muridae | Solomys | salamonis | Solomys salamonis | NA | NA | 225.94 | NA | NA | NA | 1 | NA | NA | 1 | 0.300671 |
| Rodentia | Muridae | Carpomys | phaeurus | Carpomys phaeurus | NA | NA | 230.12 | 316 | 18 | NA | NA | NA | NA | NA | 0.017626 |
| Dasyuromorphia | Dasyuridae | Pseudantechinus | mimulus | Pseudantechinus mimulus | NA | NA | 230.43 | 70 | 26.8 | NA | NA | NA | NA | NA | 1.011015 |
| Rodentia | Muridae | Rattus | osgoodi | Rattus osgoodi | NA | NA | 230.85 | 139 | 21.025 | NA | NA | NA | NA | NA | 0.013963 |
| Diprotodontia | Petauridae | Dactylopsila | tatei | Dactylopsila tatei | 252 | NA | 231.26 | 267 | 25.4 | 1 | 3 | 1 | 2 | 2 | 1.596227 |
| Rodentia | Calomyscidae | Calomyscus | hotsoni | Calomyscus hotsoni | NA | NA | 231.5 | 16.5 | 20.75 | NA | NA | NA | NA | NA | 0.019168 |
| Rodentia | Muridae | Taeromys | hamatus | Taeromys hamatus | NA | NA | 241.59 | 212.49 | 16 | NA | NA | NA | NA | NA | 0.31657 |
| Rodentia | Sciuridae | Biswamoyopterus | biswasi | Biswamoyopterus biswasi | NA | NA | 253.3 | 224.74 | 15.028 | NA | NA | NA | NA | NA | 1.265482 |
| Rodentia | Geomyidae | Geomys | tropicalis | Geomys tropicalis | 349.99 | 4 | 254.65 | NA | NA | NA | 4 | 2 | 1 | 1 | 1.350556 |
| Rodentia | Muridae | Rattus | hoogerwerfi | Rattus hoogerwerfi | NA | NA | 256.04 | 111.99 | 18.449 | NA | NA | NA | NA | NA | 0.835154 |
| Rodentia | Muridae | Rhynchomys | soricoides | Rhynchomys soricoides | NA | NA | 263.82 | 316 | 18 | NA | NA | NA | NA | NA | 0.123309 |
| Soricomorpha | Soricidae | Myosorex | blarina | Myosorex blarina | NA | NA | 268.27 | 123 | 15.8 | NA | NA | NA | NA | NA | 1.522191 |
| Peramelemorphia | Peramelidae | Echymipera | davidi | Echymipera davidi | 817.05 | NA | 280.1 | NA | NA | NA | NA | NA | NA | NA | 1.426228 |
| Rodentia | Muridae | Rattus | montanus | Rattus montanus | NA | NA | 291.53 | 180.99 | 22.399 | NA | NA | NA | NA | NA | 1.252731 |
| Chiroptera | Vespertilionidae | Myotis | cobanensis | Myotis cobanensis | NA | NA | 294.52 | 139.92 | 20.346 | NA | NA | 1 | 2 | NA | 0.276485 |
| Rodentia | Muridae | Melomys | arcium | Melomys arcium | NA | NA | 296.37 | NA | NA | NA | NA | NA | NA | NA | 0.259376 |
| Rodentia | Muridae | Maxomys | wattsi | Maxomys wattsi | NA | NA | 311.03 | 234 | 19 | NA | NA | NA | NA | NA | 1.609335 |
| Rodentia | Muridae | Microhydromys | musseri | Microhydromys musseri | NA | NA | 312.94 | 232.49 | 29.825 | NA | 1 | 1 | 1 | 3 | 0.311344 |
| Rodentia | Muridae | Margaretamys | elegans | Margaretamys elegans | NA | NA | 313.91 | 209 | 16.4 | NA | NA | NA | NA | NA | 0.176895 |
| Rodentia | Muridae | Margaretamys | parvus | Margaretamys parvus | NA | NA | 313.91 | 209 | 16.4 | NA | NA | NA | NA | NA | 0.283032 |
| Chiroptera | Pteropodidae | Pteropus | faunulus | Pteropus faunulus | NA | NA | 314.24 | NA | NA | NA | NA | 1 | 2 | NA | 0.919026 |
| Rodentia | Muridae | Macruromys | elegans | Macruromys elegans | NA | NA | 314.57 | 191.66 | 20.033 | NA | 2 | 1 | 1 | 1 | 0.311344 |
| Rodentia | Muridae | Coccymys | albidens | Coccymys albidens | NA | NA | 314.83 | 177 | 15.3 | NA | NA | 2 | 2 | NA | 0.311344 |
| Rodentia | Muridae | Pogonomys | championi | Pogonomys championi | 50.45 | 2.15 | 315.18 | 258 | 19.4 | 1 | 2 | 3 | 2 | 1 | 0.309682 |
| Rodentia | Muridae | Pithecheir | melanurus | Pithecheir melanurus | NA | NA | 316.12 | 215 | 23.2 | NA | NA | NA | NA | NA | 1.007063 |
| Rodentia | Muridae | Leptomys | signatus | Leptomys signatus | NA | NA | 317.56 | 241 | 26.2 | NA | 1 | NA | NA | 3 | 0.017095 |
| Rodentia | Muridae | Palawanomys | furvus | Palawanomys furvus | NA | NA | 318.13 | 233 | 24.3 | NA | NA | NA | NA | NA | 0.378963 |
| Rodentia | Muridae | Cremnomys | elvira | Cremnomys elvira | NA | NA | 320.43 | 81 | 27.3 | NA | NA | NA | NA | NA | 1.683825 |
| Rodentia | Nesomyidae | Macrotarsomys | ingens | Macrotarsomys ingens | 54.99 | NA | 321.5 | 129 | 26.45 | 1 | NA | 3 | 2 | NA | 1.551495 |
| Rodentia | Muridae | Bunomys | prolatus | Bunomys prolatus | NA | NA | 322.44 | 234 | 19 | NA | NA | NA | NA | NA | 1.467115 |
| Rodentia | Muridae | Chrotomys | gonzalesi | Chrotomys gonzalesi | 140.2 | NA | 323.24 | 331 | 25.2 | NA | 1 | NA | NA | 3 | 0.102874 |
| Chiroptera | Pteropodidae | Pteropus | fundatus | Pteropus fundatus | 210.67 | NA | 325.78 | NA | NA | NA | NA | 1 | 2 | NA | 1.122159 |
| Rodentia | Cricetidae | Microtus | abbreviatus | Microtus abbreviatus | 66.06 | 3 | 327.94 | NA | NA | NA | NA | NA | NA | NA | 0.01503 |
| Rodentia | Muridae | Crunomys | fallax | Crunomys fallax | NA | NA | 328.12 | 177 | 23.4 | NA | NA | NA | NA | NA | 0.18929 |
| Rodentia | Muridae | Archboldomys | luzonensis | Archboldomys luzonensis | 35.34 | NA | 328.3 | 331 | 25.2 | 3 | 1 | NA | NA | 3 | 0.627345 |
| Diprotodontia | Potoroidae | Bettongia | lesueur | Bettongia lesueur | 1447.27 | 1 | 329.35 | NA | NA | 1 | NA | NA | NA | NA | 0.173995 |
| Soricomorpha | Soricidae | Cryptotis | endersi | Cryptotis endersi | NA | NA | 335.8 | 270 | 35 | NA | NA | NA | NA | NA | 1.471915 |
| Rodentia | Muridae | Anonymomys | mindorensis | Anonymomys mindorensis | NA | NA | 340.54 | 220 | 22.7 | NA | NA | NA | NA | NA | 0.378963 |
| Rodentia | Muridae | Apomys | sacobianus | Apomys sacobianus | 34.88 | NA | 341.33 | 269.5 | 24.4 | 1 | NA | 2 | 2 | NA | 0.211549 |
| Rodentia | Muridae | Gerbillus | hoogstraali | Gerbillus hoogstraali | NA | NA | 341.84 | 17.49 | 15.31 | NA | NA | NA | NA | NA | 1.111594 |
| Rodentia | Echimyidae | Phyllomys | thomasi | Phyllomys thomasi | 502.14 | NA | 343.03 | NA | NA | 1 | NA | 2 | 2 | NA | 1.337274 |
| Rodentia | Muridae | Hydromys | habbema | Hydromys habbema | NA | NA | 345.8 | 170 | 13.7 | NA | NA | 3 | 1 | NA | 0.285607 |
| Rodentia | Muridae | Tarsomys | apoensis | Tarsomys apoensis | NA | NA | 347.14 | 159.22 | 22.474 | NA | NA | NA | NA | NA | 0.017571 |
| Soricomorpha | Soricidae | Crocidura | stenocephala | Crocidura stenocephala | NA | NA | 347.38 | 146.99 | 17.45 | NA | NA | NA | NA | NA | 1.34172 |
| Chiroptera | Pteropodidae | Pteropus | molossinus | Pteropus molossinus | NA | NA | 349.59 | NA | NA | NA | 2 | 1 | 2 | 1 | 0.733945 |
| Rodentia | Muridae | Abditomys | latidens | Abditomys latidens | 268.09 | NA | 356.58 | 316 | 18 | NA | NA | NA | NA | NA | 0.31657 |
| Rodentia | Muridae | Bunomys | fratrorum | Bunomys fratrorum | 130.31 | NA | 358.08 | 226 | 21.4 | NA | NA | NA | NA | NA | 0.978076 |
| Rodentia | Cricetidae | Habromys | chinanteco | Habromys chinanteco | 40 | NA | 360 | 123.99 | 25.145 | NA | NA | NA | NA | NA | 1.718845 |
| Soricomorpha | Talpidae | Uropsilus | investigator | Uropsilus investigator | NA | NA | 363.35 | 187.98 | 0.851 | NA | NA | NA | NA | NA | 0.383381 |
| Rodentia | Muridae | Batomys | dentatus | Batomys dentatus | NA | NA | 366.42 | 337.02 | 21.354 | NA | NA | NA | NA | NA | 0.283032 |
| Rodentia | Cricetidae | Microtus | evoronensis | Microtus evoronensis | NA | NA | 367.89 | 36.5 | -2.749 | NA | NA | NA | NA | NA | 0.211513 |
| Rodentia | Cricetidae | Neotoma | bryanti | Neotoma bryanti | 182 | NA | 369.55 | NA | NA | NA | NA | NA | NA | NA | 1.172788 |
| Didelphimorphia | Didelphidae | Marmosops | handleyi | Marmosops handleyi | 30.7 | NA | 373.34 | 145 | 18.9 | 1 | NA | NA | NA | NA | 2.149172 |
| Didelphimorphia | Didelphidae | Gracilinanus | aceramarcae | Gracilinanus aceramarcae | 20.5 | NA | 382.7 | 87.02 | 4.044 | 1 | NA | NA | NA | NA | 0.022595 |
| Rodentia | Echimyidae | Proechimys | mincae | Proechimys mincae | 284.99 | NA | 383.45 | 18 | 23.6 | NA | NA | NA | NA | NA | 0.330599 |
| Rodentia | Muridae | Rattus | timorensis | Rattus timorensis | NA | NA | 383.83 | 89.49 | 23.4 | NA | NA | NA | NA | NA | 0.265492 |
| Soricomorpha | Talpidae | Talpa | davidiana | Talpa davidiana | NA | 3.49 | 393.16 | 41 | 12.5 | 2 | 2 | 1 | 1 | 3 | 0.383762 |
| Rodentia | Muridae | Haeromys | margarettae | Haeromys margarettae | NA | NA | 398.16 | 283 | 24.8 | NA | NA | NA | NA | NA | 0.283636 |
| Chiroptera | Vespertilionidae | Myotis | findleyi | Myotis findleyi | NA | NA | 398.67 | NA | NA | NA | NA | 1 | 2 | NA | 1.189662 |
| Rodentia | Cricetidae | Neotoma | nelsoni | Neotoma nelsoni | 198 | NA | 401.2 | 135 | 10.4 | NA | NA | NA | NA | NA | 1.485531 |
| Rodentia | Muridae | Rattus | adustus | Rattus adustus | NA | NA | 402.7 | NA | NA | NA | NA | NA | NA | NA | 0.223406 |
| Rodentia | Muridae | Rattus | enganus | Rattus enganus | NA | NA | 402.7 | NA | NA | NA | NA | NA | NA | NA | 0.267249 |
| Rodentia | Muridae | Carpomys | melanurus | Carpomys melanurus | NA | NA | 404.03 | 316 | 18 | NA | NA | NA | NA | NA | 0.28202 |
| Rodentia | Muridae | Acomys | cilicicus | Acomys cilicicus | NA | NA | 404.16 | 48.02 | 15.41 | NA | NA | NA | NA | NA | 0.221612 |
| Didelphimorphia | Didelphidae | Marmosa | andersoni | Marmosa andersoni | 47.4 | NA | 412.04 | 61.99 | 9.699 | 1 | NA | NA | NA | NA | 0.399738 |
| Soricomorpha | Soricidae | Crocidura | picea | Crocidura picea | NA | NA | 413.32 | 216.5 | 22.15 | NA | NA | NA | NA | NA | 1.34172 |
| Rodentia | Cricetidae | Peromyscus | madrensis | Peromyscus madrensis | NA | 2.91 | 417.37 | NA | NA | 1 | NA | NA | NA | NA | 1.336841 |
| Lagomorpha | Leporidae | Sylvilagus | graysoni | Sylvilagus graysoni | 964.01 | NA | 417.37 | NA | NA | NA | NA | 1 | 1 | NA | 1.112757 |
| Soricomorpha | Soricidae | Chodsigoa | salenskii | Chodsigoa salenskii | 5.49 | NA | 424.87 | 73.5 | 14.901 | NA | NA | NA | NA | NA | 0.327334 |
| Chiroptera | Vespertilionidae | Myotis | anjouanensis | Myotis anjouanensis | NA | NA | 428.87 | NA | NA | NA | NA | NA | NA | NA | 0.195135 |
| Chiroptera | Vespertilionidae | Pharotis | imogene | Pharotis imogene | NA | NA | 439.62 | 283.94 | 16.039 | NA | NA | 1 | 2 | NA | 1.864173 |
| Chiroptera | Pteropodidae | Pteralopex | acrodonta | Pteralopex acrodonta | 257.53 | NA | 440.14 | NA | NA | NA | NA | 1 | 2 | NA | 1.63066 |
| Chiroptera | Vespertilionidae | Hypsugo | anthonyi | Hypsugo anthonyi | NA | NA | 447.91 | 210.47 | 19.052 | NA | NA | 1 | 2 | NA | 0.302108 |
| Chiroptera | Vespertilionidae | Murina | tenebrosa | Murina tenebrosa | NA | NA | 450.62 | NA | NA | NA | NA | 1 | 2 | NA | 1.49617 |
| Soricomorpha | Soricidae | Crocidura | telfordi | Crocidura telfordi | NA | NA | 451.92 | 73 | 24.6 | NA | NA | NA | NA | NA | 1.34172 |
| Chiroptera | Vespertilionidae | Hypsugo | joffrei | Hypsugo joffrei | NA | NA | 452.69 | 186.49 | 18.25 | NA | NA | 1 | 2 | NA | 0.302108 |
| Carnivora | Procyonidae | Procyon | pygmaeus | Procyon pygmaeus | 2958.03 | NA | 490.76 | 123 | 25.6 | 1 | 3 | 1 | 1 | 2 | 1.556748 |
| Rodentia | Cricetidae | Reithrodontomys | spectabilis | Reithrodontomys spectabilis | 20.19 | NA | 490.76 | 123 | 25.6 | 1 | NA | 2 | 2 | NA | 1.751339 |
| Rodentia | Dasyproctidae | Dasyprocta | coibae | Dasyprocta coibae | NA | NA | 503.74 | NA | NA | 3 | 3 | 1 | 1 | 1 | 0.910824 |
| Carnivora | Canidae | Urocyon | littoralis | Urocyon littoralis | 1923 | 2.17 | 511.92 | NA | NA | 2 | 6 | 1 | 1 | 2 | 1.317922 |
| Rodentia | Geomyidae | Cratogeomys | fumosus | Cratogeomys fumosus | 150 | NA | 512.65 | 97 | 20.8 | NA | 3 | 2 | 1 | 1 | 0.017788 |
| Chiroptera | Pteropodidae | Pteropus | nitendiensis | Pteropus nitendiensis | 275.56 | NA | 515.2 | NA | NA | NA | NA | 1 | 2 | NA | 1.138122 |
| Soricomorpha | Soricidae | Suncus | mertensi | Suncus mertensi | NA | NA | 518.99 | 199 | 23 | NA | NA | NA | NA | NA | 1.279622 |
| Rodentia | Muridae | Crunomys | celebensis | Crunomys celebensis | NA | NA | 525.31 | 205 | 18.15 | NA | NA | NA | NA | NA | 0.18929 |
| Artiodactyla | Suidae | Babyrousa | togeanensis | Babyrousa togeanensis | NA | NA | 536.65 | NA | NA | NA | NA | NA | NA | NA | 1.605084 |
| Peramelemorphia | Peramelidae | Echymipera | echinista | Echymipera echinista | 1204.61 | NA | 547.08 | 281 | 21.506 | NA | NA | NA | NA | NA | 0.304262 |
| Diprotodontia | Phalangeridae | Phalanger | matanim | Phalanger matanim | 1442.38 | 1.02 | 556.3 | 289.66 | 18.334 | 1 | 2 | 2 | 2 | 1 | 1.842736 |
| Chiroptera | Vespertilionidae | Myotis | oreias | Myotis oreias | NA | NA | 557.11 | 197 | 27.05 | NA | NA | 1 | 2 | NA | 0.231361 |
| Primates | Tarsiidae | Tarsius | sangirensis | Tarsius sangirensis | 165 | 1.02 | 560.08 | NA | NA | NA | NA | NA | NA | NA | 1.616223 |
| Primates | Indriidae | Propithecus | tattersalli | Propithecus tattersalli | 3531.39 | NA | 563.17 | 104.49 | 25.151 | 3 | 4 | 2 | 2 | 1 | 1.398157 |
| Chiroptera | Vespertilionidae | Myotis | stalkeri | Myotis stalkeri | NA | NA | 563.36 | NA | NA | NA | NA | 1 | 2 | NA | 0.259335 |
| Chiroptera | Pteropodidae | Latidens | salimalii | Latidens salimalii | NA | NA | 563.41 | 118.02 | 27.974 | NA | NA | 1 | 2 | NA | 1.356467 |
| Rodentia | Cricetidae | Nesoryzomys | swarthi | Nesoryzomys swarthi | NA | NA | 577.46 | NA | NA | NA | NA | NA | NA | NA | 0.713596 |
| Rodentia | Sciuridae | Cynomys | mexicanus | Cynomys mexicanus | 899.98 | 4.18 | 586.55 | 21.84 | 15.919 | 3 | 2 | 2 | 1 | 1 | 0.791814 |
| Rodentia | Cricetidae | Andalgalomys | olrogi | Andalgalomys olrogi | 32.9 | NA | 592.92 | 56.01 | 6.635 | NA | NA | 1 | 1 | NA | 0.013854 |
| Chiroptera | Vespertilionidae | Glauconycteris | machadoi | Glauconycteris machadoi | NA | NA | 599.87 | 104 | 22.4 | NA | NA | NA | NA | NA | 0.233188 |
| Rodentia | Geomyidae | Orthogeomys | cuniculus | Orthogeomys cuniculus | 499.99 | NA | 609.71 | 223.14 | 23.305 | 1 | 3 | 2 | 1 | 1 | 0.241048 |
| Rodentia | Muridae | Taeromys | arcuatus | Taeromys arcuatus | NA | NA | 610.45 | 239 | 18.799 | NA | NA | NA | NA | NA | 0.31657 |
| Rodentia | Gliridae | Dryomys | laniger | Dryomys laniger | 25.91 | 4.18 | 612.63 | 58.32 | 9.133 | 1 | 4 | 3 | 2 | 2 | 0.366986 |
| Rodentia | Sciuridae | Hylopetes | sipora | Hylopetes sipora | NA | NA | 620.62 | NA | NA | NA | NA | NA | NA | NA | 1.127695 |
| Rodentia | Muridae | Rattus | tawitawiensis | Rattus tawitawiensis | NA | NA | 625.54 | NA | NA | NA | 4 | NA | NA | 2 | 0.223406 |
| Chiroptera | Hipposideridae | Hipposideros | breviceps | Hipposideros breviceps | NA | NA | 630.6 | NA | NA | NA | 1 | 1 | 2 | 3 | 0.391718 |
| Rodentia | Muridae | Maxomys | alticola | Maxomys alticola | 158.96 | NA | 632.78 | 235.5 | 22.549 | NA | NA | NA | NA | NA | 0.021458 |
| Chiroptera | Pteropodidae | Pteropus | livingstonii | Pteropus livingstonii | 734.27 | 1 | 639.47 | NA | NA | NA | NA | 1 | 2 | NA | 1.10662 |
| Rodentia | Cricetidae | Nesoryzomys | fernandinae | Nesoryzomys fernandinae | NA | NA | 641.36 | 28 | 21.8 | NA | NA | NA | NA | NA | 0.713596 |
| Primates | Indriidae | Propithecus | perrieri | Propithecus perrieri | NA | NA | 661.89 | 90.5 | 25.25 | 3 | NA | NA | NA | NA | 1.765009 |
| Chiroptera | Vespertilionidae | Glischropus | javanus | Glischropus javanus | NA | NA | 665.21 | 345 | 22.9 | NA | NA | 1 | 2 | NA | 0.312186 |
| Rodentia | Muridae | Maxomys | dollmani | Maxomys dollmani | NA | NA | 665.57 | 229.66 | 16.665 | NA | NA | NA | NA | NA | 0.343325 |
| Rodentia | Cricetidae | Nesoryzomys | indefessus | Nesoryzomys indefessus | NA | NA | 666.88 | 28 | 21.8 | NA | NA | NA | NA | NA | 0.713596 |
| Soricomorpha | Soricidae | Crocidura | zimmeri | Crocidura zimmeri | 14.49 | NA | 671.11 | 104.5 | 25.65 | NA | NA | NA | NA | NA | 0.286234 |
| Chiroptera | Pteropodidae | Pteropus | rennelli | Pteropus rennelli | NA | NA | 679.88 | NA | NA | NA | NA | NA | NA | NA | 0.690153 |
| Rodentia | Muridae | Bunomys | coelestis | Bunomys coelestis | NA | NA | 680.95 | 234.66 | 21.765 | NA | NA | NA | NA | NA | 1.858345 |
| Rodentia | Sciuridae | Petaurillus | emiliae | Petaurillus emiliae | NA | NA | 683.58 | 278 | 22.7 | NA | NA | NA | NA | NA | 0.213518 |
| Rodentia | Muridae | Limnomys | sibuanus | Limnomys sibuanus | NA | NA | 695.52 | 163.62 | 22.964 | NA | NA | NA | NA | NA | 0.019786 |
| Rodentia | Muridae | Chrotomys | whiteheadi | Chrotomys whiteheadi | 151.98 | NA | 696.7 | 312.99 | 23.204 | NA | NA | NA | NA | NA | 0.010287 |
| Rodentia | Muridae | Paraleptomys | rufilatus | Paraleptomys rufilatus | 54.99 | NA | 700.58 | 219.99 | 29.966 | 2 | NA | 1 | 1 | NA | 1.291623 |
| Chiroptera | Pteropodidae | Pteropus | seychellensis | Pteropus seychellensis | 492.2 | 1 | 706.03 | NA | NA | NA | NA | 1 | 2 | NA | 0.014962 |
| Diprotodontia | Macropodidae | Lagostrophus | fasciatus | Lagostrophus fasciatus | 1933.48 | 0.9 | 708.99 | NA | NA | 1 | 4 | 1 | 1 | 1 | 1.559921 |
| Rodentia | Muridae | Tokudaia | osimensis | Tokudaia osimensis | 84.65 | NA | 710.54 | NA | NA | NA | NA | NA | NA | NA | 1.41709 |
| Rodentia | Muridae | Mallomys | gunung | Mallomys gunung | 2041.47 | NA | 710.81 | 178 | 14.066 | NA | 1 | 1 | 2 | 1 | 1.396981 |
| Soricomorpha | Soricidae | Paracrocidura | graueri | Paracrocidura graueri | NA | NA | 716.16 | 64.66 | 16.066 | NA | NA | NA | NA | NA | 0.251701 |
| Rodentia | Muridae | Hydromys | hussoni | Hydromys hussoni | NA | NA | 717.32 | 204.33 | 22.133 | NA | NA | 3 | 1 | NA | 0.285607 |
| Chiroptera | Pteropodidae | Pteralopex | pulchra | Pteralopex pulchra | 291.15 | NA | 728.08 | 200 | 24.75 | NA | NA | 1 | 2 | NA | 1.63066 |
| Rodentia | Muridae | Batomys | granti | Batomys granti | 169.53 | NA | 731.71 | 323.55 | 21.625 | NA | NA | NA | NA | NA | 0.176895 |
| Rodentia | Muridae | Chrotomys | silaceus | Chrotomys silaceus | NA | NA | 749.26 | 276.36 | 20.399 | NA | NA | NA | NA | NA | 0.012198 |
| Chiroptera | Pteropodidae | Pteropus | gilliardorum | Pteropus gilliardorum | 406.94 | NA | 749.41 | 206.64 | 23.332 | NA | NA | 1 | 2 | NA | 0.2935 |
| Rodentia | Geomyidae | Orthogeomys | cavator | Orthogeomys cavator | 650 | NA | 767.26 | 231.6 | 27.748 | NA | 3 | 2 | 1 | 1 | 0.015066 |
| Soricomorpha | Soricidae | Sylvisorex | morio | Sylvisorex morio | NA | 1.99 | 772.25 | 303 | 21.4 | NA | NA | NA | NA | NA | 1.522233 |
| Chiroptera | Pteropodidae | Epomophorus | grandis | Epomophorus grandis | 49.42 | NA | 775.34 | 115.22 | 24.525 | NA | 2 | 1 | 2 | 1 | 0.171767 |
| Soricomorpha | Soricidae | Suncus | ater | Suncus ater | NA | NA | 794.09 | 229 | 22.699 | NA | NA | NA | NA | NA | 0.272986 |
| Rodentia | Muridae | Dipodillus | jamesi | Dipodillus jamesi | NA | NA | 814.17 | 28.98 | 19.001 | NA | NA | NA | NA | NA | 0.35571 |
| Rodentia | Muridae | Paramelomys | levipes | Paramelomys levipes | 82.81 | 1.52 | 817.79 | 96 | 21.998 | NA | 3 | NA | NA | 1 | 0.32376 |
| Lagomorpha | Leporidae | Romerolagus | diazi | Romerolagus diazi | 465.58 | 2.1 | 820.7 | 67.5 | 13.75 | 2 | 2 | 2 | 1 | 1 | 1.765294 |
| Rodentia | Muridae | Crateromys | australis | Crateromys australis | NA | NA | 832.19 | NA | NA | NA | NA | NA | NA | NA | 1.680505 |
| Erinaceomorpha | Erinaceidae | Podogymnura | aureospinula | Podogymnura aureospinula | NA | NA | 832.19 | NA | NA | 1 | NA | NA | NA | NA | 1.750538 |
| Soricomorpha | Soricidae | Crocidura | mindorus | Crocidura mindorus | NA | NA | 836.7 | 194.5 | 23.5 | NA | NA | NA | NA | NA | 0.286234 |
| Rodentia | Muridae | Sundamys | maxi | Sundamys maxi | NA | NA | 849.1 | 253.5 | 20.3 | NA | NA | NA | NA | NA | 1.448223 |
| Rodentia | Muridae | Millardia | kondana | Millardia kondana | NA | NA | 852.02 | 72 | 24.3 | NA | NA | NA | NA | NA | 1.726648 |
| Soricomorpha | Soricidae | Crocidura | thomensis | Crocidura thomensis | NA | NA | 862.58 | NA | NA | NA | NA | NA | NA | NA | 1.34172 |
| Chiroptera | Hipposideridae | Hipposideros | thomensis | Hipposideros thomensis | NA | NA | 862.58 | NA | NA | NA | NA | NA | NA | NA | 0.020437 |
| Chiroptera | Pteropodidae | Myonycteris | brachycephala | Myonycteris brachycephala | NA | NA | 862.58 | NA | NA | NA | NA | 1 | 2 | NA | 1.198226 |
| Chiroptera | Vespertilionidae | Murina | grisea | Murina grisea | NA | NA | 867.06 | 171 | 21.9 | NA | NA | 1 | 2 | NA | 0.279752 |
| Soricomorpha | Soricidae | Myosorex | rumpii | Myosorex rumpii | NA | NA | 871.46 | 266.24 | 23.65 | NA | NA | NA | NA | NA | 1.522191 |
| Diprotodontia | Phalangeridae | Phalanger | lullulae | Phalanger lullulae | 1632.5 | 1.75 | 887.01 | NA | NA | 1 | 2 | 2 | 2 | 1 | 1.390169 |
| Chiroptera | Molossidae | Mops | niangarae | Mops niangarae | NA | NA | 893.11 | 159 | 23.9 | NA | NA | 1 | 2 | NA | 0.277788 |
| Chiroptera | Craseonycteridae | Craseonycteris | thonglongyai | Craseonycteris thonglongyai | 1.96 | 1 | 895.08 | 375.32 | 24.033 | 1 | 1 | 1 | 2 | 3 | 1.157573 |
| Rodentia | Cricetidae | Juscelinomys | candango | Juscelinomys candango | 97.29 | NA | 897.34 | 131 | 21.1 | NA | 2 | 1 | 1 | 2 | 1.981472 |
| Chiroptera | Pteropodidae | Pteropus | voeltzkowi | Pteropus voeltzkowi | 542.4 | 1 | 914.27 | NA | NA | NA | NA | 1 | 2 | NA | 0.748106 |
| Rodentia | Muridae | Haeromys | minahassae | Haeromys minahassae | NA | NA | 916.74 | 222 | 20.7 | NA | NA | NA | NA | NA | 0.886363 |
| Peramelemorphia | Peramelidae | Microperoryctes | murina | Microperoryctes murina | NA | NA | 927.71 | 199.5 | 14.147 | NA | NA | 2 | 1 | NA | 0.31616 |
| Rodentia | Muridae | Pseudohydromys | occidentalis | Pseudohydromys occidentalis | 21.9 | NA | 934.02 | 216.72 | 16.323 | NA | 2 | 1 | 1 | 2 | 0.275546 |
| Rodentia | Cricetidae | Peromyscus | guardia | Peromyscus guardia | NA | NA | 937.72 | 11 | 18.5 | 1 | NA | NA | NA | NA | 1.693332 |
| Soricomorpha | Soricidae | Crocidura | gracilipes | Crocidura gracilipes | 6.69 | 2.99 | 941.78 | 68.99 | 18.6 | NA | NA | NA | NA | NA | 0.286234 |
| Rodentia | Sciuridae | Sundasciurus | hoogstraali | Sundasciurus hoogstraali | NA | NA | 949.39 | NA | NA | NA | NA | NA | NA | NA | 0.020635 |
| Soricomorpha | Soricidae | Myosorex | babaulti | Myosorex babaulti | NA | 2.99 | 949.78 | 125.49 | 16.85 | NA | NA | NA | NA | NA | 0.202959 |
| Afrosoricida | Chrysochloridae | Amblysomus | marleyi | Amblysomus marleyi | NA | NA | 952.47 | 74.66 | 20.901 | NA | NA | NA | NA | NA | 1.743387 |
| Soricomorpha | Soricidae | Crocidura | orii | Crocidura orii | NA | NA | 960.37 | NA | NA | NA | NA | NA | NA | NA | 1.34172 |
| Lagomorpha | Leporidae | Pentalagus | furnessi | Pentalagus furnessi | NA | 2.44 | 960.37 | NA | NA | 1 | 3 | 2 | 1 | 1 | 1.769024 |
| Soricomorpha | Soricidae | Crocidura | nicobarica | Crocidura nicobarica | NA | 3 | 973.63 | NA | NA | NA | 2 | NA | NA | 3 | 1.699512 |
| Dasyuromorphia | Dasyuridae | Dasyurus | spartacus | Dasyurus spartacus | 885.67 | 6.78 | 990.45 | 153.32 | 26.066 | 2 | 5 | 1 | 1 | 2 | 0.115077 |
| Rodentia | Muridae | Eropeplus | canus | Eropeplus canus | NA | NA | 1002.75 | 207.99 | 17.3 | NA | NA | NA | NA | NA | 1.007063 |
| Soricomorpha | Soricidae | Crocidura | eisentrauti | Crocidura eisentrauti | NA | 1.99 | 1023.13 | 303 | 21.4 | NA | NA | NA | NA | NA | 0.89448 |
| Rodentia | Muridae | Dipodillus | maghrebi | Dipodillus maghrebi | NA | NA | 1026.27 | 59.47 | 15.803 | NA | NA | NA | NA | NA | 0.022232 |
| Chiroptera | Pteropodidae | Pteropus | mariannus | Pteropus mariannus | 458.58 | NA | 1030.33 | NA | NA | NA | 3 | 1 | 2 | 1 | 1.094415 |
| Chiroptera | Hipposideridae | Hipposideros | coronatus | Hipposideros coronatus | NA | NA | 1063.57 | 281 | 22 | NA | 1 | 1 | 2 | 3 | 0.336556 |
| Rodentia | Muridae | Chiropodomys | muroides | Chiropodomys muroides | 28.9 | NA | 1113.96 | 241.06 | 22.16 | NA | NA | NA | NA | NA | 0.291415 |
| Scandentia | Tupaiidae | Tupaia | nicobarica | Tupaia nicobarica | 170.25 | 0.96 | 1115.8 | NA | NA | NA | NA | NA | NA | NA | 1.760579 |
| Chiroptera | Vespertilionidae | Eptesicus | tatei | Eptesicus tatei | NA | NA | 1133.48 | 187.76 | 17.09 | NA | NA | 1 | 2 | NA | 0.268274 |
| Rodentia | Cricetidae | Tylomys | bullaris | Tylomys bullaris | 280 | NA | 1184.64 | 67.5 | 19.1 | NA | NA | NA | NA | NA | 1.861034 |
| Rodentia | Geomyidae | Orthogeomys | heterodus | Orthogeomys heterodus | 615 | NA | 1199.44 | 238.21 | 21.822 | NA | 3 | 2 | 1 | 1 | 0.015066 |
| Rodentia | Muridae | Tokudaia | muenninki | Tokudaia muenninki | NA | NA | 1209.87 | NA | NA | NA | NA | NA | NA | NA | 1.794981 |
| Rodentia | Cricetidae | Podoxymys | roraimae | Podoxymys roraimae | 33.79 | NA | 1239.18 | 144.33 | 19.5 | NA | NA | NA | NA | NA | 0.990736 |
| Rodentia | Sciuridae | Iomys | sipora | Iomys sipora | NA | NA | 1251.23 | NA | NA | NA | NA | NA | NA | NA | 1.090416 |
| Lagomorpha | Ochotonidae | Ochotona | argentata | Ochotona argentata | NA | NA | 1253.73 | 11.99 | 6.68 | NA | NA | NA | NA | NA | 1.42046 |
| Rodentia | Sciuridae | Petinomys | crinitus | Petinomys crinitus | NA | NA | 1254.65 | NA | NA | 1 | 4 | NA | NA | 2 | 0.01297 |
| Rodentia | Muridae | Rattus | burrus | Rattus burrus | NA | NA | 1257.84 | NA | NA | NA | NA | NA | NA | NA | 1.047214 |
| Rodentia | Dipodidae | Sicista | caucasica | Sicista caucasica | NA | NA | 1258.53 | 91.06 | 5.319 | NA | NA | NA | NA | NA | 1.171477 |
| Chiroptera | Vespertilionidae | Nyctophilus | heran | Nyctophilus heran | NA | NA | 1261.04 | NA | NA | NA | NA | 1 | 2 | NA | 0.257632 |
| Rodentia | Cricetidae | Nectomys | rattus | Nectomys rattus | 248.8 | NA | 1283.17 | 307.99 | 24.299 | 1 | 3 | 1 | 1 | 2 | 0.016069 |
| Rodentia | Sciuridae | Spermophilus | brunneus | Spermophilus brunneus | 300 | 7.5 | 1287.06 | 33.43 | 6.168 | NA | 8 | 2 | 1 | 2 | 0.851971 |
| Soricomorpha | Soricidae | Crocidura | longipes | Crocidura longipes | 19.3 | NA | 1301.98 | 74.75 | 27.649 | NA | NA | NA | NA | NA | 0.286234 |
| Rodentia | Heteromyidae | Heteromys | oresterus | Heteromys oresterus | 74.8 | 3.41 | 1335.88 | 215.68 | 19.334 | NA | 2 | 1 | 1 | 1 | 0.014035 |
| Artiodactyla | Cervidae | Axis | calamianensis | Axis calamianensis | 39812.6 | 2 | 1345.4 | NA | NA | 3 | NA | NA | NA | NA | 1.201819 |
| Rodentia | Cricetidae | Neusticomys | oyapocki | Neusticomys oyapocki | 47 | NA | 1365.89 | 246.74 | 24.35 | NA | 1 | 1 | NA | 3 | 0.267691 |
| Rodentia | Muridae | Gerbillus | grobbeni | Gerbillus grobbeni | NA | NA | 1373.45 | 27.32 | 18.267 | NA | NA | NA | NA | NA | 0.35571 |
| Carnivora | Procyonidae | Bassaricyon | pauli | Bassaricyon pauli | 1200 | NA | 1402.79 | 233 | 29.537 | 1 | NA | 1 | 1 | NA | 0.321695 |
| Scandentia | Tupaiidae | Tupaia | moellendorffi | Tupaia moellendorffi | NA | NA | 1403.25 | NA | NA | NA | NA | NA | NA | NA | 0.335604 |
| Primates | Cercopithecidae | Presbytis | natunae | Presbytis natunae | NA | NA | 1445.56 | 261 | 26.8 | NA | NA | NA | NA | NA | 0.585162 |
| Rodentia | Sciuridae | Sundasciurus | moellendorffi | Sundasciurus moellendorffi | NA | NA | 1467.72 | NA | NA | NA | NA | NA | NA | NA | 0.206348 |
| Chiroptera | Vespertilionidae | Eptesicus | guadeloupensis | Eptesicus guadeloupensis | NA | NA | 1476.99 | NA | NA | NA | NA | 1 | 2 | NA | 0.838358 |
| Rodentia | Cricetidae | Graomys | edithae | Graomys edithae | 40.4 | NA | 1501.77 | 31.5 | 15.946 | NA | NA | NA | NA | NA | 0.231375 |
| Rodentia | Geomyidae | Pappogeomys | alcorni | Pappogeomys alcorni | 150 | NA | 1519.92 | 83.25 | 18.75 | NA | 3 | 2 | 1 | 1 | 1.689839 |
| Chiroptera | Natalidae | Chilonatalus | tumidifrons | Chilonatalus tumidifrons | 3.62 | NA | 1525.32 | NA | NA | NA | 1 | 1 | 2 | 3 | 0.210168 |
| Chiroptera | Pteropodidae | Neopteryx | frosti | Neopteryx frosti | NA | 0.98 | 1526.23 | 219 | 19.026 | NA | NA | 1 | 2 | NA | 1.364274 |
| Soricomorpha | Soricidae | Crocidura | hispida | Crocidura hispida | NA | NA | 1535.89 | 215 | 26.8 | NA | NA | NA | NA | NA | 0.89448 |
| Rodentia | Muridae | Praomys | lukolelae | Praomys lukolelae | 42.2 | NA | 1546.35 | 162 | 26.025 | 1 | NA | 1 | 1 | NA | 0.019355 |
| Chiroptera | Rhinolophidae | Rhinolophus | nereis | Rhinolophus nereis | NA | NA | 1549.63 | 261 | 26.8 | NA | 1 | 1 | 2 | 3 | 0.158335 |
| Rodentia | Heteromyidae | Heteromys | nelsoni | Heteromys nelsoni | 67.55 | NA | 1561.5 | 134.07 | 21.744 | NA | 2 | 1 | 1 | 1 | 1.107921 |
| Chiroptera | Rhinolophidae | Rhinolophus | adami | Rhinolophus adami | NA | NA | 1569.81 | 129.5 | 24.049 | NA | NA | 1 | 2 | NA | 0.195316 |
| Primates | Cercopithecidae | Piliocolobus | kirkii | Piliocolobus kirkii | 7158.29 | 1.01 | 1573.03 | NA | NA | 3 | 4 | 1 | 2 | 1 | 1.172315 |
| Soricomorpha | Soricidae | Surdisorex | polulus | Surdisorex polulus | 8.99 | 1.49 | 1579.95 | 58 | 16.566 | NA | NA | NA | NA | NA | 0.886938 |
| Chiroptera | Phyllostomidae | Chiroderma | improvisum | Chiroderma improvisum | 35.39 | NA | 1580.07 | NA | NA | NA | 1 | 1 | 2 | 1 | 0.637534 |
| Chiroptera | Phyllostomidae | Sturnira | thomasi | Sturnira thomasi | NA | NA | 1580.07 | NA | NA | NA | 1 | 1 | 2 | 1 | 0.769557 |
| Chiroptera | Vespertilionidae | Myotis | martiniquensis | Myotis martiniquensis | NA | NA | 1587.98 | NA | NA | NA | NA | 1 | 2 | NA | 0.793108 |
| Rodentia | Cricetidae | Microtus | oaxacensis | Microtus oaxacensis | 36.52 | 1.2 | 1590.42 | 117 | 22.1 | NA | NA | NA | NA | NA | 1.217926 |
| Chiroptera | Pteropodidae | Rousettus | obliviosus | Rousettus obliviosus | 45.32 | 0.98 | 1677.4 | NA | NA | NA | NA | 1 | 2 | NA | 0.513763 |
| Rodentia | Muridae | Protochromys | fellowsi | Protochromys fellowsi | 78.99 | 2.68 | 1684.66 | 365.65 | 13.735 | NA | 3 | NA | NA | 1 | 0.020235 |
| Rodentia | Sciuridae | Spermophilus | musicus | Spermophilus musicus | NA | NA | 1698.02 | 48.51 | -0.79 | NA | NA | NA | NA | NA | 0.103729 |
| Rodentia | Geomyidae | Zygogeomys | trichopus | Zygogeomys trichopus | 473.54 | NA | 1706.58 | 84.67 | 17.637 | NA | 4 | 2 | 1 | 1 | 1.400441 |
| Chiroptera | Vespertilionidae | Myotis | insularum | Myotis insularum | NA | NA | 1746.15 | NA | NA | NA | NA | 1 | 2 | NA | 0.256739 |
| Rodentia | Cricetidae | Abrothrix | hershkovitzi | Abrothrix hershkovitzi | NA | NA | 1775.72 | 35.19 | 3.626 | NA | NA | NA | NA | NA | 0.016032 |
| Rodentia | Cricetidae | Rheomys | raptor | Rheomys raptor | 38 | 0.97 | 1835.2 | 194.5 | 24.824 | NA | NA | 2 | 1 | NA | 0.016404 |
| Chiroptera | Vespertilionidae | Miniopterus | robustior | Miniopterus robustior | NA | NA | 1837.63 | NA | NA | NA | NA | 1 | 2 | NA | 1.342749 |
| Rodentia | Muridae | Rattus | simalurensis | Rattus simalurensis | NA | NA | 1843.55 | NA | NA | NA | NA | NA | NA | NA | 1.047214 |
| Soricomorpha | Soricidae | Congosorex | polli | Congosorex polli | NA | NA | 1849.85 | 138.49 | 23.85 | NA | NA | NA | NA | NA | 0.325817 |
| Primates | Lemuridae | Eulemur | albocollaris | Eulemur albocollaris | 2397.37 | NA | 1864.78 | 187.78 | 21.739 | NA | NA | NA | NA | NA | 0.27526 |
| Chiroptera | Pteropodidae | Pteropus | niger | Pteropus niger | 474.43 | 1 | 1882.16 | 174 | 23 | NA | NA | 1 | 2 | NA | 1.122159 |
| Rodentia | Muridae | Microhydromys | richardsoni | Microhydromys richardsoni | 10.25 | NA | 1886.09 | 263.67 | 20.655 | NA | 1 | 1 | 1 | 3 | 0.311344 |
| Rodentia | Muridae | Grammomys | caniceps | Grammomys caniceps | NA | NA | 1893.85 | 46.66 | 26.833 | NA | NA | NA | NA | NA | 0.324293 |
| Primates | Galagidae | Galago | alleni | Galago alleni | 266.03 | 1.29 | 1953.16 | NA | NA | 1 | 2 | 2 | 2 | 1 | 0.020355 |
| Rodentia | Muridae | Hybomys | basilii | Hybomys basilii | NA | NA | 1953.16 | NA | NA | NA | NA | NA | NA | NA | 1.36626 |
| Soricomorpha | Soricidae | Sylvisorex | isabellae | Sylvisorex isabellae | NA | NA | 1953.16 | NA | NA | NA | NA | NA | NA | NA | 1.522233 |
| Rodentia | Muridae | Grammomys | gigas | Grammomys gigas | NA | NA | 1961.7 | 62.5 | 14.75 | NA | NA | NA | NA | NA | 1.520125 |
| Rodentia | Ctenomyidae | Ctenomys | sociabilis | Ctenomys sociabilis | 400 | NA | 1976.67 | 60.81 | 6.204 | NA | NA | NA | NA | NA | 1.607236 |
| Rodentia | Muridae | Melasmothrix | naso | Melasmothrix naso | NA | NA | 2059.07 | 223.49 | 16.425 | NA | NA | NA | NA | NA | 0.283032 |
| Soricomorpha | Soricidae | Sorex | leucogaster | Sorex leucogaster | NA | NA | 2063.39 | 114 | 1.1 | NA | NA | NA | NA | NA | 0.293879 |
| Artiodactyla | Bovidae | Hemitragus | hylocrius | Hemitragus hylocrius | 74186.41 | 1.41 | 2109.89 | 146.23 | 25.975 | 2 | 2 | 1 | 1 | 1 | 1.054269 |
| Primates | Cercopithecidae | Macaca | pagensis | Macaca pagensis | 4534.66 | 1 | 2155.21 | NA | NA | 3 | 4 | 1 | 2 | 2 | 1.151435 |
| Scandentia | Tupaiidae | Tupaia | chrysogaster | Tupaia chrysogaster | NA | NA | 2155.21 | NA | NA | NA | NA | NA | NA | NA | 1.760579 |
| Rodentia | Muridae | Diplothrix | legata | Diplothrix legata | NA | NA | 2170.25 | NA | NA | NA | NA | NA | NA | NA | 1.776388 |
| Soricomorpha | Talpidae | Mogera | tokudae | Mogera tokudae | 133.48 | 3.26 | 2190.11 | 181.1 | 11.399 | 2 | 2 | 1 | 1 | 2 | 1.570639 |
| Rodentia | Heteromyidae | Dipodomys | gravipes | Dipodomys gravipes | 83.96 | NA | 2231.05 | 18.5 | 14.004 | 1 | NA | 2 | 1 | NA | 1.272688 |
| Rodentia | Sciuridae | Tamias | palmeri | Tamias palmeri | 60.74 | 3.93 | 2237.62 | 4.79 | 14.22 | 3 | 5 | 1 | 2 | 2 | 0.857187 |
| Chiroptera | Vespertilionidae | Nyctalus | azoreum | Nyctalus azoreum | NA | NA | 2241.25 | NA | NA | NA | NA | 1 | 2 | NA | 1.186765 |
| Chiroptera | Vespertilionidae | Myotis | dominicensis | Myotis dominicensis | NA | NA | 2248.11 | NA | NA | NA | NA | 1 | 2 | NA | 0.793108 |
| Rodentia | Capromyidae | Mysateles | meridionalis | Mysateles meridionalis | NA | NA | 2253.65 | 95 | 25 | NA | NA | NA | NA | NA | 1.661717 |
| Carnivora | Mustelidae | Mustela | felipei | Mustela felipei | 211.3 | NA | 2258.77 | 113.99 | 13.1 | NA | NA | 1 | 1 | NA | 0.747572 |
| Soricomorpha | Soricidae | Crocidura | watasei | Crocidura watasei | NA | NA | 2267.41 | NA | NA | NA | NA | NA | NA | NA | 0.016053 |
| Chiroptera | Hipposideridae | Hipposideros | corynophyllus | Hipposideros corynophyllus | 15.07 | NA | 2289.85 | 281.74 | 18.6 | NA | NA | 1 | 2 | NA | 0.322153 |
| Rodentia | Muridae | Rattus | morotaiensis | Rattus morotaiensis | 148.25 | NA | 2299.31 | 174 | 24.9 | NA | NA | NA | NA | NA | 0.016589 |
| Soricomorpha | Soricidae | Crocidura | beccarii | Crocidura beccarii | NA | NA | 2324.82 | 222.33 | 22.066 | NA | NA | NA | NA | NA | 0.01789 |
| Rodentia | Cricetidae | Handleyomys | intectus | Handleyomys intectus | 60.5 | NA | 2333.17 | 138.74 | 17.599 | NA | NA | NA | NA | NA | 0.01522 |
| Rodentia | Dipodidae | Sicista | severtzovi | Sicista severtzovi | NA | NA | 2345.16 | 35.99 | 4.174 | NA | NA | NA | NA | NA | 0.02343 |
| Chiroptera | Hipposideridae | Hipposideros | macrobullatus | Hipposideros macrobullatus | NA | NA | 2348.21 | 256.52 | 22.7 | NA | 1 | 1 | 2 | 3 | 0.391718 |
| Rodentia | Muridae | Rattus | koopmani | Rattus koopmani | NA | NA | 2364.95 | 245 | 24.05 | NA | NA | NA | NA | NA | 0.223406 |
| Rodentia | Muridae | Rattus | pelurus | Rattus pelurus | NA | NA | 2364.95 | 245 | 24.05 | NA | NA | NA | NA | NA | 0.265644 |
| Primates | Tarsiidae | Tarsius | pelengensis | Tarsius pelengensis | 165 | 1.02 | 2364.95 | 245 | 24.05 | NA | NA | NA | NA | NA | 1.616223 |
| Chiroptera | Emballonuridae | Emballonura | semicaudata | Emballonura semicaudata | 6.29 | NA | 2369.52 | NA | NA | 1 | NA | 1 | 2 | NA | 1.79963 |
| Rodentia | Muridae | Rattus | stoicus | Rattus stoicus | NA | NA | 2371.86 | NA | NA | NA | NA | NA | NA | NA | 0.835154 |
| Rodentia | Cricetidae | Akodon | lindberghi | Akodon lindberghi | 26.4 | NA | 2400.72 | 127.74 | 20.824 | NA | NA | NA | NA | NA | 0.215868 |
| Primates | Cercopithecidae | Trachypithecus | poliocephalus | Trachypithecus poliocephalus | NA | NA | 2402.32 | 155.82 | 21.161 | 3 | NA | NA | NA | NA | 1.082966 |
| Rodentia | Ctenomyidae | Ctenomys | tuconax | Ctenomys tuconax | 248.99 | NA | 2421.97 | 71.26 | 13.409 | NA | 3 | 1 | 1 | 1 | 0.270692 |
| Chiroptera | Hipposideridae | Hipposideros | crumeniferus | Hipposideros crumeniferus | NA | NA | 2424.77 | 97.99 | 24.52 | NA | NA | 1 | 2 | NA | 0.336556 |
| Rodentia | Cricetidae | Peromyscus | winkelmanni | Peromyscus winkelmanni | 40 | NA | 2463.17 | 104.5 | 21.427 | 1 | NA | NA | NA | NA | 1.336841 |
| Soricomorpha | Soricidae | Myosorex | geata | Myosorex geata | 9.34 | 4 | 2470.49 | 67 | 24.1 | NA | NA | NA | NA | NA | 1.522191 |
| Lagomorpha | Leporidae | Sylvilagus | cognatus | Sylvilagus cognatus | NA | NA | 2477.48 | 21.81 | 10.361 | NA | NA | NA | NA | NA | 0.241861 |
| Soricomorpha | Soricidae | Crocidura | canariensis | Crocidura canariensis | 7.5 | NA | 2488.94 | NA | NA | NA | NA | NA | NA | NA | 1.34172 |
| Rodentia | Muridae | Melomys | fraterculus | Melomys fraterculus | NA | NA | 2505.38 | 280.5 | 23.35 | NA | NA | NA | NA | NA | 1.922328 |
| Peramelemorphia | Peramelidae | Rhynchomeles | prattorum | Rhynchomeles prattorum | NA | NA | 2513.87 | 280.5 | 23.35 | NA | NA | NA | NA | NA | 1.482 |
| Soricomorpha | Soricidae | Myosorex | longicaudatus | Myosorex longicaudatus | NA | NA | 2520.98 | 72.75 | 13.75 | NA | 2 | 1 | 2 | 2 | 1.014794 |
| Diprotodontia | Petauridae | Petaurus | biacensis | Petaurus biacensis | 90.98 | 1.45 | 2550.53 | 223 | 25.5 | NA | 5 | 1 | 2 | 2 | 0.016972 |
| Chiroptera | Vespertilionidae | Scotophilus | borbonicus | Scotophilus borbonicus | 19.02 | 2 | 2553.73 | 89 | 19.5 | 1 | NA | 1 | 2 | NA | 0.218851 |
| Chiroptera | Vespertilionidae | Miniopterus | fuscus | Miniopterus fuscus | NA | NA | 2590.39 | NA | NA | NA | NA | 1 | 2 | NA | 1.295496 |
| Rodentia | Cricetidae | Scolomys | ucayalensis | Scolomys ucayalensis | 26.5 | NA | 2596.26 | 211.75 | 25.95 | NA | NA | NA | NA | NA | 0.017303 |
| Chiroptera | Hipposideridae | Hipposideros | coxi | Hipposideros coxi | NA | NA | 2636.06 | 280.74 | 25.125 | NA | NA | 1 | 2 | NA | 0.391718 |
| Chiroptera | Pteropodidae | Dobsonia | emersa | Dobsonia emersa | 201.06 | NA | 2639.5 | 223 | 25.5 | NA | NA | 1 | 2 | NA | 0.563127 |
| Rodentia | Cricetidae | Microtus | umbrosus | Microtus umbrosus | 41.99 | NA | 2683.32 | 95.73 | 18.124 | NA | NA | NA | NA | NA | 1.217926 |
| Rodentia | Muridae | Nesoromys | ceramicus | Nesoromys ceramicus | NA | NA | 2716.44 | 280.5 | 23.35 | NA | NA | NA | NA | NA | 1.516252 |
| Primates | Cheirogaleidae | Microcebus | myoxinus | Microcebus myoxinus | 31.23 | 2 | 2721.78 | 82.26 | 24.75 | 1 | 4 | 1 | 2 | 2 | 0.324691 |
| Rodentia | Muridae | Melomys | obiensis | Melomys obiensis | 71 | NA | 2726.69 | 280 | 24.9 | NA | NA | NA | NA | NA | 0.020235 |
| Rodentia | Sciuridae | Tamiasciurus | mearnsi | Tamiasciurus mearnsi | NA | NA | 2768.05 | 11 | 15.819 | NA | NA | NA | NA | NA | 1.103877 |
| Rodentia | Cricetidae | Oryzomys | dimidiatus | Oryzomys dimidiatus | NA | NA | 2792.35 | 295.75 | 25.099 | NA | NA | NA | NA | NA | 0.01522 |
| Rodentia | Nesomyidae | Dendroprionomys | rousseloti | Dendroprionomys rousseloti | NA | NA | 2830.43 | 116.19 | 24 | NA | NA | NA | NA | NA | 0.431721 |
| Artiodactyla | Suidae | Sus | bucculentus | Sus bucculentus | NA | NA | 2834.16 | 150.27 | 20.274 | NA | NA | NA | NA | NA | 0.296276 |
| Rodentia | Muridae | Lemniscomys | hoogstraali | Lemniscomys hoogstraali | NA | NA | 2866.62 | 59.25 | 26.374 | NA | NA | NA | NA | NA | 0.311981 |
| Rodentia | Muridae | Pseudohydromys | murinus | Pseudohydromys murinus | 16.79 | 0.97 | 2880.34 | 328.75 | 13.567 | NA | 3 | 1 | 1 | 2 | 0.017222 |
| Primates | Cheirogaleidae | Phaner | electromontis | Phaner electromontis | NA | NA | 2890.84 | 98.77 | 25.36 | NA | NA | NA | NA | NA | 1.142985 |
| Rodentia | Muridae | Rattus | elaphinus | Rattus elaphinus | 131.35 | NA | 2894.95 | 263 | 23.5 | NA | NA | NA | NA | NA | 0.165889 |
| Rodentia | Cricetidae | Peromyscus | bullatus | Peromyscus bullatus | 40 | NA | 2913.33 | 129.59 | 14.435 | 1 | NA | NA | NA | NA | 1.693332 |
| Rodentia | Muridae | Pogonomys | fergussoniensis | Pogonomys fergussoniensis | NA | NA | 2938.84 | 267 | 25.4 | NA | NA | NA | NA | NA | 1.324108 |
| Rodentia | Muridae | Niviventer | hinpoon | Niviventer hinpoon | NA | NA | 2945.56 | 120.76 | 25.85 | NA | NA | NA | NA | NA | 0.343325 |
| Rodentia | Cricetidae | Cricetulus | kamensis | Cricetulus kamensis | NA | NA | 2957.22 | 10.16 | 4.112 | NA | NA | NA | NA | NA | 0.021048 |
| Soricomorpha | Soricidae | Cryptotis | hondurensis | Cryptotis hondurensis | NA | NA | 2987.92 | 82 | 21.05 | NA | NA | NA | NA | NA | 0.365391 |
| Rodentia | Muridae | Rattus | giluwensis | Rattus giluwensis | NA | NA | 2997.83 | 433.98 | 11.474 | NA | 2 | 1 | 1 | 1 | 0.265423 |
| Rodentia | Cricetidae | Ellobius | alaicus | Ellobius alaicus | NA | NA | 2999.15 | 19 | -5.525 | NA | NA | NA | NA | NA | 0.288395 |
| Rodentia | Geomyidae | Orthogeomys | matagalpae | Orthogeomys matagalpae | NA | NA | 3002.2 | 130.99 | 17.241 | NA | 3 | 2 | 1 | 1 | 0.015066 |
| Chiroptera | Vespertilionidae | Plecotus | teneriffae | Plecotus teneriffae | NA | NA | 3050.8 | 21 | 14.9 | NA | NA | 1 | 2 | NA | 1.331397 |
| Rodentia | Sciuridae | Spermophilus | perotensis | Spermophilus perotensis | 140 | 6.07 | 3089.88 | 127.83 | 12.766 | NA | NA | NA | NA | NA | 0.79673 |
| Rodentia | Muridae | Pogonomelomys | mayeri | Pogonomelomys mayeri | 111.97 | 2.15 | 3091.92 | 243.09 | 22.701 | NA | NA | 1 | 2 | NA | 0.018693 |
| Rodentia | Muridae | Mus | vulcani | Mus vulcani | NA | NA | 3092.68 | 280 | 23.05 | NA | NA | NA | NA | NA | 0.894331 |
| Primates | Cercopithecidae | Trachypithecus | laotum | Trachypithecus laotum | NA | NA | 3111.96 | 169.2 | 22.601 | 3 | NA | NA | NA | NA | 0.569982 |
| Rodentia | Cricetidae | Phyllotis | bonariensis | Phyllotis bonariensis | 42.5 | NA | 3123.42 | 60.76 | 12.923 | NA | NA | NA | NA | NA | 0.16023 |
| Rodentia | Muridae | Pseudohydromys | fuscus | Pseudohydromys fuscus | 18.74 | NA | 3155.41 | 315.03 | 14.36 | NA | 3 | 1 | 1 | 2 | 0.017222 |
| Chiroptera | Pteropodidae | Pteropus | dasymallus | Pteropus dasymallus | 491.86 | 1 | 3178.37 | 169.45 | 13.118 | 2 | 3 | 1 | 2 | 2 | 0.183805 |
| Chiroptera | Vespertilionidae | Kerivoula | agnella | Kerivoula agnella | NA | NA | 3241.93 | 267 | 25.4 | NA | NA | 1 | 2 | NA | 0.25096 |
| Rodentia | Cricetidae | Neacomys | pictus | Neacomys pictus | NA | NA | 3247.73 | 199.01 | 24.474 | 1 | 3 | 1 | 1 | 2 | 0.253837 |
| Soricomorpha | Soricidae | Crocidura | kivuana | Crocidura kivuana | NA | 1.66 | 3261.48 | 130.66 | 16.966 | NA | NA | NA | NA | NA | 0.89448 |
| Rodentia | Capromyidae | Mesocapromys | nanus | Mesocapromys nanus | NA | 1 | 3340.02 | 113.97 | 25.799 | NA | 3 | 1 | 2 | 2 | 1.711279 |
| Chiroptera | Hipposideridae | Hipposideros | demissus | Hipposideros demissus | NA | NA | 3352.82 | 225 | 25.7 | NA | NA | NA | NA | NA | 1.019271 |
| Rodentia | Cricetidae | Neusticomys | peruviensis | Neusticomys peruviensis | 40 | NA | 3393.98 | 358.15 | 23.8 | NA | 1 | 1 | NA | 3 | 0.016731 |
| Chiroptera | Pteropodidae | Pteropus | cognatus | Pteropus cognatus | NA | NA | 3397.56 | 225 | 25.7 | NA | NA | NA | NA | NA | 1.035229 |
| Rodentia | Cricetidae | Volemys | musseri | Volemys musseri | NA | NA | 3425.31 | 117.58 | 1.24 | NA | NA | NA | NA | NA | 0.286065 |
| Dasyuromorphia | Dasyuridae | Antechinus | godmani | Antechinus godmani | 75.74 | 4.58 | 3443.22 | 99.99 | 20.9 | 2 | NA | 1 | 1 | NA | 0.167576 |
| Rodentia | Ctenomyidae | Ctenomys | validus | Ctenomys validus | 232.6 | NA | 3491.05 | 26.99 | 15.402 | NA | 3 | 1 | 1 | 1 | 0.270692 |
| Rodentia | Cricetidae | Reithrodontomys | rodriguezi | Reithrodontomys rodriguezi | NA | NA | 3516.68 | 238.21 | 21.822 | 1 | NA | 2 | 2 | NA | 0.016984 |
| Diprotodontia | Macropodidae | Dendrolagus | pulcherrimus | Dendrolagus pulcherrimus | NA | NA | 3520.91 | 219.49 | 30.125 | NA | NA | NA | NA | NA | 1.238976 |
| Chiroptera | Molossidae | Chaerephon | gallagheri | Chaerephon gallagheri | NA | NA | 3529.03 | 137.79 | 23.74 | NA | NA | 1 | 2 | NA | 0.28867 |
| Rodentia | Cricetidae | Tylomys | panamensis | Tylomys panamensis | NA | NA | 3614.75 | 223.22 | 24.799 | NA | NA | NA | NA | NA | 0.313437 |
| Rodentia | Muridae | Mus | mattheyi | Mus mattheyi | NA | NA | 3619.53 | 80.66 | 26.533 | NA | NA | NA | NA | NA | 0.015632 |
| Rodentia | Muridae | Rattus | ranjiniae | Rattus ranjiniae | NA | NA | 3656.99 | 194.28 | 26.866 | NA | NA | NA | NA | NA | 1.252731 |
| Rodentia | Sciuridae | Sundasciurus | jentinki | Sundasciurus jentinki | NA | NA | 3662.22 | 277.83 | 22.916 | NA | NA | NA | NA | NA | 0.020635 |
| Rodentia | Cricetidae | Phyllotis | definitus | Phyllotis definitus | 89 | NA | 3705.21 | 108.5 | 23.599 | NA | NA | NA | NA | NA | 1.201728 |
| Rodentia | Cricetidae | Oryzomys | gorgasi | Oryzomys gorgasi | 60.5 | NA | 3724.47 | 232.87 | 25.483 | NA | NA | NA | NA | NA | 1.141482 |
| Chiroptera | Pteropodidae | Pteropus | keyensis | Pteropus keyensis | NA | NA | 3819.09 | 164 | 26.7 | NA | NA | NA | NA | NA | 0.224985 |
| Primates | Cebidae | Leontopithecus | caissara | Leontopithecus caissara | 604.01 | NA | 3822.49 | 148.79 | 16.439 | 3 | NA | 1 | 2 | NA | 1.229514 |
| Primates | Lepilemuridae | Lepilemur | ankaranensis | Lepilemur ankaranensis | NA | NA | 3823.56 | 101.31 | 25.15 | NA | NA | NA | NA | NA | 1.409588 |
| Artiodactyla | Bovidae | Cephalophus | adersi | Cephalophus adersi | 9249.95 | 1 | 3824.3 | 71.5 | 25.65 | NA | 4 | 1 | 1 | 1 | 1.508847 |
| Rodentia | Cricetidae | Thomasomys | ladewi | Thomasomys ladewi | 77 | NA | 3831.33 | 85.5 | 14.87 | NA | NA | NA | NA | NA | 0.014369 |
| Carnivora | Procyonidae | Bassaricyon | lasius | Bassaricyon lasius | 1200 | NA | 3842.65 | 238.21 | 21.822 | 1 | NA | 1 | 1 | NA | 0.321695 |
| Chiroptera | Molossidae | Molossus | barnesi | Molossus barnesi | NA | NA | 3843.41 | 303.4 | 24.659 | NA | NA | NA | NA | NA | 0.316908 |
| Rodentia | Muridae | Solomys | sapientis | Solomys sapientis | NA | NA | 3849.36 | 191.99 | 27.133 | NA | 1 | NA | NA | 1 | 1.409394 |
| Chiroptera | Pteropodidae | Nyctimene | malaitensis | Nyctimene malaitensis | 78.39 | NA | 3870.66 | 204.49 | 25.85 | NA | NA | 1 | 2 | NA | 0.258754 |
| Rodentia | Muridae | Praomys | minor | Praomys minor | NA | NA | 3908.1 | 151.83 | 26.016 | NA | NA | NA | NA | NA | 0.319247 |
| Rodentia | Muridae | Millardia | kathleenae | Millardia kathleenae | NA | NA | 3965.74 | 69.23 | 27.076 | NA | NA | NA | NA | NA | 0.018175 |
| Rodentia | Cricetidae | Nelsonia | goldmani | Nelsonia goldmani | NA | NA | 3979.58 | 89.5 | 20.329 | NA | NA | NA | NA | NA | 1.331324 |
| Rodentia | Muridae | Pithecheir | parvus | Pithecheir parvus | NA | 2 | 3988.07 | 177.25 | 25.175 | NA | NA | NA | NA | NA | 0.32226 |
| Carnivora | Eupleridae | Galidictis | grandidieri | Galidictis grandidieri | 1400 | 1 | 4044.38 | 43.21 | 24.182 | 2 | 2 | 1 | 1 | 3 | 1.487148 |
| Chiroptera | Pteropodidae | Megaerops | kusnotoi | Megaerops kusnotoi | NA | NA | 4065.54 | 239.38 | 23.067 | NA | NA | 1 | 2 | NA | 0.820417 |
| Primates | Cercopithecidae | Trachypithecus | barbei | Trachypithecus barbei | NA | NA | 4080.83 | 372.07 | 23.267 | NA | NA | NA | NA | NA | 0.167439 |
| Rodentia | Sciuridae | Ammospermophilus | nelsoni | Ammospermophilus nelsoni | 160.42 | 8.9 | 4125.09 | 40.84 | 13.354 | 3 | 5 | 2 | 1 | 2 | 0.879082 |
| Chiroptera | Vespertilionidae | Kerivoula | eriophora | Kerivoula eriophora | NA | NA | 4125.14 | 94.84 | 18.032 | NA | NA | 1 | 2 | NA | 0.25096 |
| Chiroptera | Vespertilionidae | Pipistrellus | maderensis | Pipistrellus maderensis | NA | NA | 4172.92 | 21 | 14.9 | NA | NA | 1 | 2 | NA | 1.388475 |
| Chiroptera | Phyllostomidae | Monophyllus | plethodon | Monophyllus plethodon | 15.33 | 0.99 | 4183.11 | NA | NA | NA | NA | 1 | 2 | NA | 0.017548 |
| Rodentia | Nesomyidae | Hypogeomys | antimena | Hypogeomys antimena | 1175.93 | 1.26 | 4191.4 | 78.01 | 24.76 | 1 | 5 | 2 | 1 | 1 | 1.640658 |
| Primates | Lemuridae | Eulemur | sanfordi | Eulemur sanfordi | 2394.03 | NA | 4197.82 | 101.31 | 25.15 | 2 | NA | NA | NA | NA | 1.29028 |
| Diprotodontia | Macropodidae | Dorcopsulus | macleayi | Dorcopsulus macleayi | 2780.54 | 1.24 | 4202.6 | 209.51 | 19.393 | 1 | 2 | 1 | 1 | 1 | 0.014865 |
| Rodentia | Muridae | Meriones | dahli | Meriones dahli | NA | NA | 4281.47 | 37.15 | 4.002 | NA | NA | NA | NA | NA | 1.351198 |
| Artiodactyla | Bovidae | Capra | walie | Capra walie | 100144 | 1 | 4294.5 | 81.2 | 20.638 | NA | NA | 1 | 1 | NA | 1.15736 |
| Rodentia | Muridae | Leimacomys | buettneri | Leimacomys buettneri | 54.99 | NA | 4323.64 | 113.59 | 26.439 | NA | NA | NA | NA | NA | 0.352773 |
| Rodentia | Cricetidae | Thomasomys | oreas | Thomasomys oreas | 77 | NA | 4357.67 | 116.67 | 20.351 | NA | NA | NA | NA | NA | 0.014369 |
| Dasyuromorphia | Dasyuridae | Sminthopsis | aitkeni | Sminthopsis aitkeni | 22.5 | NA | 4407.63 | 45.25 | 14.45 | 2 | NA | 1 | 1 | NA | 1.77199 |
| Soricomorpha | Soricidae | Sylvisorex | vulcanorum | Sylvisorex vulcanorum | 3.49 | NA | 4435.24 | 93.57 | 16.828 | NA | NA | NA | NA | NA | 0.202964 |
| Afrosoricida | Tenrecidae | Hemicentetes | nigriceps | Hemicentetes nigriceps | 102.99 | 2.84 | 4457.41 | 149.95 | 19.613 | 2 | 1 | NA | NA | 3 | 0.02357 |
| Chiroptera | Vespertilionidae | Hesperoptenus | tomesi | Hesperoptenus tomesi | NA | NA | 4472.42 | 224.26 | 24.035 | NA | NA | 1 | 2 | NA | 0.946355 |
| Rodentia | Erethizontidae | Sphiggurus | pruinosus | Sphiggurus pruinosus | NA | NA | 4489.82 | 106.84 | 21.35 | NA | NA | NA | NA | NA | 0.017385 |
| Rodentia | Muridae | Rattus | richardsoni | Rattus richardsoni | 64.29 | 2.15 | 4489.98 | 164.57 | 19.554 | NA | 3 | NA | NA | 2 | 0.884364 |
| Chiroptera | Phyllostomidae | Ardops | nichollsi | Ardops nichollsi | 19.23 | 0.99 | 4493.21 | NA | NA | NA | 1 | 1 | 2 | 1 | 0.012828 |
| Chiroptera | Vespertilionidae | Hypsugo | arabicus | Hypsugo arabicus | 3.17 | NA | 4494.53 | 5.85 | 21.269 | NA | NA | 1 | 2 | NA | 0.289538 |
| Rodentia | Cricetidae | Dicrostonyx | unalascensis | Dicrostonyx unalascensis | NA | 2.79 | 4501.73 | 93.02 | 2.456 | NA | NA | NA | NA | NA | 0.22404 |
| Rodentia | Muridae | Chiropodomys | karlkoopmani | Chiropodomys karlkoopmani | NA | NA | 4520.24 | 340 | 25.9 | NA | NA | NA | NA | NA | 1.366006 |
| Soricomorpha | Soricidae | Crocidura | andamanensis | Crocidura andamanensis | NA | NA | 4526.74 | 215 | 26.8 | NA | NA | NA | NA | NA | 1.699512 |
| Soricomorpha | Soricidae | Crocidura | jenkinsi | Crocidura jenkinsi | NA | NA | 4526.74 | 215 | 26.8 | NA | NA | NA | NA | NA | 1.699512 |
| Chiroptera | Rhinolophidae | Rhinolophus | cognatus | Rhinolophus cognatus | NA | NA | 4533.32 | 215 | 26.8 | NA | 1 | 1 | 2 | 3 | 1.227098 |
| Rodentia | Muridae | Kadarsanomys | sodyi | Kadarsanomys sodyi | NA | 4 | 4545.52 | 218.98 | 23.84 | NA | NA | NA | NA | NA | 1.184259 |
| Primates | Lemuridae | Prolemur | simus | Prolemur simus | 2030.41 | 1 | 4564.82 | 174.64 | 18.918 | 2 | 4 | 2 | 2 | 2 | 2.001906 |
| Rodentia | Ctenomyidae | Ctenomys | occultus | Ctenomys occultus | 150 | NA | 4611.74 | 66.07 | 14.486 | NA | 3 | 1 | 1 | 1 | 1.268871 |
| Rodentia | Muridae | Zyzomys | palatilis | Zyzomys palatilis | 123 | NA | 4667.41 | 49 | 24.716 | NA | NA | NA | NA | NA | 1.733534 |
| Rodentia | Muridae | Paraleptomys | wilhelmina | Paraleptomys wilhelmina | 33.68 | NA | 4717.36 | 198.83 | 18.531 | NA | NA | 1 | 1 | NA | 0.275546 |
| Rodentia | Cricetidae | Peromyscus | zarhynchus | Peromyscus zarhynchus | 40 | 1.94 | 4752.79 | 88.14 | 20.73 | 1 | NA | NA | NA | NA | 0.891228 |
| Rodentia | Muridae | Pseudomys | pilligaensis | Pseudomys pilligaensis | 10.72 | NA | 4765.35 | 59.4 | 15.417 | NA | NA | NA | NA | NA | 0.346391 |
| Rodentia | Muridae | Praomys | morio | Praomys morio | 41.73 | 3.28 | 4792.43 | 285.75 | 24.049 | NA | 4 | NA | NA | 2 | 1.49647 |
| Soricomorpha | Soricidae | Crocidura | zimmermanni | Crocidura zimmermanni | NA | NA | 4817.08 | 51.8 | 16.38 | NA | NA | NA | NA | NA | 0.89448 |
| Rodentia | Muridae | Rattus | jobiensis | Rattus jobiensis | 380 | NA | 4824.46 | 225.99 | 25.2 | NA | NA | NA | NA | NA | 0.165889 |
| Rodentia | Muridae | Mus | famulus | Mus famulus | NA | NA | 4826.36 | 118.17 | 25.502 | NA | NA | NA | NA | NA | 1.341497 |
| Soricomorpha | Soricidae | Crocidura | usambarae | Crocidura usambarae | 11.83 | 1.99 | 4845.06 | 107.72 | 23.612 | NA | NA | NA | NA | NA | 1.34172 |
| Rodentia | Sciuridae | Paraxerus | vincenti | Paraxerus vincenti | NA | NA | 4870.34 | 110.99 | 22.099 | NA | NA | NA | NA | NA | 1.329119 |
| Rodentia | Cricetidae | Neusticomys | mussoi | Neusticomys mussoi | 40 | NA | 4883.45 | 106.84 | 21.35 | NA | NA | 1 | NA | NA | 1.254803 |
| Rodentia | Echimyidae | Proechimys | trinitatus | Proechimys trinitatus | NA | NA | 4884.87 | 145.49 | 24.549 | NA | NA | NA | NA | NA | 0.330599 |
| Rodentia | Geomyidae | Orthogeomys | underwoodi | Orthogeomys underwoodi | 250 | NA | 4894.08 | 214.18 | 21.968 | NA | 3 | 2 | 1 | 1 | 0.015066 |
| Rodentia | Cricetidae | Dicrostonyx | nelsoni | Dicrostonyx nelsoni | NA | NA | 4909.61 | 34 | -3.7 | NA | NA | NA | NA | NA | 0.014002 |
| Soricomorpha | Soricidae | Sorex | jacksoni | Sorex jacksoni | 6.01 | NA | 4909.61 | 34 | -3.7 | NA | NA | NA | NA | NA | 0.018367 |
| Didelphimorphia | Didelphidae | Marmosops | cracens | Marmosops cracens | 26.26 | NA | 4912.21 | 72.84 | 27.35 | 1 | NA | NA | NA | NA | 0.361966 |
| Rodentia | Muridae | Meriones | sacramenti | Meriones sacramenti | NA | 5.99 | 4930.65 | 24.09 | 19.213 | NA | NA | NA | NA | NA | 0.900799 |
| Soricomorpha | Soricidae | Crocidura | zaphiri | Crocidura zaphiri | NA | NA | 4952.1 | 106.39 | 17.868 | NA | NA | NA | NA | NA | 0.286234 |
| Chiroptera | Rhinolophidae | Rhinolophus | madurensis | Rhinolophus madurensis | NA | NA | 4960.23 | 110 | 26.9 | NA | NA | NA | NA | NA | 0.742194 |
| Soricomorpha | Soricidae | Myosorex | sclateri | Myosorex sclateri | NA | NA | 5021.64 | 88.2 | 19.938 | NA | NA | NA | NA | NA | 0.202959 |
| Primates | Tarsiidae | Tarsius | dentatus | Tarsius dentatus | 110.42 | 1.01 | 5028.57 | 205.99 | 18.6 | NA | NA | NA | NA | NA | 1.079539 |
| Rodentia | Sciuridae | Callosciurus | melanogaster | Callosciurus melanogaster | 296 | NA | 5140.87 | 340 | 25.9 | 3 | 4 | 2 | 2 | 2 | 1.015335 |
| Rodentia | Sciuridae | Petinomys | lugens | Petinomys lugens | NA | NA | 5140.87 | 340 | 25.9 | NA | NA | NA | NA | NA | 1.139112 |
| Rodentia | Sciuridae | Sundasciurus | fraterculus | Sundasciurus fraterculus | NA | NA | 5140.87 | 340 | 25.9 | NA | NA | NA | NA | NA | 1.54761 |
| Chiroptera | Pteropodidae | Pteropus | pohlei | Pteropus pohlei | 352.75 | NA | 5156.49 | 225.99 | 25.2 | NA | NA | 1 | 2 | NA | 1.100918 |
| Rodentia | Ctenomyidae | Ctenomys | tucumanus | Ctenomys tucumanus | 217 | NA | 5165.21 | 68.36 | 14.632 | NA | 3 | 1 | 1 | 1 | 0.270692 |
| Rodentia | Muridae | Mus | orangiae | Mus orangiae | NA | NA | 5172.83 | 52.25 | 16.148 | NA | NA | NA | NA | NA | 0.015632 |
| Rodentia | Cricetidae | Peromyscus | melanurus | Peromyscus melanurus | 40 | 2.75 | 5191.19 | 73.19 | 18.747 | 1 | NA | NA | NA | NA | 1.336841 |
| Rodentia | Sciuridae | Marmota | olympus | Marmota olympus | 6299.99 | 4 | 5281.29 | 165.7 | 8.533 | NA | NA | NA | NA | NA | 0.008438 |
| Chiroptera | Rhinolophidae | Rhinolophus | hilli | Rhinolophus hilli | 13.6 | NA | 5351.09 | 106.5 | 17.816 | NA | NA | NA | NA | NA | 0.893767 |
| Rodentia | Ctenomyidae | Ctenomys | frater | Ctenomys frater | 172.9 | NA | 5398.92 | 50.5 | 4.718 | NA | 3 | 2 | 1 | 1 | 0.015784 |
| Carnivora | Viverridae | Paradoxurus | zeylonensis | Paradoxurus zeylonensis | 2821.3 | 2.5 | 5405.27 | 170.31 | 24.582 | 1 | 6 | 1 | 1 | 2 | 0.889216 |
| Rodentia | Cricetidae | Peromyscus | grandis | Peromyscus grandis | 71 | 2.43 | 5437.62 | 132.32 | 22.878 | 1 | NA | NA | NA | NA | 0.178246 |
| Soricomorpha | Soricidae | Crocidura | tansaniana | Crocidura tansaniana | NA | NA | 5441.49 | 107.72 | 23.612 | NA | NA | NA | NA | NA | 1.34172 |
| Chiroptera | Pteropodidae | Dobsonia | pannietensis | Dobsonia pannietensis | 239.19 | 0.98 | 5451.04 | 267 | 25.4 | NA | 1 | 1 | 2 | 1 | 0.112625 |
| Chiroptera | Hipposideridae | Hipposideros | pelingensis | Hipposideros pelingensis | NA | NA | 5493.35 | 240.34 | 21.934 | NA | NA | NA | NA | NA | 0.210538 |
| Rodentia | Muridae | Uromys | rex | Uromys rex | 419.99 | NA | 5496.09 | 200 | 24.75 | NA | NA | NA | NA | NA | 1.309 |
| Diprotodontia | Macropodidae | Petrogale | persephone | Petrogale persephone | 5585.63 | 1 | 5496.97 | 100.1 | 19.551 | 2 | 1 | 1 | 1 | 1 | 1.272033 |
| Dasyuromorphia | Dasyuridae | Antechinus | leo | Antechinus leo | 70.09 | 8.19 | 5555.28 | 103.26 | 27.925 | 2 | NA | 1 | 1 | NA | 0.015079 |
| Primates | Atelidae | Brachyteles | hypoxanthus | Brachyteles hypoxanthus | NA | NA | 5564.78 | 122.82 | 19.367 | 3 | NA | NA | NA | NA | 1.57125 |
| Soricomorpha | Soricidae | Sorex | hosonoi | Sorex hosonoi | NA | NA | 5576.94 | 136.94 | 6.237 | NA | NA | NA | NA | NA | 0.018767 |
| Rodentia | Calomyscidae | Calomyscus | urartensis | Calomyscus urartensis | NA | NA | 5580.24 | 31.81 | 9.145 | NA | NA | NA | NA | NA | 0.019168 |
| Rodentia | Sciuridae | Sundasciurus | juvencus | Sundasciurus juvencus | NA | NA | 5587.9 | 219.66 | 25.366 | NA | NA | NA | NA | NA | 0.020635 |
| Rodentia | Heteromyidae | Liomys | spectabilis | Liomys spectabilis | 65 | NA | 5600.81 | 92.25 | 20.502 | NA | NA | 1 | 1 | NA | 0.987397 |
| Soricomorpha | Soricidae | Solisorex | pearsoni | Solisorex pearsoni | NA | NA | 5615.92 | 170.49 | 24.433 | NA | NA | NA | NA | NA | 1.527265 |
| Rodentia | Cricetidae | Oligoryzomys | griseolus | Oligoryzomys griseolus | 25.2 | NA | 5640.42 | 114.18 | 13.516 | NA | NA | NA | NA | NA | 0.013332 |
| Dasyuromorphia | Dasyuridae | Planigale | novaeguineae | Planigale novaeguineae | 14.9 | NA | 5648.74 | 128.71 | 23.934 | 2 | NA | 1 | 1 | NA | 0.016473 |
| Rodentia | Erethizontidae | Sphiggurus | vestitus | Sphiggurus vestitus | 736 | NA | 5697.25 | 183.83 | 18.057 | 1 | NA | 1 | 2 | NA | 0.278152 |
| Chiroptera | Pteropodidae | Pteropus | aruensis | Pteropus aruensis | NA | NA | 5715.75 | 173 | 26.174 | NA | NA | NA | NA | NA | 1.335846 |
| Primates | Lemuridae | Eulemur | coronatus | Eulemur coronatus | 1699.85 | 1.53 | 5720.16 | 105.09 | 23.779 | 2 | 4 | 2 | 2 | 2 | 0.8702 |
| Lagomorpha | Leporidae | Sylvilagus | insonus | Sylvilagus insonus | 2999.98 | NA | 5755.87 | 110.83 | 19.234 | NA | NA | 1 | 1 | NA | 1.15894 |
| Rodentia | Abrocomidae | Abrocoma | boliviensis | Abrocoma boliviensis | 158 | NA | 5773.97 | 82.63 | 17.555 | NA | NA | NA | NA | NA | 2.527948 |
| Rodentia | Cricetidae | Oecomys | cleberi | Oecomys cleberi | 73.4 | NA | 5781.47 | 125 | 21.166 | NA | NA | NA | NA | NA | 0.204676 |
| Soricomorpha | Soricidae | Suncus | zeylanicus | Suncus zeylanicus | NA | NA | 5784.3 | 174.28 | 24.685 | NA | NA | NA | NA | NA | 1.279622 |
| Primates | Pitheciidae | Callicebus | oenanthe | Callicebus oenanthe | 992.4 | 1.02 | 5807.21 | 139.67 | 22.05 | 3 | NA | 1 | 2 | NA | 1.184427 |
| Rodentia | Cricetidae | Phyllotis | osgoodi | Phyllotis osgoodi | 45.1 | NA | 5828.69 | 31.64 | -3.256 | NA | NA | NA | NA | NA | 0.256369 |
| Chiroptera | Pteropodidae | Pteropus | melanotus | Pteropus melanotus | NA | 1 | 5919.16 | 277.5 | 26.149 | NA | NA | 1 | 2 | NA | 0.919026 |
| Soricomorpha | Soricidae | Surdisorex | norae | Surdisorex norae | 23.57 | 1.41 | 5920.86 | 66.28 | 15.999 | NA | NA | NA | NA | NA | 0.886938 |
| Rodentia | Nesomyidae | Steatomys | jacksoni | Steatomys jacksoni | NA | NA | 5980.85 | 113.75 | 26.499 | NA | NA | NA | NA | NA | 0.345698 |
| Rodentia | Cricetidae | Scotinomys | xerampelinus | Scotinomys xerampelinus | 15.07 | 3.08 | 5990.23 | 253.24 | 25.532 | NA | NA | NA | NA | NA | 0.017103 |
| Rodentia | Geomyidae | Orthogeomys | cherriei | Orthogeomys cherriei | NA | NA | 6002.72 | 304.68 | 24.857 | NA | 3 | 2 | 1 | 1 | 0.015066 |
| Primates | Hylobatidae | Hylobates | klossii | Hylobates klossii | 5822.29 | 1.01 | 6044.85 | 340 | 25.9 | 3 | 3 | 1 | 2 | 2 | 1.084189 |
| Rodentia | Sciuridae | Lariscus | obscurus | Lariscus obscurus | 241.19 | NA | 6044.85 | 340 | 25.9 | 3 | 3 | 2 | 2 | 2 | 0.179139 |
| Rodentia | Muridae | Leopoldamys | siporanus | Leopoldamys siporanus | NA | NA | 6044.85 | 340 | 25.9 | NA | NA | NA | NA | NA | 1.467115 |
| Rodentia | Muridae | Maxomys | pagensis | Maxomys pagensis | NA | NA | 6044.85 | 340 | 25.9 | NA | NA | NA | NA | NA | 1.609335 |
| Primates | Cercopithecidae | Presbytis | potenziani | Presbytis potenziani | 6449.53 | 1.02 | 6044.85 | 340 | 25.9 | 3 | NA | NA | NA | NA | 1.066251 |
| Rodentia | Muridae | Rattus | lugens | Rattus lugens | NA | NA | 6044.85 | 340 | 25.9 | NA | NA | NA | NA | NA | 1.047214 |
| Primates | Cercopithecidae | Simias | concolor | Simias concolor | 7393.9 | 1.02 | 6044.85 | 340 | 25.9 | 3 | 3 | 2 | 2 | 1 | 1.335939 |
| Rodentia | Cricetidae | Abrothrix | markhami | Abrothrix markhami | NA | NA | 6084.17 | 281.3 | 4.678 | NA | NA | NA | NA | NA | 0.013492 |
| Macroscelidea | Macroscelididae | Rhynchocyon | chrysopygus | Rhynchocyon chrysopygus | 535.2 | 1.22 | 6122.78 | 62.32 | 26.722 | 3 | 1 | 1 | 1 | 3 | 1.842022 |
| Rodentia | Geomyidae | Thomomys | bulbivorus | Thomomys bulbivorus | 359.9 | 4.99 | 6147.08 | 114.34 | 10.1 | NA | 3 | 2 | 1 | 1 | 0.020196 |
| Rodentia | Muridae | Praomys | verschureni | Praomys verschureni | NA | NA | 6171.24 | 121.5 | 19.374 | NA | NA | NA | NA | NA | 0.309682 |
| Rodentia | Octodontidae | Tympanoctomys | barrerae | Tympanoctomys barrerae | 86.02 | NA | 6195.04 | 31.67 | 14.586 | 1 | 1 | 2 | 1 | 1 | 0.245784 |
| Chiroptera | Pteropodidae | Dyacopterus | brooksi | Dyacopterus brooksi | NA | NA | 6196.65 | 316.14 | 22.657 | NA | NA | NA | NA | NA | 0.778143 |
| Rodentia | Sciuridae | Sundasciurus | steerii | Sundasciurus steerii | NA | NA | 6201.41 | 227.33 | 24.599 | NA | NA | NA | NA | NA | 0.020635 |
| Primates | Lemuridae | Varecia | rubra | Varecia rubra | 3872.6 | NA | 6367.02 | 176.31 | 22.332 | NA | NA | NA | NA | NA | 1.524873 |
| Rodentia | Cricetidae | Oryzomys | lamia | Oryzomys lamia | 60.5 | NA | 6408.1 | 137.44 | 20.786 | NA | NA | NA | NA | NA | 1.141482 |
| Rodentia | Muridae | Lemniscomys | mittendorfi | Lemniscomys mittendorfi | 41.85 | NA | 6452.05 | 152.75 | 21.999 | 3 | NA | 1 | 1 | NA | 0.974939 |
| Diprotodontia | Phalangeridae | Strigocuscus | pelengensis | Strigocuscus pelengensis | NA | NA | 6454.39 | 250.99 | 23.866 | 1 | 1 | 1 | 2 | 1 | 0.019667 |
| Soricomorpha | Soricidae | Suncus | fellowesgordoni | Suncus fellowesgordoni | NA | NA | 6493.32 | 174.28 | 24.685 | NA | NA | NA | NA | NA | 1.279622 |
| Rodentia | Cricetidae | Isthmomys | flavidus | Isthmomys flavidus | NA | NA | 6512.16 | 231.2 | 26.408 | NA | 3 | 1 | 1 | 1 | 0.178505 |
| Primates | Cheirogaleidae | Phaner | furcifer | Phaner furcifer | 409.87 | 1.01 | 6525.13 | 176.31 | 22.332 | 2 | 3 | 1 | 2 | 2 | 0.02286 |
| Rodentia | Muridae | Leopoldamys | milleti | Leopoldamys milleti | NA | NA | 6549 | 139 | 21.025 | NA | NA | NA | NA | NA | 0.017821 |
| Rodentia | Sciuridae | Marmota | vancouverensis | Marmota vancouverensis | 5232.43 | 3.59 | 6678.32 | 175.79 | 6.488 | 3 | 4 | 2 | 1 | 1 | 0.801611 |
| Rodentia | Muridae | Mus | crociduroides | Mus crociduroides | NA | NA | 6687.02 | 341.57 | 21.328 | NA | NA | NA | NA | NA | 0.286186 |
| Rodentia | Muridae | Uromys | hadrourus | Uromys hadrourus | 149.11 | 3 | 6687.39 | 123.43 | 21.08 | NA | 1 | NA | NA | 1 | 0.872667 |
| Soricomorpha | Soricidae | Crocidura | paradoxura | Crocidura paradoxura | NA | NA | 6689.37 | 298.66 | 22.666 | NA | NA | NA | NA | NA | 0.01789 |
| Rodentia | Heteromyidae | Dipodomys | ingens | Dipodomys ingens | 113.61 | 4.77 | 6724.92 | 36.13 | 13.281 | 1 | 3 | 2 | 1 | 2 | 1.004754 |
| Rodentia | Heteromyidae | Chaetodipus | dalquesti | Chaetodipus dalquesti | NA | NA | 6751.03 | 19.17 | 20.062 | NA | NA | NA | NA | NA | 0.854907 |
| Rodentia | Cricetidae | Megadontomys | cryophilus | Megadontomys cryophilus | NA | NA | 6755.29 | 104.4 | 14.497 | NA | NA | NA | NA | NA | 1.264304 |
| Rodentia | Cricetidae | Sigmodontomys | aphrastus | Sigmodontomys aphrastus | NA | NA | 6781.3 | 244.2 | 25.843 | NA | NA | NA | NA | NA | 0.264291 |
| Primates | Atelidae | Brachyteles | arachnoides | Brachyteles arachnoides | 10537.31 | 1.02 | 6796.12 | 132.7 | 21.176 | 3 | 5 | 1 | 2 | 2 | 1.24046 |
| Rodentia | Muridae | Apodemus | hyrcanicus | Apodemus hyrcanicus | NA | NA | 6808.71 | 38.8 | 12.169 | NA | NA | NA | NA | NA | 0.142339 |
| Chiroptera | Vespertilionidae | Hesperoptenus | doriae | Hesperoptenus doriae | NA | NA | 6872.68 | 240.39 | 24.445 | NA | NA | 1 | 2 | NA | 0.365693 |
| Rodentia | Muridae | Praomys | mutoni | Praomys mutoni | NA | NA | 6883.22 | 151.71 | 24.928 | NA | NA | NA | NA | NA | 0.319247 |
| Primates | Cebidae | Saimiri | oerstedii | Saimiri oerstedii | 714.18 | 1.01 | 6892.87 | 228 | 26.574 | 3 | 2 | 1 | 2 | 2 | 0.662078 |
| Rodentia | Muridae | Dipodillus | lowei | Dipodillus lowei | NA | NA | 6901.71 | 33 | 20.6 | NA | NA | NA | NA | NA | 0.35571 |
| Rodentia | Cricetidae | Thomasomys | bombycinus | Thomasomys bombycinus | 114.5 | NA | 6928.01 | 192 | 18.066 | NA | NA | NA | NA | NA | 0.229903 |
| Chiroptera | Molossidae | Chaerephon | solomonis | Chaerephon solomonis | NA | NA | 6980.78 | 189.19 | 26.959 | NA | NA | NA | NA | NA | 0.01505 |
| Primates | Lemuridae | Eulemur | mongoz | Eulemur mongoz | 1771.13 | 1.11 | 7006.3 | 125.88 | 26.199 | 2 | 4 | 1 | 2 | 2 | 0.859047 |
| Primates | Cebidae | Saimiri | vanzolinii | Saimiri vanzolinii | 783.77 | 1.02 | 7011.35 | 231.87 | 25.849 | 3 | NA | NA | NA | NA | 0.702392 |
| Artiodactyla | Cervidae | Muntiacus | rooseveltorum | Muntiacus rooseveltorum | 10762.64 | 1 | 7016.69 | 128.18 | 19.52 | NA | NA | NA | NA | NA | 0.224745 |
| Chiroptera | Pteropodidae | Nyctimene | masalai | Nyctimene masalai | 53.2 | NA | 7066.71 | 189.5 | 23.8 | NA | NA | 1 | 2 | NA | 0.258754 |
| Soricomorpha | Talpidae | Euroscaptor | mizura | Euroscaptor mizura | 25.05 | 3 | 7152.32 | 129.53 | 6.749 | 2 | 1 | 1 | 1 | 3 | 0.021789 |
| Soricomorpha | Soricidae | Feroculus | feroculus | Feroculus feroculus | 35.99 | NA | 7179.99 | 174.28 | 24.685 | NA | NA | 2 | 1 | NA | 1.527265 |
| Chiroptera | Vespertilionidae | Arielulus | societatis | Arielulus societatis | NA | NA | 7183.23 | 203.61 | 24.225 | NA | NA | 1 | 2 | NA | 0.944088 |
| Rodentia | Cricetidae | Oxymycterus | hucucha | Oxymycterus hucucha | 67.99 | NA | 7184.44 | 75.25 | 16.553 | NA | 1 | NA | NA | 3 | 1.096719 |
| Primates | Cheirogaleidae | Phaner | parienti | Phaner parienti | NA | NA | 7212.36 | 140 | 20.751 | NA | NA | NA | NA | NA | 1.142985 |
| Rodentia | Muridae | Rattus | korinchi | Rattus korinchi | NA | NA | 7261.35 | 292.49 | 22.02 | NA | NA | NA | NA | NA | 0.267249 |
| Rodentia | Muridae | Otomys | occidentalis | Otomys occidentalis | NA | NA | 7267.08 | 152.5 | 21.866 | NA | NA | NA | NA | NA | 0.964693 |
| Diprotodontia | Potoroidae | Potorous | longipes | Potorous longipes | 1835.06 | 1 | 7279.9 | 64.39 | 9.893 | 1 | 3 | 1 | 1 | 1 | 1.600952 |
| Primates | Cercopithecidae | Macaca | nigrescens | Macaca nigrescens | NA | NA | 7280.48 | 231.2 | 22.68 | 3 | NA | NA | NA | NA | 0.645407 |
| Chiroptera | Vespertilionidae | Myotis | hajastanicus | Myotis hajastanicus | NA | NA | 7282.75 | 36.37 | 4.004 | NA | NA | NA | NA | NA | 1.365136 |
| Rodentia | Cricetidae | Bibimys | chacoensis | Bibimys chacoensis | 28 | NA | 7309.86 | 106.51 | 21.014 | NA | NA | NA | NA | NA | 0.011944 |
| Chiroptera | Pteropodidae | Pteralopex | anceps | Pteralopex anceps | 574.75 | 1 | 7318.84 | 182.39 | 26.139 | NA | NA | 1 | 2 | NA | 1.287363 |
| Chiroptera | Vespertilionidae | Rhogeessa | mira | Rhogeessa mira | NA | NA | 7524.33 | 106.38 | 22.89 | NA | NA | 1 | 2 | NA | 0.779697 |
| Dasyuromorphia | Myrmecobiidae | Myrmecobius | fasciatus | Myrmecobius fasciatus | 511.44 | 3.68 | 7560.06 | 58.51 | 14.404 | 2 | 1 | 2 | 1 | 3 | 1.806954 |
| Primates | Indriidae | Avahi | occidentalis | Avahi occidentalis | 828.98 | 1.02 | 7563.78 | 118.44 | 24.949 | 2 | 1 | 1 | 2 | 1 | 1.568271 |
| Soricomorpha | Soricidae | Crocidura | miya | Crocidura miya | NA | NA | 7582.81 | 174.28 | 24.685 | NA | NA | NA | NA | NA | 1.34172 |
| Primates | Indriidae | Propithecus | coquereli | Propithecus coquereli | 4189.27 | 1 | 7668.04 | 134.79 | 25.142 | 3 | NA | NA | NA | NA | 1.393428 |
| Rodentia | Cricetidae | Dicrostonyx | vinogradovi | Dicrostonyx vinogradovi | NA | NA | 7718.13 | 8.85 | -12.028 | NA | NA | NA | NA | NA | 0.22404 |
| Rodentia | Cricetidae | Lemmus | portenkoi | Lemmus portenkoi | NA | NA | 7718.13 | 8.85 | -12.028 | NA | NA | NA | NA | NA | 0.256781 |
| Diprotodontia | Potoroidae | Potorous | gilbertii | Potorous gilbertii | 1569.46 | 1 | 7753.72 | 64.14 | 14.266 | 1 | 2 | NA | NA | 1 | 1.851915 |
| Rodentia | Ctenomyidae | Ctenomys | azarae | Ctenomys azarae | 400 | NA | 7836.88 | 36.9 | 14.936 | NA | 3 | 1 | 1 | 1 | 0.845914 |
| Rodentia | Cricetidae | Peromyscus | mekisturus | Peromyscus mekisturus | 59.99 | NA | 7871.4 | 130.21 | 16.579 | 1 | NA | NA | NA | NA | 1.693332 |
| Rodentia | Nesomyidae | Dendromus | vernayi | Dendromus vernayi | NA | NA | 7879.79 | 101.65 | 21.233 | NA | NA | NA | NA | NA | 0.360754 |
| Chiroptera | Pteropodidae | Dobsonia | beauforti | Dobsonia beauforti | 165.35 | 0.99 | 7965.78 | 222.99 | 25.866 | NA | NA | 1 | 2 | NA | 0.011041 |
| Soricomorpha | Soricidae | Crocidura | fischeri | Crocidura fischeri | NA | NA | 8016.47 | 59.72 | 21.345 | NA | NA | NA | NA | NA | 0.286234 |
| Rodentia | Sciuridae | Petaurillus | kinlochii | Petaurillus kinlochii | 30.93 | NA | 8094.4 | 183 | 25.5 | NA | NA | NA | NA | NA | 0.213518 |
| Rodentia | Heteromyidae | Perognathus | alticolus | Perognathus alticolus | 23.99 | NA | 8126.88 | 32.66 | 10.387 | NA | NA | NA | NA | NA | 1.533156 |
| Rodentia | Cricetidae | Oxymycterus | hiska | Oxymycterus hiska | 67.99 | NA | 8134.63 | 192.53 | 1.243 | NA | 1 | NA | NA | 3 | 0.014623 |
| Rodentia | Muridae | Acomys | minous | Acomys minous | 62.5 | NA | 8190.7 | 51.8 | 16.38 | NA | NA | NA | NA | NA | 0.221612 |
| Rodentia | Muridae | Gerbillus | brockmani | Gerbillus brockmani | NA | NA | 8207.28 | 13.79 | 22.64 | NA | NA | NA | NA | NA | 0.35571 |
| Rodentia | Ctenomyidae | Ctenomys | bonettoi | Ctenomys bonettoi | 202.3 | NA | 8251.77 | 93.97 | 21.282 | NA | 3 | 1 | 1 | 1 | 1.268871 |
| Rodentia | Cricetidae | Peromyscus | melanocarpus | Peromyscus melanocarpus | 58.9 | 2.23 | 8317.42 | 107.51 | 19.803 | 1 | NA | NA | NA | NA | 1.336841 |
| Diprotodontia | Petauridae | Gymnobelideus | leadbeateri | Gymnobelideus leadbeateri | 137.32 | 1.54 | 8327.67 | 57.49 | 11.674 | 1 | 2 | 1 | 2 | 2 | 1.74471 |
| Rodentia | Cricetidae | Reithrodontomys | hirsutus | Reithrodontomys hirsutus | 20 | 3.88 | 8359.01 | 77.67 | 18.885 | 1 | NA | 2 | 2 | NA | 0.862673 |
| Primates | Lemuridae | Eulemur | macaco | Eulemur macaco | 2470.43 | 1.04 | 8406.83 | 134.19 | 22.255 | 2 | 4 | 1 | 2 | 2 | 0.862661 |
| Chiroptera | Hipposideridae | Hipposideros | inexpectatus | Hipposideros inexpectatus | NA | NA | 8430.51 | 231.85 | 20.557 | NA | 1 | 1 | 2 | 3 | 0.39109 |
| Rodentia | Heteromyidae | Dipodomys | stephensi | Dipodomys stephensi | 68.49 | 2.68 | 8439.21 | 25.61 | 11.41 | NA | 1 | NA | NA | 1 | 0.95807 |
| Rodentia | Sciuridae | Paraxerus | vexillarius | Paraxerus vexillarius | 675 | NA | 8466.8 | 117.23 | 23.924 | NA | NA | NA | NA | NA | 0.177216 |
| Rodentia | Cricetidae | Thomasomys | niveipes | Thomasomys niveipes | 54.09 | NA | 8507.47 | 76.89 | 13.613 | NA | NA | NA | NA | NA | 0.014369 |
| Rodentia | Muridae | Srilankamys | ohiensis | Srilankamys ohiensis | NA | NA | 8580.23 | 148.28 | 25.229 | NA | NA | NA | NA | NA | 1.184259 |
| Rodentia | Cricetidae | Melanomys | robustulus | Melanomys robustulus | 53.5 | NA | 8638.62 | 62.56 | 18.855 | NA | NA | NA | NA | NA | 0.015847 |
| Diprotodontia | Petauridae | Petaurus | gracilis | Petaurus gracilis | 360.63 | 1.75 | 8721.42 | 152.07 | 21.354 | 1 | 4 | 1 | 2 | 2 | 1.277345 |
| Carnivora | Canidae | Lycalopex | fulvipes | Lycalopex fulvipes | NA | NA | 8795.59 | 153.53 | 10.498 | NA | NA | NA | NA | NA | 0.899634 |
| Rodentia | Sciuridae | Prosciurillus | weberi | Prosciurillus weberi | NA | NA | 8804.22 | 220.12 | 16.499 | NA | NA | NA | NA | NA | 0.307928 |
| Chiroptera | Phyllostomidae | Ariteus | flavescens | Ariteus flavescens | NA | NA | 8821.83 | 104.75 | 24 | NA | NA | 1 | 2 | NA | 0.012828 |
| Soricomorpha | Soricidae | Ruwenzorisorex | suncoides | Ruwenzorisorex suncoides | 18.2 | NA | 8823.5 | 117.31 | 18.692 | NA | NA | NA | NA | NA | 1.018177 |
| Rodentia | Ctenomyidae | Ctenomys | lewisi | Ctenomys lewisi | 117.2 | NA | 8824.32 | 43.13 | 10.348 | NA | 3 | 3 | 1 | 1 | 0.016918 |
| Chiroptera | Hipposideridae | Hipposideros | curtus | Hipposideros curtus | NA | NA | 8853.55 | 257.65 | 25.733 | NA | NA | 1 | 2 | NA | 1.224118 |
| Lagomorpha | Leporidae | Lepus | castroviejoi | Lepus castroviejoi | 2822.41 | 1.95 | 8875.2 | 60.61 | 8.408 | NA | NA | NA | NA | NA | 0.668674 |
| Rodentia | Sciuridae | Tamias | alpinus | Tamias alpinus | 36.54 | 4.5 | 8880.39 | 51.65 | 3.83 | NA | 1 | 2 | 2 | 1 | 0.014486 |
| Rodentia | Cricetidae | Microtus | canicaudus | Microtus canicaudus | 29.93 | 4.62 | 8888.55 | 106.39 | 9.754 | NA | NA | 2 | 1 | NA | 0.014203 |
| Chiroptera | Phyllostomidae | Stenoderma | rufum | Stenoderma rufum | 21.1 | 0.99 | 8961.11 | 104.75 | 24 | NA | 1 | 1 | 2 | 1 | 0.727731 |
| Primates | Cebidae | Saguinus | martinsi | Saguinus martinsi | NA | NA | 9116.87 | 133.29 | 26.049 | NA | NA | NA | NA | NA | 0.012944 |
| Rodentia | Muridae | Acomys | nesiotes | Acomys nesiotes | NA | NA | 9293.99 | 40.19 | 18.355 | NA | NA | NA | NA | NA | 0.221612 |
| Primates | Lemuridae | Hapalemur | aureus | Hapalemur aureus | 1562.41 | 1.01 | 9313.01 | 165.45 | 18.524 | 2 | 2 | 2 | 2 | 1 | 1.580452 |
| Chiroptera | Pteropodidae | Pteralopex | atrata | Pteralopex atrata | 490.8 | NA | 9345.46 | 195.19 | 26.182 | NA | NA | 1 | 2 | NA | 1.287363 |
| Afrosoricida | Tenrecidae | Microgale | gracilis | Microgale gracilis | 23.3 | NA | 9350.81 | 167.57 | 18.802 | NA | NA | 1 | 1 | NA | 0.021013 |
| Soricomorpha | Talpidae | Euroscaptor | parvidens | Euroscaptor parvidens | NA | NA | 9353.46 | 132.14 | 21.57 | NA | 1 | 2 | 1 | 3 | 0.348621 |
| Carnivora | Mustelidae | Martes | gwatkinsii | Martes gwatkinsii | 2038.49 | NA | 9366.98 | 155.37 | 25.998 | 2 | 3 | 1 | 1 | 2 | 0.865846 |
| Chiroptera | Pteropodidae | Rousettus | bidens | Rousettus bidens | NA | 0.98 | 9406.49 | 230.16 | 22.75 | NA | NA | 1 | 2 | NA | 0.943657 |
| Chiroptera | Vespertilionidae | Lasiurus | castaneus | Lasiurus castaneus | 12.51 | NA | 9516.32 | 200.91 | 24.837 | NA | 1 | 1 | 2 | 3 | 0.264955 |
| Soricomorpha | Soricidae | Suncus | dayi | Suncus dayi | NA | NA | 9522.82 | 166.68 | 27.01 | NA | NA | NA | NA | NA | 1.279622 |
| Soricomorpha | Soricidae | Crocidura | caliginea | Crocidura caliginea | NA | NA | 9530.16 | 159.74 | 24.517 | NA | NA | NA | NA | NA | 0.01789 |
| Primates | Lepilemuridae | Lepilemur | dorsalis | Lepilemur dorsalis | 503.85 | 1.01 | 9532.94 | 133.28 | 20.161 | 1 | 3 | 1 | 2 | 1 | 0.311582 |
| Rodentia | Sciuridae | Tamias | ochrogenys | Tamias ochrogenys | 91.69 | NA | 9548.52 | 68.08 | 10.497 | NA | 3 | NA | NA | 2 | 0.015605 |
| Rodentia | Cricetidae | Neotoma | angustapalata | Neotoma angustapalata | 198 | NA | 9613.18 | 54.61 | 22.157 | NA | NA | NA | NA | NA | 1.172788 |
| Primates | Lorisidae | Loris | tardigradus | Loris tardigradus | 249.22 | 1.44 | 9633.79 | 147.27 | 25.228 | 1 | 4 | 1 | 2 | 2 | 1.685172 |
| Rodentia | Cricetidae | Melanomys | zunigae | Melanomys zunigae | 53.5 | NA | 9675.46 | 94.22 | 9.723 | NA | NA | NA | NA | NA | 1.505484 |
| Rodentia | Ctenomyidae | Ctenomys | emilianus | Ctenomys emilianus | 285.29 | NA | 9676.88 | 20.64 | 6.613 | NA | 3 | 1 | 1 | 1 | 0.169183 |
| Soricomorpha | Soricidae | Crocidura | aleksandrisi | Crocidura aleksandrisi | NA | NA | 9752.34 | 1.21 | 20.556 | NA | NA | NA | NA | NA | 0.01789 |
| Diprotodontia | Pseudocheiridae | Pseudochirulus | cinereus | Pseudochirulus cinereus | 977.48 | 2 | 9764.16 | 108.26 | 22.551 | NA | 1 | 1 | 2 | 1 | 0.018543 |
| Primates | Cercopithecidae | Chlorocebus | djamdjamensis | Chlorocebus djamdjamensis | NA | NA | 9779.34 | 65.37 | 8.005 | NA | NA | NA | NA | NA | 0.713052 |
| Erinaceomorpha | Erinaceidae | Neohylomys | hainanensis | Neohylomys hainanensis | 52.21 | NA | 9825.71 | 118.88 | 22.562 | 2 | 1 | 2 | 1 | 3 | 1.748142 |
| Rodentia | Sciuridae | Tamias | bulleri | Tamias bulleri | 100 | 2.43 | 9834.59 | 44.11 | 13.411 | NA | NA | 1 | 1 | NA | 0.715785 |
| Soricomorpha | Soricidae | Sylvisorex | howelli | Sylvisorex howelli | NA | NA | 9854.66 | 102.29 | 23.324 | NA | NA | NA | NA | NA | 1.522233 |
| Artiodactyla | Bovidae | Bubalus | mindorensis | Bubalus mindorensis | 254389.7 | 1 | 9912.75 | 196.39 | 23.981 | 2 | 2 | 1 | 1 | 1 | 1.405588 |
| Rodentia | Muridae | Rattus | mindorensis | Rattus mindorensis | NA | 6.4 | 9912.75 | 196.39 | 23.981 | NA | 4 | NA | NA | 2 | 0.223406 |
| Chiroptera | Vespertilionidae | Rhogeessa | genowaysi | Rhogeessa genowaysi | NA | NA | 9923.22 | 189.46 | 23.828 | NA | NA | 1 | 2 | NA | 1.068931 |
| Soricomorpha | Soricidae | Crocidura | sicula | Crocidura sicula | NA | NA | 9968.23 | 52.99 | 16.037 | NA | NA | NA | NA | NA | 0.01789 |
| Rodentia | Nesomyidae | Megadendromus | nikolausi | Megadendromus nikolausi | 52 | NA | 9995.32 | 67.74 | 1.403 | NA | NA | NA | NA | NA | 0.431721 |
| Rodentia | Cricetidae | Reithrodontomys | paradoxus | Reithrodontomys paradoxus | NA | NA | 10040.15 | 197.42 | 24.508 | 1 | NA | 2 | 2 | NA | 0.294962 |
| Soricomorpha | Soricidae | Crocidura | lucina | Crocidura lucina | NA | NA | 10061.72 | 69.92 | 5.652 | NA | NA | NA | NA | NA | 0.89448 |
| Soricomorpha | Soricidae | Crocidura | bottegoides | Crocidura bottegoides | NA | NA | 10135.59 | 72.12 | -1.589 | NA | NA | NA | NA | NA | 1.34172 |
| Soricomorpha | Soricidae | Cryptotis | magna | Cryptotis magna | 7 | 2.99 | 10152.68 | 105.55 | 18.472 | NA | NA | 1 | 1 | NA | 1.141847 |
| Soricomorpha | Soricidae | Chimarrogale | phaeura | Chimarrogale phaeura | NA | NA | 10154.88 | 249.23 | 24.488 | NA | 1 | 2 | 1 | 3 | 1.585281 |
| Rodentia | Cricetidae | Ichthyomys | pittieri | Ichthyomys pittieri | 69.1 | NA | 10167.28 | 105.63 | 24.362 | 1 | 1 | 2 | 1 | 3 | 0.820184 |
| Rodentia | Muridae | Melomys | aerosus | Melomys aerosus | NA | NA | 10170.05 | 277.83 | 24.433 | NA | NA | NA | NA | NA | 1.517627 |
| Chiroptera | Molossidae | Chaerephon | bregullae | Chaerephon bregullae | NA | NA | 10238.58 | 293.36 | 23.63 | NA | NA | NA | NA | NA | 1.128758 |
| Rodentia | Octodontidae | Aconaemys | sagei | Aconaemys sagei | 96.49 | NA | 10262.34 | 91.85 | 8.377 | 3 | NA | 2 | 1 | NA | 0.343668 |
| Rodentia | Muridae | Stenocephalemys | ruppi | Stenocephalemys ruppi | 48.99 | NA | 10289.98 | 68.48 | 19.99 | NA | NA | NA | NA | NA | 0.325263 |
| Lagomorpha | Leporidae | Lepus | flavigularis | Lepus flavigularis | 2999.98 | NA | 10300.18 | 169.71 | 24.427 | NA | 2 | NA | NA | 1 | 0.889556 |
| Rodentia | Muridae | Lamottemys | okuensis | Lamottemys okuensis | NA | NA | 10305.29 | 154.51 | 22.773 | NA | NA | NA | NA | NA | 1.483749 |
| Diprotodontia | Pseudocheiridae | Pseudochirulus | herbertensis | Pseudochirulus herbertensis | 1098.34 | 1.89 | 10352.35 | 126.29 | 20.901 | NA | NA | 1 | 2 | NA | 0.018543 |
| Rodentia | Sciuridae | Hyosciurus | ileile | Hyosciurus ileile | NA | NA | 10386.52 | 226.57 | 21.228 | NA | NA | NA | NA | NA | 0.929012 |
| Artiodactyla | Bovidae | Bison | bison | Bison bison | 624577.1 | 0.98 | 10388.04 | 33.59 | -0.535 | 2 | 2 | 2 | 1 | 1 | 0.143647 |
| Soricomorpha | Talpidae | Uropsilus | andersoni | Uropsilus andersoni | NA | NA | 10488.24 | 131.36 | 12.397 | NA | NA | NA | NA | NA | 0.383381 |
| Paucituberculata | Caenolestidae | Caenolestes | caniventer | Caenolestes caniventer | 40 | 2 | 10517.56 | 72.73 | 20.245 | 2 | 3 | 1 | 1 | 2 | 0.242112 |
| Rodentia | Cricetidae | Deltamys | kempi | Deltamys kempi | 26.4 | NA | 10556.19 | 81.02 | 16.083 | NA | NA | 1 | 1 | NA | 0.016032 |
| Primates | Pitheciidae | Callicebus | baptista | Callicebus baptista | NA | NA | 10656.1 | 141.91 | 26.258 | NA | NA | NA | NA | NA | 0.012669 |
| Rodentia | Muridae | Pogonomelomys | bruijni | Pogonomelomys bruijni | NA | NA | 10683.24 | 296.04 | 25.319 | NA | NA | 1 | 2 | NA | 0.018693 |
| Rodentia | Muridae | Gerbillus | muriculus | Gerbillus muriculus | NA | NA | 10686.78 | 18.7 | 24.439 | NA | NA | NA | NA | NA | 0.35571 |
| Diprotodontia | Macropodidae | Dendrolagus | matschiei | Dendrolagus matschiei | 8293.4 | 1.01 | 10844.37 | 285.33 | 22.582 | 2 | 2 | 2 | 2 | 1 | 0.978139 |
| Chiroptera | Pteropodidae | Notopteris | neocaledonica | Notopteris neocaledonica | NA | 1 | 10854.67 | 87.2 | 21.001 | NA | NA | NA | NA | NA | 0.960558 |
| Primates | Cercopithecidae | Trachypithecus | geei | Trachypithecus geei | 8356 | 1.02 | 10913.89 | 147.91 | 17.584 | 3 | NA | NA | NA | NA | 0.664806 |
| Diprotodontia | Macropodidae | Dendrolagus | bennettianus | Dendrolagus bennettianus | 10458.82 | 1 | 10942.65 | 126.88 | 21.613 | NA | NA | 2 | 2 | NA | 0.172452 |
| Chiroptera | Pteropodidae | Pteropus | anetianus | Pteropus anetianus | 397.22 | 0.98 | 10979.84 | 307 | 22.5 | NA | NA | 1 | 2 | NA | 0.717582 |
| Rodentia | Capromyidae | Geocapromys | brownii | Geocapromys brownii | 1497.48 | 1.73 | 11081.39 | 172.66 | 24.033 | 1 | 4 | 2 | 2 | 1 | 0.788262 |
| Chiroptera | Vespertilionidae | Lasiurus | degelidus | Lasiurus degelidus | NA | NA | 11081.39 | 172.66 | 24.033 | NA | NA | NA | NA | NA | 0.712893 |
| Chiroptera | Natalidae | Natalus | jamaicensis | Natalus jamaicensis | NA | NA | 11081.39 | 172.66 | 24.033 | NA | NA | NA | NA | NA | 2.135353 |
| Chiroptera | Phyllostomidae | Phyllonycteris | aphylla | Phyllonycteris aphylla | 14.15 | 0.99 | 11081.39 | 172.66 | 24.033 | 2 | 3 | 1 | 2 | 2 | 0.016863 |
| Rodentia | Muridae | Gerbillus | acticola | Gerbillus acticola | NA | NA | 11121.72 | 12.85 | 24.811 | NA | NA | NA | NA | NA | 0.35571 |
| Rodentia | Ctenomyidae | Ctenomys | dorsalis | Ctenomys dorsalis | 165.6 | NA | 11182.33 | 67.11 | 25.06 | NA | 3 | 1 | 1 | 1 | 0.270692 |
| Lagomorpha | Leporidae | Lepus | hainanus | Lepus hainanus | 1511.67 | NA | 11194.41 | 118.64 | 22.88 | 1 | NA | NA | NA | NA | 0.844374 |
| Rodentia | Chinchillidae | Lagidium | wolffsohni | Lagidium wolffsohni | 2682 | NA | 11203.88 | 34.2 | 4.31 | 3 | NA | NA | NA | NA | 0.317678 |
| Rodentia | Sciuridae | Spermophilus | townsendii | Spermophilus townsendii | 207.29 | 8.49 | 11270.71 | 21.33 | 9.931 | 3 | 5 | 2 | 1 | 2 | 0.567981 |
| Rodentia | Heteromyidae | Dipodomys | nitratoides | Dipodomys nitratoides | 41.59 | 2.14 | 11321.45 | 37.6 | 14.304 | 1 | 2 | 2 | 1 | 1 | 0.696384 |
| Rodentia | Muridae | Apomys | datae | Apomys datae | 34.88 | NA | 11324.99 | 259.41 | 21.039 | 1 | NA | 2 | 2 | NA | 0.013222 |
| Chiroptera | Pteropodidae | Pteropus | mahaganus | Pteropus mahaganus | 297.28 | NA | 11364.47 | 185.99 | 26.511 | NA | NA | 1 | 2 | NA | 0.917188 |
| Carnivora | Canidae | Canis | simensis | Canis simensis | 14361.86 | NA | 11402.81 | 83.87 | 9.903 | 3 | 1 | 1 | 1 | 3 | 0.787706 |
| Rodentia | Spalacidae | Tachyoryctes | macrocephalus | Tachyoryctes macrocephalus | 621.88 | 1 | 11453.57 | 69.99 | 1.553 | 3 | 2 | 2 | 1 | 1 | 1.580563 |
| Soricomorpha | Soricidae | Crocidura | palawanensis | Crocidura palawanensis | NA | NA | 11562.34 | 224 | 24.978 | NA | NA | NA | NA | NA | 0.01789 |
| Rodentia | Sciuridae | Sundasciurus | rabori | Sundasciurus rabori | NA | NA | 11562.34 | 224 | 24.978 | NA | NA | NA | NA | NA | 0.330157 |
| Rodentia | Sciuridae | Hylopetes | nigripes | Hylopetes nigripes | NA | NA | 11578.53 | 224 | 24.978 | 1 | 5 | 1 | 2 | 2 | 0.13988 |
| Rodentia | Cricetidae | Rheomys | underwoodi | Rheomys underwoodi | NA | NA | 11619.42 | 293.09 | 26.272 | NA | 1 | 2 | 1 | 3 | 0.016404 |
| Rodentia | Muridae | Dipodillus | somalicus | Dipodillus somalicus | NA | NA | 11653.7 | 13.85 | 23.97 | NA | NA | NA | NA | NA | 0.35571 |
| Rodentia | Nesomyidae | Brachyuromys | ramirohitra | Brachyuromys ramirohitra | 92.31 | NA | 11677.6 | 169.25 | 18.549 | 2 | NA | 1 | 1 | NA | 0.019353 |
| Rodentia | Cricetidae | Oryzomys | auriventer | Oryzomys auriventer | 60.5 | NA | 11759.41 | 272.71 | 18.069 | NA | NA | NA | NA | NA | 0.01522 |
| Chiroptera | Vespertilionidae | Kerivoula | myrella | Kerivoula myrella | NA | NA | 11762.42 | 212.31 | 25.141 | NA | NA | 1 | 2 | NA | 0.25096 |
| Rodentia | Muridae | Solomys | ponceleti | Solomys ponceleti | 1000 | NA | 11862.56 | 182.39 | 26.139 | NA | 1 | NA | NA | 1 | 1.785232 |
| Rodentia | Muridae | Solomys | salebrosus | Solomys salebrosus | 397 | NA | 11862.56 | 182.39 | 26.139 | NA | NA | NA | NA | NA | 1.409394 |
| Scandentia | Tupaiidae | Tupaia | palawanensis | Tupaia palawanensis | 168.05 | NA | 11882.47 | 224 | 24.978 | 3 | 4 | NA | NA | 2 | 0.020975 |
| Artiodactyla | Tragulidae | Tragulus | nigricans | Tragulus nigricans | NA | NA | 11882.48 | 224 | 24.978 | NA | NA | NA | NA | NA | 1.457311 |
| Rodentia | Cricetidae | Scolomys | melanops | Scolomys melanops | 26.5 | NA | 11901.21 | 222.99 | 17.709 | NA | NA | NA | NA | NA | 0.017303 |
| Artiodactyla | Bovidae | Capricornis | swinhoei | Capricornis swinhoei | NA | NA | 11915.86 | 165.28 | 14.886 | NA | NA | NA | NA | NA | 0.012857 |
| Diprotodontia | Petauridae | Dactylopsila | megalura | Dactylopsila megalura | NA | NA | 11955.72 | 213.61 | 18.148 | 1 | 3 | 1 | 2 | 2 | 0.021283 |
| Pholidota | Manidae | Manis | culionensis | Manis culionensis | NA | NA | 11958.33 | 224 | 24.978 | NA | NA | NA | NA | NA | 0.213259 |
| Chiroptera | Vespertilionidae | Nyctophilus | microdon | Nyctophilus microdon | NA | NA | 11958.72 | 373.05 | 13.673 | NA | NA | 1 | 2 | NA | 0.257632 |
| Soricomorpha | Soricidae | Myosorex | okuensis | Myosorex okuensis | NA | NA | 12051.23 | 216.66 | 22.31 | NA | NA | NA | NA | NA | 1.522191 |
| Rodentia | Heteromyidae | Dipodomys | elator | Dipodomys elator | 105.82 | 2.91 | 12077.2 | 54.5 | 17.591 | 1 | 4 | NA | NA | 2 | 0.749687 |
| Diprotodontia | Macropodidae | Dendrolagus | lumholtzi | Dendrolagus lumholtzi | 6649.97 | 1 | 12095.48 | 152.07 | 21.354 | 1 | NA | 2 | 2 | NA | 0.016479 |
| Rodentia | Muridae | Mammelomys | lanosus | Mammelomys lanosus | 117.1 | 2.68 | 12239.07 | 214.53 | 19.78 | NA | 3 | NA | NA | 1 | 0.020235 |
| Rodentia | Cricetidae | Neotoma | palatina | Neotoma palatina | 198 | NA | 12276.38 | 55.03 | 16.26 | NA | NA | NA | NA | NA | 0.781858 |
| Chiroptera | Pteropodidae | Melonycteris | woodfordi | Melonycteris woodfordi | 36.2 | 0.98 | 12286.51 | 185.99 | 26.511 | NA | 1 | 1 | 2 | 1 | 0.019211 |
| Rodentia | Muridae | Melomys | bougainville | Melomys bougainville | 85.99 | NA | 12465.16 | 182.39 | 26.139 | NA | NA | NA | NA | NA | 0.32376 |
| Carnivora | Mephitidae | Mydaus | marchei | Mydaus marchei | 2500 | NA | 12526.74 | 224 | 24.978 | 2 | NA | 1 | 1 | NA | 0.019638 |
| Rodentia | Sciuridae | Spermophilus | atricapillus | Spermophilus atricapillus | 550.99 | NA | 12535.95 | 9.07 | 19.381 | NA | NA | NA | NA | NA | 0.775727 |
| Rodentia | Sciuridae | Microsciurus | santanderensis | Microsciurus santanderensis | 99.79 | NA | 12536.28 | 106.36 | 13.627 | 3 | NA | NA | NA | NA | 0.172184 |
| Rodentia | Muridae | Gerbillus | pulvinatus | Gerbillus pulvinatus | NA | NA | 12542.05 | 46.89 | 25.823 | NA | NA | NA | NA | NA | 0.022232 |
| Primates | Cebidae | Saguinus | tripartitus | Saguinus tripartitus | 385.05 | NA | 12544.34 | 331.24 | 26.537 | 3 | NA | 1 | 2 | NA | 0.143307 |
| Rodentia | Cricetidae | Peromyscus | ochraventer | Peromyscus ochraventer | 40 | 4.86 | 12708.86 | 52.69 | 21.738 | 1 | NA | NA | NA | NA | 1.336841 |
| Primates | Cheirogaleidae | Cheirogaleus | sibreei | Cheirogaleus sibreei | NA | NA | 12712.93 | 154.45 | 16.911 | NA | NA | NA | NA | NA | 0.348559 |
| Paucituberculata | Caenolestidae | Caenolestes | convelatus | Caenolestes convelatus | 31.56 | NA | 12725.49 | 142.14 | 16.906 | 2 | 1 | 1 | 1 | 3 | 1.210561 |
| Primates | Lemuridae | Eulemur | collaris | Eulemur collaris | NA | NA | 12809.32 | 155.37 | 20.221 | NA | NA | NA | NA | NA | 0.860187 |
| Rodentia | Spalacidae | Spalax | arenarius | Spalax arenarius | NA | NA | 12814.55 | 30.84 | 9.359 | NA | NA | NA | NA | NA | 1.573892 |
| Primates | Cercopithecidae | Macaca | cyclopis | Macaca cyclopis | 5748.94 | 1 | 12815.11 | 161.86 | 12.952 | 3 | NA | NA | NA | NA | 0.014222 |
| Chiroptera | Pteropodidae | Acerodon | leucotis | Acerodon leucotis | NA | NA | 12831.86 | 224 | 24.978 | NA | NA | 1 | 2 | NA | 0.764458 |
| Rodentia | Muridae | Chiropodomys | calamianensis | Chiropodomys calamianensis | 28.9 | NA | 12831.87 | 224 | 24.978 | NA | NA | NA | NA | NA | 0.291415 |
| Rodentia | Hystricidae | Hystrix | pumila | Hystrix pumila | 3544.26 | NA | 12831.87 | 224 | 24.978 | NA | 6 | 2 | 1 | 2 | 0.984245 |
| Soricomorpha | Soricidae | Crocidura | negrina | Crocidura negrina | 11.33 | NA | 12874.45 | 144.35 | 25.623 | NA | NA | NA | NA | NA | 1.34172 |
| Primates | Indriidae | Propithecus | edwardsi | Propithecus edwardsi | 6573.27 | NA | 12906.49 | 167.26 | 18.301 | 3 | NA | NA | NA | NA | 1.393428 |
| Rodentia | Cricetidae | Paralomys | gerbillus | Paralomys gerbillus | 17.4 | NA | 13008.5 | 3.89 | 13.199 | NA | NA | NA | NA | NA | 0.016023 |
| Rodentia | Muridae | Gerbillus | principulus | Gerbillus principulus | NA | NA | 13010.3 | 13.55 | 25.071 | NA | NA | NA | NA | NA | 0.35571 |
| Primates | Cercopithecidae | Macaca | nigra | Macaca nigra | 7359.39 | 1.01 | 13015.44 | 230.8 | 22.539 | 3 | 1 | NA | NA | 1 | 1.226274 |
| Chiroptera | Vespertilionidae | Myotis | peninsularis | Myotis peninsularis | NA | NA | 13115.08 | 17.63 | 20.582 | NA | NA | 1 | 2 | NA | 1.296024 |
| Afrosoricida | Tenrecidae | Oryzorictes | tetradactylus | Oryzorictes tetradactylus | 35.99 | NA | 13130.86 | 165.62 | 18.382 | 1 | NA | NA | NA | NA | 0.389263 |
| Rodentia | Nesomyidae | Prionomys | batesi | Prionomys batesi | 12.5 | NA | 13174.7 | 131.99 | 25.008 | NA | NA | NA | NA | NA | 0.431721 |
| Rodentia | Muridae | Maxomys | panglima | Maxomys panglima | 158.96 | NA | 13227.89 | 224 | 24.978 | NA | NA | NA | NA | NA | 0.021458 |
| Artiodactyla | Suidae | Babyrousa | babyrussa | Babyrousa babyrussa | 92950.09 | 1.73 | 13309.7 | 281.24 | 22.3 | 3 | 6 | 1 | 1 | 2 | 1.070056 |
| Soricomorpha | Soricidae | Crocidura | foxi | Crocidura foxi | 21 | 1.99 | 13366.6 | 112.66 | 24.799 | NA | NA | NA | NA | NA | 0.01789 |
| Carnivora | Mustelidae | Melogale | orientalis | Melogale orientalis | NA | NA | 13388.78 | 201.99 | 23.822 | NA | NA | NA | NA | NA | 0.30684 |
| Chiroptera | Phyllostomidae | Lonchorhina | fernandezi | Lonchorhina fernandezi | NA | NA | 13450.68 | 231.75 | 25.225 | NA | NA | 1 | 2 | NA | 1.573831 |
| Artiodactyla | Bovidae | Rupicapra | pyrenaica | Rupicapra pyrenaica | 29999.99 | NA | 13561.93 | 69.46 | 8.504 | NA | NA | NA | NA | NA | 0.016845 |
| Chiroptera | Rhinolophidae | Rhinolophus | shortridgei | Rhinolophus shortridgei | NA | NA | 13590.38 | 94.3 | 26.382 | NA | NA | NA | NA | NA | 0.018157 |
| Diprotodontia | Pseudocheiridae | Pseudochirops | archeri | Pseudochirops archeri | 1145.92 | 1.03 | 13596.8 | 136.25 | 20.979 | NA | 1 | 1 | 2 | 1 | 0.022331 |
| Primates | Cercopithecidae | Macaca | maura | Macaca maura | 7290.3 | 1.01 | 13648.67 | 231.33 | 23.75 | 3 | NA | NA | NA | NA | 1.072604 |
| Rodentia | Echimyidae | Olallamys | edax | Olallamys edax | 206.4 | NA | 13734.28 | 106.56 | 23.286 | 1 | NA | 1 | 2 | NA | 0.255254 |
| Rodentia | Nesomyidae | Dendromus | oreas | Dendromus oreas | NA | NA | 13754.34 | 251.22 | 23.081 | NA | NA | NA | NA | NA | 1.127357 |
| Chiroptera | Pteropodidae | Pteropus | woodfordi | Pteropus woodfordi | 122.67 | NA | 13791.81 | 196.99 | 25.868 | NA | NA | 1 | 2 | NA | 0.917188 |
| Afrosoricida | Chrysochloridae | Neamblysomus | julianae | Neamblysomus julianae | 21.99 | 0.96 | 13822.31 | 52.63 | 18.978 | NA | 1 | 1 | 1 | 3 | 1.101857 |
| Rodentia | Muridae | Papagomys | armandvillei | Papagomys armandvillei | NA | NA | 14026.28 | 214.33 | 23.567 | NA | NA | NA | NA | NA | 0.201413 |
| Carnivora | Viverridae | Viverra | civettina | Viverra civettina | 12061.27 | NA | 14075.26 | 192.47 | 25.789 | 1 | 6 | 1 | 1 | 2 | 1.529763 |
| Soricomorpha | Soricidae | Paracrocidura | maxima | Paracrocidura maxima | NA | NA | 14075.87 | 117.74 | 18.625 | NA | NA | NA | NA | NA | 0.157313 |
| Rodentia | Muridae | Gerbillus | dunni | Gerbillus dunni | NA | NA | 14101.39 | 15.09 | 26.43 | NA | NA | NA | NA | NA | 0.022232 |
| Rodentia | Muridae | Maxomys | inas | Maxomys inas | 89.99 | 2.91 | 14105.16 | 202.53 | 25.018 | NA | NA | NA | NA | NA | 0.021458 |
| Chiroptera | Molossidae | Mops | trevori | Mops trevori | NA | 0.98 | 14117.02 | 123.91 | 23.699 | NA | NA | 1 | 2 | NA | 0.277788 |
| Rodentia | Muridae | Otomys | burtoni | Otomys burtoni | NA | NA | 14149.86 | 254.63 | 23.624 | NA | NA | NA | NA | NA | 1.28858 |
| Rodentia | Muridae | Pseudomys | johnsoni | Pseudomys johnsoni | 12 | NA | 14173.68 | 31.54 | 24.762 | NA | NA | NA | NA | NA | 0.021649 |
| Soricomorpha | Soricidae | Crocidura | allex | Crocidura allex | NA | 3.99 | 14179.21 | 66.32 | 18.098 | NA | NA | NA | NA | NA | 0.89448 |
| Rodentia | Sciuridae | Syntheosciurus | brochus | Syntheosciurus brochus | NA | NA | 14181.81 | 241.53 | 25.158 | 2 | 3 | 2 | 2 | 1 | 0.120317 |
| Diprotodontia | Macropodidae | Onychogalea | fraenata | Onychogalea fraenata | 4952.05 | 1 | 14215.63 | 54.76 | 20.511 | NA | 1 | 1 | 1 | 1 | 1.035019 |
| Carnivora | Viverridae | Paradoxurus | jerdoni | Paradoxurus jerdoni | 3530.33 | 3 | 14283.81 | 167.05 | 25.701 | 1 | 6 | 1 | 1 | 2 | 0.017784 |
| Afrosoricida | Tenrecidae | Microgale | taiva | Microgale taiva | 12.4 | NA | 14300.46 | 177.7 | 19.709 | NA | NA | NA | NA | NA | 0.020969 |
| Artiodactyla | Cervidae | Muntiacus | feae | Muntiacus feae | 21999.99 | 1.22 | 14346.43 | 393.21 | 24.537 | NA | 4 | NA | NA | 2 | 0.224745 |
| Afrosoricida | Tenrecidae | Limnogale | mergulus | Limnogale mergulus | 76.86 | 2.73 | 14350.29 | 153.37 | 17.893 | 1 | 2 | 2 | 1 | 3 | 1.416125 |
| Rodentia | Muridae | Vandeleuria | nolthenii | Vandeleuria nolthenii | NA | 4 | 14381.82 | 155.65 | 25.316 | NA | NA | NA | NA | NA | 1.448223 |
| Afrosoricida | Tenrecidae | Microgale | thomasi | Microgale thomasi | 22.9 | NA | 14411.05 | 183 | 19.584 | NA | NA | 1 | 1 | NA | 0.021013 |
| Carnivora | Viverridae | Diplogale | hosei | Diplogale hosei | NA | NA | 14471.88 | 257.68 | 23.132 | 1 | 6 | 1 | 1 | 2 | 1.031593 |
| Rodentia | Cricetidae | Microtus | felteni | Microtus felteni | NA | NA | 14500.07 | 50.81 | 6.64 | NA | NA | NA | NA | NA | 0.223415 |
| Primates | Cebidae | Callithrix | leucippe | Callithrix leucippe | NA | NA | 14616.07 | 171.99 | 25.12 | 3 | NA | NA | NA | NA | 0.547035 |
| Paucituberculata | Caenolestidae | Rhyncholestes | raphanurus | Rhyncholestes raphanurus | 21.94 | NA | 14638.53 | 159.95 | 8.578 | 2 | 6 | 2 | 2 | 2 | 0.260193 |
| Chiroptera | Phyllostomidae | Brachyphylla | cavernarum | Brachyphylla cavernarum | 45.5 | 1 | 14649.83 | 104.75 | 24 | NA | 3 | 1 | 2 | 2 | 0.018591 |
| Rodentia | Cricetidae | Eligmodontia | moreni | Eligmodontia moreni | 18 | NA | 14673.54 | 48.85 | 4.143 | NA | NA | NA | NA | NA | 0.014905 |
| Artiodactyla | Cervidae | Mazama | rufina | Mazama rufina | 21099.25 | 1.22 | 14703.63 | 176.24 | 13.258 | NA | NA | 1 | 1 | NA | 0.39668 |
| Primates | Cercopithecidae | Cercopithecus | solatus | Cercopithecus solatus | 5256.91 | 1.01 | 14715.81 | 173.49 | 24.816 | 3 | NA | NA | NA | NA | 0.537021 |
| Soricomorpha | Soricidae | Cryptotis | gracilis | Cryptotis gracilis | NA | NA | 14725.54 | 262.64 | 25.218 | NA | NA | NA | NA | NA | 0.981277 |
| Rodentia | Muridae | Gerbillus | hesperinus | Gerbillus hesperinus | NA | NA | 14744.63 | 33.05 | 16.239 | NA | NA | NA | NA | NA | 1.667391 |
| Chiroptera | Pteropodidae | Pteropus | capistratus | Pteropus capistratus | NA | NA | 14821.62 | 222.33 | 25.025 | NA | NA | NA | NA | NA | 1.202821 |
| Rodentia | Muridae | Niviventer | lepturus | Niviventer lepturus | NA | NA | 14861.73 | 215.61 | 23.345 | NA | NA | NA | NA | NA | 0.021458 |
| Rodentia | Cricetidae | Rheomys | mexicanus | Rheomys mexicanus | 40 | NA | 14942.63 | 91.79 | 19.84 | NA | 2 | 2 | 1 | 3 | 1.230277 |
| Rodentia | Cricetidae | Punomys | lemminus | Punomys lemminus | 84.8 | 1.94 | 14988.79 | 170.51 | 2.71 | 3 | NA | 1 | 1 | NA | 0.990736 |
| Primates | Cercopithecidae | Trachypithecus | johnii | Trachypithecus johnii | 10595.08 | 1.02 | 15101.08 | 128.04 | 25.655 | 3 | 1 | NA | NA | 1 | 0.730523 |
| Rodentia | Muridae | Lemniscomys | roseveari | Lemniscomys roseveari | NA | NA | 15230.39 | 94.37 | 20.619 | NA | NA | NA | NA | NA | 0.311981 |
| Rodentia | Muridae | Maxomys | bartelsii | Maxomys bartelsii | 88.46 | NA | 15245.13 | 232.9 | 23.116 | NA | NA | NA | NA | NA | 0.021458 |
| Chiroptera | Hipposideridae | Anthops | ornatus | Anthops ornatus | NA | NA | 15373.46 | 189.75 | 26.279 | NA | NA | 1 | 2 | NA | 0.408294 |
| Rodentia | Cricetidae | Megadontomys | nelsoni | Megadontomys nelsoni | NA | NA | 15398.19 | 132.75 | 20.258 | NA | NA | NA | NA | NA | 1.264304 |
| Rodentia | Cricetidae | Habromys | simulatus | Habromys simulatus | 40 | NA | 15451.67 | 110.91 | 19.503 | NA | NA | NA | NA | NA | 1.356983 |
| Chiroptera | Hipposideridae | Hipposideros | halophyllus | Hipposideros halophyllus | 4 | NA | 15830.68 | 161.08 | 26.78 | NA | 1 | 1 | 2 | 3 | 1.836176 |
| Primates | Hylobatidae | Nomascus | hainanus | Nomascus hainanus | NA | NA | 15875.47 | 117.65 | 21.793 | NA | NA | NA | NA | NA | 1.352656 |
| Rodentia | Cricetidae | Oryzomys | devius | Oryzomys devius | NA | NA | 15901.86 | 245.91 | 24.961 | NA | NA | NA | NA | NA | 0.01522 |
| Lagomorpha | Leporidae | Nesolagus | netscheri | Nesolagus netscheri | 1511.67 | NA | 15903.21 | 269.74 | 23.881 | 1 | 2 | 2 | 1 | 1 | 1.10269 |
| Dasyuromorphia | Dasyuridae | Sminthopsis | butleri | Sminthopsis butleri | 25 | NA | 15927.83 | 94.95 | 28.059 | 2 | NA | 1 | 1 | NA | 0.760763 |
| Rodentia | Muridae | Hybomys | badius | Hybomys badius | NA | NA | 15939.64 | 182.01 | 21.866 | NA | NA | NA | NA | NA | 1.36626 |
| Rodentia | Muridae | Grammomys | minnae | Grammomys minnae | 35.99 | NA | 16054.94 | 66.87 | 17.912 | NA | NA | NA | NA | NA | 1.013417 |
| Diprotodontia | Macropodidae | Thylogale | lanatus | Thylogale lanatus | NA | NA | 16143.32 | 285.22 | 21.732 | NA | NA | NA | NA | NA | 1.021887 |
| Soricomorpha | Soricidae | Chimarrogale | sumatrana | Chimarrogale sumatrana | NA | NA | 16144.13 | 308.06 | 23.585 | NA | 1 | 2 | 1 | 3 | 0.338193 |
| Artiodactyla | Bovidae | Hemitragus | jayakari | Hemitragus jayakari | 22210.18 | 1 | 16233.68 | 6.05 | 22.021 | 2 | 3 | 1 | 1 | 1 | 1.054269 |
| Chiroptera | Hipposideridae | Hipposideros | dinops | Hipposideros dinops | NA | NA | 16310.89 | 193.88 | 26.15 | NA | 1 | 1 | 2 | 3 | 0.336861 |
| Rodentia | Heteromyidae | Dipodomys | venustus | Dipodomys venustus | 81.52 | 1.73 | 16390.36 | 37.96 | 12.343 | NA | 2 | 2 | 1 | 1 | 0.013174 |
| Rodentia | Muridae | Gerbillus | nancillus | Gerbillus nancillus | NA | NA | 16446.8 | 22.73 | 24.355 | NA | NA | NA | NA | NA | 0.35571 |
| Chiroptera | Vespertilionidae | Chalinolobus | neocaledonicus | Chalinolobus neocaledonicus | NA | NA | 16470.95 | 87.85 | 20.531 | NA | NA | NA | NA | NA | 1.222822 |
| Chiroptera | Pteropodidae | Pteropus | vetulus | Pteropus vetulus | 151.54 | NA | 16470.95 | 87.85 | 20.531 | NA | NA | 1 | 2 | NA | 0.758748 |
| Rodentia | Muridae | Mus | goundae | Mus goundae | NA | NA | 16481.08 | 102.1 | 26.572 | NA | NA | NA | NA | NA | 0.250107 |
| Rodentia | Muridae | Taterillus | petteri | Taterillus petteri | NA | NA | 16500.23 | 44.46 | 28.744 | NA | NA | NA | NA | NA | 0.019791 |
| Chiroptera | Vespertilionidae | Pipistrellus | wattsi | Pipistrellus wattsi | NA | NA | 16587.76 | 207.15 | 21.144 | NA | NA | NA | NA | NA | 0.015988 |
| Primates | Hominidae | Pongo | abelii | Pongo abelii | 39696.12 | NA | 16756.21 | 140.16 | 22.353 | NA | NA | NA | NA | NA | 1.769107 |
| Primates | Cheirogaleidae | Microcebus | griseorufus | Microcebus griseorufus | 70.24 | NA | 17072.63 | 49.9 | 23.88 | NA | NA | NA | NA | NA | 0.020293 |
| Carnivora | Eupleridae | Mungotictis | decemlineata | Mungotictis decemlineata | 657.03 | 0.99 | 17099.69 | 63.23 | 24.132 | 3 | 2 | 2 | 2 | 3 | 1.073209 |
| Rodentia | Cricetidae | Peromyscus | simulus | Peromyscus simulus | 40 | NA | 17160.55 | 67.39 | 22.8 | 1 | NA | NA | NA | NA | 0.891228 |
| Primates | Pitheciidae | Callicebus | modestus | Callicebus modestus | 992.4 | 1.02 | 17288.42 | 119.01 | 22.542 | 3 | NA | 1 | 2 | NA | 1.184427 |
| Primates | Pitheciidae | Callicebus | olallae | Callicebus olallae | 992.4 | 1.02 | 17288.42 | 119.01 | 22.542 | 3 | NA | 1 | 2 | NA | 1.184427 |
| Rodentia | Sciuridae | Spermophilus | washingtoni | Spermophilus washingtoni | 215.09 | 7.88 | 17288.55 | 24.37 | 10.046 | 3 | 7 | 2 | 1 | 2 | 0.11599 |
| Rodentia | Muridae | Apomys | hylocetes | Apomys hylocetes | 34.88 | NA | 17394.58 | 184.2 | 22.969 | 1 | NA | 2 | 2 | NA | 0.013222 |
| Rodentia | Cricetidae | Akodon | surdus | Akodon surdus | 38.99 | NA | 17420.46 | 72.79 | 17.864 | NA | NA | NA | NA | NA | 0.674588 |
| Rodentia | Sciuridae | Hyosciurus | heinrichi | Hyosciurus heinrichi | NA | NA | 17668.93 | 218.92 | 18.554 | NA | NA | NA | NA | NA | 0.01858 |
| Rodentia | Geomyidae | Cratogeomys | merriami | Cratogeomys merriami | 419.99 | 1.94 | 17777.06 | 98.4 | 14.951 | NA | 3 | 2 | 1 | 1 | 0.017788 |
| Dasyuromorphia | Dasyuridae | Antechinus | adustus | Antechinus adustus | NA | NA | 17800.49 | 133.36 | 21.12 | NA | NA | NA | NA | NA | 0.014575 |
| Chiroptera | Pteropodidae | Nyctimene | rabori | Nyctimene rabori | 68.25 | 1 | 17815.16 | 140.47 | 25.859 | NA | 1 | 1 | 2 | 1 | 1.212911 |
| Rodentia | Muridae | Maxomys | hylomyoides | Maxomys hylomyoides | NA | NA | 17894.61 | 281.04 | 22.642 | NA | NA | NA | NA | NA | 0.343325 |
| Diprotodontia | Macropodidae | Dendrolagus | ursinus | Dendrolagus ursinus | 13249.99 | 1.01 | 17925.03 | 216.45 | 21.89 | 2 | 1 | 2 | 2 | 1 | 0.775095 |
| Rodentia | Cricetidae | Megadontomys | thomasi | Megadontomys thomasi | 110.52 | 2.6 | 17943.47 | 107.66 | 20.936 | NA | NA | NA | NA | NA | 1.264304 |
| Rodentia | Heteromyidae | Chaetodipus | lineatus | Chaetodipus lineatus | 23 | NA | 18026.29 | 30.66 | 14.454 | 1 | 1 | 2 | 1 | 1 | 0.264013 |
| Rodentia | Sciuridae | Tamias | obscurus | Tamias obscurus | 72.99 | 3.49 | 18069.88 | 15.57 | 13.459 | 3 | 3 | 3 | 2 | 1 | 0.014687 |
| Rodentia | Cricetidae | Thomasomys | rosalinda | Thomasomys rosalinda | 77 | NA | 18091.25 | 124.89 | 15.234 | NA | NA | NA | NA | NA | 0.229903 |
| Peramelemorphia | Peramelidae | Microperoryctes | papuensis | Microperoryctes papuensis | 158 | 1.01 | 18150.86 | 173.81 | 19.617 | 1 | NA | 2 | 1 | NA | 0.01976 |
| Rodentia | Sciuridae | Spermophilus | mohavensis | Spermophilus mohavensis | 213.18 | 6.5 | 18167.56 | 28.05 | 11.5 | 3 | 2 | 2 | 1 | 1 | 0.667083 |
| Rodentia | Muridae | Microdillus | peeli | Microdillus peeli | NA | NA | 18197 | 13.69 | 25.322 | NA | NA | NA | NA | NA | 0.022286 |
| Chiroptera | Pteropodidae | Pteropus | ornatus | Pteropus ornatus | 336.25 | 0.98 | 18308.58 | 87.85 | 20.531 | NA | NA | 1 | 2 | NA | 0.919026 |
| Rodentia | Cricetidae | Abrothrix | sanborni | Abrothrix sanborni | 24.7 | NA | 18335.19 | 133.02 | 7.892 | 2 | 6 | NA | NA | 2 | 0.129784 |
| Rodentia | Muridae | Gerbillus | burtoni | Gerbillus burtoni | NA | NA | 18441.09 | 28.34 | 21.932 | NA | NA | NA | NA | NA | 0.35571 |
| Rodentia | Muridae | Gerbillus | rosalinda | Gerbillus rosalinda | NA | NA | 18621.39 | 40.15 | 24.313 | NA | NA | NA | NA | NA | 0.022232 |
| Chiroptera | Hipposideridae | Hipposideros | durgadasi | Hipposideros durgadasi | NA | NA | 18651.07 | 115.65 | 24.878 | NA | NA | NA | NA | NA | 1.577607 |
| Soricomorpha | Soricidae | Cryptotis | squamipes | Cryptotis squamipes | 11 | NA | 18765.47 | 171.5 | 18.269 | NA | 1 | NA | NA | 3 | 0.019626 |
| Rodentia | Cricetidae | Ichthyomys | tweedii | Ichthyomys tweedii | 118.5 | 1.94 | 18775.04 | 150.14 | 25.6 | 1 | 2 | 2 | 1 | 3 | 0.262459 |
| Soricomorpha | Soricidae | Crocidura | levicula | Crocidura levicula | NA | NA | 18810.7 | 238.8 | 21.48 | NA | NA | NA | NA | NA | 0.01789 |
| Rodentia | Dipodidae | Sicista | kluchorica | Sicista kluchorica | NA | NA | 18842.52 | 62.89 | -0.305 | NA | NA | NA | NA | NA | 0.234295 |
| Didelphimorphia | Didelphidae | Lestodelphys | halli | Lestodelphys halli | 78.27 | NA | 18932.66 | 19.39 | 8.955 | 1 | 3 | NA | NA | 2 | 0.024355 |
| Rodentia | Sciuridae | Tamias | canipes | Tamias canipes | 70.2 | 3.88 | 18973.09 | 23.35 | 13.391 | NA | 5 | 2 | 2 | 2 | 0.015355 |
| Rodentia | Cricetidae | Microtus | kikuchii | Microtus kikuchii | 46.3 | 1.97 | 19032.5 | 160.89 | 15.554 | NA | NA | NA | NA | NA | 0.178791 |
| Primates | Cercopithecidae | Macaca | ochreata | Macaca ochreata | 2745.5 | NA | 19033.57 | 244.11 | 23.765 | 3 | NA | NA | NA | NA | 0.709149 |
| Rodentia | Muridae | Mastomys | kollmannspergeri | Mastomys kollmannspergeri | NA | NA | 19075.98 | 65.36 | 27.959 | NA | NA | NA | NA | NA | 0.020022 |
| Soricomorpha | Talpidae | Dymecodon | pilirostris | Dymecodon pilirostris | 15.46 | 3 | 19085.78 | 137.95 | 7.836 | 2 | 1 | 2 | 1 | 3 | 0.023099 |
| Rodentia | Cricetidae | Peromyscus | guatemalensis | Peromyscus guatemalensis | 40 | 1.94 | 19099.86 | 134.11 | 20.103 | 1 | NA | NA | NA | NA | 0.017825 |
| Rodentia | Gliridae | Myomimus | roachi | Myomimus roachi | NA | NA | 19296.94 | 49.37 | 11.807 | NA | NA | NA | NA | NA | 1.110117 |
| Rodentia | Ctenomyidae | Ctenomys | steinbachi | Ctenomys steinbachi | 384.99 | NA | 19311.11 | 87.36 | 22.289 | NA | 3 | 2 | 1 | 1 | 0.016918 |
| Chiroptera | Pteropodidae | Notopteris | macdonaldi | Notopteris macdonaldi | 68.17 | 0.99 | 19355.8 | 206.67 | 23.667 | NA | NA | 1 | 2 | NA | 0.960558 |
| Soricomorpha | Soricidae | Crocidura | maurisca | Crocidura maurisca | 14 | NA | 19556.07 | 99.73 | 21.639 | NA | NA | NA | NA | NA | 0.01789 |
| Rodentia | Ctenomyidae | Ctenomys | latro | Ctenomys latro | 192 | NA | 19561.91 | 62.72 | 14.436 | NA | 3 | 1 | 1 | 1 | 0.845914 |
| Rodentia | Muridae | Lophuromys | melanonyx | Lophuromys melanonyx | 10.2 | NA | 19567.01 | 68.76 | 3.435 | NA | NA | NA | NA | NA | 0.739171 |
| Soricomorpha | Soricidae | Crocidura | fumosa | Crocidura fumosa | 14.49 | 3.74 | 19575.94 | 77.73 | 17.533 | NA | NA | NA | NA | NA | 0.89448 |
| Chiroptera | Pteropodidae | Pteropus | samoensis | Pteropus samoensis | 309.99 | NA | 19596 | 206.67 | 23.667 | 3 | 2 | 1 | 2 | 1 | 0.143516 |
| Rodentia | Sciuridae | Sundasciurus | samarensis | Sundasciurus samarensis | 225 | NA | 19683.01 | 251.02 | 25.587 | NA | NA | NA | NA | NA | 0.020635 |
| Rodentia | Muridae | Leptomys | elegans | Leptomys elegans | 83.52 | 1.88 | 19694.75 | 197.38 | 21.743 | NA | 2 | 4 | 2 | 3 | 0.017095 |
| Chiroptera | Vespertilionidae | Hypsugo | vordermanni | Hypsugo vordermanni | NA | NA | 19828.26 | 276.72 | 25.672 | NA | NA | NA | NA | NA | 0.28224 |
| Rodentia | Cricetidae | Peromyscus | hooperi | Peromyscus hooperi | 35.99 | 3.09 | 19897.04 | 17.54 | 18.039 | 1 | NA | NA | NA | NA | 0.017825 |
| Rodentia | Cricetidae | Thomasomys | taczanowskii | Thomasomys taczanowskii | 77 | NA | 19972.08 | 45.46 | 14.65 | NA | NA | NA | NA | NA | 0.014369 |
| Chiroptera | Pteropodidae | Syconycteris | carolinae | Syconycteris carolinae | 39.67 | NA | 20131.34 | 192.66 | 25.011 | NA | 1 | 1 | 2 | 1 | 0.943522 |
| Primates | Hylobatidae | Hylobates | moloch | Hylobates moloch | 5860.81 | 1 | 20157.87 | 211.91 | 23.825 | 3 | 4 | NA | NA | 2 | 1.067786 |
| Rodentia | Sciuridae | Hylopetes | bartelsi | Hylopetes bartelsi | NA | NA | 20202.96 | 215.51 | 24.267 | NA | NA | NA | NA | NA | 0.231673 |
| Primates | Cercopithecidae | Semnopithecus | hector | Semnopithecus hector | NA | NA | 20231.94 | 110.6 | 15.89 | NA | NA | NA | NA | NA | 0.13347 |
| Primates | Cercopithecidae | Cercocebus | galeritus | Cercocebus galeritus | 7077.66 | 1.01 | 20246.09 | 53.99 | 27.378 | 3 | 2 | 2 | 2 | 1 | 1.090517 |
| Rodentia | Cricetidae | Akodon | budini | Akodon budini | 26.9 | NA | 20316.47 | 40.9 | -1.127 | NA | NA | NA | NA | NA | 0.013291 |
| Primates | Lemuridae | Hapalemur | occidentalis | Hapalemur occidentalis | NA | NA | 20361.36 | 117.41 | 23.768 | NA | NA | NA | NA | NA | 0.997366 |
| Rodentia | Muridae | Mallomys | istapantap | Mallomys istapantap | 1990.43 | 1.07 | 20432.86 | 235.57 | 14.777 | NA | 2 | 3 | 2 | 1 | 0.018626 |
| Lagomorpha | Leporidae | Sylvilagus | dicei | Sylvilagus dicei | NA | 4.5 | 20643.01 | 274.02 | 24.814 | NA | 2 | 1 | 1 | 1 | 0.229133 |
| Afrosoricida | Chrysochloridae | Chlorotalpa | duthieae | Chlorotalpa duthieae | NA | 2 | 20651.08 | 53.64 | 14.618 | NA | 1 | NA | NA | 3 | 1.202605 |
| Rodentia | Cricetidae | Isthmomys | pirrensis | Isthmomys pirrensis | 137.71 | 2.43 | 20652.28 | 216.88 | 25.191 | NA | 3 | 1 | 1 | 2 | 0.01785 |
| Chiroptera | Pteropodidae | Aethalops | aequalis | Aethalops aequalis | NA | NA | 20667.22 | 270.73 | 22.58 | NA | NA | NA | NA | NA | 0.017299 |
| Rodentia | Cricetidae | Sigmodon | inopinatus | Sigmodon inopinatus | 140.5 | NA | 20681.64 | 212.79 | 14.154 | NA | NA | NA | NA | NA | 0.723034 |
| Rodentia | Muridae | Maxomys | moi | Maxomys moi | NA | NA | 20733.37 | 179.33 | 22.736 | NA | NA | NA | NA | NA | 0.021458 |
| Rodentia | Cricetidae | Reithrodontomys | brevirostris | Reithrodontomys brevirostris | 12.9 | 3.4 | 20739.72 | 191.98 | 22.338 | 1 | NA | 2 | 2 | NA | 0.018435 |
| Rodentia | Muridae | Dasymys | foxi | Dasymys foxi | NA | NA | 20749.75 | 110.41 | 25.422 | NA | NA | NA | NA | NA | 0.277048 |
| Rodentia | Sciuridae | Callosciurus | baluensis | Callosciurus baluensis | 323.91 | 3 | 20853.19 | 271.96 | 22.605 | 3 | 6 | 1 | 2 | 2 | 0.020145 |
| Rodentia | Ctenomyidae | Ctenomys | knighti | Ctenomys knighti | 316 | NA | 20864.38 | 47.45 | 10.848 | NA | 3 | 1 | 1 | 1 | 0.242218 |
| Rodentia | Sciuridae | Callosciurus | quinquestriatus | Callosciurus quinquestriatus | NA | NA | 21108.92 | 136.44 | 7.919 | NA | NA | NA | NA | NA | 0.204697 |
| Rodentia | Cricetidae | Reithrodontomys | creper | Reithrodontomys creper | 22.8 | 2.21 | 21120.56 | 230.99 | 25.092 | 1 | NA | 2 | 2 | NA | 0.016984 |
| Rodentia | Muridae | Leopoldamys | neilli | Leopoldamys neilli | NA | NA | 21254.21 | 147.54 | 26.673 | NA | NA | NA | NA | NA | 0.312984 |
| Rodentia | Cricetidae | Akodon | kofordi | Akodon kofordi | 29.49 | NA | 21268.55 | 333.69 | 8.947 | NA | NA | NA | NA | NA | 0.013492 |
| Rodentia | Cricetidae | Reithrodontomys | tenuirostris | Reithrodontomys tenuirostris | 20 | NA | 21302.1 | 101.14 | 19.602 | 1 | NA | 2 | 2 | NA | 0.849184 |
| Artiodactyla | Bovidae | Capricornis | rubidus | Capricornis rubidus | NA | NA | 21552.31 | 181.29 | 11.503 | NA | NA | NA | NA | NA | 0.126928 |
| Rodentia | Ctenomyidae | Ctenomys | goodfellowi | Ctenomys goodfellowi | NA | NA | 21761.32 | 87.28 | 23.524 | NA | NA | NA | NA | NA | 0.015139 |
| Diprotodontia | Potoroidae | Bettongia | tropica | Bettongia tropica | 1256.58 | 1 | 21795.64 | 82.33 | 24.795 | 1 | NA | NA | NA | NA | 1.278981 |
| Rodentia | Cricetidae | Bibimys | labiosus | Bibimys labiosus | NA | NA | 21850.7 | 115.72 | 20.596 | NA | NA | NA | NA | NA | 0.011944 |
| Primates | Cercopithecidae | Macaca | hecki | Macaca hecki | NA | NA | 21888.19 | 221.77 | 21.866 | 3 | NA | NA | NA | NA | 0.639069 |
| Rodentia | Nesomyidae | Dendromus | kahuziensis | Dendromus kahuziensis | NA | 3.49 | 21928.26 | 129.07 | 20.607 | NA | NA | NA | NA | NA | 2.141978 |
| Rodentia | Cricetidae | Arborimus | pomo | Arborimus pomo | 32.49 | 2 | 22031.98 | 69.74 | 8.568 | 1 | 1 | 2 | 2 | 1 | 0.135792 |
| Diprotodontia | Burramyidae | Burramys | parvus | Burramys parvus | 44.27 | 3.6 | 22142.85 | 64.13 | 9.874 | 1 | 5 | 2 | 2 | 2 | 2.35315 |
| Rodentia | Cricetidae | Thomasomys | pyrrhonotus | Thomasomys pyrrhonotus | 77 | NA | 22177.36 | 46.43 | 17.944 | NA | NA | NA | NA | NA | 0.718446 |
| Diprotodontia | Pseudocheiridae | Hemibelideus | lemuroides | Hemibelideus lemuroides | 993.59 | 1 | 22321.21 | 103.34 | 21.444 | NA | NA | 1 | 2 | NA | 0.198753 |
| Rodentia | Ctenomyidae | Ctenomys | colburni | Ctenomys colburni | 400 | NA | 22326.04 | 12.88 | 4.741 | NA | 3 | 1 | 1 | 1 | 0.270692 |
| Rodentia | Cricetidae | Alticola | roylei | Alticola roylei | 37 | 5.44 | 22396.36 | 102.8 | 5.27 | NA | NA | NA | NA | NA | 0.142709 |
| Rodentia | Muridae | Melomys | dollmani | Melomys dollmani | NA | NA | 22399.9 | 355.32 | 16.655 | NA | NA | NA | NA | NA | 0.020235 |
| Rodentia | Muridae | Dasymys | montanus | Dasymys montanus | NA | NA | 22409.71 | 111.93 | 21.053 | NA | NA | NA | NA | NA | 1.298662 |
| Diprotodontia | Phalangeridae | Phalanger | ornatus | Phalanger ornatus | 1787.5 | NA | 22430.66 | 192.66 | 25.011 | 1 | 1 | 1 | 2 | 1 | 0.019397 |
| Diprotodontia | Pseudocheiridae | Pseudochirops | albertisii | Pseudochirops albertisii | 784.85 | 1.02 | 22493.05 | 210.67 | 24.256 | 2 | 3 | 2 | 2 | 1 | 0.198272 |
| Rodentia | Cricetidae | Peromyscus | furvus | Peromyscus furvus | 32.99 | 1.94 | 22500.45 | 102.43 | 19.912 | 1 | NA | NA | NA | NA | 0.285193 |
| Rodentia | Sciuridae | Tamias | siskiyou | Tamias siskiyou | 75 | NA | 22545.56 | 80.33 | 7.1 | NA | NA | NA | NA | NA | 0.015043 |
| Chiroptera | Pteropodidae | Dobsonia | anderseni | Dobsonia anderseni | 233.99 | NA | 22628.33 | 204.26 | 24.473 | NA | NA | NA | NA | NA | 0.011125 |
| Chiroptera | Vespertilionidae | Eptesicus | japonensis | Eptesicus japonensis | NA | NA | 22821.83 | 136.14 | 7.051 | NA | NA | NA | NA | NA | 1.100491 |
| Primates | Cebidae | Leontopithecus | rosalia | Leontopithecus rosalia | 592.52 | 1.94 | 22874.24 | 128.32 | 20.65 | 3 | 3 | 1 | 2 | 2 | 0.974671 |
| Rodentia | Muridae | Mastomys | shortridgei | Mastomys shortridgei | 46.5 | NA | 22875.26 | 46.3 | 23.469 | NA | NA | NA | NA | NA | 0.020022 |
| Rodentia | Echimyidae | Proechimys | oconnelli | Proechimys oconnelli | 284.99 | NA | 22909.05 | 203.33 | 25.433 | NA | NA | NA | NA | NA | 0.330599 |
| Carnivora | Viverridae | Macrogalidia | musschenbroekii | Macrogalidia musschenbroekii | 5148.86 | NA | 22966.97 | 226.87 | 19.706 | NA | 6 | 1 | 1 | 2 | 1.044088 |
| Primates | Lepilemuridae | Lepilemur | leucopus | Lepilemur leucopus | 599.99 | 1.01 | 22985.68 | 56.82 | 23.113 | 1 | 2 | 1 | 2 | 1 | 0.311582 |
| Diprotodontia | Potoroidae | Bettongia | penicillata | Bettongia penicillata | 1184.35 | 1 | 22986.14 | 64.7 | 14.688 | 1 | NA | NA | NA | NA | 1.620042 |
| Primates | Cercopithecidae | Rhinopithecus | bieti | Rhinopithecus bieti | 11000.54 | NA | 23060.04 | 182.09 | 2.558 | 3 | 5 | NA | NA | 2 | 0.963787 |
| Primates | Pitheciidae | Callicebus | ornatus | Callicebus ornatus | NA | NA | 23066.97 | 130.8 | 20.93 | NA | NA | NA | NA | NA | 0.722095 |
| Rodentia | Cricetidae | Oligoryzomys | vegetus | Oligoryzomys vegetus | NA | NA | 23114.34 | 262.19 | 24.967 | NA | NA | NA | NA | NA | 0.013332 |
| Rodentia | Geomyidae | Geomys | texensis | Geomys texensis | NA | NA | 23475.95 | 55.06 | 17.858 | NA | NA | 2 | 1 | NA | 0.015239 |
| Rodentia | Muridae | Niviventer | cameroni | Niviventer cameroni | NA | NA | 23506.95 | 209.77 | 24.728 | NA | NA | NA | NA | NA | 0.898658 |
| Primates | Lepilemuridae | Lepilemur | edwardsi | Lepilemur edwardsi | 821.58 | NA | 23515.8 | 114.2 | 25.476 | 1 | 5 | 1 | 2 | 2 | 0.973695 |
| Chiroptera | Pteropodidae | Pteropus | intermedius | Pteropus intermedius | NA | NA | 23524.64 | 184.84 | 25.373 | NA | NA | NA | NA | NA | 0.217441 |
| Rodentia | Ctenomyidae | Ctenomys | argentinus | Ctenomys argentinus | 221.3 | NA | 23664.72 | 92.69 | 21.423 | NA | 3 | 1 | 1 | 1 | 0.169183 |
| Didelphimorphia | Didelphidae | Marmosops | invictus | Marmosops invictus | 29.19 | NA | 23715.9 | 210.58 | 25.429 | 1 | 3 | 2 | 2 | 2 | 0.022623 |
| Rodentia | Ctenomyidae | Ctenomys | australis | Ctenomys australis | 403.32 | NA | 23805.63 | 61.85 | 13.934 | NA | 3 | 2 | 1 | 1 | 1.268871 |
| Chiroptera | Vespertilionidae | Myotis | aelleni | Myotis aelleni | NA | NA | 23827.25 | 54.47 | 5.728 | NA | NA | 1 | 2 | NA | 0.276485 |
| Rodentia | Cricetidae | Proedromys | bedfordi | Proedromys bedfordi | NA | NA | 23967.19 | 59.12 | 6.027 | NA | NA | NA | NA | NA | 1.052489 |
| Dasyuromorphia | Dasyuridae | Sminthopsis | archeri | Sminthopsis archeri | 16 | 8.15 | 24146.82 | 123.67 | 26.416 | 2 | 2 | 1 | 1 | 3 | 0.285687 |
| Chiroptera | Pteropodidae | Dobsonia | praedatrix | Dobsonia praedatrix | 178.74 | 1 | 24235.72 | 202.73 | 24.483 | NA | 1 | 1 | 2 | 1 | 0.011041 |
| Rodentia | Muridae | Hyomys | dammermani | Hyomys dammermani | 932.5 | 1.07 | 24335.07 | 200.75 | 20.255 | NA | 3 | 3 | 2 | 1 | 0.311344 |
| Dasyuromorphia | Dasyuridae | Sminthopsis | douglasi | Sminthopsis douglasi | 54.99 | 6.78 | 24392.06 | 42.47 | 24.914 | 2 | 1 | 1 | 1 | 3 | 0.152153 |
| Rodentia | Sciuridae | Cynomys | parvidens | Cynomys parvidens | 899.98 | 4.89 | 24425.78 | 22.76 | 6.149 | 3 | 1 | 2 | 1 | 1 | 0.822976 |
| Chiroptera | Pteropodidae | Pteropus | caniceps | Pteropus caniceps | 525.12 | NA | 24432.1 | 192.66 | 25.011 | NA | NA | 1 | 2 | NA | 0.183805 |
| Soricomorpha | Soricidae | Chimarrogale | hantu | Chimarrogale hantu | 54.99 | NA | 24502.23 | 203.26 | 24.628 | NA | 1 | 2 | 1 | 3 | 0.211371 |
| Artiodactyla | Cervidae | Rusa | alfredi | Rusa alfredi | 46475.04 | NA | 24504.78 | 160.44 | 25.332 | NA | NA | NA | NA | NA | 1.236876 |
| Chiroptera | Molossidae | Otomops | papuensis | Otomops papuensis | NA | NA | 24515.96 | 203.37 | 20.925 | NA | NA | 1 | 2 | NA | 0.294193 |
| Rodentia | Capromyidae | Mesocapromys | melanurus | Mesocapromys melanurus | NA | 1.75 | 24599.31 | 88.48 | 25.648 | NA | 3 | 1 | 2 | 2 | 0.279868 |
| Chiroptera | Molossidae | Chaerephon | johorensis | Chaerephon johorensis | NA | NA | 24699.24 | 210.92 | 25.2 | NA | 1 | 1 | 2 | 3 | 0.902094 |
| Rodentia | Muridae | Mastomys | pernanus | Mastomys pernanus | NA | NA | 24744.58 | 74.76 | 19.788 | NA | NA | NA | NA | NA | 0.320348 |
| Rodentia | Ctenomyidae | Ctenomys | rionegrensis | Ctenomys rionegrensis | NA | NA | 24759.25 | 89.03 | 17.428 | NA | 3 | 1 | 1 | 1 | 1.135396 |
| Perissodactyla | Rhinocerotidae | Rhinoceros | unicornis | Rhinoceros unicornis | 1843656 | 1.41 | 24776.82 | 171.98 | 23.599 | 2 | 3 | 2 | 1 | 1 | 1.148966 |
| Chiroptera | Rhinolophidae | Rhinolophus | formosae | Rhinolophus formosae | NA | NA | 24880.25 | 160.08 | 16.456 | NA | NA | NA | NA | NA | 0.094081 |
| Rodentia | Cricetidae | Thomasomys | notatus | Thomasomys notatus | 77 | NA | 25007.61 | 135.05 | 13.341 | NA | NA | NA | NA | NA | 0.014369 |
| Chiroptera | Hipposideridae | Hipposideros | papua | Hipposideros papua | NA | NA | 25011.58 | 202.64 | 25.007 | NA | 1 | 1 | 2 | 3 | 0.024482 |
| Rodentia | Cricetidae | Oryzomys | hammondi | Oryzomys hammondi | 60.5 | NA | 25037.04 | 133.52 | 18.887 | NA | NA | NA | NA | NA | 1.141482 |
| Soricomorpha | Soricidae | Crocidura | xantippe | Crocidura xantippe | NA | NA | 25181.09 | 76.16 | 23.387 | NA | NA | NA | NA | NA | 0.01789 |
| Rodentia | Muridae | Apodemus | gurkha | Apodemus gurkha | NA | NA | 25245.52 | 114.5 | 5.283 | NA | NA | NA | NA | NA | 1.35887 |
| Rodentia | Cricetidae | Rheomys | thomasi | Rheomys thomasi | 40 | 0.97 | 25340.02 | 125.05 | 21.485 | NA | 2 | 2 | 1 | 3 | 0.164037 |
| Chiroptera | Pteropodidae | Pteropus | personatus | Pteropus personatus | 130.84 | NA | 25383.48 | 201.4 | 25 | NA | NA | 1 | 2 | NA | 0.018348 |
| Rodentia | Muridae | Arvicanthis | blicki | Arvicanthis blicki | 128 | NA | 25420.47 | 71.06 | 6.691 | NA | NA | NA | NA | NA | 0.185532 |
| Soricomorpha | Soricidae | Crocidura | selina | Crocidura selina | NA | NA | 25492.41 | 102 | 22.906 | NA | NA | NA | NA | NA | 0.286234 |
| Rodentia | Sciuridae | Tamias | quadrimaculatus | Tamias quadrimaculatus | 83.62 | 5.93 | 25579.83 | 56.96 | 7.965 | 3 | 5 | 2 | 2 | 2 | 0.016241 |
| Soricomorpha | Soricidae | Cryptotis | montivaga | Cryptotis montivaga | 11.29 | NA | 25609.05 | 107.6 | 16.771 | 2 | 1 | NA | NA | 3 | 0.019626 |
| Monotremata | Tachyglossidae | Zaglossus | bruijni | Zaglossus bruijni | 8951.71 | 1 | 25654.49 | 211.51 | 22.02 | 1 | 1 | 1 | 1 | 3 | 2.660959 |
| Primates | Lemuridae | Eulemur | fulvus | Eulemur fulvus | 2376.99 | 1.1 | 25656.44 | 156.13 | 21.416 | 2 | 3 | 1 | 2 | 1 | 0.172037 |
| Primates | Cercopithecidae | Semnopithecus | hypoleucos | Semnopithecus hypoleucos | 10026.65 | NA | 25713.57 | 191.3 | 25.87 | NA | NA | NA | NA | NA | 0.667351 |
| Artiodactyla | Suidae | Sus | salvanius | Sus salvanius | 7974.13 | 3.66 | 25728.78 | 162.48 | 20.349 | 2 | 4 | 1 | 1 | 2 | 1.759141 |
| Chiroptera | Pteropodidae | Dobsonia | crenulata | Dobsonia crenulata | 218.21 | NA | 25941.38 | 201.4 | 25 | NA | NA | NA | NA | NA | 0.010826 |
| Rodentia | Ctenomyidae | Ctenomys | haigi | Ctenomys haigi | 164 | NA | 25958.86 | 40.32 | 6.947 | NA | NA | 2 | 1 | NA | 0.016918 |
| Chiroptera | Pteropodidae | Pteropus | rayneri | Pteropus rayneri | 661.28 | 0.99 | 26002.69 | 190.69 | 26.236 | NA | NA | 1 | 2 | NA | 0.138031 |
| Chiroptera | Pteropodidae | Pteropus | ocularis | Pteropus ocularis | 228.76 | NA | 26128.32 | 281 | 23.588 | NA | NA | 1 | 2 | NA | 0.764522 |
| Rodentia | Cricetidae | Rhipidomys | venustus | Rhipidomys venustus | 89 | NA | 26254.03 | 112.55 | 22.424 | 1 | NA | 1 | 2 | NA | 0.015785 |
| Rodentia | Cricetidae | Peromyscus | stirtoni | Peromyscus stirtoni | 29.19 | 2.38 | 26436.82 | 115.6 | 21.545 | 1 | NA | NA | NA | NA | 0.017825 |
| Soricomorpha | Soricidae | Crocidura | elongata | Crocidura elongata | NA | NA | 26519.84 | 220.93 | 18.854 | NA | NA | NA | NA | NA | 0.01789 |
| Rodentia | Heteromyidae | Liomys | adspersus | Liomys adspersus | 50.99 | 3.2 | 26538.1 | 192.32 | 24.834 | NA | NA | 1 | 1 | NA | 0.013453 |
| Diprotodontia | Hypsiprymnodontidae | Hypsiprymnodon | moschatus | Hypsiprymnodon moschatus | 534.25 | 2.04 | 26547.14 | 135.64 | 20.993 | 2 | 4 | NA | NA | 2 | 0.024976 |
| Rodentia | Dipodidae | Sicista | pseudonapaea | Sicista pseudonapaea | NA | NA | 26692.53 | 29.22 | -4.516 | NA | NA | NA | NA | NA | 0.374873 |
| Soricomorpha | Soricidae | Crocidura | glassi | Crocidura glassi | NA | NA | 26724.54 | 64.33 | 10.055 | NA | NA | NA | NA | NA | 0.89448 |
| Rodentia | Echimyidae | Makalata | rhipidura | Makalata rhipidura | 315 | NA | 26835.71 | 232.38 | 26.562 | NA | NA | NA | NA | NA | 0.285285 |
| Rodentia | Echimyidae | Diplomys | caniceps | Diplomys caniceps | 394.49 | NA | 26895.61 | 177.61 | 16.688 | 1 | 2 | 1 | 2 | 1 | 0.257744 |
| Chiroptera | Pteropodidae | Pteropus | temminckii | Pteropus temminckii | NA | NA | 26928.33 | 281 | 23.588 | NA | NA | NA | NA | NA | 0.801881 |
| Primates | Cercopithecidae | Piliocolobus | rufomitratus | Piliocolobus rufomitratus | 8030.75 | 1.01 | 26952.75 | 61.11 | 26.782 | 3 | 3 | 1 | 2 | 1 | 0.016664 |
| Rodentia | Muridae | Apomys | microdon | Apomys microdon | 34.98 | NA | 26990.03 | 254.07 | 25.619 | 2 | 4 | 2 | 2 | 2 | 0.013222 |
| Soricomorpha | Soricidae | Cryptotis | meridensis | Cryptotis meridensis | 12.63 | NA | 27050.11 | 104.67 | 20.991 | NA | 1 | 2 | 1 | 3 | 0.022837 |
| Carnivora | Felidae | Lynx | pardinus | Lynx pardinus | 11050 | 2.5 | 27058.5 | 48.41 | 13.369 | NA | 1 | 1 | 1 | 3 | 1.465674 |
| Rodentia | Sciuridae | Sciurus | richmondi | Sciurus richmondi | 237.51 | 2.59 | 27219.65 | 202.67 | 22.516 | 3 | NA | 2 | 2 | NA | 0.104116 |
| Primates | Cebidae | Callithrix | flaviceps | Callithrix flaviceps | 372.97 | 2 | 27330.89 | 111.78 | 20.081 | 3 | 3 | 1 | 2 | 2 | 0.773673 |
| Chiroptera | Vespertilionidae | Myotis | planiceps | Myotis planiceps | NA | NA | 27602.56 | 20.79 | 15.937 | NA | NA | 1 | 2 | NA | 0.822331 |
| Rodentia | Muridae | Thallomys | shortridgei | Thallomys shortridgei | NA | NA | 27645.77 | 10.7 | 20.167 | NA | NA | NA | NA | NA | 0.290273 |
| Rodentia | Muridae | Grammomys | aridulus | Grammomys aridulus | NA | NA | 27654.16 | 43.54 | 22.221 | NA | NA | NA | NA | NA | 0.324293 |
| Dasyuromorphia | Dasyuridae | Micromurexia | habbema | Micromurexia habbema | NA | NA | 27671.72 | 334.09 | 15.886 | 1 | NA | 2 | 1 | NA | 0.014614 |
| Chiroptera | Rhinolophidae | Rhinolophus | ruwenzorii | Rhinolophus ruwenzorii | NA | NA | 27684.67 | 107.55 | 19.955 | NA | NA | NA | NA | NA | 0.470404 |
| Chiroptera | Vespertilionidae | Hypsugo | kitcheneri | Hypsugo kitcheneri | NA | NA | 27759.22 | 209.66 | 25.483 | NA | NA | 1 | 2 | NA | 0.289402 |
| Artiodactyla | Suidae | Sus | cebifrons | Sus cebifrons | 190792.3 | 4.58 | 27761.56 | 160.41 | 25.491 | 2 | 6 | NA | NA | 2 | 1.759141 |
| Lagomorpha | Ochotonidae | Ochotona | muliensis | Ochotona muliensis | NA | NA | 27843.24 | 113.25 | 12.488 | NA | NA | NA | NA | NA | 0.262474 |
| Carnivora | Viverridae | Chrotogale | owstoni | Chrotogale owstoni | 3267.71 | NA | 27879.9 | 136.85 | 18.306 | 2 | 6 | 1 | 1 | 2 | 1.031593 |
| Diprotodontia | Potoroidae | Bettongia | gaimardi | Bettongia gaimardi | 1667.55 | 1 | 27971.29 | 66.21 | 10.348 | 1 | NA | NA | NA | NA | 0.173995 |
| Rodentia | Muridae | Gerbillus | famulus | Gerbillus famulus | NA | NA | 27974.75 | 5.74 | 18.602 | NA | NA | NA | NA | NA | 0.022232 |
| Primates | Cercopithecidae | Piliocolobus | gordonorum | Piliocolobus gordonorum | NA | NA | 28144.75 | 73.76 | 23.403 | NA | NA | NA | NA | NA | 1.172315 |
| Chiroptera | Pteropodidae | Pteropus | admiralitatum | Pteropus admiralitatum | 306.46 | 1 | 28281.49 | 189.91 | 25.551 | NA | NA | 1 | 2 | NA | 0.018381 |
| Rodentia | Cricetidae | Phyllotis | haggardi | Phyllotis haggardi | 42.5 | NA | 28320.27 | 147.46 | 15.394 | NA | NA | NA | NA | NA | 0.016023 |
| Rodentia | Cricetidae | Thomasomys | paramorum | Thomasomys paramorum | 77 | NA | 28336.34 | 173.76 | 13.879 | NA | NA | NA | NA | NA | 0.014369 |
| Soricomorpha | Soricidae | Crocidura | ultima | Crocidura ultima | 16 | NA | 28532.21 | 77.93 | 16.156 | NA | NA | NA | NA | NA | 0.286234 |
| Diprotodontia | Vombatidae | Lasiorhinus | krefftii | Lasiorhinus krefftii | 31849.99 | 1 | 28559.17 | 49.95 | 21.197 | 1 | 3 | 2 | 1 | 1 | 2.174837 |
| Diprotodontia | Macropodidae | Thylogale | brunii | Thylogale brunii | NA | 1 | 28572.95 | 195.33 | 26.163 | NA | NA | 1 | 1 | NA | 0.681258 |
| Peramelemorphia | Peramelidae | Peroryctes | broadbenti | Peroryctes broadbenti | 4800 | 1.76 | 28581.77 | 183.09 | 21.722 | 1 | 3 | 1 | 1 | 2 | 1.354997 |
| Rodentia | Cricetidae | Xenomys | nelsoni | Xenomys nelsoni | 129.99 | 1.6 | 28727.89 | 79.07 | 22.262 | NA | NA | 2 | 2 | NA | 1.479141 |
| Soricomorpha | Soricidae | Crocidura | tenuis | Crocidura tenuis | NA | NA | 28767.92 | 111.37 | 24.644 | NA | NA | NA | NA | NA | 0.286234 |
| Chiroptera | Hipposideridae | Hipposideros | doriae | Hipposideros doriae | 4.24 | NA | 28807.06 | 295.21 | 22.926 | NA | 1 | 1 | 2 | 3 | 0.244824 |
| Rodentia | Muridae | Pseudomys | glaucus | Pseudomys glaucus | NA | NA | 28931.98 | 93.93 | 26.889 | NA | NA | NA | NA | NA | 2.164946 |
| Soricomorpha | Soricidae | Crocidura | elgonius | Crocidura elgonius | 6.5 | NA | 28984.98 | 86.44 | 21.264 | NA | NA | NA | NA | NA | 0.01789 |
| Rodentia | Nesomyidae | Eliurus | tanala | Eliurus tanala | 89.99 | NA | 29102.38 | 191.57 | 19.196 | NA | NA | NA | NA | NA | 0.017257 |
| Rodentia | Calomyscidae | Calomyscus | baluchi | Calomyscus baluchi | NA | NA | 29169.13 | 31.18 | 6.353 | NA | NA | NA | NA | NA | 0.019168 |
| Rodentia | Cricetidae | Thomasomys | rhoadsi | Thomasomys rhoadsi | 77 | NA | 29270.61 | 186.3 | 12.857 | NA | NA | NA | NA | NA | 0.014369 |
| Primates | Indriidae | Propithecus | deckenii | Propithecus deckenii | NA | NA | 29372.01 | 111.93 | 24.474 | NA | NA | NA | NA | NA | 1.393428 |
| Rodentia | Muridae | Rattus | novaeguineae | Rattus novaeguineae | 133 | 5.37 | 29467.48 | 319.33 | 17.593 | NA | NA | 1 | 1 | NA | 0.016589 |
| Rodentia | Sciuridae | Funambulus | layardi | Funambulus layardi | NA | 2.43 | 29545.66 | 154.53 | 25.761 | NA | NA | NA | NA | NA | 0.864651 |
| Rodentia | Sciuridae | Glyphotes | simus | Glyphotes simus | NA | NA | 29554.63 | 281.47 | 22.508 | NA | NA | NA | NA | NA | 0.329083 |
| Primates | Cercopithecidae | Rhinopithecus | avunculus | Rhinopithecus avunculus | 9086.19 | 1 | 29614.99 | 131.32 | 19.118 | 3 | NA | 2 | 2 | NA | 1.234406 |
| Rodentia | Cricetidae | Anotomys | leander | Anotomys leander | 66.39 | 0.97 | 29663.44 | 166.83 | 14.185 | NA | NA | 1 | NA | NA | 0.96383 |
| Rodentia | Nesomyidae | Gymnuromys | roberti | Gymnuromys roberti | 97.5 | 1.94 | 29664.32 | 178.18 | 18.809 | NA | 2 | NA | NA | 1 | 0.020727 |
| Chiroptera | Pteropodidae | Pteropus | chrysoproctus | Pteropus chrysoproctus | 730.19 | NA | 29871.72 | 280.9 | 23.72 | NA | NA | 1 | 2 | NA | 0.239394 |
| Rodentia | Cricetidae | Neotoma | chrysomelas | Neotoma chrysomelas | NA | 0.97 | 29901.48 | 122.37 | 20.073 | NA | NA | NA | NA | NA | 0.015637 |
| Rodentia | Sciuridae | Petaurista | nobilis | Petaurista nobilis | NA | NA | 30022.25 | 181.56 | 11.927 | NA | NA | NA | NA | NA | 0.66124 |
| Primates | Cercopithecidae | Cercopithecus | sclateri | Cercopithecus sclateri | 3067.29 | 1.02 | 30025.23 | 193.22 | 26.033 | 3 | NA | NA | NA | NA | 0.81357 |
| Rodentia | Muridae | Phloeomys | cumingi | Phloeomys cumingi | 1842.46 | 1 | 30046.13 | 254.07 | 25.619 | 1 | NA | 1 | 2 | NA | 1.007063 |
| Rodentia | Geomyidae | Orthogeomys | dariensis | Orthogeomys dariensis | 437.99 | NA | 30081.13 | 207.7 | 25.2 | NA | 3 | 2 | 1 | 1 | 0.015066 |
| Artiodactyla | Bovidae | Beatragus | hunteri | Beatragus hunteri | 79132.17 | 1 | 30188.68 | 42.28 | 27.223 | NA | 1 | 1 | 1 | 1 | 1.808498 |
| Rodentia | Sciuridae | Callosciurus | adamsi | Callosciurus adamsi | 208.72 | 1.94 | 30384.46 | 287.63 | 23.552 | 3 | 6 | 1 | 2 | 2 | 1.001092 |
| Artiodactyla | Cervidae | Mazama | pandora | Mazama pandora | NA | NA | 30465.15 | 75.83 | 25.527 | NA | NA | NA | NA | NA | 0.381647 |
| Artiodactyla | Bovidae | Madoqua | piacentinii | Madoqua piacentinii | 2500 | 1 | 30486.05 | 18.05 | 26.491 | 3 | 2 | NA | NA | 1 | 0.34834 |
| Rodentia | Ctenomyidae | Ctenomys | pontifex | Ctenomys pontifex | 400 | NA | 30540.4 | 25.84 | 9.521 | NA | 3 | 1 | 1 | 1 | 0.270692 |
| Primates | Cercopithecidae | Trachypithecus | hatinhensis | Trachypithecus hatinhensis | NA | NA | 30579.77 | 171.41 | 21.786 | NA | NA | NA | NA | NA | 0.854974 |
| Diprotodontia | Macropodidae | Petrogale | burbidgei | Petrogale burbidgei | 1258 | 1 | 30601.39 | 90.56 | 28.745 | NA | NA | 1 | 1 | NA | 0.119522 |
| Dasyuromorphia | Dasyuridae | Phascolosorex | doriae | Phascolosorex doriae | NA | NA | 30648.71 | 192.74 | 19.693 | 2 | 2 | 1 | 1 | 2 | 0.012732 |
| Dasyuromorphia | Dasyuridae | Neophascogale | lorentzi | Neophascogale lorentzi | 212 | NA | 30928.61 | 230.41 | 19.483 | 2 | NA | 2 | 2 | NA | 0.013756 |
| Chiroptera | Pteropodidae | Pteropus | melanopogon | Pteropus melanopogon | 874.94 | NA | 30972.95 | 269.37 | 23.897 | NA | NA | 1 | 2 | NA | 1.054615 |
| Chiroptera | Emballonuridae | Taphozous | achates | Taphozous achates | NA | NA | 31015.35 | 111.37 | 24.644 | NA | NA | NA | NA | NA | 0.333216 |
| Rodentia | Sciuridae | Spermophilus | adocetus | Spermophilus adocetus | 155.52 | NA | 31030.21 | 95.75 | 22.176 | NA | 2 | 2 | 1 | 1 | 0.010373 |
| Rodentia | Muridae | Pelomys | hopkinsi | Pelomys hopkinsi | NA | NA | 31127.3 | 96.09 | 19.745 | NA | NA | NA | NA | NA | 0.347679 |
| Rodentia | Cricetidae | Eothenomys | custos | Eothenomys custos | NA | NA | 31182.61 | 149.66 | 3.578 | NA | NA | NA | NA | NA | 0.013 |
| Rodentia | Muridae | Maxomys | ochraceiventer | Maxomys ochraceiventer | 158.96 | NA | 31248.43 | 270.46 | 22.144 | NA | NA | NA | NA | NA | 0.343325 |
| Rodentia | Heteromyidae | Microdipodops | pallidus | Microdipodops pallidus | 13.36 | 3.89 | 31277.67 | 18.04 | 8.774 | 1 | 2 | 2 | 1 | 2 | 0.019028 |
| Dasyuromorphia | Dasyuridae | Parantechinus | apicalis | Parantechinus apicalis | 71.31 | 7.5 | 31495.39 | 56.91 | 14.57 | 2 | 2 | 1 | 1 | 2 | 1.198883 |
| Rodentia | Spalacidae | Spalax | uralensis | Spalax uralensis | NA | NA | 31510.94 | 23.23 | 3.962 | NA | NA | NA | NA | NA | 0.190852 |
| Rodentia | Sciuridae | Marmota | menzbieri | Marmota menzbieri | NA | 2.73 | 31538.38 | 18.87 | 1.946 | 3 | 4 | 1 | 1 | 2 | 0.492246 |
| Rodentia | Muridae | Dipodillus | bottai | Dipodillus bottai | NA | NA | 31544.35 | 29.59 | 27.93 | NA | NA | NA | NA | NA | 0.35571 |
| Rodentia | Muridae | Taterillus | lacustris | Taterillus lacustris | NA | NA | 31681.03 | 50.2 | 27.934 | NA | NA | NA | NA | NA | 0.019791 |
| Soricomorpha | Soricidae | Crocidura | lanosa | Crocidura lanosa | NA | 3.29 | 31734.51 | 114.74 | 17.894 | NA | NA | NA | NA | NA | 1.34172 |
| Rodentia | Dasyproctidae | Dasyprocta | guamara | Dasyprocta guamara | 2650.03 | NA | 31799.28 | 125.99 | 24.905 | 3 | 3 | 1 | 1 | 1 | 0.182165 |
| Soricomorpha | Talpidae | Talpa | stankovici | Talpa stankovici | 71.12 | NA | 32002.56 | 68.91 | 8.989 | 2 | 1 | 2 | 1 | 3 | 0.023985 |
| Rodentia | Cricetidae | Oxymycterus | akodontius | Oxymycterus akodontius | 67.99 | NA | 32016.42 | 44.37 | 4.486 | NA | NA | 1 | 1 | NA | 0.233967 |
| Afrosoricida | Chrysochloridae | Amblysomus | septentrionalis | Amblysomus septentrionalis | NA | NA | 32027.78 | 56.76 | 14.736 | NA | NA | NA | NA | NA | 0.232452 |
| Rodentia | Muridae | Xenuromys | barbatus | Xenuromys barbatus | 975.3 | NA | 32191.27 | 266.38 | 23.877 | NA | 4 | 1 | 1 | 2 | 0.022938 |
| Rodentia | Geomyidae | Cratogeomys | tylorhinus | Cratogeomys tylorhinus | 403.42 | 1.94 | 32204.11 | 70.59 | 17.874 | NA | 4 | 2 | 1 | 1 | 0.017788 |
| Rodentia | Cricetidae | Rhagomys | rufescens | Rhagomys rufescens | 21.2 | NA | 32243.17 | 141.09 | 19.532 | NA | NA | 1 | 2 | NA | 0.198147 |
| Rodentia | Erethizontidae | Echinoprocta | rufescens | Echinoprocta rufescens | 831.79 | NA | 32259.39 | 207.19 | 20.606 | NA | NA | NA | NA | NA | 0.019305 |
| Soricomorpha | Soricidae | Crocidura | desperata | Crocidura desperata | NA | NA | 32266.19 | 82.32 | 20.239 | NA | NA | NA | NA | NA | 1.34172 |
| Rodentia | Cricetidae | Akodon | siberiae | Akodon siberiae | 34.6 | NA | 32319.18 | 70.95 | 17.372 | NA | NA | NA | NA | NA | 0.132909 |
| Rodentia | Octodontidae | Octodon | lunatus | Octodon lunatus | 200.55 | NA | 32534.97 | 35.27 | 11.62 | NA | 2 | NA | NA | 1 | 1.037517 |
| Chiroptera | Hipposideridae | Hipposideros | marisae | Hipposideros marisae | NA | NA | 32672.05 | 172.74 | 23.973 | NA | NA | 1 | 2 | NA | 1.224118 |
| Artiodactyla | Bovidae | Capra | caucasica | Capra caucasica | 58149.52 | 1.09 | 32781.87 | 57.31 | -0.989 | NA | NA | NA | NA | NA | 1.15736 |
| Soricomorpha | Soricidae | Cryptotis | thomasi | Cryptotis thomasi | 11.77 | NA | 32860.44 | 101.78 | 19.096 | NA | 1 | NA | NA | 3 | 0.019088 |
| Dasyuromorphia | Dasyuridae | Paramurexia | rothschildi | Paramurexia rothschildi | 53.77 | 3.39 | 32993.43 | 174.74 | 22.684 | 2 | 1 | 3 | 2 | 3 | 0.81744 |
| Rodentia | Muridae | Chiruromys | lamia | Chiruromys lamia | 47.2 | 2.15 | 33089.66 | 180.67 | 21.636 | NA | NA | 2 | 2 | NA | 0.018693 |
| Rodentia | Gliridae | Myomimus | setzeri | Myomimus setzeri | NA | NA | 33100.62 | 29.41 | 14.83 | NA | NA | NA | NA | NA | 0.355238 |
| Rodentia | Heteromyidae | Heteromys | australis | Heteromys australis | 267.49 | 2.75 | 33112.2 | 217.57 | 25.117 | 1 | 2 | 1 | 1 | 1 | 0.015586 |
| Primates | Atelidae | Oreonax | flavicauda | Oreonax flavicauda | 8229.12 | 1 | 33168.59 | 127.89 | 16.483 | 3 | 4 | 2 | 2 | 1 | 1.676799 |
| Rodentia | Muridae | Rattus | xanthurus | Rattus xanthurus | NA | NA | 33179.44 | 228.54 | 21.699 | NA | NA | NA | NA | NA | 0.708382 |
| Rodentia | Cricetidae | Neusticomys | monticolus | Neusticomys monticolus | 39.5 | 1.94 | 33242.18 | 140.46 | 15.828 | NA | NA | 1 | NA | NA | 0.016731 |
| Rodentia | Ctenomyidae | Ctenomys | sylvanus | Ctenomys sylvanus | NA | NA | 33278.04 | 71.94 | 15.337 | NA | NA | NA | NA | NA | 0.252543 |
| Rodentia | Muridae | Tryphomys | adustus | Tryphomys adustus | NA | NA | 33281.15 | 232.43 | 25.14 | NA | NA | NA | NA | NA | 0.31657 |
| Rodentia | Cricetidae | Alticola | albicaudus | Alticola albicaudus | NA | NA | 33357.58 | 8.19 | -4.957 | NA | NA | NA | NA | NA | 0.228334 |
| Primates | Cercopithecidae | Trachypithecus | delacouri | Trachypithecus delacouri | NA | 1.5 | 33489.77 | 131.41 | 21.248 | 3 | 3 | NA | NA | 1 | 1.082966 |
| Chiroptera | Pteropodidae | Nyctimene | vizcaccia | Nyctimene vizcaccia | 41.96 | NA | 33659.02 | 197.47 | 25.393 | NA | NA | 1 | 2 | NA | 0.016172 |
| Rodentia | Bathyergidae | Bathyergus | janetta | Bathyergus janetta | 388.69 | 3.49 | 33849.47 | 7.72 | 16.497 | NA | 4 | 1 | 1 | 1 | 0.017809 |
| Primates | Cebidae | Leontopithecus | chrysomelas | Leontopithecus chrysomelas | 572.8 | 2.04 | 33921.69 | 153.12 | 22.399 | 3 | NA | 1 | 2 | NA | 0.985195 |
| Rodentia | Sciuridae | Lariscus | hosei | Lariscus hosei | 180 | 1.94 | 34178.18 | 276.34 | 22.726 | NA | NA | 1 | 1 | NA | 0.179139 |
| Rodentia | Cricetidae | Thomasomys | hylophilus | Thomasomys hylophilus | 77 | NA | 34302.85 | 112.54 | 18.534 | NA | NA | 1 | 1 | NA | 1.077669 |
| Rodentia | Cricetidae | Peromyscus | polius | Peromyscus polius | 40 | NA | 34323.83 | 29.42 | 13.562 | 1 | NA | NA | NA | NA | 0.178246 |
| Rodentia | Geomyidae | Geomys | personatus | Geomys personatus | 397 | 3.09 | 34379.88 | 48.57 | 21.553 | NA | 3 | 2 | 1 | 1 | 0.014216 |
| Primates | Cercopithecidae | Rhinopithecus | brelichi | Rhinopithecus brelichi | 12267.15 | 1 | 34391.58 | 114.3 | 13.944 | 3 | 2 | 2 | 2 | 1 | 0.963787 |
| Rodentia | Sciuridae | Dremomys | everetti | Dremomys everetti | 129.78 | NA | 34475.51 | 278.96 | 22.472 | NA | 5 | 2 | 2 | 2 | 0.018891 |
| Chiroptera | Pteropodidae | Dobsonia | inermis | Dobsonia inermis | 152.6 | 0.99 | 34503.07 | 192.96 | 26.2 | NA | NA | 1 | 2 | NA | 0.011041 |
| Primates | Cebidae | Callithrix | kuhlii | Callithrix kuhlii | 374.99 | NA | 34583.88 | 149.75 | 22.994 | 3 | 4 | 1 | 2 | 2 | 0.101574 |
| Primates | Cercopithecidae | Piliocolobus | pennantii | Piliocolobus pennantii | 10896 | 1.02 | 34938.83 | 159.81 | 25.435 | 3 | NA | NA | NA | NA | 1.484932 |
| Chiroptera | Emballonuridae | Taphozous | troughtoni | Taphozous troughtoni | NA | NA | 34968.86 | 37.66 | 24.067 | NA | NA | NA | NA | NA | 0.020402 |
| Rodentia | Muridae | Mus | oubanguii | Mus oubanguii | NA | NA | 34990.93 | 134.48 | 24.643 | NA | NA | NA | NA | NA | 0.250107 |
| Primates | Lepilemuridae | Lepilemur | ruficaudatus | Lepilemur ruficaudatus | 763.36 | 1.02 | 35030.08 | 57.89 | 23.861 | 1 | 2 | 1 | 2 | 1 | 0.311582 |
| Rodentia | Sciuridae | Callosciurus | orestes | Callosciurus orestes | 323.91 | 3 | 35107.14 | 281.49 | 22.326 | 3 | 6 | 1 | 2 | 2 | 0.01997 |
| Rodentia | Cricetidae | Abrothrix | illuteus | Abrothrix illuteus | 47.79 | NA | 35359.55 | 57.04 | 4.189 | NA | NA | NA | NA | NA | 0.160319 |
| Chiroptera | Vespertilionidae | Pipistrellus | aero | Pipistrellus aero | NA | NA | 35388.28 | 65.76 | 20.014 | NA | NA | 1 | 2 | NA | 0.296208 |
| Lagomorpha | Ochotonidae | Ochotona | himalayana | Ochotona himalayana | NA | 3.42 | 35498.06 | 102.73 | -8.418 | NA | NA | 1 | 1 | NA | 0.018885 |
| Soricomorpha | Soricidae | Sorex | bairdi | Sorex bairdi | 8.33 | NA | 35561.66 | 99.53 | 7.744 | NA | NA | NA | NA | NA | 0.018367 |
| Rodentia | Muridae | Hydromys | neobritannicus | Hydromys neobritannicus | NA | NA | 35567.2 | 217.69 | 25.385 | NA | NA | NA | NA | NA | 0.285607 |
| Primates | Lemuridae | Eulemur | albifrons | Eulemur albifrons | NA | NA | 35602.37 | 168.69 | 20.158 | NA | NA | NA | NA | NA | 0.860187 |
| Rodentia | Geomyidae | Pappogeomys | bulleri | Pappogeomys bulleri | 150 | 0.97 | 35746.47 | 79.39 | 20.546 | NA | 3 | 2 | 1 | 1 | 0.017788 |
| Artiodactyla | Hippopotamidae | Hexaprotodon | liberiensis | Hexaprotodon liberiensis | 235001.2 | 1 | 35806.59 | 213.58 | 25.156 | 2 | 4 | 2 | 1 | 1 | 1.831511 |
| Diprotodontia | Macropodidae | Dendrolagus | spadix | Dendrolagus spadix | 8468.2 | 1 | 35926.62 | 411.38 | 24.328 | 2 | 2 | 2 | 2 | 1 | 0.013042 |
| Rodentia | Caviidae | Microcavia | shiptoni | Microcavia shiptoni | 185 | 3.16 | 35972.81 | 66.12 | 5.372 | NA | NA | NA | NA | NA | 0.1916 |
| Chiroptera | Phyllostomidae | Sturnira | mordax | Sturnira mordax | 11.79 | 0.98 | 36057.38 | 248.5 | 24.778 | NA | 1 | 1 | 2 | 1 | 0.153911 |
| Soricomorpha | Soricidae | Anourosorex | yamashinai | Anourosorex yamashinai | NA | NA | 36065.98 | 160.5 | 16.58 | NA | NA | NA | NA | NA | 0.020904 |
| Rodentia | Muridae | Apodemus | semotus | Apodemus semotus | 25.77 | 3.56 | 36065.98 | 160.5 | 16.58 | NA | NA | NA | NA | NA | 0.018118 |
| Soricomorpha | Soricidae | Crocidura | tanakae | Crocidura tanakae | NA | NA | 36065.98 | 160.5 | 16.58 | NA | NA | NA | NA | NA | 0.015538 |
| Soricomorpha | Soricidae | Episoriculus | fumidus | Episoriculus fumidus | 6.95 | 3.42 | 36065.98 | 160.5 | 16.58 | NA | NA | NA | NA | NA | 0.018741 |
| Chiroptera | Vespertilionidae | Murina | puta | Murina puta | NA | NA | 36065.98 | 160.5 | 16.58 | NA | NA | 1 | 2 | NA | 0.149844 |
| Rodentia | Muridae | Niviventer | coninga | Niviventer coninga | 80.65 | 2.37 | 36065.98 | 160.5 | 16.58 | NA | NA | NA | NA | NA | 0.021458 |
| Rodentia | Muridae | Niviventer | culturatus | Niviventer culturatus | 81.17 | NA | 36065.98 | 160.5 | 16.58 | NA | NA | NA | NA | NA | 0.214578 |
| Chiroptera | Vespertilionidae | Plecotus | taivanus | Plecotus taivanus | NA | NA | 36065.98 | 160.5 | 16.58 | NA | NA | 1 | 2 | NA | 0.173099 |
| Rodentia | Muridae | Niviventer | brahma | Niviventer brahma | NA | NA | 36091.7 | 183.95 | 5.899 | NA | NA | NA | NA | NA | 0.021458 |
| Carnivora | Ursidae | Ailuropoda | melanoleuca | Ailuropoda melanoleuca | 118000 | 1.62 | 36206.89 | 101.81 | 6.551 | 2 | 3 | 1 | 1 | 1 | 1.907665 |
| Rodentia | Chinchillidae | Chinchilla | lanigera | Chinchilla lanigera | 480.28 | 1.87 | 36255.13 | 11.48 | 7.217 | 2 | 6 | 1 | 1 | 1 | 1.847554 |
| Rodentia | Sciuridae | Prosciurillus | murinus | Prosciurillus murinus | NA | NA | 36268.59 | 228.75 | 19.89 | NA | NA | NA | NA | NA | 0.307928 |
| Rodentia | Muridae | Pseudomys | fumeus | Pseudomys fumeus | 68.75 | NA | 36320.16 | 63.74 | 10.579 | NA | NA | NA | NA | NA | 1.623709 |
| Rodentia | Cricetidae | Microtus | guatemalensis | Microtus guatemalensis | 41.99 | NA | 36364.52 | 96.96 | 19.828 | NA | NA | NA | NA | NA | 0.16239 |
| Artiodactyla | Bovidae | Cephalophus | spadix | Cephalophus spadix | 56857.02 | NA | 36583.37 | 77.3 | 22.271 | 1 | 3 | 1 | 1 | 1 | 0.958734 |
| Primates | Pitheciidae | Callicebus | medemi | Callicebus medemi | NA | NA | 36587.57 | 152.17 | 29.208 | NA | NA | NA | NA | NA | 0.808168 |
| Diprotodontia | Macropodidae | Macropus | bernardus | Macropus bernardus | 17000 | 1 | 36606.69 | 96.41 | 26.21 | NA | NA | 1 | 1 | NA | 0.164319 |
| Rodentia | Muridae | Crateromys | schadenbergi | Crateromys schadenbergi | NA | NA | 36658.29 | 226.03 | 22.683 | 2 | NA | NA | NA | NA | 1.326714 |
| Rodentia | Muridae | Bullimus | luzonicus | Bullimus luzonicus | NA | NA | 36674.54 | 206.13 | 22.305 | NA | NA | NA | NA | NA | 0.017571 |
| Soricomorpha | Soricidae | Cryptotis | alticola | Cryptotis alticola | NA | NA | 36737.67 | 83.11 | 19.104 | NA | NA | NA | NA | NA | 0.290261 |
| Chiroptera | Pteropodidae | Nyctimene | major | Nyctimene major | 107.06 | NA | 36810.07 | 203.55 | 26.024 | NA | NA | 1 | 2 | NA | 0.016212 |
| Chiroptera | Vespertilionidae | Myotis | vivesi | Myotis vivesi | 25.63 | 0.99 | 36822.86 | 13.39 | 19.881 | NA | 1 | 1 | 2 | 3 | 0.810421 |
| Lagomorpha | Leporidae | Bunolagus | monticularis | Bunolagus monticularis | 1750.01 | 1.22 | 37052.01 | 14.27 | 15.615 | 1 | 3 | NA | NA | 1 | 2.20727 |
| Rodentia | Muridae | Myomyscus | yemeni | Myomyscus yemeni | NA | NA | 37193.53 | 17.08 | 17.569 | NA | NA | NA | NA | NA | 0.020329 |
| Rodentia | Nesomyidae | Brachyuromys | betsileoensis | Brachyuromys betsileoensis | 93.58 | NA | 37287.31 | 167.8 | 18.484 | 2 | NA | 1 | 1 | NA | 0.019353 |
| Rodentia | Cricetidae | Oxymycterus | delator | Oxymycterus delator | 81.49 | NA | 37329.03 | 125.73 | 21.727 | NA | NA | NA | NA | NA | 0.014623 |
| Soricomorpha | Soricidae | Crocidura | batesi | Crocidura batesi | 16.49 | NA | 37339.81 | 147.78 | 23.908 | NA | NA | NA | NA | NA | 0.01789 |
| Rodentia | Sciuridae | Tamias | panamintinus | Tamias panamintinus | 51.73 | 4.04 | 37448.25 | 22.24 | 10.896 | 3 | 6 | 2 | 2 | 2 | 0.016803 |
| Chiroptera | Emballonuridae | Peropteryx | trinitatis | Peropteryx trinitatis | 4.19 | NA | 37561.11 | 153.74 | 24.908 | NA | NA | NA | NA | NA | 0.317091 |
| Didelphimorphia | Didelphidae | Monodelphis | maraxina | Monodelphis maraxina | NA | NA | 37711.99 | 217.7 | 26.024 | 1 | 1 | 1 | 1 | 3 | 0.386822 |
| Rodentia | Echimyidae | Callistomys | pictus | Callistomys pictus | 518.99 | NA | 37773.91 | 123.66 | 23.911 | NA | NA | NA | NA | NA | 1.337274 |
| Diprotodontia | Pseudocheiridae | Pseudochirulus | caroli | Pseudochirulus caroli | 456.02 | NA | 38148.52 | 183.77 | 20.936 | NA | 2 | 1 | 2 | 1 | 0.019417 |
| Chiroptera | Molossidae | Mormopterus | doriae | Mormopterus doriae | NA | NA | 38232.28 | 169.22 | 23.694 | NA | 1 | 1 | 2 | 3 | 0.27785 |
| Rodentia | Muridae | Haeromys | pusillus | Haeromys pusillus | NA | NA | 38248.7 | 285.78 | 22.315 | NA | 1 | NA | NA | 1 | 0.886363 |
| Rodentia | Muridae | Praomys | hartwigi | Praomys hartwigi | 38.86 | 3.49 | 38315.64 | 183.69 | 22.826 | NA | 4 | NA | NA | 2 | 1.310497 |
| Lagomorpha | Ochotonidae | Ochotona | koslowi | Ochotona koslowi | NA | 1 | 38543.68 | 20.76 | -9.26 | 3 | NA | 2 | 1 | NA | 1.347695 |
| Chiroptera | Pteropodidae | Rousettus | spinalatus | Rousettus spinalatus | 92.32 | 1 | 38709.46 | 272.7 | 22.668 | NA | NA | 1 | 2 | NA | 0.513763 |
| Primates | Cebidae | Saguinus | leucopus | Saguinus leucopus | 456.68 | 2 | 38875.35 | 143.66 | 23.106 | 3 | 5 | 1 | 2 | 2 | 0.985969 |
| Rodentia | Sciuridae | Sciurus | arizonensis | Sciurus arizonensis | 646.99 | 3.09 | 39352.69 | 33.75 | 14.581 | 3 | 3 | 2 | 2 | 1 | 0.144529 |
| Soricomorpha | Soricidae | Crocidura | congobelgica | Crocidura congobelgica | NA | NA | 39431.51 | 126.69 | 21.108 | NA | NA | NA | NA | NA | 0.01789 |
| Primates | Indriidae | Indri | indri | Indri indri | 8565.48 | 1.01 | 39504.84 | 187.11 | 19.975 | 3 | 4 | 1 | 2 | 1 | 1.843504 |
| Diprotodontia | Phalangeridae | Wyulda | squamicaudata | Wyulda squamicaudata | 1799.21 | 1.01 | 39625.83 | 80.16 | 28.076 | 1 | 1 | 2 | 2 | 1 | 0.321129 |
| Primates | Indriidae | Propithecus | diadema | Propithecus diadema | 6568.99 | 1.01 | 39817.06 | 183.83 | 19.834 | 3 | 3 | 2 | 2 | 1 | 1.765009 |
| Primates | Cercopithecidae | Semnopithecus | schistaceus | Semnopithecus schistaceus | NA | NA | 39835.34 | 161.15 | -2.69 | NA | NA | NA | NA | NA | 0.013347 |
| Chiroptera | Vespertilionidae | Kerivoula | intermedia | Kerivoula intermedia | NA | NA | 39838.82 | 244.12 | 24.047 | NA | NA | 1 | 2 | NA | 0.15685 |
| Soricomorpha | Soricidae | Crocidura | ansellorum | Crocidura ansellorum | NA | NA | 39928.17 | 104.85 | 20.658 | NA | NA | NA | NA | NA | 1.34172 |
| Rodentia | Muridae | Apomys | abrae | Apomys abrae | 34.88 | NA | 39988.41 | 224.37 | 23.144 | 1 | NA | 2 | 2 | NA | 0.211549 |
| Chiroptera | Phyllostomidae | Platyrrhinus | chocoensis | Platyrrhinus chocoensis | NA | NA | 40113.01 | 361.44 | 20.791 | NA | NA | 1 | 2 | NA | 1.204279 |
| Soricomorpha | Soricidae | Suncus | montanus | Suncus montanus | NA | NA | 40185.65 | 148.78 | 25.781 | NA | NA | NA | NA | NA | 0.853081 |
| Rodentia | Cricetidae | Peromyscus | gymnotis | Peromyscus gymnotis | 40 | NA | 40365.9 | 152.62 | 23.862 | 1 | NA | NA | NA | NA | 0.017825 |
| Soricomorpha | Soricidae | Sylvisorex | oriundus | Sylvisorex oriundus | NA | NA | 40446.36 | 139.82 | 22.93 | NA | NA | NA | NA | NA | 0.324743 |
| Rodentia | Cricetidae | Thomasomys | kalinowskii | Thomasomys kalinowskii | 77 | NA | 40500.76 | 125.83 | 14.295 | NA | NA | NA | NA | NA | 0.718446 |
| Rodentia | Cricetidae | Euneomys | mordax | Euneomys mordax | 81.99 | NA | 40516.08 | 34.21 | 0.824 | NA | NA | 2 | 1 | NA | 0.015865 |
| Artiodactyla | Bovidae | Procapra | przewalskii | Procapra przewalskii | 27499.99 | NA | 40688.43 | 27.29 | -2.413 | NA | NA | NA | NA | NA | 1.308517 |
| Afrosoricida | Chrysochloridae | Carpitalpa | arendsi | Carpitalpa arendsi | 52.34 | NA | 40698.67 | 95.34 | 21.827 | NA | 1 | 1 | 1 | 3 | 1.202605 |
| Primates | Cheirogaleidae | Cheirogaleus | adipicaudatus | Cheirogaleus adipicaudatus | NA | NA | 40707.46 | 73.64 | 22.03 | NA | NA | NA | NA | NA | 0.348559 |
| Chiroptera | Vespertilionidae | Falsistrellus | mackenziei | Falsistrellus mackenziei | 23 | NA | 40804.15 | 64.03 | 15.35 | NA | NA | NA | NA | NA | 0.174357 |
| Rodentia | Cricetidae | Oecomys | flavicans | Oecomys flavicans | 73.4 | NA | 40871.5 | 99 | 23.369 | NA | NA | NA | NA | NA | 0.012792 |
| Artiodactyla | Bovidae | Bubalus | depressicornis | Bubalus depressicornis | 256989.3 | 1 | 41026.7 | 227.75 | 19.783 | 3 | 3 | 1 | 1 | 1 | 1.109674 |
| Chiroptera | Hipposideridae | Hipposideros | sumbae | Hipposideros sumbae | NA | NA | 41062.47 | 254.68 | 23.977 | NA | NA | NA | NA | NA | 0.020385 |
| Soricomorpha | Soricidae | Sorex | buchariensis | Sorex buchariensis | NA | NA | 41192.77 | 11.17 | -9.799 | NA | NA | NA | NA | NA | 0.018346 |
| Diprotodontia | Pseudocheiridae | Pseudochirulus | forbesi | Pseudochirulus forbesi | 639.16 | 1.26 | 41306.37 | 197.82 | 21.095 | NA | 2 | 1 | 2 | 1 | 0.01893 |
| Rodentia | Cricetidae | Akodon | torques | Akodon torques | 38.99 | NA | 41379.72 | 199.78 | 12.362 | NA | NA | NA | NA | NA | 0.013492 |
| Soricomorpha | Talpidae | Talpa | caucasica | Talpa caucasica | NA | NA | 41455.61 | 47.08 | 2.935 | 2 | 1 | 2 | 1 | 3 | 0.023985 |
| Primates | Lepilemuridae | Lepilemur | mustelinus | Lepilemur mustelinus | 669.03 | 1 | 41461.51 | 173.61 | 19.875 | 1 | 3 | 1 | 2 | 1 | 0.311582 |
| Carnivora | Eupleridae | Salanoia | concolor | Salanoia concolor | 711.49 | NA | 41471.69 | 197.43 | 20.52 | 3 | 3 | 1 | 1 | 2 | 1.073209 |
| Rodentia | Cricetidae | Ichthyomys | stolzmanni | Ichthyomys stolzmanni | 84.7 | NA | 41721.31 | 43.57 | 16.132 | 1 | 2 | 2 | 1 | 3 | 0.262459 |
| Rodentia | Muridae | Apodemus | alpicola | Apodemus alpicola | NA | NA | 41907.92 | 87.45 | 1.831 | NA | NA | NA | NA | NA | 0.014234 |
| Artiodactyla | Bovidae | Tragelaphus | buxtoni | Tragelaphus buxtoni | 215000.8 | 1 | 41913.3 | 65.43 | 10.912 | 2 | 1 | 1 | 1 | 1 | 1.530041 |
| Primates | Cercopithecidae | Presbytis | comata | Presbytis comata | 6550.07 | 1.01 | 42000.55 | 217.85 | 24.413 | 3 | 3 | 1 | 2 | 1 | 0.880777 |
| Rodentia | Muridae | Lemniscomys | linulus | Lemniscomys linulus | NA | NA | 42103.2 | 110.95 | 26.674 | NA | NA | NA | NA | NA | 0.019499 |
| Rodentia | Cricetidae | Habromys | lophurus | Habromys lophurus | 40 | NA | 42237.85 | 105.26 | 20.527 | NA | NA | NA | NA | NA | 0.180931 |
| Rodentia | Muridae | Pseudomys | apodemoides | Pseudomys apodemoides | 23.78 | 4.9 | 42383.45 | 36.7 | 14.735 | NA | NA | NA | NA | NA | 0.021649 |
| Rodentia | Muridae | Pseudomys | higginsi | Pseudomys higginsi | 63.16 | 2.62 | 42390.86 | 71.57 | 10.251 | 1 | 3 | 1 | 1 | 2 | 0.021649 |
| Primates | Cheirogaleidae | Allocebus | trichotis | Allocebus trichotis | 78.09 | 1 | 42488.06 | 190.23 | 20.317 | 2 | 4 | 1 | 2 | 2 | 0.368008 |
| Rodentia | Cricetidae | Neotoma | phenax | Neotoma phenax | 227.49 | 2 | 42812.73 | 26.35 | 23.877 | NA | 2 | NA | NA | 1 | 0.197929 |
| Rodentia | Cricetidae | Lenoxus | apicalis | Lenoxus apicalis | 53.6 | NA | 43008.18 | 247.68 | 8.929 | NA | NA | NA | NA | NA | 0.019815 |
| Rodentia | Cricetidae | Abrothrix | lanosus | Abrothrix lanosus | 27.83 | NA | 43016.67 | 60.63 | 4.534 | NA | NA | NA | NA | NA | 0.012978 |
| Primates | Cheirogaleidae | Phaner | pallescens | Phaner pallescens | NA | NA | 43081.65 | 83.91 | 24.448 | NA | NA | NA | NA | NA | 0.02286 |
| Rodentia | Cricetidae | Rhipidomys | fulviventer | Rhipidomys fulviventer | 89 | NA | 43102.02 | 101.01 | 18.441 | 1 | NA | 1 | 2 | NA | 0.015785 |
| Chiroptera | Emballonuridae | Saccolaimus | mixtus | Saccolaimus mixtus | NA | NA | 43137.49 | 137.09 | 25.281 | 1 | NA | 1 | 2 | NA | 0.34063 |
| Chiroptera | Emballonuridae | Taphozous | hildegardeae | Taphozous hildegardeae | 29.37 | 1 | 43393.84 | 98.46 | 25.272 | 1 | 1 | 1 | 2 | 3 | 1.147692 |
| Rodentia | Cricetidae | Peromyscus | megalops | Peromyscus megalops | 66.2 | 2 | 43655.36 | 100.12 | 19.206 | 1 | NA | NA | NA | NA | 0.017825 |
| Rodentia | Spalacidae | Spalax | graecus | Spalax graecus | NA | NA | 43748.63 | 49.65 | 7.839 | NA | NA | NA | NA | NA | 0.209852 |
| Afrosoricida | Chrysochloridae | Chrysospalax | trevelyani | Chrysospalax trevelyani | 434.04 | 1.41 | 43844.11 | 63.73 | 15.604 | 2 | 2 | 2 | 1 | 3 | 1.660658 |
| Chiroptera | Molossidae | Otomops | secundus | Otomops secundus | NA | NA | 43995 | 272.12 | 18.799 | NA | NA | 1 | 2 | NA | 0.294193 |
| Rodentia | Cricetidae | Thomasomys | ischyrus | Thomasomys ischyrus | 77 | NA | 44262.39 | 112.39 | 16.208 | NA | NA | NA | NA | NA | 0.718446 |
| Artiodactyla | Bovidae | Bos | taurus | Bos taurus | 618642.4 | NA | 44447.62 | 174.94 | 22.873 | NA | NA | NA | NA | NA | 1.437927 |
| Lagomorpha | Leporidae | Caprolagus | hispidus | Caprolagus hispidus | 2496.99 | 3.46 | 44515.42 | 128.92 | 23.621 | 1 | 3 | 2 | 1 | 1 | 1.460899 |
| Diprotodontia | Macropodidae | Setonix | brachyurus | Setonix brachyurus | 3027.67 | 1 | 44560.4 | 67.08 | 14.861 | 1 | 2 | 1 | 1 | 1 | 0.874415 |
| Rodentia | Muridae | Melomys | capensis | Melomys capensis | 70 | NA | 44665.92 | 119.04 | 26.784 | NA | NA | NA | NA | NA | 0.020235 |
| Rodentia | Muridae | Lophuromys | medicaudatus | Lophuromys medicaudatus | NA | NA | 44946.37 | 100.43 | 18.572 | NA | NA | NA | NA | NA | 0.739171 |
| Diprotodontia | Macropodidae | Dendrolagus | stellarum | Dendrolagus stellarum | NA | NA | 45017.53 | 205.37 | 19.923 | NA | NA | NA | NA | NA | 0.651652 |
| Rodentia | Echimyidae | Olallamys | albicauda | Olallamys albicauda | 273.5 | NA | 45105.03 | 122.16 | 20.986 | NA | NA | NA | NA | NA | 0.255254 |
| Chiroptera | Pteropodidae | Dobsonia | moluccensis | Dobsonia moluccensis | 447.64 | 0.99 | 45157.74 | 236.8 | 24.3 | NA | 1 | 1 | 2 | 1 | 0.011125 |
| Chiroptera | Pteropodidae | Melonycteris | melanops | Melonycteris melanops | 47.59 | NA | 45403.57 | 214.16 | 25.186 | NA | 1 | 1 | 2 | 1 | 0.019211 |
| Rodentia | Muridae | Pseudomys | shortridgei | Pseudomys shortridgei | 72.06 | 3 | 45747.71 | 50.51 | 14.443 | NA | NA | NA | NA | NA | 0.216495 |
| Rodentia | Sciuridae | Tamias | sonomae | Tamias sonomae | 75 | 4 | 45834.06 | 59.13 | 9.656 | NA | 2 | 2 | 2 | 1 | 0.013003 |
| Didelphimorphia | Didelphidae | Monodelphis | osgoodi | Monodelphis osgoodi | 112 | NA | 45995.47 | 65.93 | 16.727 | 1 | 1 | 1 | 1 | 3 | 0.024176 |
| Soricomorpha | Soricidae | Crocidura | mutesae | Crocidura mutesae | 23.91 | NA | 46052.61 | 100.92 | 22.918 | NA | NA | NA | NA | NA | 0.286234 |
| Rodentia | Cricetidae | Oligoryzomys | magellanicus | Oligoryzomys magellanicus | 25.2 | NA | 46140.03 | 33.28 | 4.103 | NA | NA | NA | NA | NA | 0.013332 |
| Rodentia | Cricetidae | Nelsonia | neotomodon | Nelsonia neotomodon | 80 | NA | 46169.86 | 54.62 | 15.267 | NA | NA | NA | NA | NA | 0.17751 |
| Rodentia | Ctenomyidae | Ctenomys | porteousi | Ctenomys porteousi | 192.4 | NA | 46290.24 | 62.88 | 14.579 | NA | 3 | 1 | 1 | 1 | 0.169183 |
| Rodentia | Ctenomyidae | Ctenomys | pearsoni | Ctenomys pearsoni | 212 | NA | 46304.08 | 84.6 | 15.925 | NA | 3 | 1 | 1 | 1 | 0.169183 |
| Rodentia | Muridae | Gerbillurus | tytonis | Gerbillurus tytonis | 29.79 | 4.4 | 46333.88 | 2.17 | 12.519 | NA | NA | 2 | 1 | NA | 0.017972 |
| Rodentia | Bathyergidae | Cryptomys | foxi | Cryptomys foxi | NA | NA | 46366.94 | 110.11 | 25.576 | NA | NA | NA | NA | NA | 0.312923 |
| Rodentia | Muridae | Niviventer | fraternus | Niviventer fraternus | NA | NA | 46452.46 | 212.15 | 24.459 | NA | NA | NA | NA | NA | 0.017973 |
| Chiroptera | Rhinolophidae | Rhinolophus | robinsoni | Rhinolophus robinsoni | 8.54 | NA | 46677.33 | 189.82 | 25.319 | NA | 1 | 1 | 2 | 3 | 0.122072 |
| Rodentia | Cricetidae | Microtus | quasiater | Microtus quasiater | 40 | 1.4 | 47131.29 | 102.42 | 20.285 | NA | NA | NA | NA | NA | 0.144656 |
| Afrosoricida | Chrysochloridae | Amblysomus | corriae | Amblysomus corriae | NA | NA | 47134.97 | 34.4 | 15.112 | NA | NA | NA | NA | NA | 0.232452 |
| Rodentia | Muridae | Crossomys | moncktoni | Crossomys moncktoni | 168.42 | 1.07 | 47340.55 | 370.04 | 16.751 | 3 | 2 | 3 | 1 | 3 | 0.022938 |
| Primates | Cebidae | Saguinus | melanoleucus | Saguinus melanoleucus | NA | NA | 47371.93 | 183.93 | 24.781 | NA | NA | NA | NA | NA | 0.013493 |
| Rodentia | Sciuridae | Sciurus | flammifer | Sciurus flammifer | NA | NA | 47391.27 | 107.8 | 23.347 | 3 | NA | 2 | 2 | NA | 0.17047 |
| Chiroptera | Mystacinidae | Mystacina | tuberculata | Mystacina tuberculata | 13.14 | 0.99 | 47521.83 | 86.52 | 10.713 | 1 | 4 | 2 | 2 | 2 | 1.396215 |
| Artiodactyla | Suidae | Babyrousa | celebensis | Babyrousa celebensis | NA | NA | 47657.62 | 231.15 | 20.652 | NA | NA | NA | NA | NA | 1.070056 |
| Rodentia | Cricetidae | Neotomodon | alstoni | Neotomodon alstoni | 44.64 | 3.2 | 47683.5 | 90.97 | 17.271 | 1 | NA | 2 | 1 | NA | 0.0189 |
| Rodentia | Muridae | Coccymys | ruemmleri | Coccymys ruemmleri | 29.7 | NA | 47734.24 | 308.34 | 16.687 | NA | 2 | 2 | 2 | 1 | 0.019459 |
| Primates | Cheirogaleidae | Cheirogaleus | crossleyi | Cheirogaleus crossleyi | NA | NA | 47826.13 | 180.87 | 18.764 | NA | NA | NA | NA | NA | 0.348559 |
| Rodentia | Muridae | Hydromys | shawmayeri | Hydromys shawmayeri | NA | NA | 47925.94 | 289.97 | 18.272 | NA | 2 | 3 | 1 | 3 | 0.01785 |
| Soricomorpha | Soricidae | Crocidura | greenwoodi | Crocidura greenwoodi | NA | NA | 47947.78 | 40.57 | 26.921 | NA | NA | NA | NA | NA | 0.01789 |
| Rodentia | Cricetidae | Oryzomys | balneator | Oryzomys balneator | 60.5 | NA | 48034.34 | 158.46 | 15.944 | NA | NA | NA | NA | NA | 0.243516 |
| Rodentia | Sciuridae | Tamias | durangae | Tamias durangae | 84.99 | 2.38 | 48090.07 | 44.34 | 12.972 | NA | NA | NA | NA | NA | 0.015355 |
| Rodentia | Echimyidae | Diplomys | rufodorsalis | Diplomys rufodorsalis | 144.8 | NA | 48212.86 | 102.76 | 23.856 | 1 | 2 | 1 | 2 | 1 | 0.257744 |
| Rodentia | Cricetidae | Reithrodontomys | darienensis | Reithrodontomys darienensis | 12.99 | 3.88 | 48257.72 | 188.64 | 25.268 | 1 | NA | 2 | 2 | NA | 0.018435 |
| Rodentia | Cricetidae | Oryzomys | rhabdops | Oryzomys rhabdops | NA | NA | 48320.79 | 113.17 | 20.998 | NA | NA | NA | NA | NA | 0.760988 |
| Rodentia | Cricetidae | Akodon | sylvanus | Akodon sylvanus | 38.99 | NA | 48393.63 | 68.04 | 12.057 | NA | NA | NA | NA | NA | 0.011161 |
| Chiroptera | Phyllostomidae | Artibeus | inopinatus | Artibeus inopinatus | NA | NA | 48689.76 | 134.62 | 23.002 | NA | 1 | 1 | 2 | 1 | 0.183995 |
| Rodentia | Sciuridae | Spermophilus | madrensis | Spermophilus madrensis | 207.12 | 4.34 | 48758.56 | 34.24 | 11.911 | NA | NA | NA | NA | NA | 0.104477 |
| Afrosoricida | Tenrecidae | Oryzorictes | hova | Oryzorictes hova | 35.74 | NA | 48829.19 | 196.86 | 20.402 | 1 | NA | 2 | 1 | NA | 0.024329 |
| Chiroptera | Hipposideridae | Hipposideros | inornatus | Hipposideros inornatus | 25.9 | NA | 48918.55 | 106.09 | 27.001 | NA | NA | NA | NA | NA | 1.019271 |
| Rodentia | Heteromyidae | Dipodomys | compactus | Dipodomys compactus | 49.25 | 1.94 | 48975.08 | 49.53 | 21.794 | 1 | NA | NA | NA | NA | 0.015401 |
| Peramelemorphia | Peramelidae | Perameles | gunnii | Perameles gunnii | 903.46 | 2.31 | 49082.44 | 62.9 | 11.438 | 2 | 7 | 2 | 1 | 2 | 0.187706 |
| Rodentia | Sciuridae | Sciurus | alleni | Sciurus alleni | 465.14 | 3 | 49149.19 | 38.04 | 20.8 | 3 | 3 | 2 | 2 | 2 | 0.010282 |
| Soricomorpha | Solenodontidae | Solenodon | cubanus | Solenodon cubanus | 824.62 | 1.5 | 49168.47 | 95.04 | 25.076 | 2 | 2 | 3 | 2 | 3 | 2.149992 |
| Rodentia | Sciuridae | Petinomys | fuscocapillus | Petinomys fuscocapillus | 794.5 | 2 | 49209.63 | 156.65 | 25.904 | NA | NA | NA | NA | NA | 0.129699 |
| Rodentia | Muridae | Chiruromys | forbesi | Chiruromys forbesi | 105.15 | 2.68 | 49211.92 | 197.79 | 21.428 | NA | NA | 2 | 2 | NA | 0.018693 |
| Rodentia | Muridae | Pseudohydromys | ellermani | Pseudohydromys ellermani | 21.4 | NA | 49257.1 | 347.67 | 15.295 | NA | 3 | 2 | 1 | 2 | 0.017222 |
| Rodentia | Sciuridae | Petaurista | magnificus | Petaurista magnificus | 1475.49 | 0.97 | 49308.69 | 203.15 | 2.743 | NA | NA | NA | NA | NA | 0.140023 |
| Primates | Cercopithecidae | Presbytis | thomasi | Presbytis thomasi | 6689.99 | NA | 49763.07 | 145.84 | 25.053 | 3 | NA | NA | NA | NA | 0.587185 |
| Rodentia | Muridae | Bunomys | penitus | Bunomys penitus | NA | NA | 49798.01 | 227.45 | 20.109 | NA | NA | NA | NA | NA | 0.195615 |
| Chiroptera | Vespertilionidae | Laephotis | namibensis | Laephotis namibensis | NA | NA | 49895.04 | 4.11 | 12.346 | NA | NA | 1 | 2 | NA | 0.018927 |
| Rodentia | Cricetidae | Thomasomys | baeops | Thomasomys baeops | 77 | NA | 49972.03 | 120.89 | 18.409 | NA | NA | NA | NA | NA | 0.014369 |
| Primates | Cebidae | Leontopithecus | chrysopygus | Leontopithecus chrysopygus | 656.12 | NA | 50004.81 | 127.26 | 21.864 | 3 | 4 | 1 | 2 | 2 | 0.970669 |
| Rodentia | Nesomyidae | Delanymys | brooksi | Delanymys brooksi | 4.99 | 3.19 | 50029.97 | 113.18 | 19.249 | NA | NA | NA | NA | NA | 1.076373 |
| Rodentia | Nesomyidae | Dendromus | nyasae | Dendromus nyasae | NA | NA | 50091.76 | 112.07 | 19.125 | NA | NA | NA | NA | NA | 0.022547 |
| Soricomorpha | Soricidae | Crocidura | niobe | Crocidura niobe | 16 | 2.99 | 50103.11 | 106.65 | 19.992 | NA | NA | NA | NA | NA | 0.178896 |
| Rodentia | Sciuridae | Spermophilus | annulatus | Spermophilus annulatus | 499.99 | 3.88 | 50385.66 | 91.12 | 22.028 | NA | 3 | 3 | 2 | 2 | 0.014174 |
| Chiroptera | Molossidae | Mormopterus | phrudus | Mormopterus phrudus | NA | NA | 50558.21 | 106.09 | 21.295 | NA | NA | 1 | 2 | NA | 0.868281 |
| Rodentia | Nesomyidae | Petromyscus | monticularis | Petromyscus monticularis | 20.72 | NA | 50605.31 | 9.28 | 19.903 | NA | NA | NA | NA | NA | 0.020919 |
| Perissodactyla | Rhinocerotidae | Rhinoceros | sondaicus | Rhinoceros sondaicus | 1750000 | 1 | 50679.79 | 167.05 | 24.458 | NA | 2 | 1 | 1 | 1 | 2.183036 |
| Chiroptera | Nycteridae | Nycteris | madagascariensis | Nycteris madagascariensis | 17.69 | NA | 50763.9 | 137.4 | 21.765 | NA | 1 | NA | NA | 3 | 0.28518 |
| Primates | Cebidae | Saguinus | oedipus | Saguinus oedipus | 462.04 | 1.9 | 51020.84 | 120.29 | 26.343 | 3 | 4 | 1 | 2 | 2 | 1.118141 |
| Rodentia | Cricetidae | Akodon | serrensis | Akodon serrensis | 28.3 | NA | 51047.49 | 152.66 | 19.934 | NA | NA | NA | NA | NA | 0.013492 |
| Rodentia | Muridae | Zyzomys | maini | Zyzomys maini | 93.99 | NA | 51093.3 | 90.76 | 26.53 | NA | NA | NA | NA | NA | 0.182477 |
| Rodentia | Muridae | Chiruromys | vates | Chiruromys vates | 45.46 | 1.07 | 51126.1 | 334.35 | 20.237 | NA | NA | 2 | 2 | NA | 0.018693 |
| Carnivora | Mustelidae | Mustela | subpalmata | Mustela subpalmata | NA | NA | 51212.85 | 5.34 | 20.131 | NA | NA | NA | NA | NA | 0.015165 |
| Rodentia | Echimyidae | Proechimys | canicollis | Proechimys canicollis | 312.57 | NA | 51317.01 | 56.41 | 24.942 | NA | NA | NA | NA | NA | 0.020662 |
| Rodentia | Muridae | Tateomys | rhinogradoides | Tateomys rhinogradoides | NA | NA | 51361.43 | 221.03 | 20.301 | NA | NA | NA | NA | NA | 0.26777 |
| Dasyuromorphia | Dasyuridae | Phascolosorex | dorsalis | Phascolosorex dorsalis | 123.03 | 3.39 | 51363.46 | 298.96 | 17.406 | 2 | 2 | 1 | 1 | 2 | 0.012732 |
| Rodentia | Ctenomyidae | Ctenomys | maulinus | Ctenomys maulinus | 215 | NA | 51536 | 60.56 | 6.108 | NA | 3 | 2 | 1 | 1 | 0.016918 |
| Chiroptera | Pteropodidae | Cynopterus | nusatenggara | Cynopterus nusatenggara | NA | NA | 51558.74 | 241.32 | 23.933 | NA | NA | 1 | 2 | NA | 0.015632 |
| Rodentia | Muridae | Gerbillus | latastei | Gerbillus latastei | NA | 4.86 | 51764.65 | 14.54 | 20.138 | NA | NA | NA | NA | NA | 0.022232 |
| Carnivora | Mustelidae | Mustela | lutreolina | Mustela lutreolina | 466.25 | NA | 52045.25 | 236.58 | 24.551 | 2 | 1 | 1 | 1 | 3 | 0.222996 |
| Lagomorpha | Leporidae | Lepus | corsicanus | Lepus corsicanus | NA | NA | 52093.48 | 69.15 | 14.719 | NA | NA | NA | NA | NA | 0.668674 |
| Primates | Lepilemuridae | Lepilemur | microdon | Lepilemur microdon | 952.33 | NA | 52152.56 | 183 | 19.715 | 1 | 4 | 1 | 2 | 2 | 0.311582 |
| Rodentia | Ctenomyidae | Ctenomys | leucodon | Ctenomys leucodon | 243.6 | NA | 52340.92 | 72.39 | 6.93 | NA | 3 | 2 | 1 | 1 | 0.016918 |
| Soricomorpha | Soricidae | Cryptotis | goodwini | Cryptotis goodwini | 7 | NA | 52453.87 | 118.43 | 21.131 | NA | NA | 2 | 1 | NA | 0.022837 |
| Rodentia | Bathyergidae | Cryptomys | zechi | Cryptomys zechi | NA | NA | 52619.38 | 108.6 | 27.117 | NA | NA | NA | NA | NA | 0.019558 |
| Rodentia | Gliridae | Graphiurus | surdus | Graphiurus surdus | NA | 4.99 | 52630.81 | 176.22 | 23.571 | 1 | 4 | 2 | 2 | 2 | 0.31765 |
| Rodentia | Muridae | Thallomys | loringi | Thallomys loringi | NA | NA | 52750.18 | 62.9 | 19.257 | NA | NA | NA | NA | NA | 0.018142 |
| Rodentia | Geomyidae | Thomomys | monticola | Thomomys monticola | 80.99 | 3.49 | 52761.97 | 52.73 | 8.015 | NA | 3 | 2 | 1 | 1 | 0.019885 |
| Primates | Cercopithecidae | Trachypithecus | vetulus | Trachypithecus vetulus | 7205.08 | 1.01 | 52878.45 | 143.41 | 25.75 | 3 | 2 | NA | NA | 1 | 1.095785 |
| Rodentia | Muridae | Lophuromys | rahmi | Lophuromys rahmi | NA | 1.94 | 53044.48 | 100.43 | 18.572 | NA | NA | NA | NA | NA | 1.108756 |
| Rodentia | Muridae | Abeomelomys | sevia | Abeomelomys sevia | 54.88 | 1 | 53261.73 | 348.51 | 16.432 | NA | NA | 2 | 2 | NA | 0.018693 |
| Diprotodontia | Macropodidae | Macropus | parma | Macropus parma | 4156.62 | 0.9 | 53268.32 | 86.85 | 15.442 | NA | 1 | 1 | 1 | 1 | 0.149162 |
| Diprotodontia | Macropodidae | Petrogale | godmani | Petrogale godmani | 4750 | NA | 53365.92 | 101.62 | 24.655 | NA | NA | 1 | 1 | NA | 0.016833 |
| Rodentia | Cricetidae | Blanfordimys | bucharensis | Blanfordimys bucharensis | NA | NA | 53428.69 | 14.12 | 4.366 | NA | NA | NA | NA | NA | 0.017983 |
| Chiroptera | Vespertilionidae | Myotis | nesopolus | Myotis nesopolus | 3.56 | NA | 53573.46 | 47.93 | 25.902 | NA | NA | 1 | 2 | NA | 0.015862 |
| Chiroptera | Molossidae | Mops | petersoni | Mops petersoni | NA | NA | 53738.65 | 127.67 | 24.94 | NA | NA | 1 | 2 | NA | 0.794909 |
| Rodentia | Cricetidae | Lundomys | molitor | Lundomys molitor | 238.49 | 2.91 | 53868.95 | 85.78 | 16.153 | 2 | NA | 2 | 1 | NA | 0.010768 |
| Rodentia | Cricetidae | Reithrodontomys | chrysopsis | Reithrodontomys chrysopsis | 19 | 3.4 | 53949.66 | 89.52 | 17.745 | 1 | NA | 2 | 2 | NA | 0.018428 |
| Rodentia | Cricetidae | Arborimus | longicaudus | Arborimus longicaudus | 22.39 | 2.91 | 54116.11 | 95.78 | 8.309 | 1 | 1 | 2 | 2 | 1 | 0.135792 |
| Rodentia | Muridae | Gerbillus | andersoni | Gerbillus andersoni | 27.5 | 3.89 | 54377.38 | 8.48 | 19.97 | 1 | 1 | NA | NA | 1 | 0.022232 |
| Rodentia | Muridae | Xeromys | myoides | Xeromys myoides | 45.2 | 2 | 54426.5 | 103.71 | 23.963 | NA | 1 | 2 | 1 | 3 | 0.939806 |
| Chiroptera | Rhinolophidae | Rhinolophus | canuti | Rhinolophus canuti | NA | NA | 54571.58 | 181.58 | 24.143 | NA | 1 | 1 | 2 | 3 | 0.494796 |
| Rodentia | Abrocomidae | Abrocoma | bennettii | Abrocoma bennettii | 250.5 | 4.86 | 54615.98 | 20.44 | 1.766 | 1 | NA | 3 | 2 | NA | 0.02661 |
| Rodentia | Octodontidae | Octomys | mimax | Octomys mimax | 124.43 | NA | 54799.69 | 14.59 | 5.982 | 1 | 2 | 2 | 1 | 1 | 0.024578 |
| Rodentia | Ctenomyidae | Ctenomys | saltarius | Ctenomys saltarius | 230 | NA | 54806.9 | 55.35 | 5.408 | NA | 3 | 1 | 1 | 1 | 0.270692 |
| Rodentia | Muridae | Praomys | misonnei | Praomys misonnei | NA | NA | 54954.33 | 148 | 23.83 | NA | NA | NA | NA | NA | 0.019953 |
| Rodentia | Muridae | Chiropodomys | major | Chiropodomys major | 28.9 | NA | 55075.67 | 288.32 | 23.526 | NA | NA | NA | NA | NA | 0.291415 |
| Rodentia | Sciuridae | Funambulus | sublineatus | Funambulus sublineatus | 70.93 | 2 | 55081.67 | 137.77 | 26.291 | NA | NA | NA | NA | NA | 0.864651 |
| Rodentia | Cricetidae | Akodon | affinis | Akodon affinis | 24.89 | NA | 55129.8 | 202.1 | 21.003 | 2 | 4 | 1 | 1 | 2 | 0.013492 |
| Rodentia | Echimyidae | Proechimys | poliopus | Proechimys poliopus | 284.99 | NA | 55323.21 | 63.84 | 24.846 | NA | NA | NA | NA | NA | 1.033121 |
| Chiroptera | Vespertilionidae | Kerivoula | flora | Kerivoula flora | 6.01 | 1 | 55582.84 | 271.76 | 22.921 | NA | NA | 1 | 2 | NA | 0.78425 |
| Rodentia | Ctenomyidae | Ctenomys | peruanus | Ctenomys peruanus | 488.36 | 3 | 55816.24 | 125.29 | 5.215 | NA | 3 | 2 | 1 | 1 | 0.016918 |
| Rodentia | Muridae | Notomys | aquilo | Notomys aquilo | 38.99 | NA | 55857.79 | 104.03 | 26.609 | 1 | 4 | NA | NA | 2 | 1.426307 |
| Artiodactyla | Bovidae | Bubalus | quarlesi | Bubalus quarlesi | 181718.9 | 1 | 56312.24 | 228.75 | 20.508 | 3 | 3 | NA | NA | 1 | 1.109674 |
| Rodentia | Geomyidae | Geomys | attwateri | Geomys attwateri | 144.06 | 1.89 | 56381.49 | 66 | 20.376 | 2 | 2 | 2 | 1 | 1 | 0.015239 |
| Soricomorpha | Soricidae | Sorex | arizonae | Sorex arizonae | 2.7 | NA | 56446.9 | 27.23 | 16.817 | NA | NA | NA | NA | NA | 0.019481 |
| Rodentia | Cricetidae | Myodes | regulus | Myodes regulus | NA | NA | 56527.6 | 98.22 | 7.521 | NA | NA | NA | NA | NA | 0.013 |
| Rodentia | Cricetidae | Euneomys | petersoni | Euneomys petersoni | 83 | NA | 56740.83 | 93.03 | 3.292 | NA | NA | NA | NA | NA | 0.015865 |
| Primates | Lemuridae | Eulemur | rubriventer | Eulemur rubriventer | 2015.4 | 1.01 | 56908.42 | 178.11 | 19.433 | 2 | 4 | 1 | 2 | 2 | 0.859047 |
| Chiroptera | Pteropodidae | Pteropus | tonganus | Pteropus tonganus | 561.51 | 1 | 56956.39 | 172.39 | 22.89 | 2 | 1 | 1 | 2 | 1 | 0.015175 |
| Primates | Cercopithecidae | Macaca | silenus | Macaca silenus | 5995.25 | 1.01 | 56995.19 | 88.07 | 25.303 | 3 | 4 | 3 | 2 | 2 | 0.920952 |
| Lagomorpha | Ochotonidae | Ochotona | iliensis | Ochotona iliensis | NA | NA | 57046.16 | 15.53 | -1.796 | NA | NA | 1 | 1 | NA | 1.313695 |
| Rodentia | Cricetidae | Thomasomys | incanus | Thomasomys incanus | 77 | NA | 57159.77 | 94.3 | 15.154 | NA | NA | NA | NA | NA | 0.718446 |
| Afrosoricida | Tenrecidae | Micropotamogale | lamottei | Micropotamogale lamottei | 69.59 | 3.86 | 57563.41 | 189.05 | 24.132 | 1 | 2 | 3 | 2 | 3 | 1.803413 |
| Soricomorpha | Soricidae | Sorex | samniticus | Sorex samniticus | 8.24 | NA | 57915.64 | 73.64 | 12.849 | NA | NA | NA | NA | NA | 0.018136 |
| Rodentia | Cricetidae | Thomasomys | silvestris | Thomasomys silvestris | 77 | NA | 58234.91 | 153.52 | 17.244 | NA | NA | NA | NA | NA | 0.014369 |
| Chiroptera | Vespertilionidae | Myotis | scotti | Myotis scotti | NA | NA | 58291.83 | 85.86 | 16.328 | NA | NA | 1 | 2 | NA | 0.858415 |
| Soricomorpha | Soricidae | Sorex | pacificus | Sorex pacificus | 10.56 | 4.19 | 58327.78 | 81.96 | 8.15 | 1 | 3 | NA | NA | 2 | 0.016855 |
| Rodentia | Dipodidae | Salpingotus | heptneri | Salpingotus heptneri | NA | NA | 58531.41 | 6.8 | 9.304 | NA | NA | NA | NA | NA | 0.352001 |
| Rodentia | Erethizontidae | Coendou | rothschildi | Coendou rothschildi | 2000 | NA | 58668.52 | 197.46 | 25.861 | 1 | 2 | 1 | 2 | 1 | 0.014307 |
| Primates | Cheirogaleidae | Mirza | coquereli | Mirza coquereli | 326.5 | 1.71 | 58761.86 | 92.48 | 23.889 | 1 | 4 | 1 | 2 | 2 | 0.229197 |
| Rodentia | Cricetidae | Microtus | sachalinensis | Microtus sachalinensis | NA | 7.89 | 58787.82 | 49.29 | -1.367 | NA | NA | NA | NA | NA | 0.132196 |
| Microbiotheria | Microbiotheriidae | Dromiciops | gliroides | Dromiciops gliroides | 25 | 3 | 58876.08 | 106.14 | 9.093 | 1 | 6 | 2 | 2 | 2 | 0.289251 |
| Chiroptera | Vespertilionidae | Murina | fusca | Murina fusca | NA | NA | 58971.74 | 28.77 | 3.223 | NA | NA | 1 | 2 | NA | 0.218417 |
| Rodentia | Heteromyidae | Chaetodipus | goldmani | Chaetodipus goldmani | NA | NA | 59093.22 | 25.66 | 20.52 | NA | NA | NA | NA | NA | 0.709424 |
| Rodentia | Bathyergidae | Bathyergus | suillus | Bathyergus suillus | 777.38 | 2.89 | 59404.22 | 31.42 | 15.893 | NA | 3 | 2 | 1 | 1 | 0.017809 |
| Chiroptera | Emballonuridae | Taphozous | kapalgensis | Taphozous kapalgensis | 26.45 | NA | 59481.74 | 101.68 | 26.984 | 1 | NA | 1 | 2 | NA | 0.021906 |
| Rodentia | Cricetidae | Kunsia | fronto | Kunsia fronto | 167.99 | NA | 59646.88 | 124.77 | 20.304 | NA | 2 | NA | NA | 1 | 1.141399 |
| Rodentia | Muridae | Chiropodomys | pusillus | Chiropodomys pusillus | 28.9 | NA | 59722.74 | 266.76 | 23.851 | NA | NA | NA | NA | NA | 0.291415 |
| Rodentia | Heteromyidae | Dipodomys | agilis | Dipodomys agilis | 60.21 | 2.6 | 59736.35 | 32.27 | 12.209 | NA | NA | NA | NA | NA | 0.012579 |
| Rodentia | Muridae | Niviventer | excelsior | Niviventer excelsior | NA | NA | 60049.13 | 101.12 | 7.516 | NA | NA | NA | NA | NA | 0.021458 |
| Primates | Pitheciidae | Callicebus | melanochir | Callicebus melanochir | NA | NA | 60497.71 | 137.48 | 23.278 | NA | NA | NA | NA | NA | 0.931666 |
| Primates | Lemuridae | Varecia | variegata | Varecia variegata | 3849.99 | 2.16 | 60508.59 | 188.58 | 19.9 | 2 | 4 | 2 | 2 | 1 | 1.931505 |
| Primates | Cercopithecidae | Cercopithecus | preussi | Cercopithecus preussi | 5132.57 | 1.02 | 60552.81 | 234.82 | 24.541 | 3 | 4 | NA | NA | 2 | 0.794374 |
| Rodentia | Nesomyidae | Petromyscus | barbouri | Petromyscus barbouri | NA | NA | 60647.65 | 11.84 | 16.655 | NA | NA | NA | NA | NA | 0.020919 |
| Primates | Cebidae | Callithrix | chrysoleuca | Callithrix chrysoleuca | NA | NA | 60733.28 | 170.99 | 25.862 | 3 | NA | NA | NA | NA | 0.176416 |
| Rodentia | Echimyidae | Trinomys | albispinus | Trinomys albispinus | 284.99 | NA | 60927.75 | 107.94 | 23.631 | NA | NA | NA | NA | NA | 0.020662 |
| Rodentia | Dasyproctidae | Dasyprocta | kalinowskii | Dasyprocta kalinowskii | 2650.03 | NA | 61009.2 | 287.95 | 15.549 | 3 | 3 | 1 | 1 | 1 | 0.291464 |
| Diprotodontia | Macropodidae | Dorcopsis | luctuosa | Dorcopsis luctuosa | 4939.56 | 1.01 | 61047.01 | 173.41 | 24.115 | 2 | 3 | 1 | 1 | 1 | 0.793237 |
| Rodentia | Heteromyidae | Dipodomys | panamintinus | Dipodomys panamintinus | 73.67 | 3.78 | 61106.76 | 32.89 | 9.829 | 1 | NA | 2 | 1 | NA | 0.013453 |
| Artiodactyla | Bovidae | Pseudois | schaeferi | Pseudois schaeferi | 29249.99 | NA | 61143.76 | 160.67 | -1.181 | NA | NA | 1 | 1 | NA | 1.130942 |
| Afrosoricida | Tenrecidae | Microgale | pusilla | Microgale pusilla | 3.4 | NA | 61204.09 | 162.43 | 20.205 | NA | NA | 2 | 2 | NA | 0.021013 |
| Soricomorpha | Soricidae | Sorex | maritimensis | Sorex maritimensis | NA | NA | 61243.95 | 96.38 | 5.545 | NA | NA | NA | NA | NA | 0.015033 |
| Rodentia | Muridae | Rattus | annandalei | Rattus annandalei | 197.49 | NA | 61557.6 | 195.77 | 25.914 | NA | NA | NA | NA | NA | 0.016703 |
| Afrosoricida | Tenrecidae | Echinops | telfairi | Echinops telfairi | 152.25 | 5.49 | 61701.13 | 57.94 | 23.645 | 1 | 1 | 2 | 2 | 3 | 0.02357 |
| Primates | Lemuridae | Lemur | catta | Lemur catta | 2626.48 | 1.18 | 61778.7 | 62.94 | 22.971 | 2 | 4 | 2 | 2 | 2 | 0.227385 |
| Peramelemorphia | Peramelidae | Isoodon | auratus | Isoodon auratus | 425 | 2.6 | 61802.53 | 79.91 | 27.91 | 2 | 2 | 2 | 1 | 2 | 0.841888 |
| Dasyuromorphia | Dasyuridae | Sminthopsis | psammophila | Sminthopsis psammophila | 33.5 | 5.49 | 61813.87 | 16.54 | 18.242 | 2 | 2 | 1 | 1 | 3 | 1.348822 |
| Primates | Cercopithecidae | Piliocolobus | preussi | Piliocolobus preussi | 8865.71 | 1.01 | 62033.7 | 227.62 | 24.207 | 3 | 4 | 2 | 2 | 1 | 1.358434 |
| Rodentia | Sciuridae | Trogopterus | xanthipes | Trogopterus xanthipes | NA | 1.64 | 62202.44 | 69.48 | 6.45 | NA | 1 | NA | NA | 1 | 0.140883 |
| Rodentia | Cricetidae | Andalgalomys | pearsoni | Andalgalomys pearsoni | 25.4 | NA | 62301.94 | 76.13 | 25.418 | NA | NA | 1 | 1 | NA | 0.013854 |
| Rodentia | Muridae | Pseudomys | occidentalis | Pseudomys occidentalis | 34 | 3 | 62370.27 | 46.35 | 15.138 | 1 | 3 | 1 | 1 | 1 | 0.021649 |
| Scandentia | Tupaiidae | Tupaia | montana | Tupaia montana | 168.05 | 2.01 | 62408.01 | 267.88 | 23.774 | 3 | 4 | 1 | 1 | 2 | 0.023474 |
| Rodentia | Cricetidae | Akodon | latebricola | Akodon latebricola | 38.99 | NA | 62437.75 | 153.63 | 16.419 | NA | NA | NA | NA | NA | 0.648918 |
| Primates | Cebidae | Cebus | xanthosternos | Cebus xanthosternos | NA | NA | 62866.63 | 116.23 | 21.659 | 3 | NA | NA | NA | NA | 1.550786 |
| Rodentia | Hystricidae | Hystrix | sumatrae | Hystrix sumatrae | NA | NA | 63160.2 | 205.24 | 24.942 | NA | 6 | 2 | 1 | 2 | 0.019685 |
| Afrosoricida | Tenrecidae | Geogale | aurita | Geogale aurita | 6.69 | 3.4 | 63241.93 | 57.89 | 23.607 | 1 | 1 | 1 | 1 | 3 | 0.028322 |
| Rodentia | Sciuridae | Sciurus | pyrrhinus | Sciurus pyrrhinus | 482 | NA | 63480.04 | 138.42 | 21.1 | 3 | NA | 2 | 2 | NA | 0.17702 |
| Rodentia | Capromyidae | Mysateles | prehensilis | Mysateles prehensilis | NA | 2 | 63506.47 | 111.22 | 25.062 | NA | 3 | 1 | 2 | 2 | 1.311882 |
| Dasyuromorphia | Dasyuridae | Sarcophilus | harrisii | Sarcophilus harrisii | 8202.25 | 2.88 | 63567.96 | 71.3 | 10.427 | 2 | 1 | 1 | 1 | 3 | 1.056315 |
| Diprotodontia | Macropodidae | Dorcopsis | muelleri | Dorcopsis muelleri | 5370.79 | 1 | 63643.38 | 207.48 | 23.01 | NA | NA | 1 | 1 | NA | 0.015865 |
| Primates | Cebidae | Saguinus | nigricollis | Saguinus nigricollis | 450 | 1.82 | 63703.04 | 256.15 | 26.118 | 3 | NA | 1 | 2 | NA | 0.0158 |
| Rodentia | Echimyidae | Trinomys | dimidiatus | Trinomys dimidiatus | 167.6 | 2 | 63705.5 | 135.72 | 18.836 | NA | NA | NA | NA | NA | 0.020662 |
| Rodentia | Cricetidae | Phyllotis | magister | Phyllotis magister | 68.49 | NA | 64146.09 | 13.5 | 7.346 | NA | 3 | 1 | 1 | 2 | 0.016023 |
| Rodentia | Muridae | Stenocephalemys | albocaudata | Stenocephalemys albocaudata | 144 | NA | 64708.89 | 77.76 | 11.519 | NA | NA | NA | NA | NA | 0.019357 |
| Rodentia | Cricetidae | Peromyscus | eva | Peromyscus eva | 21.99 | NA | 64741.35 | 12.43 | 19.897 | 1 | NA | NA | NA | NA | 0.017825 |
| Rodentia | Octodontidae | Octodon | degus | Octodon degus | 203.27 | 5.31 | 64949.08 | 26.48 | 6.347 | 2 | 7 | 3 | 2 | 2 | 0.02075 |
| Rodentia | Cricetidae | Hyperacrius | fertilis | Hyperacrius fertilis | 22.25 | 2.5 | 64975.52 | 57.3 | 10.453 | NA | NA | NA | NA | NA | 0.859563 |
| Rodentia | Cricetidae | Hyperacrius | wynnei | Hyperacrius wynnei | 52.6 | 2.5 | 64975.52 | 57.3 | 10.453 | NA | NA | NA | NA | NA | 0.017191 |
| Chiroptera | Pteropodidae | Pteropus | lombocensis | Pteropus lombocensis | 256.15 | NA | 65052.84 | 200.69 | 24.261 | NA | NA | 1 | 2 | NA | 0.234863 |
| Primates | Cebidae | Saguinus | bicolor | Saguinus bicolor | 465 | 2 | 65246.44 | 129.99 | 26.218 | 3 | 4 | 1 | 2 | 2 | 0.970796 |
| Rodentia | Geomyidae | Geomys | knoxjonesi | Geomys knoxjonesi | 172.49 | NA | 65255.15 | 32.6 | 17.622 | NA | 2 | 1 | 1 | 1 | 0.015239 |
| Rodentia | Nesomyidae | Petromyscus | shortridgei | Petromyscus shortridgei | NA | NA | 65327.98 | 25.63 | 17.309 | NA | NA | NA | NA | NA | 0.020919 |
| Primates | Indriidae | Propithecus | verreauxi | Propithecus verreauxi | 3588.26 | 1.02 | 65373.06 | 58.13 | 23.537 | 3 | 3 | 1 | 2 | 1 | 0.928952 |
| Rodentia | Nesomyidae | Nesomys | rufus | Nesomys rufus | 163.96 | NA | 65566.48 | 169.07 | 19.18 | 2 | 2 | NA | NA | 1 | 0.019631 |
| Soricomorpha | Soricidae | Sorex | tenellus | Sorex tenellus | 3.8 | NA | 65627.48 | 22.22 | 9.995 | NA | NA | NA | NA | NA | 0.018367 |
| Soricomorpha | Soricidae | Crocidura | horsfieldii | Crocidura horsfieldii | 5.47 | 3.14 | 65979.11 | 91.38 | 15.527 | NA | NA | NA | NA | NA | 0.256845 |
| Chiroptera | Phyllostomidae | Musonycteris | harrisoni | Musonycteris harrisoni | NA | NA | 66456.72 | 101.03 | 22.008 | NA | NA | 1 | 2 | NA | 0.638954 |
| Dasyuromorphia | Dasyuridae | Dasyurus | viverrinus | Dasyurus viverrinus | 1101.49 | 4.7 | 66496.29 | 71.08 | 10.505 | 2 | 4 | 2 | 1 | 2 | 0.12197 |
| Diprotodontia | Macropodidae | Thylogale | billardierii | Thylogale billardierii | 5865.86 | 1 | 66496.92 | 71.08 | 10.505 | NA | NA | 1 | 1 | NA | 0.0151 |
| Chiroptera | Natalidae | Nyctiellus | lepidus | Nyctiellus lepidus | NA | NA | 66549.07 | 111.11 | 25.096 | NA | 1 | 1 | 2 | 3 | 0.022541 |
| Primates | Cercopithecidae | Macaca | sinica | Macaca sinica | 4655.99 | 1.01 | 66626.48 | 141.67 | 25.811 | 3 | 2 | NA | NA | 1 | 1.164973 |
| Rodentia | Muridae | Mus | fernandoni | Mus fernandoni | NA | NA | 66626.48 | 141.67 | 25.811 | NA | NA | NA | NA | NA | 0.780019 |
| Rodentia | Muridae | Mus | mayori | Mus mayori | NA | 2.95 | 66626.48 | 141.67 | 25.811 | NA | NA | NA | NA | NA | 0.894331 |
| Chiroptera | Pteropodidae | Aproteles | bulmerae | Aproteles bulmerae | 623.91 | 1 | 66662.67 | 354.23 | 17.117 | NA | 2 | 1 | 2 | 1 | 1.840076 |
| Rodentia | Cricetidae | Oryzomys | polius | Oryzomys polius | 60.5 | NA | 66779.09 | 147.9 | 24.651 | NA | NA | NA | NA | NA | 0.243516 |
| Scandentia | Tupaiidae | Dendrogale | melanura | Dendrogale melanura | 59.99 | NA | 66841.07 | 270.18 | 23.717 | NA | NA | 2 | 2 | NA | 0.33086 |
| Rodentia | Cricetidae | Peromyscus | perfulvus | Peromyscus perfulvus | 40 | 2.79 | 66891.6 | 91.14 | 21.775 | 1 | NA | 2 | 2 | NA | 0.017825 |
| Rodentia | Sciuridae | Petinomys | hageni | Petinomys hageni | NA | NA | 67222.49 | 239.54 | 25.755 | 1 | 4 | NA | NA | 2 | 0.243011 |
| Rodentia | Sciuridae | Tamias | speciosus | Tamias speciosus | 60.83 | 4.14 | 67913.09 | 45.47 | 8.777 | 3 | 5 | 2 | 2 | 2 | 0.016364 |
| Soricomorpha | Soricidae | Crocidura | lea | Crocidura lea | NA | NA | 67923.05 | 220.06 | 20.769 | NA | NA | NA | NA | NA | 0.01789 |
| Soricomorpha | Soricidae | Cryptotis | mexicana | Cryptotis mexicana | 7 | 2.99 | 68045.83 | 114.7 | 20.974 | NA | NA | 1 | 1 | NA | 0.019127 |
| Primates | Cercopithecidae | Semnopithecus | ajax | Semnopithecus ajax | NA | NA | 68186.13 | 93.6 | 11.199 | NA | NA | NA | NA | NA | 1.001027 |
| Rodentia | Nesomyidae | Eliurus | majori | Eliurus majori | 100 | NA | 68679.86 | 174.65 | 19.768 | NA | NA | NA | NA | NA | 0.017257 |
| Chiroptera | Emballonuridae | Balantiopteryx | infusca | Balantiopteryx infusca | NA | NA | 68801.56 | 121.57 | 24.22 | 1 | NA | 1 | 2 | NA | 1.604271 |
| Rodentia | Muridae | Thamnomys | kempi | Thamnomys kempi | 75 | NA | 68852.98 | 105.83 | 18.846 | NA | NA | NA | NA | NA | 0.928204 |
| Rodentia | Cricetidae | Podomys | floridanus | Podomys floridanus | 30.74 | 2.6 | 69082.12 | 112.22 | 22.198 | 1 | 4 | 2 | 1 | 2 | 0.945014 |
| Primates | Galagidae | Galago | orinus | Galago orinus | NA | NA | 69256.66 | 71.04 | 22.683 | NA | NA | NA | NA | NA | 0.207078 |
| Dasyuromorphia | Dasyuridae | Pseudantechinus | bilarni | Pseudantechinus bilarni | 23.05 | 4.75 | 69385 | 91.34 | 26.959 | 2 | NA | 1 | 1 | NA | 0.156651 |
| Rodentia | Ctenomyidae | Ctenomys | boliviensis | Ctenomys boliviensis | 462.6 | NA | 70023.94 | 103.11 | 25.202 | NA | 3 | 2 | 1 | 1 | 0.015139 |
| Soricomorpha | Soricidae | Sorex | granarius | Sorex granarius | 6.39 | 4.89 | 70026.27 | 71.04 | 12.575 | NA | NA | NA | NA | NA | 0.013156 |
| Primates | Aotidae | Aotus | miconax | Aotus miconax | 800 | NA | 70306.69 | 141.98 | 24.078 | 1 | 2 | 1 | 2 | 2 | 1.070966 |
| Rodentia | Muridae | Phloeomys | pallidus | Phloeomys pallidus | 1744.14 | NA | 70334.12 | 225.12 | 23.007 | 1 | NA | 1 | 2 | NA | 0.020141 |
| Rodentia | Heteromyidae | Dipodomys | heermanni | Dipodomys heermanni | 63.08 | 3.11 | 70348.64 | 40.92 | 12.775 | 1 | 4 | 2 | 1 | 2 | 0.012774 |
| Chiroptera | Rhinolophidae | Rhinolophus | keyensis | Rhinolophus keyensis | 6.26 | 1 | 70406.34 | 212.21 | 24.355 | NA | NA | 1 | 2 | NA | 0.195316 |
| Rodentia | Dasyproctidae | Dasyprocta | mexicana | Dasyprocta mexicana | 4999.99 | NA | 70505.63 | 139.62 | 24.482 | 3 | 3 | 1 | 1 | 1 | 1.730566 |
| Rodentia | Muridae | Zyzomys | woodwardi | Zyzomys woodwardi | 95.02 | 2.23 | 70724.82 | 79.45 | 28.041 | NA | NA | NA | NA | NA | 0.018248 |
| Rodentia | Cricetidae | Alticola | montosa | Alticola montosa | NA | NA | 70730.17 | 33.52 | -1.648 | NA | NA | NA | NA | NA | 0.713545 |
| Soricomorpha | Soricidae | Suncus | malayanus | Suncus malayanus | 4.99 | NA | 70847.37 | 201.25 | 25.079 | NA | NA | NA | NA | NA | 0.272986 |
| Soricomorpha | Soricidae | Crocidura | serezkyensis | Crocidura serezkyensis | NA | NA | 71197.69 | 16.64 | 14.447 | NA | NA | NA | NA | NA | 0.015389 |
| Cingulata | Dasypodidae | Dasypus | pilosus | Dasypus pilosus | 4444.98 | NA | 71200.05 | 118.89 | 15.56 | NA | 3 | 2 | 1 | 2 | 1.027452 |
| Rodentia | Muridae | Leptomys | ernstmayri | Leptomys ernstmayri | 42.85 | NA | 71234.08 | 269.19 | 20.035 | NA | 1 | 1 | 1 | 3 | 0.017095 |
| Rodentia | Muridae | Maxomys | baeodon | Maxomys baeodon | 158.96 | NA | 71553.03 | 282.34 | 23.375 | NA | NA | NA | NA | NA | 0.343325 |
| Primates | Lemuridae | Eulemur | rufus | Eulemur rufus | 2394.62 | NA | 71558.47 | 106.88 | 22.842 | 2 | NA | NA | NA | NA | 0.27526 |
| Rodentia | Cricetidae | Phyllotis | wolffsohni | Phyllotis wolffsohni | 42.5 | NA | 71594.56 | 63.63 | 15.48 | NA | NA | NA | NA | NA | 0.016023 |
| Diprotodontia | Pseudocheiridae | Pseudochirulus | mayeri | Pseudochirulus mayeri | 151.75 | 1.26 | 71728.11 | 257.96 | 18.147 | NA | 3 | 1 | 2 | 1 | 0.021648 |
| Rodentia | Cricetidae | Akodon | dolores | Akodon dolores | 50.49 | 3.89 | 72177.75 | 55.24 | 16.15 | NA | NA | NA | NA | NA | 0.009155 |
| Rodentia | Cricetidae | Thomasomys | vestitus | Thomasomys vestitus | 76.5 | NA | 72313.5 | 154.57 | 25.442 | NA | NA | NA | NA | NA | 0.014369 |
| Rodentia | Muridae | Margaretamys | beccarii | Margaretamys beccarii | NA | NA | 72428.12 | 220.09 | 20.21 | NA | NA | NA | NA | NA | 0.884476 |
| Soricomorpha | Soricidae | Crocidura | baileyi | Crocidura baileyi | NA | NA | 72438.02 | 98.83 | 16.498 | NA | NA | NA | NA | NA | 1.34172 |
| Macroscelidea | Macroscelididae | Rhynchocyon | petersi | Rhynchocyon petersi | 423.99 | 1.93 | 72695.75 | 96.03 | 24.836 | NA | NA | NA | NA | NA | 1.228015 |
| Diprotodontia | Phalangeridae | Phalanger | mimicus | Phalanger mimicus | NA | NA | 72824.49 | 305.2 | 23.456 | NA | NA | NA | NA | NA | 0.016412 |
| Rodentia | Nesomyidae | Eliurus | minor | Eliurus minor | 57.53 | NA | 72857.79 | 187.18 | 19.729 | 1 | NA | 1 | 2 | NA | 0.017257 |
| Rodentia | Echimyidae | Makalata | grandis | Makalata grandis | 584 | NA | 73011.97 | 148.5 | 25.905 | 1 | NA | 1 | 2 | NA | 0.01783 |
| Rodentia | Cricetidae | Akodon | orophilus | Akodon orophilus | 38.99 | NA | 73051.28 | 128.09 | 20.687 | NA | NA | NA | NA | NA | 0.013492 |
| Primates | Cebidae | Callithrix | intermedia | Callithrix intermedia | NA | 2 | 73178.5 | 179.43 | 24.952 | 3 | NA | NA | NA | NA | 0.010941 |
| Lagomorpha | Leporidae | Sylvilagus | transitionalis | Sylvilagus transitionalis | 813.51 | 4.41 | 73236.48 | 86.14 | 7.666 | 2 | 3 | 1 | 1 | 1 | 0.855191 |
| Rodentia | Cricetidae | Notiomys | edwardsii | Notiomys edwardsii | 21.3 | NA | 73368.05 | 33.51 | 6.393 | NA | 1 | 2 | 1 | 3 | 0.019815 |
| Rodentia | Sciuridae | Funisciurus | substriatus | Funisciurus substriatus | 185.73 | NA | 73507.66 | 131.24 | 26.708 | NA | NA | NA | NA | NA | 0.295697 |
| Soricomorpha | Soricidae | Crocidura | nigripes | Crocidura nigripes | NA | NA | 73630.86 | 221.77 | 20.487 | NA | NA | NA | NA | NA | 0.01789 |
| Rodentia | Cricetidae | Neotoma | devia | Neotoma devia | 200 | NA | 73864.9 | 18.87 | 16.799 | NA | NA | NA | NA | NA | 0.015637 |
| Rodentia | Heteromyidae | Perognathus | inornatus | Perognathus inornatus | 10.16 | 4 | 73899.38 | 42.43 | 13.578 | 1 | 2 | 2 | 1 | 2 | 0.020442 |
| Dasyuromorphia | Dasyuridae | Phascogale | calura | Phascogale calura | 43.03 | 7.5 | 74127.1 | 39.62 | 15.544 | 2 | 3 | 2 | 2 | 2 | 0.160232 |
| Primates | Galagidae | Galago | nyasae | Galago nyasae | NA | NA | 74222.64 | 85.56 | 23.338 | NA | NA | NA | NA | NA | 0.308648 |
| Primates | Cercopithecidae | Presbytis | chrysomelas | Presbytis chrysomelas | NA | NA | 74224.44 | 284.35 | 24.231 | NA | NA | NA | NA | NA | 1.111808 |
| Rodentia | Cricetidae | Akodon | neocenus | Akodon neocenus | 42.39 | NA | 74279.32 | 24.08 | 14.094 | NA | NA | NA | NA | NA | 0.146488 |
| Rodentia | Sciuridae | Tamias | cinereicollis | Tamias cinereicollis | 61.59 | 4.89 | 74328.35 | 28.88 | 13.311 | NA | 6 | 2 | 2 | 2 | 0.011583 |
| Chiroptera | Vespertilionidae | Murina | aenea | Murina aenea | 7.5 | NA | 74408.21 | 220.26 | 24.665 | NA | NA | 1 | 2 | NA | 0.749218 |
| Rodentia | Cricetidae | Euneomys | chinchilloides | Euneomys chinchilloides | 75.57 | NA | 74442.36 | 47.29 | 4.435 | NA | NA | 1 | 1 | NA | 0.253837 |
| Rodentia | Cricetidae | Prometheomys | schaposchnikowi | Prometheomys schaposchnikowi | 75 | 3.4 | 74444 | 66.37 | 3.1 | NA | NA | NA | NA | NA | 0.185783 |
| Notoryctemorphia | Notoryctidae | Notoryctes | caurinus | Notoryctes caurinus | NA | NA | 74482.27 | 31.2 | 24.418 | NA | NA | 1 | 1 | NA | 0.390365 |
| Chiroptera | Natalidae | Natalus | major | Natalus major | NA | NA | 74512.66 | 104.89 | 23.905 | NA | NA | NA | NA | NA | 0.224774 |
| Soricomorpha | Solenodontidae | Solenodon | paradoxus | Solenodon paradoxus | 899.99 | 1.64 | 74512.66 | 104.89 | 23.905 | 2 | 2 | 3 | 2 | 3 | 2.149992 |
| Soricomorpha | Soricidae | Crocidura | arispa | Crocidura arispa | NA | NA | 74611 | 54.42 | 11.978 | NA | NA | NA | NA | NA | 0.015389 |
| Rodentia | Echimyidae | Dactylomys | peruanus | Dactylomys peruanus | 382 | NA | 74745.24 | 190.99 | 17.209 | NA | NA | NA | NA | NA | 0.237862 |
| Chiroptera | Pteropodidae | Acerodon | mackloti | Acerodon mackloti | 467.93 | 0.98 | 74770.92 | 204.54 | 24.32 | NA | 1 | 1 | 2 | 1 | 0.764458 |
| Rodentia | Sciuridae | Spermophilus | saturatus | Spermophilus saturatus | NA | NA | 74865.2 | 82.05 | 4.708 | NA | NA | NA | NA | NA | 0.014514 |
| Didelphimorphia | Didelphidae | Marmosa | xerophila | Marmosa xerophila | 46.2 | NA | 74940.72 | 58.71 | 25.842 | 1 | NA | 1 | 2 | NA | 1.24918 |
| Rodentia | Echimyidae | Proechimys | decumanus | Proechimys decumanus | 284.99 | NA | 74964.95 | 96.5 | 23.833 | NA | NA | NA | NA | NA | 1.033121 |
| Carnivora | Canidae | Lycalopex | sechurae | Lycalopex sechurae | 4234 | NA | 75180.74 | 15 | 16.929 | 1 | 7 | 1 | 1 | 2 | 0.096444 |
| Rodentia | Capromyidae | Plagiodontia | aedium | Plagiodontia aedium | 1268.86 | 1.14 | 75198.74 | 104.89 | 23.905 | 1 | 3 | 1 | 1 | 1 | 1.590243 |
| Chiroptera | Emballonuridae | Emballonura | dianae | Emballonura dianae | NA | NA | 75205.95 | 343.56 | 21.13 | 1 | NA | 1 | 2 | NA | 0.023995 |
| Rodentia | Cricetidae | Thalpomys | lasiotis | Thalpomys lasiotis | 23.99 | NA | 75615.35 | 124.33 | 20.648 | NA | NA | NA | NA | NA | 0.016518 |
| Rodentia | Muridae | Praomys | rostratus | Praomys rostratus | NA | NA | 75658.69 | 211.26 | 24.568 | NA | NA | NA | NA | NA | 0.019953 |
| Rodentia | Nesomyidae | Brachytarsomys | albicauda | Brachytarsomys albicauda | 200 | NA | 75743.16 | 181.85 | 19.581 | NA | 2 | 1 | 2 | 1 | 0.019315 |
| Soricomorpha | Soricidae | Suncus | remyi | Suncus remyi | 4.03 | NA | 75819.4 | 139.24 | 24.146 | NA | NA | NA | NA | NA | 0.017062 |
| Rodentia | Cricetidae | Thomasomys | laniger | Thomasomys laniger | 35.74 | NA | 76001.8 | 108.3 | 19.844 | NA | NA | NA | NA | NA | 0.014369 |
| Soricomorpha | Soricidae | Crocidura | nigricans | Crocidura nigricans | 20 | 3.99 | 76023.56 | 81.77 | 20.146 | NA | NA | NA | NA | NA | 0.01789 |
| Rodentia | Cricetidae | Dicrostonyx | nunatakensis | Dicrostonyx nunatakensis | NA | NA | 76122.24 | 19.98 | -12.421 | NA | NA | NA | NA | NA | 0.014002 |
| Primates | Cebidae | Saguinus | geoffroyi | Saguinus geoffroyi | 492.5 | 1.97 | 76159.09 | 279.43 | 25.925 | 3 | 4 | 1 | 2 | 2 | 0.01177 |
| Rodentia | Muridae | Apodemus | rusiges | Apodemus rusiges | NA | NA | 76241.02 | 40.76 | 0.265 | NA | NA | NA | NA | NA | 0.014234 |
| Dasyuromorphia | Dasyuridae | Sminthopsis | granulipes | Sminthopsis granulipes | 25 | NA | 76315.75 | 41.56 | 15.818 | 2 | NA | 1 | 1 | NA | 0.019113 |
| Rodentia | Cricetidae | Nectomys | palmipes | Nectomys palmipes | NA | NA | 76476.81 | 126.21 | 23.697 | 1 | NA | 1 | 1 | NA | 0.016069 |
| Rodentia | Sciuridae | Paraxerus | lucifer | Paraxerus lucifer | 692.45 | NA | 76574.94 | 79.93 | 21.716 | NA | NA | NA | NA | NA | 0.283545 |
| Rodentia | Octodontidae | Spalacopus | cyanus | Spalacopus cyanus | 100.86 | 3.14 | 76659.01 | 30.41 | 8.128 | 2 | 2 | 2 | 1 | 1 | 0.026397 |
| Rodentia | Cricetidae | Akodon | mimus | Akodon mimus | 23.99 | NA | 76741.65 | 131.58 | 14.134 | NA | NA | NA | NA | NA | 0.012978 |
| Rodentia | Muridae | Aethomys | silindensis | Aethomys silindensis | NA | NA | 77194.26 | 89 | 22.069 | NA | NA | NA | NA | NA | 0.341551 |
| Rodentia | Muridae | Niviventer | tenaster | Niviventer tenaster | NA | NA | 77232.22 | 183.25 | 22.317 | NA | NA | NA | NA | NA | 0.021458 |
| Rodentia | Muridae | Meriones | chengi | Meriones chengi | NA | NA | 77321.17 | 16.77 | 5.47 | NA | NA | NA | NA | NA | 0.018016 |
| Rodentia | Ctenomyidae | Ctenomys | perrensi | Ctenomys perrensi | 400 | NA | 77423.01 | 122.04 | 19.729 | NA | 3 | 1 | 1 | 1 | 0.016918 |
| Rodentia | Cricetidae | Phyllotis | caprinus | Phyllotis caprinus | 50.8 | NA | 77627.4 | 53.05 | 9.791 | NA | NA | NA | NA | NA | 0.016023 |
| Rodentia | Cricetidae | Arborimus | albipes | Arborimus albipes | 23 | 2.73 | 77783.68 | 95.54 | 8.283 | NA | 3 | 2 | 2 | 1 | 0.013579 |
| Artiodactyla | Bovidae | Dorcatragus | megalotis | Dorcatragus megalotis | 10918.12 | 1 | 77951.19 | 14.58 | 24.349 | 3 | 3 | 1 | 1 | 1 | 0.859767 |
| Rodentia | Ctenomyidae | Ctenomys | conoveri | Ctenomys conoveri | 859.99 | NA | 78001.32 | 73.89 | 23.119 | NA | 3 | 2 | 1 | 1 | 0.016918 |
| Rodentia | Cricetidae | Myodes | rex | Myodes rex | NA | NA | 78510.41 | 85.74 | 3.648 | NA | NA | NA | NA | NA | 0.011652 |
| Chiroptera | Pteropodidae | Nyctimene | draconilla | Nyctimene draconilla | 30.15 | NA | 78720.86 | 268.88 | 24.661 | NA | 3 | 1 | 2 | 2 | 0.242071 |
| Afrosoricida | Tenrecidae | Microgale | longicaudata | Microgale longicaudata | 8.08 | NA | 78758.03 | 178.07 | 19.887 | 1 | 1 | 2 | 2 | 3 | 0.021013 |
| Rodentia | Sciuridae | Dremomys | gularis | Dremomys gularis | NA | NA | 79065.48 | 128.11 | 17.06 | NA | NA | NA | NA | NA | 0.016936 |
| Rodentia | Cricetidae | Akodon | sanctipaulensis | Akodon sanctipaulensis | 27.1 | NA | 79134.33 | 143.72 | 18.966 | NA | NA | NA | NA | NA | 0.215868 |
| Rodentia | Muridae | Rattus | colletti | Rattus colletti | 146.48 | NA | 79228.17 | 100.53 | 27.105 | NA | NA | NA | NA | NA | 0.016593 |
| Rodentia | Cricetidae | Eothenomys | proditor | Eothenomys proditor | NA | NA | 79297.07 | 135.29 | 6.221 | NA | NA | NA | NA | NA | 0.013 |
| Peramelemorphia | Peramelidae | Echymipera | clara | Echymipera clara | 1204.61 | 2.2 | 79519.52 | 257.88 | 25.129 | 1 | 2 | 2 | 1 | 2 | 0.019016 |
| Rodentia | Cricetidae | Handleyomys | fuscatus | Handleyomys fuscatus | 49.5 | NA | 79797.77 | 192.38 | 20.12 | NA | NA | NA | NA | NA | 0.015514 |
| Chiroptera | Natalidae | Natalus | primus | Natalus primus | NA | NA | 79953.26 | 97.62 | 25.138 | NA | NA | NA | NA | NA | 2.135353 |
| Rodentia | Muridae | Hapalomys | longicaudatus | Hapalomys longicaudatus | 70 | NA | 79996.41 | 233.51 | 24.933 | NA | 1 | NA | NA | 1 | 1.274527 |
| Chiroptera | Vespertilionidae | Murina | rozendaali | Murina rozendaali | NA | NA | 80063.86 | 210.06 | 25.235 | NA | NA | 1 | 2 | NA | 0.749218 |
| Rodentia | Sciuridae | Aeretes | melanopterus | Aeretes melanopterus | NA | NA | 80788.68 | 65.15 | 7.527 | NA | NA | NA | NA | NA | 0.161651 |
| Primates | Pitheciidae | Pithecia | albicans | Pithecia albicans | 2800.07 | NA | 80794.37 | 176.07 | 26.333 | 3 | NA | NA | NA | NA | 0.767308 |
| Rodentia | Geomyidae | Thomomys | mazama | Thomomys mazama | 93.07 | 3.79 | 80946.15 | 88.44 | 7.938 | NA | 3 | 2 | 1 | 1 | 0.019885 |
| Primates | Cercopithecidae | Trachypithecus | shortridgei | Trachypithecus shortridgei | NA | NA | 80950.94 | 153.88 | 10.962 | NA | NA | NA | NA | NA | 0.650129 |
| Rodentia | Gliridae | Myomimus | personatus | Myomimus personatus | NA | NA | 81095.72 | 22.36 | 11.322 | NA | NA | NA | NA | NA | 0.355238 |
| Primates | Cercopithecidae | Macaca | tonkeana | Macaca tonkeana | 10035.53 | NA | 81338.19 | 222.91 | 20.436 | 3 | NA | NA | NA | NA | 0.639069 |
| Carnivora | Herpestidae | Bdeogale | jacksoni | Bdeogale jacksoni | 2500 | NA | 81428.64 | 80.08 | 19.641 | NA | 2 | 1 | 1 | 3 | 0.175895 |
| Diprotodontia | Phalangeridae | Phalanger | vestitus | Phalanger vestitus | 1850 | 1.01 | 81554.54 | 326.78 | 18.15 | 1 | 3 | 1 | 2 | 1 | 0.016572 |
| Rodentia | Sciuridae | Sciurus | colliaei | Sciurus colliaei | 498 | 2.66 | 81663.79 | 52.37 | 20.272 | 3 | 2 | 2 | 2 | 1 | 0.010543 |
| Artiodactyla | Bovidae | Kobus | megaceros | Kobus megaceros | 85805.93 | NA | 82555.03 | 71.9 | 26.948 | NA | NA | NA | NA | NA | 1.424 |
| Diprotodontia | Macropodidae | Petrogale | purpureicollis | Petrogale purpureicollis | NA | NA | 82704.32 | 32.42 | 23.803 | NA | NA | NA | NA | NA | 0.014502 |
| Rodentia | Heteromyidae | Chaetodipus | fallax | Chaetodipus fallax | 19.19 | 3.45 | 82998.2 | 16.49 | 14.493 | NA | 3 | 1 | 1 | 1 | 0.018431 |
| Rodentia | Muridae | Mus | baoulei | Mus baoulei | NA | NA | 83068 | 127.38 | 26.157 | NA | NA | NA | NA | NA | 0.015632 |
| Artiodactyla | Cervidae | Mazama | bricenii | Mazama bricenii | 16499.85 | NA | 83134.92 | 106.98 | 19.315 | NA | NA | 1 | 1 | NA | 0.39668 |
| Chiroptera | Phyllostomidae | Erophylla | bombifrons | Erophylla bombifrons | 16.28 | NA | 83334.5 | 104.87 | 23.916 | NA | 1 | NA | NA | 1 | 0.016863 |
| Chiroptera | Vespertilionidae | Lasiurus | minor | Lasiurus minor | NA | NA | 83334.5 | 104.87 | 23.916 | NA | NA | NA | NA | NA | 0.712893 |
| Soricomorpha | Soricidae | Blarinella | wardi | Blarinella wardi | NA | NA | 83336.25 | 120.82 | 7.809 | NA | NA | 2 | 1 | NA | 0.020378 |
| Rodentia | Muridae | Maxomys | inflatus | Maxomys inflatus | NA | NA | 83340.37 | 261.89 | 24.196 | NA | NA | NA | NA | NA | 1.07289 |
| Rodentia | Cricetidae | Microtus | schelkovnikovi | Microtus schelkovnikovi | NA | NA | 83449.22 | 74 | 8.006 | NA | NA | NA | NA | NA | 0.142354 |
| Rodentia | Cricetidae | Akodon | spegazzinii | Akodon spegazzinii | 28.6 | NA | 83477.58 | 52.42 | 6.99 | NA | NA | NA | NA | NA | 0.013492 |
| Rodentia | Cricetidae | Aepeomys | lugens | Aepeomys lugens | 37 | NA | 83652.35 | 104.03 | 19.15 | NA | NA | 1 | 1 | NA | 0.015514 |
| Primates | Hylobatidae | Nomascus | siki | Nomascus siki | NA | NA | 83883.77 | 196.94 | 22.704 | NA | NA | NA | NA | NA | 1.156518 |
| Chiroptera | Pteropodidae | Dobsonia | peronii | Dobsonia peronii | 226.51 | 0.98 | 84055.51 | 204.26 | 24.137 | NA | 1 | 1 | 2 | 1 | 0.011263 |
| Rodentia | Cricetidae | Chilomys | instans | Chilomys instans | 19 | NA | 84512.45 | 106.61 | 19.76 | NA | NA | NA | NA | NA | 0.019815 |
| Rodentia | Heteromyidae | Chaetodipus | arenarius | Chaetodipus arenarius | 23 | 2.91 | 84613.78 | 13.35 | 18.115 | NA | NA | NA | NA | NA | 0.017098 |
| Diprotodontia | Phalangeridae | Phalanger | intercastellanus | Phalanger intercastellanus | 1747.5 | 2.02 | 84657.38 | 215.93 | 21.36 | NA | 4 | 1 | 2 | 1 | 0.016412 |
| Soricomorpha | Soricidae | Crocidura | latona | Crocidura latona | NA | NA | 84664.72 | 142.94 | 23.048 | NA | NA | NA | NA | NA | 0.01789 |
| Primates | Indriidae | Avahi | laniger | Avahi laniger | 1092.28 | 1.01 | 85075.49 | 176.9 | 19.746 | 1 | 3 | 2 | 2 | 1 | 0.02091 |
| Rodentia | Nesomyidae | Eliurus | webbi | Eliurus webbi | 61.13 | NA | 85117.66 | 178.33 | 20.016 | NA | 1 | NA | NA | 1 | 0.017257 |
| Soricomorpha | Soricidae | Crocidura | armenica | Crocidura armenica | NA | NA | 85332.27 | 39.05 | 6.889 | NA | NA | NA | NA | NA | 0.286234 |
| Diprotodontia | Macropodidae | Petrogale | inornata | Petrogale inornata | 4555.52 | 1 | 85550.06 | 78.81 | 20.621 | NA | NA | 1 | 1 | NA | 0.016864 |
| Soricomorpha | Soricidae | Crocidura | montis | Crocidura montis | 14.49 | NA | 86115.53 | 78.61 | 19.362 | NA | NA | NA | NA | NA | 0.01789 |
| Diprotodontia | Vombatidae | Lasiorhinus | latifrons | Lasiorhinus latifrons | 26163.8 | 1 | 86125.03 | 19.98 | 17.044 | 1 | 2 | 2 | 1 | 1 | 0.022893 |
| Rodentia | Sciuridae | Spermophilus | ralli | Spermophilus ralli | NA | NA | 86182.73 | 28.1 | -1.758 | NA | NA | NA | NA | NA | 0.008649 |
| Rodentia | Heteromyidae | Dipodomys | californicus | Dipodomys californicus | 84.95 | 2.6 | 86283.94 | 58.3 | 9.648 | NA | 4 | NA | NA | 1 | 0.014994 |
| Rodentia | Muridae | Gerbillus | perpallidus | Gerbillus perpallidus | 52.22 | 4.65 | 86678.61 | 5.57 | 20.27 | NA | NA | NA | NA | NA | 0.022232 |
| Rodentia | Echimyidae | Trinomys | setosus | Trinomys setosus | 284.99 | NA | 86849.07 | 107.38 | 20.259 | NA | NA | NA | NA | NA | 0.020662 |
| Rodentia | Cricetidae | Ichthyomys | hydrobates | Ichthyomys hydrobates | 66.39 | NA | 86926.23 | 112.61 | 18.532 | NA | 1 | 2 | 1 | 3 | 0.164037 |
| Carnivora | Eupleridae | Galidictis | fasciata | Galidictis fasciata | 549.99 | 1 | 87237.59 | 178.59 | 19.778 | 2 | 2 | 1 | 1 | 3 | 0.198286 |
| Rodentia | Sciuridae | Spermophilus | brevicauda | Spermophilus brevicauda | NA | NA | 87259.87 | 30.96 | 4.109 | NA | NA | NA | NA | NA | 0.008715 |
| Rodentia | Sciuridae | Exilisciurus | whiteheadi | Exilisciurus whiteheadi | 24.91 | 1.94 | 87340.95 | 284.42 | 23.482 | NA | NA | NA | NA | NA | 0.018795 |
| Primates | Cercopithecidae | Cercopithecus | erythrotis | Cercopithecus erythrotis | 3254.98 | 1.02 | 87403.19 | 214.26 | 24.093 | 3 | 2 | 1 | 2 | 2 | 0.81357 |
| Primates | Cercopithecidae | Presbytis | siamensis | Presbytis siamensis | 5895.06 | NA | 87550.36 | 214.58 | 25.407 | 3 | NA | NA | NA | NA | 0.117032 |
| Primates | Cebidae | Callithrix | aurita | Callithrix aurita | 386.2 | 2 | 87653.49 | 148.06 | 18.819 | 3 | 3 | 1 | 2 | 2 | 0.515782 |
| Rodentia | Platacanthomyidae | Platacanthomys | lasiurus | Platacanthomys lasiurus | NA | NA | 87720.27 | 159.54 | 25.692 | NA | NA | NA | NA | NA | 1.157273 |
| Chiroptera | Pteropodidae | Thoopterus | nigrescens | Thoopterus nigrescens | 66.12 | 0.98 | 87948.88 | 223.65 | 21.223 | NA | NA | 1 | 2 | NA | 0.017247 |
| Chiroptera | Vespertilionidae | Hypsugo | alaschanicus | Hypsugo alaschanicus | NA | NA | 87983.24 | 97.04 | 9.683 | NA | NA | NA | NA | NA | 0.017083 |
| Chiroptera | Phyllostomidae | Lonchorhina | marinkellei | Lonchorhina marinkellei | 17.67 | NA | 88343.63 | 262.06 | 24.504 | NA | NA | 1 | 2 | NA | 1.573831 |
| Chiroptera | Vespertilionidae | Vespadelus | douglasorum | Vespadelus douglasorum | 4.99 | NA | 88721.66 | 76.59 | 27.847 | NA | NA | 1 | 2 | NA | 0.017918 |
| Rodentia | Muridae | Lemniscomys | bellieri | Lemniscomys bellieri | NA | NA | 88830.3 | 115.44 | 26.651 | NA | NA | NA | NA | NA | 0.019499 |
| Soricomorpha | Talpidae | Talpa | romana | Talpa romana | 92.5 | NA | 88832.74 | 69.79 | 13.816 | 2 | 1 | 2 | 1 | 3 | 0.023985 |
| Primates | Hylobatidae | Nomascus | gabriellae | Nomascus gabriellae | NA | NA | 88851.07 | 142.76 | 24.915 | 3 | NA | NA | NA | NA | 1.156518 |
| Primates | Lemuridae | Hapalemur | griseus | Hapalemur griseus | 916 | 1.5 | 89049.73 | 177.28 | 19.744 | 2 | 2 | 3 | 2 | 1 | 0.997366 |
| Rodentia | Muridae | Hylomyscus | baeri | Hylomyscus baeri | NA | NA | 89121.81 | 119.87 | 26.319 | NA | NA | NA | NA | NA | 1.149667 |
| Lagomorpha | Leporidae | Sylvilagus | robustus | Sylvilagus robustus | NA | NA | 89282.05 | 26.23 | 18.865 | NA | NA | NA | NA | NA | 1.133725 |
| Rodentia | Heteromyidae | Chaetodipus | artus | Chaetodipus artus | NA | NA | 89639.36 | 39.44 | 19.518 | NA | 1 | NA | NA | 1 | 0.014188 |
| Afrosoricida | Tenrecidae | Microgale | drouhardi | Microgale drouhardi | 10.5 | NA | 89926.07 | 175.85 | 20.102 | NA | NA | NA | NA | NA | 0.020969 |
| Rodentia | Sciuridae | Funisciurus | carruthersi | Funisciurus carruthersi | 276.85 | NA | 90485.48 | 106.5 | 19.335 | NA | NA | NA | NA | NA | 0.018481 |
| Rodentia | Cricetidae | Juliomys | pictipes | Juliomys pictipes | 22.9 | NA | 90582.12 | 135.72 | 20.661 | NA | NA | NA | NA | NA | 0.016518 |
| Rodentia | Muridae | Mammelomys | rattoides | Mammelomys rattoides | 209.49 | 1.69 | 90705.71 | 234.26 | 25.535 | NA | 3 | 2 | 1 | 1 | 0.020235 |
| Rodentia | Cricetidae | Chionomys | roberti | Chionomys roberti | NA | 1.94 | 90790.68 | 77.97 | 2.475 | NA | NA | NA | NA | NA | 0.018112 |
| Rodentia | Cricetidae | Reithrodontomys | burti | Reithrodontomys burti | 20 | NA | 90816.12 | 26.17 | 22.582 | 1 | NA | 2 | 2 | NA | 0.294846 |
| Rodentia | Cricetidae | Zygodontomys | brunneus | Zygodontomys brunneus | 75.59 | NA | 91161.44 | 150.38 | 19.509 | NA | NA | NA | NA | NA | 0.016518 |
| Primates | Atelidae | Alouatta | pigra | Alouatta pigra | 7172.06 | 1.01 | 91384.8 | 134.13 | 25.078 | 3 | 2 | 1 | 2 | 1 | 1.146362 |
| Carnivora | Herpestidae | Herpestes | fuscus | Herpestes fuscus | NA | NA | 91597.58 | 132.76 | 25.519 | NA | NA | NA | NA | NA | 0.868346 |
| Rodentia | Sciuridae | Tamias | merriami | Tamias merriami | 74.77 | 4.5 | 91618.34 | 39.04 | 11.071 | 3 | 5 | NA | NA | 2 | 0.014687 |
| Rodentia | Muridae | Lophuromys | woosnami | Lophuromys woosnami | 42.14 | 1.97 | 91746.47 | 98.83 | 19.633 | NA | NA | NA | NA | NA | 0.014783 |
| Rodentia | Cricetidae | Delomys | dorsalis | Delomys dorsalis | 67.5 | NA | 91990.81 | 144.21 | 17.506 | 3 | NA | 1 | 1 | NA | 0.015285 |
| Rodentia | Nesomyidae | Eliurus | myoxinus | Eliurus myoxinus | 69.76 | NA | 92064.6 | 82.98 | 24.091 | 1 | 3 | 2 | 2 | 2 | 0.017257 |
| Rodentia | Echimyidae | Phyllomys | lamarum | Phyllomys lamarum | 215.19 | NA | 92612.71 | 127.15 | 23.557 | 1 | NA | 1 | 2 | NA | 0.285285 |
| Rodentia | Cricetidae | Oligoryzomys | arenalis | Oligoryzomys arenalis | 25.2 | NA | 92796.32 | 22.33 | 15.001 | NA | NA | NA | NA | NA | 0.013332 |
| Primates | Pitheciidae | Callicebus | pallescens | Callicebus pallescens | NA | NA | 93328.33 | 86.34 | 25.842 | NA | NA | NA | NA | NA | 0.014113 |
| Rodentia | Muridae | Gerbilliscus | afra | Gerbilliscus afra | 92.13 | 3.92 | 93519.01 | 27.98 | 15.815 | NA | NA | NA | NA | NA | 0.01967 |
| Rodentia | Cricetidae | Phyllotis | amicus | Phyllotis amicus | 20.19 | NA | 93686.6 | 21.19 | 8.741 | NA | NA | NA | NA | NA | 0.016023 |
| Diprotodontia | Burramyidae | Cercartetus | lepidus | Cercartetus lepidus | 8.03 | 3.33 | 93697.61 | 49.34 | 13.035 | 1 | 2 | 2 | 2 | 2 | 0.021551 |
| Rodentia | Cricetidae | Brucepattersonius | iheringi | Brucepattersonius iheringi | 43 | NA | 93980.28 | 125.57 | 18.076 | NA | 1 | NA | NA | 3 | 0.014623 |
| Rodentia | Gliridae | Graphiurus | microtis | Graphiurus microtis | NA | NA | 94285.38 | 89.97 | 20.609 | NA | NA | NA | NA | NA | 0.019853 |
| Afrosoricida | Tenrecidae | Microgale | cowani | Microgale cowani | 12.27 | NA | 94309.7 | 175.91 | 19.589 | NA | NA | 1 | 1 | NA | 0.020969 |
| Diprotodontia | Macropodidae | Macropus | eugenii | Macropus eugenii | 5278.48 | 1.01 | 94354.1 | 41.77 | 15.409 | NA | NA | 1 | 1 | NA | 0.017225 |
| Rodentia | Nesomyidae | Macrotarsomys | bastardi | Macrotarsomys bastardi | 28.45 | 2.5 | 94419.33 | 75.62 | 24.067 | 1 | 5 | 2 | 1 | 2 | 0.020687 |
| Erinaceomorpha | Erinaceidae | Podogymnura | truei | Podogymnura truei | NA | NA | 94772.99 | 170.68 | 23.639 | 1 | 2 | 2 | 1 | 2 | 0.023341 |
| Rodentia | Sciuridae | Sundasciurus | mindanensis | Sundasciurus mindanensis | NA | NA | 94772.99 | 170.68 | 23.639 | NA | NA | NA | NA | NA | 0.020635 |
| Rodentia | Cricetidae | Oryzomys | legatus | Oryzomys legatus | 61.5 | NA | 95064.98 | 78.03 | 18.751 | NA | NA | NA | NA | NA | 0.01522 |
| Rodentia | Sciuridae | Petaurillus | hosei | Petaurillus hosei | NA | NA | 95230.53 | 279.2 | 23.569 | 1 | NA | NA | NA | NA | 0.213518 |
| Primates | Cercopithecidae | Macaca | sylvanus | Macaca sylvanus | 11471.53 | 1.02 | 95331.43 | 45.93 | 14.784 | 3 | 6 | 2 | 2 | 2 | 1.229022 |
| Carnivora | Mephitidae | Spilogale | pygmaea | Spilogale pygmaea | 365 | 4 | 95598.41 | 97.2 | 21.713 | 2 | 6 | 1 | 1 | 2 | 0.875062 |
| Rodentia | Muridae | Apomys | insignis | Apomys insignis | 34.88 | NA | 95605.19 | 170.68 | 23.639 | 1 | NA | 2 | 2 | NA | 0.132218 |
| Rodentia | Cricetidae | Microtus | tatricus | Microtus tatricus | NA | 2.91 | 95800.84 | 60.87 | 5.571 | NA | NA | NA | NA | NA | 0.013894 |
| Rodentia | Cricetidae | Sigmodon | peruanus | Sigmodon peruanus | NA | NA | 95910.21 | 57.16 | 21.819 | NA | NA | NA | NA | NA | 0.014461 |
| Scandentia | Tupaiidae | Urogale | everetti | Urogale everetti | 300.79 | 1.69 | 96031.85 | 170.68 | 23.639 | NA | 4 | NA | NA | 2 | 0.020816 |
| Rodentia | Sciuridae | Petinomys | mindanensis | Petinomys mindanensis | NA | NA | 96031.86 | 170.68 | 23.639 | NA | NA | NA | NA | NA | 0.01297 |
| Rodentia | Cricetidae | Rhipidomys | latimanus | Rhipidomys latimanus | 57.5 | NA | 96039.74 | 176.56 | 22.788 | 1 | 4 | 1 | 2 | 2 | 0.015785 |
| Afrosoricida | Tenrecidae | Microgale | principula | Microgale principula | 10.2 | NA | 96125.86 | 177.02 | 19.775 | NA | NA | NA | NA | NA | 0.021013 |
| Afrosoricida | Tenrecidae | Microgale | talazaci | Microgale talazaci | 43.07 | 2.23 | 96252.65 | 169.39 | 20.078 | NA | 1 | 2 | 2 | 3 | 0.021013 |
| Rodentia | Cricetidae | Akodon | mollis | Akodon mollis | 30.39 | NA | 96269.64 | 157.29 | 22.599 | NA | NA | NA | NA | NA | 0.013492 |
| Rodentia | Muridae | Hapalomys | delacouri | Hapalomys delacouri | NA | NA | 96345.31 | 136.32 | 20.698 | NA | NA | NA | NA | NA | 0.849685 |
| Chiroptera | Pteropodidae | Pteropus | speciosus | Pteropus speciosus | NA | NA | 96863.56 | 170.68 | 23.639 | NA | NA | 1 | 2 | NA | 0.294088 |
| Rodentia | Muridae | Myomyscus | verreauxii | Myomyscus verreauxii | 41 | NA | 96936.75 | 32.09 | 15.551 | 1 | NA | NA | NA | NA | 0.020329 |
| Soricomorpha | Soricidae | Crocidura | rhoditis | Crocidura rhoditis | NA | NA | 97134.65 | 222.87 | 20.896 | NA | NA | NA | NA | NA | 0.01789 |
| Rodentia | Sciuridae | Microsciurus | alfari | Microsciurus alfari | 87.49 | NA | 97137.79 | 231.32 | 24.945 | 3 | NA | 2 | 2 | NA | 0.011846 |
| Rodentia | Muridae | Rattus | mordax | Rattus mordax | 230 | 2.47 | 97464.01 | 223.18 | 20.883 | NA | 2 | NA | NA | 1 | 0.016589 |
| Soricomorpha | Soricidae | Sorex | shinto | Sorex shinto | NA | NA | 97473.52 | 110.41 | 5.595 | NA | NA | NA | NA | NA | 0.018903 |
| Chiroptera | Pteropodidae | Syconycteris | hobbit | Syconycteris hobbit | 20.06 | 0.98 | 97642.01 | 315 | 18.627 | NA | 1 | 1 | 2 | 1 | 0.943522 |
| Afrosoricida | Tenrecidae | Hemicentetes | semispinosus | Hemicentetes semispinosus | 134 | 5.32 | 97855.43 | 175.17 | 19.899 | 2 | 1 | 2 | 1 | 3 | 0.02357 |
| Soricomorpha | Soricidae | Crocidura | arabica | Crocidura arabica | NA | NA | 97952.06 | 5.22 | 23.522 | NA | NA | NA | NA | NA | 0.01789 |
| Artiodactyla | Suidae | Sus | verrucosus | Sus verrucosus | 89406.23 | 5.47 | 97964.63 | 190.85 | 24.283 | NA | 3 | NA | NA | 2 | 1.388796 |
| Primates | Pitheciidae | Callicebus | hoffmannsi | Callicebus hoffmannsi | 1067.61 | 1.02 | 98076.46 | 158.41 | 25.818 | 3 | NA | 1 | 2 | NA | 0.012669 |
| Chiroptera | Vespertilionidae | Hypsugo | macrotis | Hypsugo macrotis | NA | NA | 98079.15 | 235.27 | 24.56 | NA | NA | 1 | 2 | NA | 0.28224 |
| Chiroptera | Hipposideridae | Hipposideros | wollastoni | Hipposideros wollastoni | 6.93 | NA | 98200.95 | 267.81 | 24.103 | NA | NA | 1 | 2 | NA | 0.018372 |
| Soricomorpha | Soricidae | Myosorex | tenuis | Myosorex tenuis | NA | NA | 98496.49 | 65.36 | 17.195 | NA | NA | NA | NA | NA | 0.324734 |
| Chiroptera | Vespertilionidae | Kerivoula | minuta | Kerivoula minuta | 2.03 | NA | 98737.55 | 207.47 | 25.035 | NA | NA | 1 | 2 | NA | 0.15685 |
| Rodentia | Muridae | Hyomys | goliath | Hyomys goliath | 866.74 | 1 | 99063.72 | 287 | 18.72 | 1 | 2 | 3 | 2 | 1 | 0.019459 |
| Rodentia | Sciuridae | Tamias | senex | Tamias senex | 89.35 | 4.37 | 99452.58 | 54.63 | 7.764 | 3 | 2 | 2 | 2 | 2 | 0.013003 |
| Rodentia | Heteromyidae | Chaetodipus | pernix | Chaetodipus pernix | 17 | NA | 99496.23 | 38.96 | 21.241 | NA | 1 | NA | NA | 1 | 0.01835 |
| Rodentia | Muridae | Thamnomys | venustus | Thamnomys venustus | 61.88 | 1.33 | 99705.53 | 112.45 | 19.626 | NA | NA | NA | NA | NA | 0.297025 |
| Rodentia | Sciuridae | Sundasciurus | philippinensis | Sundasciurus philippinensis | NA | NA | 99812.67 | 169.61 | 23.866 | NA | NA | NA | NA | NA | 0.020635 |
| Rodentia | Sciuridae | Callosciurus | phayrei | Callosciurus phayrei | NA | NA | 99941.58 | 126.01 | 17.82 | NA | NA | NA | NA | NA | 0.020242 |
| Lagomorpha | Leporidae | Lepus | starcki | Lepus starcki | 2760.67 | NA | 100134.9 | 82.63 | 11.881 | NA | NA | 1 | 1 | NA | 0.015443 |
| Rodentia | Muridae | Mus | bufo | Mus bufo | 8.88 | NA | 100286.3 | 105.95 | 19.38 | NA | NA | NA | NA | NA | 0.015632 |
| Rodentia | Heteromyidae | Chaetodipus | rudinoris | Chaetodipus rudinoris | NA | NA | 100302.5 | 12.01 | 17.957 | NA | NA | NA | NA | NA | 0.017113 |
| Afrosoricida | Tenrecidae | Microgale | dobsoni | Microgale dobsoni | 36.66 | 2.66 | 100500.7 | 178.65 | 19.592 | NA | 1 | 1 | 1 | 3 | 0.021013 |
| Rodentia | Cricetidae | Neofiber | alleni | Neofiber alleni | 265.38 | 2.33 | 100572.2 | 111.09 | 21.869 | 1 | 3 | 2 | 1 | 1 | 0.02105 |
| Chiroptera | Vespertilionidae | Myotis | fimbriatus | Myotis fimbriatus | NA | NA | 100729 | 114.1 | 17.279 | NA | NA | NA | NA | NA | 0.013033 |
| Chiroptera | Phyllostomidae | Lonchophylla | dekeyseri | Lonchophylla dekeyseri | NA | NA | 101542.3 | 123.36 | 20.79 | NA | 3 | 1 | 2 | 2 | 0.176312 |
| Rodentia | Sciuridae | Paraxerus | cooperi | Paraxerus cooperi | 250 | NA | 101664 | 169.35 | 23.416 | NA | NA | NA | NA | NA | 0.283545 |
| Soricomorpha | Talpidae | Talpa | caeca | Talpa caeca | 71.08 | NA | 101761.8 | 76.88 | 9.466 | 2 | 1 | 2 | 1 | 3 | 0.023985 |
| Diprotodontia | Pseudocheiridae | Pseudochirulus | canescens | Pseudochirulus canescens | 300 | 2.57 | 101962.2 | 282.76 | 20.384 | NA | 2 | 1 | 2 | 1 | 0.021648 |
| Rodentia | Cricetidae | Oecomys | phaeotis | Oecomys phaeotis | 73.4 | NA | 102039.1 | 207.59 | 20.18 | NA | NA | NA | NA | NA | 0.012792 |
| Rodentia | Calomyscidae | Calomyscus | bailwardi | Calomyscus bailwardi | 21.38 | 4 | 102086.1 | 24.52 | 13.014 | NA | NA | NA | NA | NA | 0.018006 |
| Rodentia | Muridae | Crunomys | melanius | Crunomys melanius | NA | NA | 102114.9 | 174.05 | 23.838 | NA | NA | NA | NA | NA | 0.59153 |
| Soricomorpha | Soricidae | Crocidura | pitmani | Crocidura pitmani | NA | NA | 102228 | 81.17 | 21.221 | NA | NA | NA | NA | NA | 0.286234 |
| Rodentia | Sciuridae | Sciurus | oculatus | Sciurus oculatus | 649.98 | NA | 102332.9 | 73.27 | 18.091 | 3 | NA | 2 | 2 | NA | 0.009518 |
| Primates | Cercopithecidae | Cercopithecus | erythrogaster | Cercopithecus erythrogaster | 3444.88 | 1.02 | 102343.8 | 137 | 26.319 | 3 | 2 | 1 | 2 | 2 | 0.81357 |
| Rodentia | Muridae | Batomys | salomonseni | Batomys salomonseni | 185.76 | NA | 102368.1 | 175.7 | 23.887 | NA | NA | NA | NA | NA | 0.01769 |
| Rodentia | Cricetidae | Myodes | andersoni | Myodes andersoni | NA | NA | 102632.1 | 135.08 | 8.124 | NA | NA | NA | NA | NA | 0.01504 |
| Rodentia | Octodontidae | Octodon | bridgesi | Octodon bridgesi | 162.25 | NA | 102723.4 | 60.22 | 7.688 | 3 | 3 | 1 | 1 | 1 | 1.037517 |
| Chiroptera | Pteropodidae | Ptenochirus | minor | Ptenochirus minor | 47.02 | NA | 103200.3 | 175.7 | 23.887 | NA | NA | 1 | 2 | NA | 0.015563 |
| Rodentia | Cricetidae | Oryzomys | keaysi | Oryzomys keaysi | 58.29 | NA | 103630.8 | 157.98 | 15.643 | NA | NA | NA | NA | NA | 0.01522 |
| Primates | Pitheciidae | Cacajao | calvus | Cacajao calvus | 3421.04 | 1.01 | 103819 | 211.86 | 25.526 | 3 | 4 | 1 | 2 | 2 | 0.71568 |
| Chiroptera | Pteropodidae | Myonycteris | relicta | Myonycteris relicta | 53.32 | NA | 104213.4 | 85.47 | 23.906 | NA | NA | 1 | 2 | NA | 0.798818 |
| Rodentia | Sciuridae | Sciurus | sanborni | Sciurus sanborni | 136 | NA | 104684 | 309.04 | 19.493 | 3 | NA | 2 | 2 | NA | 0.170783 |
| Rodentia | Cricetidae | Mesocricetus | newtoni | Mesocricetus newtoni | 97.5 | NA | 104825.1 | 49.08 | 10.043 | NA | NA | NA | NA | NA | 0.204342 |
| Rodentia | Cricetidae | Tylomys | watsoni | Tylomys watsoni | NA | 2.91 | 104864.3 | 220.68 | 25.039 | NA | NA | NA | NA | NA | 0.01959 |
| Dasyuromorphia | Dasyuridae | Sminthopsis | leucopus | Sminthopsis leucopus | 23.36 | 8.4 | 105026.4 | 66.89 | 11.607 | 2 | NA | 1 | 1 | NA | 0.848537 |
| Primates | Cheirogaleidae | Microcebus | rufus | Microcebus rufus | 48.39 | 2.52 | 105086.8 | 171.44 | 19.961 | 1 | 5 | 1 | 2 | 2 | 0.020342 |
| Artiodactyla | Bovidae | Naemorhedus | baileyi | Naemorhedus baileyi | 28863.45 | 1.5 | 105198.5 | 173.39 | 5.305 | NA | NA | NA | NA | NA | 0.642872 |
| Chiroptera | Pteropodidae | Harpyionycteris | celebensis | Harpyionycteris celebensis | 116.77 | 0.98 | 105330.6 | 224.32 | 20.495 | NA | NA | 1 | 2 | NA | 0.943395 |
| Soricomorpha | Talpidae | Mogera | imaizumii | Mogera imaizumii | 65.53 | 3.25 | 105817.7 | 141.9 | 8.795 | 2 | 2 | 1 | 1 | 2 | 0.020942 |
| Didelphimorphia | Didelphidae | Gracilinanus | dryas | Gracilinanus dryas | 18.53 | NA | 105840.9 | 108.13 | 20.744 | 1 | NA | 2 | 2 | NA | 0.229938 |
| Chiroptera | Vespertilionidae | Harpiocephalus | mordax | Harpiocephalus mordax | NA | NA | 105934.5 | 136.13 | 22.757 | NA | NA | NA | NA | NA | 0.239956 |
| Rodentia | Muridae | Mesembriomys | macrurus | Mesembriomys macrurus | 294.28 | 2 | 106059.1 | 85.75 | 27.415 | NA | NA | NA | NA | NA | 0.01682 |
| Rodentia | Muridae | Rattus | marmosurus | Rattus marmosurus | NA | NA | 106195.3 | 226.99 | 20.936 | NA | NA | NA | NA | NA | 0.016603 |
| Rodentia | Dipodidae | Sicista | caudata | Sicista caudata | NA | NA | 106216.8 | 54.05 | -2.075 | NA | NA | NA | NA | NA | 0.374873 |
| Rodentia | Ctenomyidae | Ctenomys | magellanicus | Ctenomys magellanicus | 271.44 | NA | 106225.8 | 49.16 | 4.717 | NA | 3 | 2 | 1 | 1 | 0.756931 |
| Rodentia | Dipodidae | Stylodipus | sungorus | Stylodipus sungorus | NA | NA | 106238.2 | 12.05 | 1.277 | 1 | 3 | 2 | 1 | 1 | 0.01888 |
| Primates | Cercopithecidae | Mandrillus | leucophaeus | Mandrillus leucophaeus | 14253.3 | 1.01 | 106294.3 | 203.29 | 24.278 | 3 | 3 | 1 | 1 | 2 | 0.988636 |
| Rodentia | Muridae | Muriculus | imberbis | Muriculus imberbis | 18.5 | NA | 106402.3 | 97.68 | 16.308 | NA | NA | NA | NA | NA | 0.023685 |
| Soricomorpha | Soricidae | Crocidura | beatus | Crocidura beatus | 10.55 | NA | 106437.4 | 173.61 | 24.008 | NA | NA | NA | NA | NA | 0.01789 |
| Carnivora | Eupleridae | Fossa | fossana | Fossa fossana | 1853.98 | 1 | 106522.1 | 168.03 | 20.281 | 1 | 6 | 2 | 2 | 2 | 0.223478 |
| Soricomorpha | Talpidae | Euroscaptor | klossi | Euroscaptor klossi | NA | 4.5 | 106925 | 139.25 | 21.758 | NA | 1 | 2 | 1 | 3 | 0.021789 |
| Rodentia | Muridae | Apomys | littoralis | Apomys littoralis | 30.95 | NA | 106985.3 | 173.61 | 24.008 | 1 | NA | 2 | 2 | NA | 0.211549 |
| Chiroptera | Vespertilionidae | Lasiurus | insularis | Lasiurus insularis | NA | NA | 107157.2 | 103.5 | 25.234 | NA | NA | NA | NA | NA | 0.763118 |
| Chiroptera | Vespertilionidae | Lasiurus | pfeifferi | Lasiurus pfeifferi | NA | NA | 107157.2 | 103.5 | 25.234 | NA | NA | NA | NA | NA | 0.142579 |
| Chiroptera | Molossidae | Mormopterus | minutus | Mormopterus minutus | NA | NA | 107157.2 | 103.5 | 25.234 | NA | NA | 1 | 2 | NA | 0.868281 |
| Chiroptera | Vespertilionidae | Nycticeius | cubanus | Nycticeius cubanus | NA | NA | 107157.2 | 103.5 | 25.234 | NA | NA | NA | NA | NA | 0.195117 |
| Rodentia | Muridae | Macruromys | major | Macruromys major | 357.25 | NA | 107183.1 | 304.67 | 20.051 | 1 | 2 | 1 | 1 | 1 | 0.019459 |
| Rodentia | Hystricidae | Hystrix | crassispinis | Hystrix crassispinis | 4584.6 | NA | 107350.3 | 262.97 | 24.793 | NA | 6 | 2 | 1 | 2 | 0.019685 |
| Rodentia | Muridae | Dipodillus | mackilligini | Dipodillus mackilligini | NA | NA | 107415 | 0.05 | 24.205 | NA | NA | NA | NA | NA | 0.022232 |
| Diprotodontia | Pseudocheiridae | Pseudochirulus | larvatus | Pseudochirulus larvatus | NA | NA | 107678.2 | 344.76 | 18.887 | NA | NA | NA | NA | NA | 0.01893 |
| Primates | Hominidae | Gorilla | beringei | Gorilla beringei | 149325.2 | NA | 107960.3 | 122.55 | 20.649 | NA | NA | NA | NA | NA | 1.305142 |
| Chiroptera | Pteropodidae | Pteropus | leucopterus | Pteropus leucopterus | 343.81 | NA | 107982.2 | 238.29 | 24.072 | NA | NA | 1 | 2 | NA | 0.015175 |
| Rodentia | Cricetidae | Calomys | boliviae | Calomys boliviae | 27 | NA | 108148.2 | 59.05 | 13.445 | NA | NA | NA | NA | NA | 0.016197 |
| Artiodactyla | Cervidae | Muntiacus | atherodes | Muntiacus atherodes | 18939.1 | NA | 108466.5 | 255.41 | 23.632 | 2 | 2 | NA | NA | 1 | 0.015064 |
| Rodentia | Cricetidae | Necromys | temchuki | Necromys temchuki | 47.2 | NA | 108564.1 | 105.35 | 21.082 | NA | NA | NA | NA | NA | 0.012975 |
| Artiodactyla | Suidae | Sus | ahoenobarbus | Sus ahoenobarbus | NA | NA | 108626.4 | 177.32 | 23.806 | NA | NA | NA | NA | NA | 0.864404 |
| Rodentia | Sciuridae | Petinomys | vordermanni | Petinomys vordermanni | 36.87 | 2 | 108682.4 | 238.78 | 23.756 | 1 | 4 | NA | NA | 2 | 0.759408 |
| Dasyuromorphia | Dasyuridae | Dasyuroides | byrnei | Dasyuroides byrnei | 109.47 | 5.14 | 108699 | 20.57 | 22.707 | 2 | 2 | 1 | 1 | 3 | 0.78896 |
| Rodentia | Ctenomyidae | Ctenomys | minutus | Ctenomys minutus | 92 | NA | 108713.5 | 111.66 | 25.64 | NA | 3 | 2 | 1 | 1 | 0.242218 |
| Rodentia | Cricetidae | Tylomys | mirae | Tylomys mirae | 183.6 | NA | 108833 | 142.95 | 19.693 | 1 | 1 | 1 | 2 | 3 | 0.01959 |
| Afrosoricida | Tenrecidae | Microgale | parvula | Microgale parvula | 3.53 | NA | 109243.5 | 170.29 | 20.001 | NA | NA | 2 | 2 | NA | 0.021013 |
| Rodentia | Capromyidae | Capromys | pilorides | Capromys pilorides | 5200 | 2 | 109410.9 | 103.29 | 25.228 | NA | 3 | 1 | 2 | 2 | 0.015765 |
| Dasyuromorphia | Dasyuridae | Antechinus | minimus | Antechinus minimus | 52.56 | 7.19 | 109624.7 | 66.04 | 11.769 | 2 | NA | 1 | 1 | NA | 0.016758 |
| Paucituberculata | Caenolestidae | Lestoros | inca | Lestoros inca | 23.07 | NA | 109634.3 | 235.43 | 15.408 | 2 | 2 | 1 | 1 | 3 | 0.026019 |
| Soricomorpha | Soricidae | Crocidura | tarella | Crocidura tarella | NA | NA | 109789.1 | 103.16 | 23.189 | NA | NA | NA | NA | NA | 1.34172 |
| Rodentia | Muridae | Mastacomys | fuscus | Mastacomys fuscus | 125.72 | 1.41 | 110002.9 | 66.31 | 11.644 | NA | NA | NA | NA | NA | 0.216495 |
| Rodentia | Cricetidae | Thomasomys | daphne | Thomasomys daphne | 77 | NA | 110024.4 | 180.42 | 15.758 | NA | NA | NA | NA | NA | 0.014369 |
| Rodentia | Erethizontidae | Chaetomys | subspinosus | Chaetomys subspinosus | 1299.99 | NA | 110201.6 | 127.76 | 22.764 | 1 | 3 | 1 | 2 | 1 | 1.033121 |
| Rodentia | Muridae | Paramelomys | naso | Paramelomys naso | NA | NA | 110306.3 | 195.44 | 24.538 | NA | NA | NA | NA | NA | 0.016916 |
| Rodentia | Dipodidae | Salpingotulus | michaelis | Salpingotulus michaelis | NA | 3 | 110350.8 | 9.1 | 18.962 | 1 | 4 | 2 | 1 | 1 | 0.352001 |
| Artiodactyla | Bovidae | Capra | pyrenaica | Capra pyrenaica | 60898.77 | 1.5 | 110472.5 | 42.64 | 13.193 | NA | 2 | NA | NA | 1 | 0.015431 |
| Rodentia | Muridae | Mallomys | aroaensis | Mallomys aroaensis | 1663.99 | 1.02 | 110553.7 | 298.45 | 18.33 | NA | 1 | 3 | 2 | 1 | 0.018626 |
| Rodentia | Muridae | Taeromys | callitrichus | Taeromys callitrichus | NA | NA | 110913.1 | 227.89 | 21.251 | NA | NA | NA | NA | NA | 0.281128 |
| Rodentia | Muridae | Taeromys | celebensis | Taeromys celebensis | NA | NA | 110913.1 | 227.89 | 21.251 | NA | NA | NA | NA | NA | 0.019786 |
| Chiroptera | Pteropodidae | Aethalops | alecto | Aethalops alecto | 15 | 0.99 | 110914.6 | 250.35 | 24.264 | NA | NA | 1 | 2 | NA | 0.017299 |
| Rodentia | Cricetidae | Chionomys | gud | Chionomys gud | NA | 5.83 | 110928.2 | 62.87 | 2.955 | NA | NA | NA | NA | NA | 0.018112 |
| Diprotodontia | Macropodidae | Dendrolagus | goodfellowi | Dendrolagus goodfellowi | 7948.78 | 1 | 111020.2 | 293.65 | 19.28 | 2 | 2 | 2 | 2 | 1 | 0.978139 |
| Rodentia | Cricetidae | Myodes | shanseius | Myodes shanseius | NA | NA | 111286.4 | 36.57 | 4.766 | NA | NA | NA | NA | NA | 0.013 |
| Soricomorpha | Soricidae | Crocidura | erica | Crocidura erica | NA | NA | 111398.2 | 103.73 | 20.066 | NA | NA | NA | NA | NA | 0.286234 |
| Carnivora | Eupleridae | Eupleres | goudotii | Eupleres goudotii | 2763.34 | 1 | 111808.3 | 163.67 | 20.715 | 2 | 6 | 1 | 1 | 2 | 0.223478 |
| Rodentia | Cricetidae | Otonyctomys | hatti | Otonyctomys hatti | 36.2 | NA | 111895.3 | 97.1 | 25.404 | NA | NA | NA | NA | NA | 0.020103 |
| Carnivora | Procyonidae | Nasuella | olivacea | Nasuella olivacea | 1339.99 | NA | 111926.5 | 118.71 | 17.703 | 2 | 6 | 1 | 1 | 2 | 0.3054 |
| Chiroptera | Pteropodidae | Pteropus | lylei | Pteropus lylei | 319.75 | NA | 111952.4 | 167.45 | 25.948 | NA | 1 | 1 | 2 | 1 | 0.737747 |
| Artiodactyla | Cervidae | Mazama | chunyi | Mazama chunyi | 16071.74 | 1.5 | 112162.1 | 205.34 | 17.489 | NA | NA | 1 | 1 | NA | 0.39668 |
| Rodentia | Muridae | Gerbilliscus | phillipsi | Gerbilliscus phillipsi | 127.07 | NA | 112212.2 | 56.21 | 17.064 | NA | NA | NA | NA | NA | 0.01967 |
| Rodentia | Cricetidae | Phyllotis | andium | Phyllotis andium | 53 | NA | 112900.2 | 64.59 | 14.391 | NA | NA | NA | NA | NA | 0.016023 |
| Rodentia | Muridae | Gerbillurus | setzeri | Gerbillurus setzeri | 38.29 | NA | 112926.9 | 2.92 | 14.059 | NA | 3 | NA | NA | 2 | 0.017972 |
| Primates | Cheirogaleidae | Cheirogaleus | major | Cheirogaleus major | 446.02 | 2.26 | 112950.3 | 167.63 | 20.298 | 1 | 5 | 1 | 2 | 2 | 0.021785 |
| Primates | Hylobatidae | Symphalangus | syndactylus | Symphalangus syndactylus | 10839 | 1.02 | 113063.1 | 226.72 | 24.168 | 3 | 4 | NA | NA | 2 | 1.253062 |
| Diprotodontia | Phalangeridae | Spilocuscus | rufoniger | Spilocuscus rufoniger | 5999.99 | 1.02 | 113157.5 | 252.11 | 26.015 | 2 | NA | NA | NA | NA | 1.73917 |
| Artiodactyla | Bovidae | Capricornis | crispus | Capricornis crispus | 43030.64 | 1 | 113318.5 | 144.41 | 9.381 | NA | NA | NA | NA | NA | 0.012857 |
| Chiroptera | Phyllostomidae | Mimon | koepckeae | Mimon koepckeae | NA | NA | 113389.5 | 115.61 | 17.104 | NA | NA | NA | NA | NA | 0.27039 |
| Primates | Pitheciidae | Callicebus | cinerascens | Callicebus cinerascens | 992.4 | 1.02 | 113467.5 | 180.52 | 24.844 | 3 | NA | 1 | 2 | NA | 0.013031 |
| Chiroptera | Pteropodidae | Styloctenium | wallacei | Styloctenium wallacei | 172.42 | NA | 113590.5 | 227.03 | 20.903 | NA | NA | 1 | 2 | NA | 0.909516 |
| Primates | Pitheciidae | Pithecia | aequatorialis | Pithecia aequatorialis | 2375.91 | NA | 113679.1 | 263.1 | 27.105 | 3 | 3 | 1 | 2 | 1 | 0.016757 |
| Chiroptera | Vespertilionidae | Myotis | ater | Myotis ater | NA | NA | 114590.7 | 250.47 | 22.398 | NA | NA | NA | NA | NA | 0.012465 |
| Dasyuromorphia | Dasyuridae | Sminthopsis | gilberti | Sminthopsis gilberti | 19.5 | NA | 114805.1 | 38.94 | 15.74 | 2 | NA | 1 | 1 | NA | 0.016971 |
| Rodentia | Muridae | Grammomys | dryas | Grammomys dryas | NA | NA | 115505.7 | 111.73 | 20.853 | NA | NA | NA | NA | NA | 0.202683 |
| Artiodactyla | Bovidae | Bos | sauveli | Bos sauveli | 791321.8 | NA | 115557.2 | 170.36 | 25.145 | 2 | 2 | 1 | 1 | 1 | 1.366031 |
| Rodentia | Muridae | Apomys | musculus | Apomys musculus | 21.2 | NA | 115585.4 | 233.49 | 24.053 | 2 | 4 | 2 | 2 | 2 | 0.013222 |
| Rodentia | Muridae | Chrotomys | mindorensis | Chrotomys mindorensis | 151.98 | NA | 115585.4 | 233.49 | 24.053 | NA | NA | NA | NA | NA | 0.102874 |
| Rodentia | Muridae | Niviventer | rapit | Niviventer rapit | 79.73 | NA | 115873.3 | 282.45 | 23.918 | NA | NA | NA | NA | NA | 0.017973 |
| Rodentia | Sciuridae | Sciurus | pucheranii | Sciurus pucheranii | 803 | NA | 116015.3 | 148.79 | 18.952 | 3 | NA | 2 | 2 | NA | 0.1928 |
| Soricomorpha | Soricidae | Sylvisorex | lunaris | Sylvisorex lunaris | 18.78 | 2.69 | 116023.1 | 103.68 | 19.277 | NA | NA | NA | NA | NA | 1.014822 |
| Chiroptera | Rhinolophidae | Rhinolophus | guineensis | Rhinolophus guineensis | NA | NA | 116184.3 | 187.1 | 25.396 | NA | NA | 1 | 2 | NA | 0.436052 |
| Rodentia | Heteromyidae | Chaetodipus | californicus | Chaetodipus californicus | 23.01 | 4 | 116215.9 | 34.72 | 11.704 | NA | NA | NA | NA | NA | 0.018479 |
| Soricomorpha | Talpidae | Scapanus | townsendii | Scapanus townsendii | NA | NA | 116660.4 | 111.5 | 8.095 | NA | NA | NA | NA | NA | 0.020748 |
| Soricomorpha | Soricidae | Crocidura | grayi | Crocidura grayi | 10.7 | NA | 117062.8 | 233.78 | 24.062 | NA | 1 | NA | NA | 3 | 0.01789 |
| Rodentia | Cricetidae | Dinaromys | bogdanovi | Dinaromys bogdanovi | 56 | 2.5 | 117073.5 | 85.98 | 7.405 | NA | NA | NA | NA | NA | 1.047604 |
| Rodentia | Sciuridae | Ratufa | macroura | Ratufa macroura | 1326.24 | 3.49 | 117108.4 | 136.29 | 26.431 | NA | 4 | 2 | 2 | 2 | 0.175382 |
| Didelphimorphia | Didelphidae | Marmosops | fuscatus | Marmosops fuscatus | 45.15 | 5.47 | 117128.9 | 99.22 | 21.842 | 1 | 2 | 2 | 2 | 2 | 0.339057 |
| Rodentia | Cricetidae | Akodon | bogotensis | Akodon bogotensis | 12.99 | NA | 117248.5 | 109.88 | 20.166 | NA | NA | NA | NA | NA | 0.012978 |
| Primates | Galagidae | Euoticus | pallidus | Euoticus pallidus | 277.37 | NA | 117645.3 | 192.29 | 24.25 | NA | NA | NA | NA | NA | 0.018999 |
| Rodentia | Muridae | Lenomys | meyeri | Lenomys meyeri | NA | NA | 117714.2 | 223.06 | 21.09 | NA | NA | NA | NA | NA | 0.020141 |
| Rodentia | Muridae | Bunomys | andrewsi | Bunomys andrewsi | NA | NA | 117802.5 | 228.96 | 21.336 | NA | NA | NA | NA | NA | 0.019562 |
| Rodentia | Cricetidae | Bibimys | torresi | Bibimys torresi | 28 | NA | 117884 | 113.47 | 19.737 | NA | NA | NA | NA | NA | 0.119437 |
| Rodentia | Muridae | Stenocephalemys | griseicauda | Stenocephalemys griseicauda | 85.75 | NA | 117886.5 | 88.04 | 13.305 | NA | NA | NA | NA | NA | 0.019357 |
| Rodentia | Muridae | Meriones | arimalius | Meriones arimalius | NA | NA | 117976.6 | 7.3 | 25.494 | NA | NA | NA | NA | NA | 0.018016 |
| Dasyuromorphia | Dasyuridae | Antechinus | bellus | Antechinus bellus | 38.73 | 10.08 | 118056.9 | 94.51 | 26.638 | 2 | NA | 1 | 1 | NA | 0.014798 |
| Chiroptera | Phyllostomidae | Vampyressa | nymphaea | Vampyressa nymphaea | 69 | 0.99 | 118464.3 | 230.23 | 24.961 | NA | NA | 1 | 2 | NA | 0.01533 |
| Rodentia | Sciuridae | Heliosciurus | undulatus | Heliosciurus undulatus | 347.33 | NA | 118631 | 79.16 | 23.36 | NA | NA | NA | NA | NA | 0.277435 |
| Chiroptera | Vespertilionidae | Phoniscus | atrox | Phoniscus atrox | 4.81 | NA | 118977.9 | 201.65 | 25.414 | NA | NA | 1 | 2 | NA | 0.164218 |
| Rodentia | Spalacidae | Eospalax | rothschildi | Eospalax rothschildi | NA | NA | 119023.3 | 65.09 | 11.449 | NA | NA | NA | NA | NA | 0.02021 |
| Chiroptera | Rhinolophidae | Rhinolophus | inops | Rhinolophus inops | 13.65 | NA | 119027.9 | 179.22 | 24.155 | NA | 1 | 1 | 2 | 3 | 0.012207 |
| Rodentia | Cricetidae | Chelemys | megalonyx | Chelemys megalonyx | 50.8 | NA | 119540.4 | 54.92 | 8.114 | NA | NA | 2 | 1 | NA | 0.151042 |
| Rodentia | Muridae | Bullimus | bagobus | Bullimus bagobus | 395.38 | NA | 119550.4 | 183.1 | 24.107 | NA | NA | NA | NA | NA | 0.017571 |
| Artiodactyla | Cervidae | Rucervus | eldii | Rucervus eldii | 95471.61 | 1.01 | 119941.7 | 157.98 | 24.309 | NA | 3 | 1 | 1 | 1 | 1.236876 |
| Rodentia | Muridae | Mus | platythrix | Mus platythrix | 29.08 | 6.93 | 119949.6 | 134.5 | 24.963 | NA | NA | NA | NA | NA | 0.0104 |
| Soricomorpha | Talpidae | Euroscaptor | grandis | Euroscaptor grandis | NA | NA | 120325.7 | 154.94 | 4.34 | NA | 1 | 2 | 1 | 3 | 0.021789 |
| Chiroptera | Mormoopidae | Pteronotus | macleayii | Pteronotus macleayii | NA | NA | 120492.3 | 108.01 | 25.147 | NA | 1 | 1 | 2 | 3 | 0.020245 |
| Rodentia | Sciuridae | Eoglaucomys | fimbriatus | Eoglaucomys fimbriatus | 510 | 3.74 | 120533 | 66.26 | 10.791 | 1 | NA | 1 | 2 | NA | 0.013882 |
| Carnivora | Eupleridae | Galidia | elegans | Galidia elegans | 810 | 1 | 120561.3 | 154.88 | 21.2 | 2 | 6 | 4 | 2 | 2 | 0.021464 |
| Dermoptera | Cynocephalidae | Cynocephalus | volans | Cynocephalus volans | 1250 | 1 | 120788.3 | 183.1 | 24.107 | 2 | 4 | 1 | 2 | 1 | 0.027444 |
| Rodentia | Geomyidae | Geomys | arenarius | Geomys arenarius | 206 | 4.71 | 120898.7 | 24.73 | 16.43 | NA | 3 | 2 | 1 | 1 | 0.153007 |
| Soricomorpha | Soricidae | Sorex | portenkoi | Sorex portenkoi | NA | NA | 121022.1 | 13.88 | -11.477 | NA | NA | NA | NA | NA | 0.293879 |
| Chiroptera | Pteropodidae | Dyacopterus | spadiceus | Dyacopterus spadiceus | 81.1 | NA | 121150.4 | 245.06 | 23.727 | NA | NA | 1 | 2 | NA | 0.155629 |
| Artiodactyla | Moschidae | Moschus | fuscus | Moschus fuscus | 13697.9 | NA | 121178.8 | 163.32 | 5.758 | 2 | 2 | NA | NA | 1 | 1.006989 |
| Artiodactyla | Cervidae | Hippocamelus | bisulcus | Hippocamelus bisulcus | 69240.81 | 1 | 121225 | 143.42 | 3.495 | 2 | 2 | 1 | 1 | 1 | 0.87744 |
| Rodentia | Sciuridae | Exilisciurus | concinnus | Exilisciurus concinnus | 27.72 | 1.94 | 121262.3 | 183.1 | 24.107 | NA | NA | NA | NA | NA | 0.020337 |
| Primates | Tarsiidae | Tarsius | syrichta | Tarsius syrichta | 115.91 | 1.01 | 121292 | 183.1 | 24.107 | 1 | 1 | NA | NA | 1 | 0.215908 |
| Rodentia | Sciuridae | Microsciurus | mimulus | Microsciurus mimulus | 120 | NA | 121472.5 | 274.1 | 24.619 | 3 | 1 | 2 | 2 | 3 | 0.00982 |
| Rodentia | Muridae | Lophuromys | luteogaster | Lophuromys luteogaster | NA | 1 | 121605.5 | 139.9 | 22.367 | NA | NA | NA | NA | NA | 0.014783 |
| Rodentia | Geomyidae | Thomomys | townsendii | Thomomys townsendii | 263.35 | 6.15 | 121622.1 | 25.75 | 6.859 | NA | 3 | 2 | 1 | 1 | 0.019214 |
| Rodentia | Geomyidae | Cratogeomys | goldmani | Cratogeomys goldmani | NA | NA | 121756.6 | 30.61 | 16.325 | NA | NA | NA | NA | NA | 0.015395 |
| Rodentia | Cricetidae | Eothenomys | olitor | Eothenomys olitor | NA | NA | 121816.2 | 114.53 | 9.819 | NA | NA | NA | NA | NA | 0.013 |
| Primates | Cheirogaleidae | Microcebus | murinus | Microcebus murinus | 69 | 2 | 122051.8 | 85.12 | 24.086 | 1 | 5 | 1 | 2 | 2 | 0.020293 |
| Chiroptera | Molossidae | Mormopterus | norfolkensis | Mormopterus norfolkensis | 8 | 1 | 122196.5 | 83.81 | 16.08 | NA | NA | 1 | 2 | NA | 0.909676 |
| Chiroptera | Pteropodidae | Nyctimene | certans | Nyctimene certans | 43.24 | NA | 122392.3 | 278.65 | 18.501 | NA | NA | 1 | 2 | NA | 0.014644 |
| Rodentia | Sciuridae | Aeromys | thomasi | Aeromys thomasi | 1430.19 | NA | 122621.6 | 285.05 | 24.311 | 1 | NA | NA | NA | NA | 0.22395 |
| Primates | Cheirogaleidae | Cheirogaleus | medius | Cheirogaleus medius | 196.76 | 2.04 | 122848.9 | 85.08 | 24.048 | 1 | 5 | 1 | 2 | 2 | 0.021852 |
| Primates | Cebidae | Callithrix | argentata | Callithrix argentata | 382.92 | 1.84 | 123514 | 171.85 | 25.442 | 3 | 4 | 1 | 2 | 2 | 0.010941 |
| Rodentia | Muridae | Hybomys | planifrons | Hybomys planifrons | 49.6 | NA | 123523.7 | 230.62 | 24.806 | NA | NA | NA | NA | NA | 0.018217 |
| Chiroptera | Phyllostomidae | Erophylla | sezekorni | Erophylla sezekorni | 15.87 | 1 | 123555.6 | 108.01 | 25.147 | NA | NA | 1 | 2 | NA | 0.016863 |
| Rodentia | Nesomyidae | Beamys | hindei | Beamys hindei | 75.8 | 3.49 | 123659.9 | 85.18 | 24.704 | NA | NA | NA | NA | NA | 0.023294 |
| Chiroptera | Rhinolophidae | Rhinolophus | maclaudi | Rhinolophus maclaudi | NA | 0.98 | 123994.4 | 182.55 | 25.793 | NA | NA | 1 | 2 | NA | 0.705606 |
| Chiroptera | Vespertilionidae | Rhogeessa | aeneus | Rhogeessa aeneus | NA | NA | 124920 | 92 | 25.451 | NA | NA | NA | NA | NA | 0.013634 |
| Rodentia | Cricetidae | Microtus | daghestanicus | Microtus daghestanicus | NA | NA | 125293.7 | 44.25 | 3.597 | NA | NA | NA | NA | NA | 0.2223 |
| Carnivora | Herpestidae | Liberiictis | kuhni | Liberiictis kuhni | 1825.47 | NA | 125428.7 | 240.02 | 24.482 | 3 | 6 | 1 | 1 | 2 | 1.012772 |
| Rodentia | Muridae | Nesokia | bunnii | Nesokia bunnii | NA | NA | 125600.9 | 13.8 | 23.402 | NA | NA | NA | NA | NA | 1.510595 |
| Diprotodontia | Potoroidae | Potorous | tridactylus | Potorous tridactylus | 1054.67 | 1 | 125855 | 76.57 | 13.669 | 1 | NA | NA | NA | NA | 0.019494 |
| Rodentia | Cricetidae | Peromyscus | spicilegus | Peromyscus spicilegus | 35.99 | NA | 126047.3 | 61.33 | 17.399 | 1 | NA | NA | NA | NA | 0.017825 |
| Rodentia | Cricetidae | Abrawayaomys | ruschii | Abrawayaomys ruschii | 62.99 | NA | 126137.5 | 107.06 | 20.91 | NA | NA | NA | NA | NA | 0.019815 |
| Rodentia | Heteromyidae | Chaetodipus | spinatus | Chaetodipus spinatus | 16.34 | 3.5 | 126559.6 | 12.35 | 17.758 | NA | NA | NA | NA | NA | 0.018431 |
| Rodentia | Muridae | Acomys | subspinosus | Acomys subspinosus | 21.92 | NA | 126595.8 | 30.22 | 15.589 | 1 | NA | NA | NA | NA | 0.013851 |
| Chiroptera | Vespertilionidae | Falsistrellus | mordax | Falsistrellus mordax | NA | NA | 126776.2 | 195.11 | 24.551 | NA | NA | 1 | 2 | NA | 0.284934 |
| Chiroptera | Molossidae | Otomops | formosus | Otomops formosus | NA | NA | 126776.2 | 195.11 | 24.551 | NA | 1 | 1 | 2 | 3 | 0.294193 |
| Rodentia | Sciuridae | Petinomys | sagitta | Petinomys sagitta | 49.99 | NA | 126776.2 | 195.11 | 24.551 | 1 | NA | NA | NA | NA | 0.243011 |
| Rodentia | Gliridae | Graphiurus | monardi | Graphiurus monardi | NA | NA | 126848.5 | 105.35 | 21.448 | NA | NA | NA | NA | NA | 0.31765 |
| Rodentia | Cricetidae | Microtus | clarkei | Microtus clarkei | NA | NA | 126895.2 | 120.22 | 9.693 | NA | NA | NA | NA | NA | 0.017879 |
| Diprotodontia | Macropodidae | Dendrolagus | inustus | Dendrolagus inustus | 12625 | 1.11 | 126998.3 | 216.33 | 25.998 | 2 | 2 | 2 | 2 | 1 | 0.787818 |
| Rodentia | Cricetidae | Oryzomys | saturatior | Oryzomys saturatior | NA | NA | 127187.9 | 132.74 | 22.117 | NA | NA | NA | NA | NA | 0.152198 |
| Paucituberculata | Caenolestidae | Caenolestes | fuliginosus | Caenolestes fuliginosus | 28.64 | 3.62 | 127434.1 | 103.05 | 18.816 | 2 | 4 | 1 | 1 | 2 | 0.024211 |
| Rodentia | Cricetidae | Oryzomys | levipes | Oryzomys levipes | 60.5 | NA | 127740.8 | 151.2 | 13.365 | NA | NA | NA | NA | NA | 0.01522 |
| Macroscelidea | Macroscelididae | Elephantulus | revoili | Elephantulus revoili | 32.49 | NA | 127810 | 18.06 | 24.32 | NA | NA | NA | NA | NA | 0.302111 |
| Rodentia | Muridae | Pogonomys | sylvestris | Pogonomys sylvestris | 41.67 | 2.64 | 128124 | 276.55 | 18.829 | 1 | 2 | 3 | 2 | 1 | 0.019355 |
| Artiodactyla | Cervidae | Muntiacus | crinifrons | Muntiacus crinifrons | 18590.01 | 1 | 128303.4 | 117.9 | 14.2 | NA | 4 | NA | NA | 2 | 0.753217 |
| Chiroptera | Phyllostomidae | Lonchophylla | bokermanni | Lonchophylla bokermanni | NA | NA | 128491.5 | 120.37 | 20.147 | NA | 3 | 1 | 2 | 2 | 0.282099 |
| Soricomorpha | Soricidae | Crocidura | manengubae | Crocidura manengubae | NA | NA | 128796.1 | 143.9 | 23.425 | NA | NA | NA | NA | NA | 0.89448 |
| Rodentia | Muridae | Otomys | laminatus | Otomys laminatus | 150 | NA | 128843.8 | 70.61 | 17.251 | NA | NA | NA | NA | NA | 0.019294 |
| Chiroptera | Myzopodidae | Myzopoda | aurita | Myzopoda aurita | 9.1 | NA | 129074.2 | 177.94 | 20.13 | NA | 1 | 1 | 2 | 3 | 0.028545 |
| Rodentia | Sciuridae | Heliosciurus | ruwenzorii | Heliosciurus ruwenzorii | 291 | 2.91 | 129123.3 | 113.81 | 20.124 | NA | NA | NA | NA | NA | 0.01734 |
| Rodentia | Heteromyidae | Perognathus | amplus | Perognathus amplus | 11.97 | NA | 129256.1 | 20.84 | 17.726 | NA | NA | NA | NA | NA | 0.020442 |
| Chiroptera | Pteropodidae | Megaerops | wetmorei | Megaerops wetmorei | 18.7 | NA | 129280.2 | 253.22 | 23.481 | NA | NA | 1 | 2 | NA | 0.820417 |
| Primates | Cercopithecidae | Pygathrix | nigripes | Pygathrix nigripes | 10333.32 | 1 | 129519.6 | 158.37 | 24.634 | NA | 2 | NA | NA | 1 | 1.011098 |
| Primates | Cebidae | Callithrix | humeralifera | Callithrix humeralifera | 374.99 | NA | 129628 | 163.67 | 25.866 | 3 | 4 | 1 | 2 | 2 | 0.176416 |
| Primates | Atelidae | Lagothrix | lugens | Lagothrix lugens | NA | NA | 129980.9 | 117.3 | 20.631 | NA | NA | NA | NA | NA | 1.62864 |
| Artiodactyla | Bovidae | Hemitragus | jemlahicus | Hemitragus jemlahicus | 68616.43 | 1.01 | 130100.3 | 121.77 | 5.431 | NA | 2 | NA | NA | 1 | 0.140569 |
| Primates | Cercopithecidae | Presbytis | melalophos | Presbytis melalophos | 6439.12 | 1.01 | 130402.6 | 278.5 | 24.703 | 3 | 1 | NA | NA | 1 | 0.925884 |
| Chiroptera | Molossidae | Molossops | aequatorianus | Molossops aequatorianus | NA | NA | 130439.1 | 148.35 | 19.996 | NA | 1 | 1 | 2 | 3 | 0.988618 |
| Chiroptera | Emballonuridae | Emballonura | atrata | Emballonura atrata | 4.55 | NA | 130634.9 | 141.88 | 21.189 | 2 | 1 | 1 | 2 | 3 | 0.023995 |
| Rodentia | Cricetidae | Peromyscus | yucatanicus | Peromyscus yucatanicus | 27.13 | 3 | 130695.7 | 94.84 | 25.462 | 1 | NA | NA | NA | NA | 0.017825 |
| Scandentia | Tupaiidae | Tupaia | picta | Tupaia picta | 168.05 | NA | 130848.1 | 261.79 | 23.726 | 3 | 4 | NA | NA | 2 | 0.023474 |
| Primates | Cercopithecidae | Procolobus | verus | Procolobus verus | 3977.86 | 1.02 | 130901.2 | 186.89 | 25.288 | 3 | 1 | NA | NA | 1 | 0.173116 |
| Rodentia | Cricetidae | Phyllotis | darwini | Phyllotis darwini | 50.82 | 4.02 | 130933.1 | 29.86 | 8.992 | 2 | 3 | NA | NA | 2 | 0.013379 |
| Artiodactyla | Bovidae | Cephalophus | jentinki | Cephalophus jentinki | 68489.54 | 1 | 130952.1 | 210.72 | 25.15 | 1 | 4 | NA | NA | 1 | 1.07927 |
| Chiroptera | Vespertilionidae | Myotis | sicarius | Myotis sicarius | NA | NA | 131274.3 | 156.61 | 9.628 | NA | NA | 1 | 2 | NA | 0.615124 |
| Rodentia | Sciuridae | Prosciurillus | leucomus | Prosciurillus leucomus | NA | NA | 131517.5 | 229.25 | 21.43 | NA | NA | NA | NA | NA | 0.28023 |
| Monotremata | Tachyglossidae | Zaglossus | bartoni | Zaglossus bartoni | NA | NA | 131753.8 | 277.69 | 18.029 | NA | NA | NA | NA | NA | 2.660959 |
| Chiroptera | Emballonuridae | Taphozous | australis | Taphozous australis | NA | NA | 131852.5 | 124.55 | 23.205 | NA | NA | NA | NA | NA | 0.219059 |
| Rodentia | Spalacidae | Eospalax | smithii | Eospalax smithii | NA | NA | 132161.7 | 44.26 | 6.694 | NA | NA | NA | NA | NA | 0.02021 |
| Artiodactyla | Bovidae | Connochaetes | gnou | Connochaetes gnou | 156547.5 | 1 | 132200.7 | 47.98 | 15.429 | 2 | 2 | 1 | 1 | 1 | 0.017129 |
| Artiodactyla | Cervidae | Rusa | timorensis | Rusa timorensis | 66375.5 | 1 | 132249 | 196.77 | 24.46 | 1 | 2 | NA | NA | 1 | 0.824584 |
| Chiroptera | Nycteridae | Nycteris | javanica | Nycteris javanica | 17.78 | 1 | 132686.3 | 196.77 | 24.46 | NA | 2 | 1 | 2 | 3 | 0.904609 |
| Primates | Cebidae | Callithrix | geoffroyi | Callithrix geoffroyi | 342 | NA | 132745.3 | 108.43 | 21.605 | 3 | NA | 1 | 2 | NA | 0.010157 |
| Chiroptera | Phyllostomidae | Neonycteris | pusilla | Neonycteris pusilla | NA | NA | 132901.2 | 261.74 | 24.803 | NA | 2 | 1 | 2 | 2 | 0.989631 |
| Chiroptera | Hipposideridae | Asellia | patrizii | Asellia patrizii | NA | NA | 132910.2 | 37.13 | 25.516 | NA | NA | 1 | 2 | NA | 0.019192 |
| Chiroptera | Vespertilionidae | Myotis | rosseti | Myotis rosseti | 3.29 | NA | 132994.3 | 141.17 | 26.285 | NA | NA | 1 | 2 | NA | 0.014229 |
| Chiroptera | Hipposideridae | Triaenops | furculus | Triaenops furculus | 5.56 | NA | 133107.8 | 126.86 | 23.705 | 2 | 1 | 1 | 2 | 3 | 0.019659 |
| Rodentia | Muridae | Vernaya | fulva | Vernaya fulva | NA | NA | 133272.2 | 93.48 | 8.391 | NA | NA | NA | NA | NA | 0.023685 |
| Rodentia | Muridae | Aethomys | bocagei | Aethomys bocagei | NA | NA | 133883.7 | 82.98 | 21.757 | NA | NA | NA | NA | NA | 0.021347 |
| Chiroptera | Vespertilionidae | Phoniscus | jagorii | Phoniscus jagorii | 4.7 | 1 | 134207.3 | 240.14 | 24.667 | NA | NA | 1 | 2 | NA | 0.016422 |
| Rodentia | Muridae | Otomys | lacustris | Otomys lacustris | NA | NA | 134274.7 | 81.58 | 20.946 | NA | NA | NA | NA | NA | 0.815226 |
| Rodentia | Sciuridae | Rubrisciurus | rubriventer | Rubrisciurus rubriventer | NA | NA | 134381.8 | 229.38 | 21.297 | NA | NA | NA | NA | NA | 1.012033 |
| Rodentia | Heteromyidae | Dipodomys | simulans | Dipodomys simulans | NA | NA | 134662.9 | 16.29 | 15.63 | NA | NA | NA | NA | NA | 0.012579 |
| Chiroptera | Hipposideridae | Hipposideros | pygmaeus | Hipposideros pygmaeus | 3.53 | NA | 134870.5 | 216.76 | 24.473 | NA | 1 | 1 | 2 | 3 | 0.024482 |
| Chiroptera | Rhinolophidae | Rhinolophus | creaghi | Rhinolophus creaghi | NA | NA | 134974.2 | 273.12 | 24.512 | NA | 1 | 1 | 2 | 3 | 0.009896 |
| Chiroptera | Phyllostomidae | Sturnira | nana | Sturnira nana | NA | 0.98 | 134979.8 | 131.46 | 16.258 | NA | 1 | 1 | 2 | 1 | 1.433141 |
| Rodentia | Muridae | Malacomys | cansdalei | Malacomys cansdalei | NA | NA | 135469.4 | 118.5 | 26.375 | NA | NA | NA | NA | NA | 0.019355 |
| Dasyuromorphia | Dasyuridae | Myoictis | wallacii | Myoictis wallacii | NA | NA | 135495.3 | 222.08 | 26.777 | NA | NA | NA | NA | NA | 0.014587 |
| Rodentia | Cricetidae | Microtus | thomasi | Microtus thomasi | NA | 4.32 | 135652.5 | 75.94 | 10.448 | NA | NA | NA | NA | NA | 0.013216 |
| Rodentia | Muridae | Taterillus | pygargus | Taterillus pygargus | 60.19 | 4.47 | 135754.1 | 53.49 | 27.528 | NA | 1 | NA | NA | 1 | 0.019791 |
| Primates | Cercopithecidae | Trachypithecus | auratus | Trachypithecus auratus | 9719.6 | NA | 136882.3 | 201.2 | 24.435 | 3 | NA | NA | NA | NA | 0.575295 |
| Chiroptera | Hipposideridae | Coelops | robinsoni | Coelops robinsoni | 6.5 | NA | 136979.5 | 253.05 | 22.743 | NA | 1 | 1 | 2 | 3 | 0.333059 |
| Rodentia | Muridae | Pseudomys | novaehollandiae | Pseudomys novaehollandiae | 16.8 | 4.43 | 137359.8 | 75.67 | 14.125 | NA | NA | NA | NA | NA | 1.082473 |
| Afrosoricida | Tenrecidae | Microgale | brevicaudata | Microgale brevicaudata | 8.99 | 4.5 | 137477.3 | 132.3 | 22.208 | 1 | 1 | 2 | 1 | 3 | 0.021013 |
| Primates | Pitheciidae | Callicebus | brunneus | Callicebus brunneus | 850.84 | 1.02 | 137589.1 | 173.37 | 24.448 | 3 | NA | 1 | 2 | NA | 0.012996 |
| Diprotodontia | Macropodidae | Dorcopsis | hageni | Dorcopsis hageni | 5499.99 | 1.01 | 137660.4 | 252.76 | 25.816 | 3 | 3 | 1 | 1 | 2 | 0.015865 |
| Erinaceomorpha | Erinaceidae | Neotetracus | sinensis | Neotetracus sinensis | NA | 4.28 | 137875.2 | 134.5 | 8.751 | 1 | 1 | 1 | 1 | 3 | 0.023309 |
| Rodentia | Cricetidae | Reithrodontomys | microdon | Reithrodontomys microdon | 20 | NA | 138082 | 92.17 | 19.549 | 1 | NA | 2 | 2 | NA | 0.849184 |
| Chiroptera | Phyllostomidae | Ectophylla | alba | Ectophylla alba | 5.55 | 0.99 | 138091.2 | 232.56 | 23.95 | NA | 1 | 1 | 2 | 1 | 0.177138 |
| Rodentia | Muridae | Sundamys | infraluteus | Sundamys infraluteus | 418.5 | NA | 139160.4 | 244.69 | 24.241 | NA | NA | NA | NA | NA | 0.01931 |
| Rodentia | Cricetidae | Akodon | molinae | Akodon molinae | 32.99 | 3.77 | 139180.2 | 30.73 | 14.313 | NA | NA | 1 | 1 | NA | 0.009155 |
| Rodentia | Cricetidae | Oryzomys | melanotis | Oryzomys melanotis | 49.99 | 3.88 | 139186.1 | 88.73 | 20.643 | NA | NA | 2 | 2 | NA | 0.01522 |
| Rodentia | Cricetidae | Microtus | cabrerae | Microtus cabrerae | 52.5 | NA | 139707.6 | 50.22 | 12.995 | NA | NA | NA | NA | NA | 0.159327 |
| Rodentia | Muridae | Pseudomys | oralis | Pseudomys oralis | 95 | NA | 139910.8 | 81.84 | 15.791 | NA | NA | NA | NA | NA | 1.082473 |
| Primates | Hylobatidae | Hylobates | agilis | Hylobates agilis | 5829.08 | 1.02 | 140009.7 | 239.8 | 25.416 | 3 | 4 | NA | NA | 2 | 0.984852 |
| Rodentia | Echimyidae | Diplomys | labilis | Diplomys labilis | 282.17 | 1.18 | 140013.7 | 260.52 | 24.507 | 1 | 2 | 2 | 2 | 1 | 0.016109 |
| Rodentia | Cricetidae | Oryzomys | chapmani | Oryzomys chapmani | 49.99 | NA | 140377.7 | 97.38 | 21.11 | NA | NA | NA | NA | NA | 0.01522 |
| Primates | Hylobatidae | Hylobates | pileatus | Hylobates pileatus | 5542.37 | 1 | 140831 | 157.6 | 25.99 | 3 | 5 | 1 | 2 | 2 | 1.067786 |
| Rodentia | Muridae | Mallomys | rothschildi | Mallomys rothschildi | 1157.5 | 1.02 | 141297.5 | 267.81 | 19.387 | 1 | 1 | 2 | 2 | 1 | 0.018626 |
| Rodentia | Muridae | Micaelamys | granti | Micaelamys granti | 40 | NA | 141399.2 | 19.65 | 15.745 | NA | NA | NA | NA | NA | 0.021347 |
| Rodentia | Cricetidae | Galenomys | garleppi | Galenomys garleppi | 59.3 | NA | 141711 | 54.88 | 3.183 | NA | NA | NA | NA | NA | 0.317036 |
| Primates | Daubentoniidae | Daubentonia | madagascariensis | Daubentonia madagascariensis | 2731.37 | 1.01 | 141979.7 | 149.13 | 21.7 | 1 | 6 | 2 | 2 | 2 | 0.260496 |
| Didelphimorphia | Didelphidae | Didelphis | imperfecta | Didelphis imperfecta | NA | NA | 142295.3 | 161.83 | 23.167 | NA | NA | NA | NA | NA | 0.019608 |
| Rodentia | Sciuridae | Sciurus | stramineus | Sciurus stramineus | 433.3 | NA | 142389.1 | 73.79 | 19.687 | 3 | NA | 2 | 2 | NA | 0.01121 |
| Diprotodontia | Macropodidae | Macropus | irma | Macropus irma | 8000 | 1 | 142571.5 | 52.21 | 15.599 | 1 | NA | 1 | 1 | NA | 0.017283 |
| Rodentia | Sciuridae | Tamias | rufus | Tamias rufus | 53.6 | NA | 142587.3 | 20.81 | 6.034 | 3 | 3 | 2 | 2 | 1 | 0.013459 |
| Rodentia | Cricetidae | Scapteromys | aquaticus | Scapteromys aquaticus | NA | NA | 142975.6 | 93.53 | 23.194 | NA | NA | NA | NA | NA | 0.015226 |
| Dasyuromorphia | Dasyuridae | Phascomurexia | naso | Phascomurexia naso | 52.62 | 3.68 | 142979.9 | 274.79 | 18.553 | 2 | 1 | 3 | 2 | 3 | 0.014614 |
| Chiroptera | Emballonuridae | Emballonura | furax | Emballonura furax | NA | NA | 143071.8 | 350.99 | 21.349 | 1 | NA | 1 | 2 | NA | 0.383921 |
| Rodentia | Cricetidae | Blarinomys | breviceps | Blarinomys breviceps | 36.79 | 1.21 | 143514 | 119.91 | 21.25 | NA | 1 | 2 | 1 | 3 | 0.019815 |
| Macroscelidea | Macroscelididae | Elephantulus | edwardii | Elephantulus edwardii | 49.69 | 1.8 | 143549.2 | 22.65 | 16.12 | 1 | NA | 1 | 1 | NA | 0.020288 |
| Perissodactyla | Equidae | Equus | zebra | Equus zebra | 282462.1 | 1 | 143855.8 | 19.95 | 14.442 | 3 | 2 | 1 | 1 | 1 | 0.883401 |
| Chiroptera | Rhinolophidae | Rhinolophus | silvestris | Rhinolophus silvestris | NA | NA | 144051.4 | 154.38 | 24.104 | NA | NA | 1 | 2 | NA | 0.195316 |
| Soricomorpha | Soricidae | Chimarrogale | platycephalus | Chimarrogale platycephalus | 36.32 | NA | 144298.5 | 145.89 | 11.315 | 3 | 2 | 2 | 1 | 3 | 0.021137 |
| Primates | Atelidae | Ateles | hybridus | Ateles hybridus | NA | NA | 144643.8 | 114.72 | 25.557 | NA | NA | NA | NA | NA | 1.355231 |
| Artiodactyla | Bovidae | Capra | ibex | Capra ibex | 69546.83 | 1.11 | 145098.1 | 87.78 | 4.276 | NA | 2 | 1 | 1 | 1 | 0.015431 |
| Rodentia | Bathyergidae | Georychus | capensis | Georychus capensis | 188.36 | 5.94 | 145197.2 | 51.45 | 15.187 | 3 | 2 | 1 | 1 | 2 | 0.01994 |
| Afrosoricida | Chrysochloridae | Chrysochloris | asiatica | Chrysochloris asiatica | 37.16 | 3.49 | 145686 | 19.96 | 16.109 | 2 | 2 | 2 | 1 | 3 | 0.022697 |
| Scandentia | Tupaiidae | Tupaia | javanica | Tupaia javanica | NA | 2.95 | 146194.8 | 223 | 24.25 | NA | NA | NA | NA | NA | 0.023474 |
| Rodentia | Sciuridae | Marmota | marmota | Marmota marmota | 4059.15 | 4 | 146907.4 | 83.37 | 4.667 | NA | 3 | NA | NA | 1 | 0.010285 |
| Rodentia | Muridae | Otomys | saundersiae | Otomys saundersiae | NA | NA | 147404.8 | 38.92 | 15.181 | NA | NA | NA | NA | NA | 0.019294 |
| Diprotodontia | Macropodidae | Dorcopsulus | vanheurni | Dorcopsulus vanheurni | 1890.36 | 1.18 | 147605.9 | 270.82 | 19.611 | 2 | 2 | 1 | 1 | 1 | 0.148648 |
| Chiroptera | Vespertilionidae | Miniopterus | pusillus | Miniopterus pusillus | 8.95 | NA | 147746.6 | 154.37 | 23.356 | NA | NA | 1 | 2 | NA | 0.016494 |
| Rodentia | Sciuridae | Tamias | ruficaudus | Tamias ruficaudus | 60.06 | 4.85 | 147756.8 | 52.71 | 3.281 | 3 | 4 | 2 | 2 | 2 | 0.015605 |
| Rodentia | Cricetidae | Lasiopodomys | fuscus | Lasiopodomys fuscus | NA | NA | 148192.7 | 77.81 | -7.777 | NA | NA | NA | NA | NA | 0.017657 |
| Rodentia | Cricetidae | Irenomys | tarsalis | Irenomys tarsalis | 43.15 | 3.85 | 148428.5 | 134.88 | 6.87 | 1 | 7 | 2 | 2 | 2 | 0.019815 |
| Rodentia | Echimyidae | Phyllomys | brasiliensis | Phyllomys brasiliensis | 312.49 | NA | 148527.9 | 113.33 | 20.211 | 1 | NA | 1 | 2 | NA | 1.337274 |
| Diprotodontia | Macropodidae | Petrogale | rothschildi | Petrogale rothschildi | 4546.31 | NA | 148697.3 | 22.49 | 24.114 | NA | NA | 1 | 1 | NA | 0.01696 |
| Chiroptera | Rhinolophidae | Rhinolophus | bocharicus | Rhinolophus bocharicus | 15.05 | NA | 149108.2 | 19.81 | 11.604 | NA | NA | NA | NA | NA | 0.009408 |
| Rodentia | Muridae | Gerbillus | poecilops | Gerbillus poecilops | NA | NA | 149223.4 | 12.6 | 21.026 | NA | NA | NA | NA | NA | 0.022232 |
| Dasyuromorphia | Dasyuridae | Myoictis | melas | Myoictis melas | 221.77 | NA | 149974.1 | 230.66 | 22.136 | 2 | NA | 1 | 1 | NA | 0.23339 |
| Primates | Cercopithecidae | Pygathrix | nemaeus | Pygathrix nemaeus | 9411.1 | 1.01 | 150210.7 | 189.53 | 22.537 | 3 | 2 | NA | NA | 1 | 1.011098 |
| Chiroptera | Emballonuridae | Emballonura | beccarii | Emballonura beccarii | 4.31 | NA | 150539 | 312.79 | 19.568 | 1 | NA | 1 | 2 | NA | 0.023995 |
| Rodentia | Cricetidae | Necromys | lactens | Necromys lactens | 32.9 | NA | 151012.6 | 64.92 | 11.977 | NA | NA | NA | NA | NA | 0.012975 |
| Rodentia | Muridae | Otomys | typus | Otomys typus | NA | 1.7 | 151160.2 | 88.73 | 16.336 | NA | NA | NA | NA | NA | 0.019294 |
| Rodentia | Cricetidae | Mesocricetus | raddei | Mesocricetus raddei | NA | 8.99 | 151443 | 35.42 | 8.961 | NA | NA | NA | NA | NA | 0.020434 |
| Chiroptera | Molossidae | Tadarida | lobata | Tadarida lobata | NA | NA | 151632.3 | 72.41 | 20.052 | NA | NA | 1 | 2 | NA | 0.017669 |
| Chiroptera | Molossidae | Mormopterus | acetabulosus | Mormopterus acetabulosus | NA | NA | 151638.1 | 68.18 | 17.838 | NA | NA | 1 | 2 | NA | 0.868281 |
| Rodentia | Ctenodactylidae | Felovia | vae | Felovia vae | 205 | 1 | 151907.2 | 49.5 | 28.478 | 3 | 3 | NA | NA | 1 | 0.365239 |
| Primates | Cercopithecidae | Cercopithecus | dryas | Cercopithecus dryas | 2784.06 | 1.02 | 151925.2 | 161.66 | 24.893 | 3 | NA | NA | NA | NA | 1.457185 |
| Rodentia | Sciuridae | Tamias | townsendii | Tamias townsendii | 79.12 | 3.75 | 151998.1 | 100.27 | 7.313 | 3 | 8 | 3 | 2 | 2 | 0.013314 |
| Rodentia | Muridae | Acomys | mullah | Acomys mullah | NA | NA | 152076.4 | 32.6 | 23.182 | NA | NA | NA | NA | NA | 0.013851 |
| Soricomorpha | Soricidae | Crocidura | maxi | Crocidura maxi | NA | NA | 152372.8 | 201.34 | 24.468 | NA | NA | NA | NA | NA | 0.01789 |
| Rodentia | Geomyidae | Orthogeomys | grandis | Orthogeomys grandis | 499.99 | NA | 152714.6 | 115.58 | 21.028 | NA | 3 | 2 | 1 | 1 | 0.015066 |
| Rodentia | Cricetidae | Cricetulus | alticola | Cricetulus alticola | NA | NA | 153013.4 | 52.17 | -1.107 | NA | NA | NA | NA | NA | 0.021048 |
| Rodentia | Cricetidae | Myodes | centralis | Myodes centralis | NA | 4.83 | 153338.9 | 25.45 | -2.431 | NA | NA | NA | NA | NA | 0.013359 |
| Rodentia | Muridae | Bunomys | chrysocomus | Bunomys chrysocomus | 111.29 | NA | 153381.8 | 227.92 | 21.467 | NA | NA | NA | NA | NA | 0.019562 |
| Rodentia | Muridae | Parahydromys | asper | Parahydromys asper | 526.66 | NA | 153393.2 | 276.5 | 19.812 | NA | 2 | 2 | 1 | 2 | 0.022938 |
| Dasyuromorphia | Dasyuridae | Dasyurus | geoffroii | Dasyurus geoffroii | 1106.62 | 5.47 | 153816.1 | 47.24 | 15.433 | 2 | 2 | 1 | 1 | 3 | 0.115077 |
| Primates | Cercopithecidae | Theropithecus | gelada | Theropithecus gelada | 15964.11 | 1.01 | 153882.5 | 92.49 | 17.339 | 3 | 2 | NA | NA | 1 | 0.016453 |
| Primates | Cercopithecidae | Presbytis | femoralis | Presbytis femoralis | 7026.91 | NA | 154945.8 | 202.31 | 25.769 | 3 | NA | NA | NA | NA | 0.117032 |
| Dasyuromorphia | Dasyuridae | Murexia | longicaudata | Murexia longicaudata | 126.79 | NA | 155023 | 265.83 | 20.144 | 2 | 1 | 2 | 2 | 3 | 0.01563 |
| Artiodactyla | Bovidae | Capra | falconeri | Capra falconeri | 54303.37 | 1.73 | 155075.9 | 22.18 | 10.854 | 3 | 2 | 1 | 1 | 1 | 1.15736 |
| Rodentia | Muridae | Leopoldamys | ciliatus | Leopoldamys ciliatus | NA | NA | 155314.8 | 232.8 | 24.088 | NA | NA | NA | NA | NA | 0.017821 |
| Rodentia | Cricetidae | Neusticomys | venezuelae | Neusticomys venezuelae | 47.1 | NA | 156124.9 | 152.67 | 23.041 | NA | 1 | 1 | NA | 3 | 0.836535 |
| Diprotodontia | Macropodidae | Thylogale | thetis | Thylogale thetis | 5399.95 | 1 | 156197 | 83.16 | 16.16 | 1 | NA | 1 | 1 | NA | 0.01326 |
| Rodentia | Muridae | Acomys | ignitus | Acomys ignitus | NA | NA | 156582 | 64.98 | 23.982 | NA | NA | NA | NA | NA | 0.013851 |
| Soricomorpha | Talpidae | Euroscaptor | micrura | Euroscaptor micrura | 60.46 | NA | 157260.5 | 159.76 | 13.222 | NA | 1 | 2 | 1 | 3 | 0.021789 |
| Rodentia | Sciuridae | Sundasciurus | brookei | Sundasciurus brookei | 114.05 | NA | 157725.8 | 281.4 | 22.295 | NA | NA | NA | NA | NA | 0.020635 |
| Primates | Aotidae | Aotus | nancymaae | Aotus nancymaae | 791.03 | 1.02 | 157986 | 228.04 | 26.124 | 1 | 2 | 1 | 2 | 2 | 0.021419 |
| Rodentia | Dipodidae | Allactaga | vinogradovi | Allactaga vinogradovi | NA | NA | 158260.7 | 22.66 | 10.319 | NA | NA | NA | NA | NA | 0.231243 |
| Rodentia | Cricetidae | Peromyscus | californicus | Peromyscus californicus | 42.68 | 1.94 | 158708.4 | 31.03 | 12.513 | 1 | 6 | NA | NA | 2 | 0.017825 |
| Primates | Hylobatidae | Hylobates | albibarbis | Hylobates albibarbis | NA | NA | 159603.7 | 243.75 | 25.884 | NA | NA | NA | NA | NA | 0.984852 |
| Chiroptera | Hipposideridae | Hipposideros | semoni | Hipposideros semoni | 14 | NA | 159686.8 | 132.29 | 22.849 | NA | NA | 1 | 2 | NA | 0.293954 |
| Rodentia | Echimyidae | Echimys | saturnus | Echimys saturnus | 626.89 | NA | 159720.9 | 238 | 25.272 | NA | NA | NA | NA | NA | 0.285285 |
| Rodentia | Cricetidae | Akodon | simulator | Akodon simulator | 42.5 | NA | 160100.3 | 55.54 | 13.357 | NA | NA | NA | NA | NA | 0.009155 |
| Rodentia | Gliridae | Graphiurus | crassicaudatus | Graphiurus crassicaudatus | NA | 3.16 | 160434.4 | 188.67 | 24.984 | 1 | 4 | 2 | 2 | 2 | 0.382504 |
| Primates | Cebidae | Callithrix | emiliae | Callithrix emiliae | 309.58 | NA | 160569 | 193.59 | 24.926 | NA | NA | NA | NA | NA | 0.175051 |
| Rodentia | Cricetidae | Necromys | obscurus | Necromys obscurus | 40.69 | 4.86 | 160780.6 | 75.58 | 14.815 | 3 | 3 | 3 | 1 | 2 | 0.121253 |
| Rodentia | Cricetidae | Akodon | aerosus | Akodon aerosus | 59.99 | NA | 160799.5 | 200.29 | 19.443 | NA | NA | NA | NA | NA | 0.016032 |
| Rodentia | Muridae | Paramelomys | moncktoni | Paramelomys moncktoni | 80.56 | 2.15 | 160894.5 | 294.31 | 20.991 | NA | 3 | NA | NA | 1 | 0.020235 |
| Rodentia | Dipodidae | Zapus | trinotatus | Zapus trinotatus | 27.45 | 5.47 | 162680.2 | 95.81 | 7.586 | 2 | 3 | NA | NA | 2 | 0.019963 |
| Pilosa | Bradypodidae | Bradypus | torquatus | Bradypus torquatus | 4468.42 | 1 | 163652.8 | 126.13 | 22.069 | 2 | 4 | 3 | 2 | 2 | 1.76848 |
| Rodentia | Heteromyidae | Liomys | salvini | Liomys salvini | 41.99 | 3.69 | 163721.2 | 152.01 | 22.204 | NA | 3 | 1 | 1 | 2 | 0.013453 |
| Rodentia | Gliridae | Graphiurus | rupicola | Graphiurus rupicola | NA | NA | 164009.3 | 16.6 | 16.706 | NA | NA | NA | NA | NA | 0.019853 |
| Rodentia | Gliridae | Selevinia | betpakdalaensis | Selevinia betpakdalaensis | 17.12 | 6.9 | 164721.4 | 25.79 | 7.764 | 2 | 1 | 2 | 1 | 3 | 0.365831 |
| Rodentia | Cricetidae | Akodon | juninensis | Akodon juninensis | 38.99 | NA | 164823.9 | 99.24 | 12.723 | NA | NA | NA | NA | NA | 0.011694 |
| Diprotodontia | Macropodidae | Petrogale | concinna | Petrogale concinna | 1398.2 | 1 | 166175.4 | 92.79 | 27.52 | 1 | NA | 1 | 1 | NA | 0.191236 |
| Lagomorpha | Ochotonidae | Ochotona | huangensis | Ochotona huangensis | 105.33 | NA | 166222.2 | 59.62 | 7.046 | NA | NA | NA | NA | NA | 0.018114 |
| Primates | Pitheciidae | Callicebus | barbarabrownae | Callicebus barbarabrownae | NA | NA | 167232.1 | 73.82 | 24.067 | NA | NA | NA | NA | NA | 1.770166 |
| Rodentia | Echimyidae | Phyllomys | blainvillii | Phyllomys blainvillii | 243.3 | NA | 167672.4 | 71.01 | 23.432 | 1 | NA | 1 | 2 | NA | 0.015266 |
| Dasyuromorphia | Dasyuridae | Pseudantechinus | ningbing | Pseudantechinus ningbing | 20.74 | 4 | 167787.2 | 65.5 | 27.314 | 2 | NA | 1 | 1 | NA | 0.016133 |
| Rodentia | Heteromyidae | Heteromys | gaumeri | Heteromys gaumeri | 63.59 | 2.95 | 168039.1 | 107.19 | 25.312 | NA | 2 | 1 | 1 | 1 | 0.01769 |
| Dasyuromorphia | Dasyuridae | Ningaui | timealeyi | Ningaui timealeyi | 6.37 | 5.7 | 169167.7 | 22.1 | 24.219 | 2 | NA | 1 | 1 | NA | 0.019046 |
| Rodentia | Hystricidae | Hystrix | javanica | Hystrix javanica | NA | NA | 170023.7 | 211.08 | 24.328 | NA | 6 | 2 | 1 | 2 | 0.019685 |
| Primates | Cercopithecidae | Semnopithecus | priam | Semnopithecus priam | NA | NA | 170165.5 | 107.23 | 26.883 | NA | NA | NA | NA | NA | 0.13347 |
| Rodentia | Dipodidae | Salpingotus | pallidus | Salpingotus pallidus | NA | NA | 170262.7 | 20.95 | 6.094 | NA | NA | NA | NA | NA | 0.352001 |
| Chiroptera | Pteropodidae | Cynopterus | luzoniensis | Cynopterus luzoniensis | NA | NA | 170486.4 | 229.41 | 21.547 | NA | NA | NA | NA | NA | 0.015386 |
| Rodentia | Muridae | Maxomys | hellwaldii | Maxomys hellwaldii | NA | NA | 170486.4 | 229.41 | 21.547 | NA | NA | NA | NA | NA | 0.021458 |
| Rodentia | Muridae | Maxomys | musschenbroekii | Maxomys musschenbroekii | NA | NA | 170486.4 | 229.41 | 21.547 | NA | NA | NA | NA | NA | 0.021458 |
| Rodentia | Muridae | Paruromys | dominator | Paruromys dominator | 325.31 | NA | 170486.4 | 229.41 | 21.547 | NA | NA | NA | NA | NA | 0.023685 |
| Chiroptera | Vespertilionidae | Scotophilus | celebensis | Scotophilus celebensis | NA | NA | 170486.4 | 229.41 | 21.547 | NA | NA | 1 | 2 | NA | 0.218851 |
| Rodentia | Muridae | Rattus | hoffmanni | Rattus hoffmanni | NA | NA | 170563.2 | 229.41 | 21.547 | NA | NA | NA | NA | NA | 0.013963 |
| Chiroptera | Emballonuridae | Saccopteryx | gymnura | Saccopteryx gymnura | NA | NA | 170607 | 162.68 | 25.502 | 1 | 1 | 1 | 2 | 3 | 0.32863 |
| Afrosoricida | Chrysochloridae | Chrysospalax | villosus | Chrysospalax villosus | 108.35 | 1.93 | 170736.4 | 69.15 | 16.581 | NA | 1 | 2 | 1 | 3 | 1.107105 |
| Primates | Tarsiidae | Tarsius | tarsier | Tarsius tarsier | 168.04 | 1 | 170743.5 | 229.41 | 21.547 | 1 | 3 | NA | NA | 2 | 1.077482 |
| Scandentia | Tupaiidae | Tupaia | splendidula | Tupaia splendidula | 168.05 | NA | 170822.4 | 229.89 | 25.816 | 3 | 4 | NA | NA | 2 | 0.023474 |
| Artiodactyla | Bovidae | Gazella | spekei | Gazella spekei | 20000 | 1 | 170828.3 | 13.51 | 24.839 | NA | NA | 1 | 1 | NA | 0.836167 |
| Carnivora | Viverridae | Cynogale | bennettii | Cynogale bennettii | 4246.57 | 2.3 | 170977.1 | 235.45 | 25.44 | 2 | 6 | 2 | 1 | 2 | 1.55091 |
| Rodentia | Anomaluridae | Zenkerella | insignis | Zenkerella insignis | 200 | NA | 171070.5 | 151.21 | 23.898 | NA | NA | 2 | 2 | NA | 0.023359 |
| Chiroptera | Pteropodidae | Megaerops | niphanae | Megaerops niphanae | 32.59 | NA | 171269.5 | 137.76 | 22.622 | NA | NA | 1 | 2 | NA | 0.016408 |
| Soricomorpha | Talpidae | Galemys | pyrenaicus | Galemys pyrenaicus | 60.17 | 3.54 | 171300.9 | 65.84 | 10.711 | 2 | 2 | 3 | 1 | 3 | 1.147565 |
| Rodentia | Cricetidae | Neotoma | goldmani | Neotoma goldmani | 198 | 1.29 | 171526.5 | 25.72 | 16.775 | NA | NA | NA | NA | NA | 0.015637 |
| Chiroptera | Vespertilionidae | Murina | ussuriensis | Murina ussuriensis | NA | NA | 171545.7 | 47.62 | -0.011 | NA | NA | 1 | 2 | NA | 0.015749 |
| Artiodactyla | Bovidae | Cephalophus | zebra | Cephalophus zebra | 15654.75 | 1 | 171546.5 | 214.55 | 24.786 | NA | 3 | 1 | 1 | 2 | 0.75658 |
| Rodentia | Sciuridae | Spermophilus | relictus | Spermophilus relictus | 599.99 | 4.89 | 171697 | 26.21 | -1.179 | NA | NA | NA | NA | NA | 0.008649 |
| Diprotodontia | Macropodidae | Petrogale | penicillata | Petrogale penicillata | 6931.6 | 1 | 171742.4 | 72.16 | 15.413 | 1 | NA | 1 | 1 | NA | 0.147039 |
| Lagomorpha | Leporidae | Pronolagus | crassicaudatus | Pronolagus crassicaudatus | 2415.27 | 1.5 | 172093.9 | 70.4 | 16.441 | 1 | 2 | 1 | 1 | 1 | 0.020022 |
| Primates | Pitheciidae | Callicebus | personatus | Callicebus personatus | 1390.8 | 1.01 | 172664.5 | 107.39 | 21.234 | 3 | 4 | 1 | 2 | 1 | 0.931666 |
| Rodentia | Muridae | Otomys | denti | Otomys denti | 120 | 1.59 | 172854.7 | 101.67 | 21.3 | NA | NA | NA | NA | NA | 0.019294 |
| Dasyuromorphia | Dasyuridae | Antechinus | stuartii | Antechinus stuartii | 29.01 | 6.89 | 173022.7 | 87.48 | 16.872 | 2 | 3 | 2 | 2 | 2 | 0.014575 |
| Artiodactyla | Suidae | Sus | celebensis | Sus celebensis | 53813.21 | 3.24 | 173576.4 | 229.41 | 21.547 | 2 | 5 | NA | NA | 2 | 0.185173 |
| Chiroptera | Pteropodidae | Acerodon | celebensis | Acerodon celebensis | 384.84 | 0.98 | 173613.3 | 229.41 | 21.547 | NA | NA | 1 | 2 | NA | 0.015289 |
| Chiroptera | Pteropodidae | Rousettus | celebensis | Rousettus celebensis | 63.07 | 1 | 173659.3 | 229.41 | 21.547 | NA | NA | 1 | 2 | NA | 0.010275 |
| Diprotodontia | Macropodidae | Petrogale | xanthopus | Petrogale xanthopus | 8499.99 | 1.02 | 173888.4 | 20.98 | 18.345 | NA | 3 | 1 | 1 | 1 | 0.169604 |
| Chiroptera | Pteropodidae | Dobsonia | exoleta | Dobsonia exoleta | 302.03 | NA | 174051.7 | 229.47 | 21.595 | NA | NA | 1 | 2 | NA | 0.011263 |
| Chiroptera | Vespertilionidae | Eptesicus | platyops | Eptesicus platyops | NA | NA | 174149.9 | 125.05 | 26.553 | NA | NA | 1 | 2 | NA | 0.268274 |
| Rodentia | Muridae | Grammomys | buntingi | Grammomys buntingi | NA | NA | 174512.9 | 214.16 | 25.242 | NA | NA | NA | NA | NA | 0.324293 |
| Rodentia | Cricetidae | Peromyscus | keeni | Peromyscus keeni | 28.25 | 5.99 | 174924.3 | 115.59 | 4.306 | 1 | NA | NA | NA | NA | 0.017825 |
| Chiroptera | Vespertilionidae | Chalinolobus | tuberculatus | Chalinolobus tuberculatus | NA | 0.98 | 174975.2 | 90.49 | 9.458 | 2 | 1 | 1 | 2 | 3 | 0.910697 |
| Rodentia | Cricetidae | Scotinomys | teguina | Scotinomys teguina | 11.59 | 2.46 | 175099.2 | 158.13 | 22.284 | NA | 2 | 1 | 1 | 2 | 0.017103 |
| Soricomorpha | Soricidae | Megasorex | gigas | Megasorex gigas | 11.71 | NA | 175398.2 | 95.26 | 20.186 | NA | NA | 1 | 1 | NA | 0.020867 |
| Didelphimorphia | Didelphidae | Monodelphis | scalops | Monodelphis scalops | 763.19 | NA | 175704.1 | 142.13 | 18.419 | 1 | 1 | 1 | 1 | 3 | 0.024176 |
| Rodentia | Cricetidae | Rhipidomys | austrinus | Rhipidomys austrinus | 89 | NA | 175757.5 | 80.18 | 16.557 | 1 | NA | 1 | 2 | NA | 0.015785 |
| Diprotodontia | Phalangeridae | Ailurops | ursinus | Ailurops ursinus | 10000 | NA | 175900.1 | 229.47 | 21.595 | 1 | 1 | 1 | 2 | 1 | 1.013875 |
| Rodentia | Cricetidae | Delomys | sublineatus | Delomys sublineatus | 89.99 | NA | 176241.1 | 119.42 | 20.039 | 3 | NA | 1 | 1 | NA | 0.016213 |
| Rodentia | Cricetidae | Akodon | fumeus | Akodon fumeus | 22.69 | NA | 176335.2 | 103.06 | 15.305 | NA | NA | NA | NA | NA | 0.013492 |
| Rodentia | Sciuridae | Spermophilus | armatus | Spermophilus armatus | 306.48 | 5.4 | 176342 | 27.61 | 3.072 | NA | 3 | 2 | 1 | 2 | 0.010185 |
| Chiroptera | Vespertilionidae | Pipistrellus | collinus | Pipistrellus collinus | 6.75 | NA | 176698.3 | 268.58 | 19.139 | NA | NA | NA | NA | NA | 0.015988 |
| Afrosoricida | Tenrecidae | Micropotamogale | ruwenzorii | Micropotamogale ruwenzorii | 109.13 | 1.86 | 177049.5 | 112.06 | 20.263 | 1 | 2 | 3 | 1 | 3 | 0.240455 |
| Primates | Cercopithecidae | Cercopithecus | diana | Cercopithecus diana | 4358.91 | 1.01 | 177249.9 | 237.57 | 24.813 | 3 | 3 | NA | NA | 2 | 0.766939 |
| Didelphimorphia | Didelphidae | Monodelphis | theresa | Monodelphis theresa | 112 | NA | 177325.1 | 135.65 | 19.007 | 1 | 1 | 1 | 1 | 3 | 0.386822 |
| Primates | Cercopithecidae | Macaca | fuscata | Macaca fuscata | 10114.76 | 1.02 | 177385.9 | 147.66 | 10.222 | 3 | 5 | 2 | 2 | 2 | 0.014311 |
| Rodentia | Heteromyidae | Dipodomys | phillipsii | Dipodomys phillipsii | 41 | 2.59 | 177726 | 64.72 | 15.619 | 1 | 3 | 2 | 1 | 1 | 0.014994 |
| Rodentia | Cricetidae | Sigmodon | alleni | Sigmodon alleni | 174.09 | NA | 177949.9 | 90.52 | 21.078 | NA | NA | NA | NA | NA | 0.654247 |
| Artiodactyla | Bovidae | Capra | nubiana | Capra nubiana | 47763.72 | 1 | 177986.8 | 7.67 | 20.854 | NA | NA | NA | NA | NA | 0.771573 |
| Perissodactyla | Tapiridae | Tapirus | pinchaque | Tapirus pinchaque | 156923.4 | 1 | 178168.3 | 148.68 | 21.412 | 2 | 4 | 1 | 1 | 1 | 1.679259 |
| Rodentia | Caviidae | Cavia | magna | Cavia magna | 459.99 | NA | 178490.9 | 119.18 | 16.622 | NA | NA | 1 | 1 | NA | 0.020458 |
| Chiroptera | Hipposideridae | Hipposideros | ridleyi | Hipposideros ridleyi | 9.59 | 0.98 | 178832.5 | 268.14 | 23.702 | NA | 1 | 1 | 2 | 3 | 1.224118 |
| Chiroptera | Vespertilionidae | Eudiscopus | denticulus | Eudiscopus denticulus | NA | NA | 179005.7 | 132.37 | 21.717 | NA | NA | 1 | 2 | NA | 0.365693 |
| Diprotodontia | Phalangeridae | Strigocuscus | celebensis | Strigocuscus celebensis | NA | NA | 179049.4 | 230.23 | 21.645 | 1 | NA | NA | NA | NA | 0.883462 |
| Rodentia | Sciuridae | Atlantoxerus | getulus | Atlantoxerus getulus | 250.57 | NA | 179364.3 | 25.6 | 15.601 | NA | NA | NA | NA | NA | 0.020838 |
| Didelphimorphia | Didelphidae | Gracilinanus | marica | Gracilinanus marica | 23.8 | 5.99 | 179495 | 99.07 | 22.953 | 1 | NA | 1 | 2 | NA | 0.022595 |
| Chiroptera | Vespertilionidae | Vespadelus | pumilus | Vespadelus pumilus | 5.4 | 1.21 | 179988.9 | 82.86 | 17.9 | NA | 1 | 1 | 2 | 3 | 0.017218 |
| Dasyuromorphia | Dasyuridae | Ningaui | yvonnae | Ningaui yvonnae | 9.3 | 7 | 180419.1 | 21.92 | 17.651 | 2 | NA | 1 | 1 | NA | 0.018197 |
| Rodentia | Echimyidae | Clyomys | bishopi | Clyomys bishopi | 30 | NA | 180436.5 | 134.55 | 19.02 | NA | NA | NA | NA | NA | 0.279727 |
| Carnivora | Viverridae | Genetta | johnstoni | Genetta johnstoni | 2225 | NA | 180606.4 | 219.22 | 24.668 | NA | NA | 1 | 1 | NA | 0.80482 |
| Carnivora | Felidae | Leopardus | colocolo | Leopardus colocolo | NA | NA | 180642.4 | 25.86 | 1.24 | NA | NA | NA | NA | NA | 0.154916 |
| Rodentia | Cricetidae | Osgoodomys | banderanus | Osgoodomys banderanus | 49.99 | 1.68 | 180754.4 | 91.63 | 21.132 | NA | NA | 2 | 2 | NA | 0.02046 |
| Chiroptera | Phyllostomidae | Phyllops | falcatus | Phyllops falcatus | NA | 0.98 | 181669.9 | 104.1 | 24.656 | NA | NA | 1 | 2 | NA | 0.014555 |
| Rodentia | Muridae | Pelomys | campanae | Pelomys campanae | NA | NA | 181683.6 | 92.11 | 22.753 | NA | NA | NA | NA | NA | 0.02173 |
| Diprotodontia | Phalangeridae | Phalanger | carmelitae | Phalanger carmelitae | 1820.88 | 1.01 | 181857.6 | 283.5 | 19.803 | 1 | 5 | 1 | 2 | 2 | 0.016572 |
| Rodentia | Cricetidae | Geoxus | valdivianus | Geoxus valdivianus | 30.8 | 3.4 | 182342.6 | 108.34 | 6.653 | 2 | 4 | 2 | 1 | 2 | 0.019815 |
| Rodentia | Muridae | Paramelomys | rubex | Paramelomys rubex | 47.16 | 2 | 182359.8 | 280.5 | 20.615 | NA | 3 | 3 | 2 | 1 | 0.016916 |
| Diprotodontia | Phalangeridae | Phalanger | sericeus | Phalanger sericeus | 2003 | 1.02 | 182383.6 | 285.3 | 19.715 | 1 | 2 | 1 | 2 | 1 | 0.017689 |
| Chiroptera | Molossidae | Tadarida | kuboriensis | Tadarida kuboriensis | 26.8 | NA | 182715.8 | 269.77 | 20.803 | NA | NA | NA | NA | NA | 0.016512 |
| Rodentia | Muridae | Melomys | cervinipes | Melomys cervinipes | 71.2 | 2.2 | 182871.4 | 97.37 | 20.451 | 1 | NA | 2 | 2 | NA | 0.020235 |
| Chiroptera | Vespertilionidae | Eptesicus | floweri | Eptesicus floweri | NA | NA | 182901.2 | 34.73 | 27.397 | NA | NA | 1 | 2 | NA | 0.021206 |
| Chiroptera | Phyllostomidae | Phyllonycteris | poeyi | Phyllonycteris poeyi | NA | 0.98 | 183923.6 | 103.98 | 24.661 | NA | NA | 1 | 2 | NA | 0.016863 |
| Primates | Atelidae | Alouatta | nigerrima | Alouatta nigerrima | NA | NA | 183994.3 | 170.18 | 25.674 | NA | NA | NA | NA | NA | 0.014213 |
| Chiroptera | Vespertilionidae | Myotis | ridleyi | Myotis ridleyi | 4.06 | NA | 184038.9 | 219.81 | 24.719 | NA | NA | 1 | 2 | NA | 0.142288 |
| Chiroptera | Phyllostomidae | Brachyphylla | nana | Brachyphylla nana | 37.25 | 0.98 | 184267.9 | 103.98 | 24.661 | NA | NA | 1 | 2 | NA | 0.018591 |
| Chiroptera | Rhinolophidae | Rhinolophus | deckenii | Rhinolophus deckenii | NA | NA | 184645.4 | 81 | 24.559 | NA | NA | 1 | 2 | NA | 0.122072 |
| Primates | Galagidae | Galago | matschiei | Galago matschiei | 211.61 | 0.99 | 184700.2 | 116.5 | 21.501 | NA | NA | NA | NA | NA | 0.021087 |
| Soricomorpha | Soricidae | Crocidura | nigeriae | Crocidura nigeriae | 23 | NA | 184841.8 | 183.39 | 25.466 | NA | NA | NA | NA | NA | 0.01789 |
| Chiroptera | Vespertilionidae | Lasiurus | egregius | Lasiurus egregius | NA | NA | 184920.3 | 137.5 | 17.949 | NA | NA | 1 | 2 | NA | 0.287758 |
| Primates | Lorisidae | Arctocebus | calabarensis | Arctocebus calabarensis | 258.01 | 1.01 | 185093.8 | 184.44 | 24.775 | 1 | 3 | NA | NA | 2 | 0.02175 |
| Chiroptera | Hipposideridae | Hipposideros | megalotis | Hipposideros megalotis | NA | NA | 186130 | 82.36 | 17.469 | NA | NA | 1 | 2 | NA | 0.024482 |
| Rodentia | Cricetidae | Microtus | transcaspicus | Microtus transcaspicus | NA | 2.1 | 186224.9 | 31.29 | 12.944 | NA | NA | NA | NA | NA | 0.013117 |
| Rodentia | Muridae | Arvicanthis | nairobae | Arvicanthis nairobae | NA | NA | 186397 | 63.55 | 21.144 | NA | NA | NA | NA | NA | 0.018553 |
| Rodentia | Sciuridae | Hylopetes | lepidus | Hylopetes lepidus | 63.28 | 2 | 186400.7 | 230.63 | 23.631 | 1 | 5 | 1 | 2 | 2 | 0.211113 |
| Chiroptera | Vespertilionidae | Myotis | morrisi | Myotis morrisi | NA | NA | 186988.7 | 115.66 | 23.13 | NA | NA | 1 | 2 | NA | 0.219846 |
| Soricomorpha | Soricidae | Crocidura | smithii | Crocidura smithii | NA | NA | 187243 | 55.83 | 24.1 | NA | NA | NA | NA | NA | 0.01789 |
| Rodentia | Muridae | Uromys | anak | Uromys anak | 745.38 | 4.3 | 187554.9 | 262.1 | 19.972 | NA | 3 | 2 | 2 | 1 | 0.017453 |
| Diprotodontia | Phalangeridae | Trichosurus | caninus | Trichosurus caninus | 3115.06 | 1.02 | 187766.8 | 77.62 | 14.339 | 1 | 1 | 1 | 2 | 1 | 0.018404 |
| Rodentia | Muridae | Melomys | leucogaster | Melomys leucogaster | 104.17 | 2.15 | 188251.5 | 266.38 | 25.127 | NA | 3 | 2 | 2 | 1 | 0.016211 |
| Carnivora | Viverridae | Poiana | leightoni | Poiana leightoni | NA | NA | 188494.8 | 200.92 | 24.965 | NA | NA | NA | NA | NA | 0.269384 |
| Chiroptera | Vespertilionidae | Pipistrellus | westralis | Pipistrellus westralis | NA | NA | 188611.6 | 78.31 | 27.16 | NA | NA | NA | NA | NA | 0.015988 |
| Artiodactyla | Camelidae | Camelus | bactrianus | Camelus bactrianus | 554515.9 | 1.39 | 188693 | 9.94 | 5.372 | NA | 2 | 1 | 1 | 1 | 2.198565 |
| Perissodactyla | Tapiridae | Tapirus | bairdii | Tapirus bairdii | 293781.6 | 1 | 188831.8 | 166.98 | 23.549 | 2 | 4 | 1 | 1 | 1 | 1.896278 |
| Didelphimorphia | Didelphidae | Caluromysiops | irrupta | Caluromysiops irrupta | 257.48 | 2 | 188864.4 | 189.96 | 24.603 | 1 | 3 | 1 | 2 | 2 | 0.022321 |
| Chiroptera | Vespertilionidae | Eptesicus | bobrinskoi | Eptesicus bobrinskoi | 7.54 | NA | 189044.1 | 15.49 | 5.675 | NA | NA | 1 | 2 | NA | 0.0158 |
| Rodentia | Muridae | Aethomys | thomasi | Aethomys thomasi | NA | NA | 189060 | 98.49 | 20.186 | NA | NA | NA | NA | NA | 0.021347 |
| Chiroptera | Molossidae | Mormopterus | jugularis | Mormopterus jugularis | 11.56 | NA | 189665.1 | 147.12 | 20.193 | NA | 1 | 1 | 2 | 3 | 0.017366 |
| Carnivora | Felidae | Leopardus | guigna | Leopardus guigna | NA | NA | 190039.8 | 102.85 | 8.363 | NA | NA | NA | NA | NA | 0.812035 |
| Rodentia | Gliridae | Glirulus | japonicus | Glirulus japonicus | NA | 4 | 190065.3 | 150.46 | 11.654 | 1 | 4 | 1 | 2 | 2 | 0.025532 |
| Soricomorpha | Soricidae | Crocidura | religiosa | Crocidura religiosa | 7 | NA | 190264.7 | 1.07 | 21.909 | NA | NA | NA | NA | NA | 0.286234 |
| Soricomorpha | Soricidae | Crocidura | pasha | Crocidura pasha | 6.5 | NA | 191006.2 | 4.13 | 26.542 | NA | NA | NA | NA | NA | 0.01789 |
| Rodentia | Cricetidae | Rhipidomys | macconnelli | Rhipidomys macconnelli | 41.59 | NA | 191016.9 | 174.87 | 23.021 | 1 | NA | 1 | 2 | NA | 0.015785 |
| Primates | Cebidae | Saguinus | imperator | Saguinus imperator | 407.91 | NA | 191381.6 | 212.79 | 24.94 | 3 | 2 | 1 | 2 | 2 | 0.014176 |
| Rodentia | Muridae | Pseudomys | australis | Pseudomys australis | 52.97 | 3 | 191946.3 | 16.65 | 19.306 | NA | NA | NA | NA | NA | 1.082473 |
| Primates | Hylobatidae | Nomascus | concolor | Nomascus concolor | 6410.47 | 1.01 | 191956.2 | 119.62 | 17.293 | 3 | 5 | 1 | 2 | 2 | 1.352656 |
| Rodentia | Muridae | Niviventer | langbianis | Niviventer langbianis | NA | NA | 192161.8 | 179.56 | 22.351 | NA | NA | NA | NA | NA | 0.021458 |
| Chiroptera | Natalidae | Chilonatalus | micropus | Chilonatalus micropus | NA | NA | 192774 | 106.88 | 24.631 | NA | 1 | 1 | 2 | 3 | 0.210168 |
| Soricomorpha | Soricidae | Crocidura | crenata | Crocidura crenata | NA | NA | 192943.9 | 170.65 | 23.909 | NA | NA | NA | NA | NA | 0.01789 |
| Perissodactyla | Equidae | Equus | grevyi | Equus grevyi | 408000.4 | 1 | 192991.3 | 50.35 | 24.821 | NA | 1 | 1 | 1 | 1 | 1.325101 |
| Rodentia | Nesomyidae | Dendromus | lovati | Dendromus lovati | 12 | NA | 193162.6 | 89.72 | 14.937 | NA | NA | NA | NA | NA | 0.022547 |
| Rodentia | Heteromyidae | Dipodomys | nelsoni | Dipodomys nelsoni | 87.93 | 1.94 | 193502.5 | 23.26 | 17.785 | 1 | 1 | 2 | 1 | 1 | 0.014152 |
| Rodentia | Muridae | Lorentzimys | nouhuysi | Lorentzimys nouhuysi | 14.44 | 2.75 | 193622.2 | 271.15 | 20.323 | 2 | 5 | 2 | 2 | 2 | 0.019897 |
| Rodentia | Dipodidae | Pygeretmus | shitkovi | Pygeretmus shitkovi | NA | 5.49 | 194200.5 | 31.63 | 8.923 | 1 | 4 | 2 | 1 | 2 | 0.214241 |
| Primates | Hylobatidae | Nomascus | leucogenys | Nomascus leucogenys | 7320 | NA | 194572.8 | 123.41 | 19.067 | 3 | NA | NA | NA | NA | 1.369327 |
| Soricomorpha | Soricidae | Sorex | ornatus | Sorex ornatus | 5.31 | 4.99 | 194623.5 | 35.99 | 12.76 | 3 | NA | NA | NA | NA | 0.018367 |
| Diprotodontia | Macropodidae | Thylogale | browni | Thylogale browni | 5478 | 1.02 | 194749.1 | 279.46 | 22.836 | NA | NA | 1 | 1 | NA | 0.681258 |
| Rodentia | Muridae | Rattus | argentiventer | Rattus argentiventer | 165.37 | 9.85 | 195050.2 | 157.85 | 26.239 | NA | 5 | NA | NA | 2 | 0.013963 |
| Chiroptera | Vespertilionidae | Cistugo | lesueuri | Cistugo lesueuri | NA | NA | 195426.4 | 27.82 | 15.483 | NA | NA | 1 | 2 | NA | 0.013729 |
| Didelphimorphia | Didelphidae | Micoureus | alstoni | Micoureus alstoni | 132.3 | NA | 196544.9 | 209.32 | 23.063 | 1 | NA | NA | NA | NA | 0.021375 |
| Primates | Cercopithecidae | Presbytis | hosei | Presbytis hosei | 6277.78 | NA | 196668.5 | 283.13 | 23.696 | 3 | NA | NA | NA | NA | 0.587185 |
| Soricomorpha | Soricidae | Crocidura | monax | Crocidura monax | 16 | NA | 196976.5 | 72.83 | 19.671 | NA | NA | NA | NA | NA | 0.01789 |
| Rodentia | Sciuridae | Spermophilus | alashanicus | Spermophilus alashanicus | NA | NA | 197144.2 | 28.29 | 0.906 | NA | NA | NA | NA | NA | 0.010922 |
| Carnivora | Herpestidae | Herpestes | vitticollis | Herpestes vitticollis | 2380 | 2.73 | 197229 | 129.65 | 25.843 | 2 | NA | 1 | 1 | NA | 0.020899 |
| Soricomorpha | Soricidae | Crocidura | virgata | Crocidura virgata | NA | NA | 197818.1 | 183.29 | 25.367 | NA | NA | NA | NA | NA | 0.24623 |
| Dasyuromorphia | Dasyuridae | Murexechinus | melanurus | Murexechinus melanurus | 38.02 | 3.49 | 198231.1 | 264.71 | 20.158 | 2 | 1 | 1 | 1 | 3 | 0.016099 |
| Erinaceomorpha | Erinaceidae | Atelerix | sclateri | Atelerix sclateri | NA | NA | 198274.3 | 12.2 | 24.523 | 1 | NA | NA | NA | NA | 0.018239 |
| Rodentia | Cricetidae | Peromyscus | merriami | Peromyscus merriami | 40 | 3.4 | 198360.4 | 26.37 | 21.04 | 1 | NA | NA | NA | NA | 0.017825 |
| Rodentia | Cricetidae | Phyllotis | osilae | Phyllotis osilae | 43.64 | 4.25 | 198550.5 | 125.01 | 13.439 | 1 | 3 | NA | NA | 2 | 0.016023 |
| Rodentia | Cricetidae | Chelemys | macronyx | Chelemys macronyx | 70.99 | 4.37 | 199621 | 74.44 | 5.462 | 2 | 4 | 2 | 1 | 2 | 0.015847 |
| Chiroptera | Pteropodidae | Nyctimene | minutus | Nyctimene minutus | NA | NA | 199965.8 | 236.37 | 21.84 | NA | NA | 1 | 2 | NA | 0.756471 |
| Chiroptera | Hipposideridae | Hipposideros | lekaguli | Hipposideros lekaguli | 31.08 | NA | 200167.3 | 218.84 | 24.795 | NA | 1 | 1 | 2 | 3 | 0.244431 |
| Chiroptera | Pteropodidae | Plerotes | anchietae | Plerotes anchietae | NA | NA | 200249.8 | 105.99 | 21.254 | NA | NA | 1 | 2 | NA | 0.300586 |
| Rodentia | Cricetidae | Myodes | californicus | Myodes californicus | 18.3 | 3.1 | 200498.4 | 71.63 | 8.701 | NA | NA | NA | NA | NA | 0.013359 |
| Rodentia | Cricetidae | Oligoryzomys | andinus | Oligoryzomys andinus | 25.2 | NA | 200929.6 | 72.87 | 10.964 | NA | NA | NA | NA | NA | 0.013332 |
| Chiroptera | Molossidae | Tadarida | latouchei | Tadarida latouchei | NA | NA | 200951.1 | 66.77 | 12.632 | NA | NA | NA | NA | NA | 0.258238 |
| Chiroptera | Vespertilionidae | Laephotis | angolensis | Laephotis angolensis | NA | NA | 200961.4 | 105.97 | 21.379 | NA | NA | 1 | 2 | NA | 0.302833 |
| Chiroptera | Vespertilionidae | Hypsugo | ariel | Hypsugo ariel | NA | NA | 201108.3 | 12.18 | 25.871 | NA | NA | 1 | 2 | NA | 0.295873 |
| Rodentia | Echimyidae | Proechimys | guairae | Proechimys guairae | 349.28 | 2.79 | 201471.7 | 98.28 | 24.846 | 1 | 2 | 1 | 1 | 1 | 0.020662 |
| Chiroptera | Mormoopidae | Pteronotus | quadridens | Pteronotus quadridens | 5.64 | 1.21 | 201573.1 | 106.77 | 24.598 | NA | 1 | 1 | 2 | 3 | 0.020245 |
| Primates | Atelidae | Ateles | fusciceps | Ateles fusciceps | 9067.94 | 1.01 | 201579.7 | 220.34 | 24.081 | 3 | 2 | 1 | 2 | 1 | 1.562686 |
| Rodentia | Cricetidae | Microtus | gerbei | Microtus gerbei | NA | NA | 202038.8 | 66.07 | 10.531 | NA | NA | NA | NA | NA | 0.013963 |
| Chiroptera | Mormoopidae | Mormoops | blainvillei | Mormoops blainvillei | 8.69 | NA | 202317.8 | 106.77 | 24.598 | NA | 1 | 1 | 2 | 3 | 0.023649 |
| Chiroptera | Rhinolophidae | Rhinolophus | hillorum | Rhinolophus hillorum | NA | NA | 202441.4 | 145.65 | 24.407 | NA | NA | NA | NA | NA | 0.470404 |
| Chiroptera | Pteropodidae | Pteropus | griseus | Pteropus griseus | NA | NA | 202738.6 | 212.28 | 21.997 | NA | NA | NA | NA | NA | 0.294088 |
| Rodentia | Spalacidae | Spalax | zemni | Spalax zemni | NA | NA | 203090.8 | 41.92 | 6.872 | NA | NA | NA | NA | NA | 1.049261 |
| Chiroptera | Phyllostomidae | Monophyllus | redmani | Monophyllus redmani | 8.79 | 0.99 | 203276.2 | 106.77 | 24.598 | NA | 3 | 1 | 2 | 2 | 0.017548 |
| Primates | Cercopithecidae | Presbytis | frontata | Presbytis frontata | 6096.6 | NA | 203400 | 249.94 | 24.076 | 3 | NA | NA | NA | NA | 0.710834 |
| Rodentia | Calomyscidae | Calomyscus | elburzensis | Calomyscus elburzensis | NA | NA | 204039.7 | 28.57 | 11.802 | NA | NA | NA | NA | NA | 0.018006 |
| Macroscelidea | Macroscelididae | Elephantulus | fuscipes | Elephantulus fuscipes | 57 | 0.96 | 204288.7 | 120.79 | 23.735 | NA | NA | NA | NA | NA | 0.297658 |
| Rodentia | Caviidae | Microcavia | niata | Microcavia niata | 254.67 | 3.16 | 204842.3 | 43.26 | 3.34 | NA | 2 | NA | NA | 1 | 0.01916 |
| Rodentia | Cricetidae | Rhipidomys | wetzeli | Rhipidomys wetzeli | 89 | NA | 205046.8 | 179.27 | 22.711 | 1 | NA | 1 | 2 | NA | 0.015785 |
| Dasyuromorphia | Dasyuridae | Sminthopsis | griseoventer | Sminthopsis griseoventer | 17.5 | NA | 205150.9 | 48.02 | 15.95 | 2 | NA | 1 | 1 | NA | 0.018653 |
| Diprotodontia | Pseudocheiridae | Pseudochirops | cupreus | Pseudochirops cupreus | 1764.08 | 1.01 | 205225 | 265.72 | 20.227 | 2 | 2 | 2 | 2 | 1 | 0.022331 |
| Primates | Cercopithecidae | Papio | papio | Papio papio | 18026.05 | 1.01 | 205349.1 | 170.11 | 26.322 | 3 | NA | NA | NA | NA | 0.160275 |
| Rodentia | Cricetidae | Calomys | sorellus | Calomys sorellus | 20 | NA | 205439.9 | 62.31 | 7.089 | NA | NA | NA | NA | NA | 0.016197 |
| Lagomorpha | Ochotonidae | Ochotona | gloveri | Ochotona gloveri | NA | NA | 205591.1 | 152.24 | -1.618 | NA | NA | 1 | 1 | NA | 0.016405 |
| Rodentia | Muridae | Notomys | fuscus | Notomys fuscus | 39.28 | 3.07 | 205961.5 | 15.52 | 20.956 | 1 | 5 | 2 | 1 | 2 | 0.950872 |
| Rodentia | Cricetidae | Microtus | oregoni | Microtus oregoni | 20.35 | 3.32 | 206882.6 | 91.61 | 7.396 | NA | 2 | 2 | 1 | 1 | 0.014923 |
| Rodentia | Cricetidae | Auliscomys | boliviensis | Auliscomys boliviensis | 70.91 | 3.4 | 207032.8 | 54.21 | 7.172 | 3 | NA | 1 | 1 | NA | 0.016108 |
| Chiroptera | Molossidae | Tomopeas | ravus | Tomopeas ravus | NA | NA | 207270.6 | 33.35 | 10.338 | NA | NA | 2 | 2 | NA | 1.281259 |
| Soricomorpha | Soricidae | Sorex | bendirii | Sorex bendirii | 15.76 | NA | 207366.9 | 90.96 | 7.729 | NA | 1 | 3 | 2 | 3 | 0.016654 |
| Rodentia | Muridae | Dipodillus | harwoodi | Dipodillus harwoodi | NA | NA | 207700.8 | 71.33 | 20.858 | NA | NA | NA | NA | NA | 0.022232 |
| Afrosoricida | Chrysochloridae | Calcochloris | obtusirostris | Calcochloris obtusirostris | 24.05 | 2 | 209272.4 | 67.42 | 23.162 | NA | 1 | 1 | 1 | 3 | 0.024087 |
| Carnivora | Herpestidae | Herpestes | semitorquatus | Herpestes semitorquatus | NA | NA | 209629.9 | 273.83 | 24.151 | NA | NA | 1 | 1 | NA | 0.334378 |
| Primates | Cercopithecidae | Trachypithecus | obscurus | Trachypithecus obscurus | 7247.88 | 1.01 | 209688.4 | 201.98 | 25.504 | 3 | 1 | NA | NA | 1 | 0.115059 |
| Diprotodontia | Macropodidae | Petrogale | assimilis | Petrogale assimilis | 4618.88 | 1 | 209697.6 | 49.03 | 23.125 | NA | NA | 1 | 1 | NA | 0.016833 |
| Diprotodontia | Pseudocheiridae | Pseudochirops | corinnae | Pseudochirops corinnae | 1123 | 1.02 | 209842 | 272.67 | 19.911 | 2 | 2 | 1 | 2 | 1 | 0.22379 |
| Rodentia | Muridae | Gerbillus | nigeriae | Gerbillus nigeriae | 29.7 | NA | 210504.7 | 40.43 | 28.298 | NA | NA | NA | NA | NA | 0.022232 |
| Diprotodontia | Petauridae | Dactylopsila | palpator | Dactylopsila palpator | 369.87 | 1.02 | 210628.5 | 265.73 | 19.903 | 1 | 7 | 2 | 2 | 2 | 0.021283 |
| Carnivora | Viverridae | Genetta | cristata | Genetta cristata | 1863.23 | 2.5 | 210874.7 | 179.62 | 24.195 | 1 | 2 | NA | NA | 3 | 0.770902 |
| Rodentia | Echimyidae | Proechimys | semispinosus | Proechimys semispinosus | 353.32 | 2.84 | 211449.2 | 218.21 | 24.649 | 1 | 4 | 1 | 1 | 2 | 0.020662 |
| Chiroptera | Vespertilionidae | Glauconycteris | gleni | Glauconycteris gleni | 10.81 | NA | 211544.6 | 125.05 | 23.658 | NA | NA | 1 | 2 | NA | 0.250694 |
| Rodentia | Gliridae | Graphiurus | christyi | Graphiurus christyi | NA | 4.99 | 211548.5 | 145.59 | 23.302 | 1 | 4 | 2 | 2 | 2 | 0.019853 |
| Peramelemorphia | Peramelidae | Microperoryctes | longicauda | Microperoryctes longicauda | 542.07 | 1.03 | 211571.4 | 267.77 | 19.811 | NA | 2 | NA | NA | 2 | 0.01976 |
| Chiroptera | Emballonuridae | Balantiopteryx | io | Balantiopteryx io | 3.97 | 1 | 211758.2 | 144.32 | 23.945 | 1 | NA | 1 | 2 | NA | 1.069514 |
| Diprotodontia | Tarsipedidae | Tarsipes | rostratus | Tarsipes rostratus | 9.66 | 2.93 | 211836.3 | 45.23 | 16.077 | 2 | 1 | 2 | 2 | 1 | 0.024014 |
| Rodentia | Cricetidae | Reithrodontomys | zacatecae | Reithrodontomys zacatecae | NA | NA | 212008.5 | 51.24 | 14.977 | 1 | NA | 2 | 2 | NA | 0.018428 |
| Rodentia | Muridae | Apodemus | epimelas | Apodemus epimelas | NA | NA | 212820.6 | 75.11 | 9.818 | NA | NA | NA | NA | NA | 0.017279 |
| Rodentia | Muridae | Niviventer | eha | Niviventer eha | NA | 4.86 | 212869.4 | 182.18 | 9.468 | NA | NA | NA | NA | NA | 0.021458 |
| Rodentia | Cricetidae | Eligmodontia | morgani | Eligmodontia morgani | 16.5 | NA | 213111.6 | 37.02 | 7.045 | NA | NA | NA | NA | NA | 0.014905 |
| Chiroptera | Rhinolophidae | Rhinolophus | capensis | Rhinolophus capensis | 12.87 | 0.99 | 213936.8 | 29.75 | 15.955 | NA | NA | 1 | 2 | NA | 0.012207 |
| Rodentia | Cricetidae | Microtus | townsendii | Microtus townsendii | 52.02 | 5.06 | 214060.2 | 108.76 | 7.285 | 2 | 2 | 3 | 1 | 1 | 0.014365 |
| Artiodactyla | Bovidae | Oryx | dammah | Oryx dammah | 199799 | 1 | 214297.2 | 15.15 | 28.102 | NA | 5 | 1 | 1 | 1 | 1.419758 |
| Carnivora | Eupleridae | Cryptoprocta | ferox | Cryptoprocta ferox | 9500 | 2.98 | 214312.1 | 125.27 | 22.198 | 2 | 2 | 3 | 2 | 3 | 1.228015 |
| Soricomorpha | Soricidae | Sylvisorex | granti | Sylvisorex granti | NA | 1.78 | 215046.6 | 85.53 | 19.448 | NA | NA | NA | NA | NA | 0.017503 |
| Rodentia | Cricetidae | Hodomys | alleni | Hodomys alleni | 367.59 | 1.39 | 215347.2 | 93.64 | 20.252 | NA | NA | 2 | 1 | NA | 0.019722 |
| Chiroptera | Molossidae | Otomops | madagascariensis | Otomops madagascariensis | NA | NA | 215525.9 | 92.01 | 23.923 | NA | NA | NA | NA | NA | 0.016584 |
| Peramelemorphia | Peramelidae | Peroryctes | raffrayana | Peroryctes raffrayana | 905.63 | 1.27 | 215767.6 | 263.55 | 20.246 | 1 | 1 | 1 | 1 | 1 | 0.018067 |
| Rodentia | Muridae | Otomys | tropicalis | Otomys tropicalis | NA | 1.29 | 216009.1 | 87.55 | 19.89 | NA | NA | NA | NA | NA | 0.017181 |
| Chiroptera | Vespertilionidae | Murina | florium | Murina florium | 4.41 | NA | 216194.1 | 217.06 | 23.017 | NA | NA | 1 | 2 | NA | 0.015749 |
| Rodentia | Ctenomyidae | Ctenomys | talarum | Ctenomys talarum | 140.84 | 4.34 | 216393.6 | 67.75 | 14.536 | 1 | 4 | 1 | 1 | 1 | 0.016918 |
| Lagomorpha | Leporidae | Lepus | coreanus | Lepus coreanus | NA | NA | 216940.6 | 88.44 | 5.871 | NA | NA | NA | NA | NA | 0.013895 |
| Rodentia | Sciuridae | Marmota | broweri | Marmota broweri | NA | NA | 217532 | 12.79 | -14.348 | NA | NA | NA | NA | NA | 0.010285 |
| Diprotodontia | Macropodidae | Dendrolagus | dorianus | Dendrolagus dorianus | 8976.58 | 1.01 | 217597.1 | 272.01 | 19.463 | 2 | 3 | 2 | 2 | 2 | 1.238139 |
| Rodentia | Sciuridae | Spermophilus | canus | Spermophilus canus | NA | NA | 217875.1 | 22.61 | 7.109 | NA | NA | NA | NA | NA | 0.012833 |
| Rodentia | Nesomyidae | Dendromus | insignis | Dendromus insignis | NA | NA | 218282.1 | 76.36 | 16.553 | NA | NA | NA | NA | NA | 0.022547 |
| Rodentia | Cricetidae | Rhipidomys | venezuelae | Rhipidomys venezuelae | 89.99 | NA | 218910 | 89.6 | 23.699 | 1 | NA | 1 | 2 | NA | 0.015785 |
| Chiroptera | Hipposideridae | Hipposideros | obscurus | Hipposideros obscurus | 9.6 | NA | 219776.4 | 199.6 | 24.124 | NA | 1 | 1 | 2 | 3 | 0.024482 |
| Artiodactyla | Cervidae | Rusa | marianna | Rusa marianna | 49457.61 | NA | 220497.9 | 206.29 | 23.959 | NA | NA | NA | NA | NA | 0.824584 |
| Diprotodontia | Burramyidae | Cercartetus | caudatus | Cercartetus caudatus | 23.05 | 2.37 | 221312 | 233.35 | 20.307 | 1 | 3 | 3 | 2 | 2 | 0.022655 |
| Soricomorpha | Talpidae | Neurotrichus | gibbsii | Neurotrichus gibbsii | 9.56 | 2.99 | 221424 | 93.39 | 7.745 | 2 | 4 | 2 | 1 | 2 | 0.025404 |
| Rodentia | Cricetidae | Chibchanomys | trichotis | Chibchanomys trichotis | 49.99 | 0.97 | 222554.1 | 134.21 | 20.449 | NA | NA | NA | NA | NA | 0.308426 |
| Carnivora | Viverridae | Genetta | piscivora | Genetta piscivora | 1648.05 | 0.96 | 222754.6 | 144.85 | 23.626 | 1 | 1 | 2 | 1 | 3 | 0.309915 |
| Rodentia | Sciuridae | Sciurus | yucatanensis | Sciurus yucatanensis | 225 | 3 | 222990.2 | 115.46 | 24.661 | 3 | 1 | 2 | 2 | 1 | 0.011813 |
| Artiodactyla | Cervidae | Pudu | puda | Pudu puda | 9642.62 | 1.22 | 223036.9 | 109.54 | 6.055 | 2 | 4 | 1 | 1 | 1 | 0.596515 |
| Chiroptera | Vespertilionidae | Scotorepens | orion | Scotorepens orion | 11.83 | NA | 223272.9 | 75.58 | 13.864 | NA | NA | NA | NA | NA | 0.017002 |
| Didelphimorphia | Didelphidae | Thylamys | macrurus | Thylamys macrurus | 34.43 | NA | 223583.7 | 106.08 | 22.835 | 1 | NA | NA | NA | NA | 0.243048 |
| Diprotodontia | Macropodidae | Thylogale | stigmatica | Thylogale stigmatica | 4511.47 | 1 | 223751.1 | 107.23 | 20.836 | 1 | 3 | 1 | 1 | 1 | 0.01326 |
| Chiroptera | Pteropodidae | Eonycteris | robusta | Eonycteris robusta | 78.36 | NA | 223952 | 201.68 | 24.128 | NA | NA | NA | NA | NA | 0.177355 |
| Chiroptera | Rhinolophidae | Rhinolophus | rufus | Rhinolophus rufus | NA | NA | 224260 | 204.58 | 24.016 | NA | NA | NA | NA | NA | 0.122072 |
| Rodentia | Heteromyidae | Chaetodipus | baileyi | Chaetodipus baileyi | 28.44 | 3.68 | 225471.2 | 22.76 | 19.761 | NA | 2 | NA | NA | 2 | 0.017113 |
| Chiroptera | Pteropodidae | Dobsonia | viridis | Dobsonia viridis | 240.66 | NA | 225635.4 | 231.15 | 22.2 | NA | NA | 1 | 2 | NA | 0.010826 |
| Chiroptera | Rhinolophidae | Rhinolophus | subrufus | Rhinolophus subrufus | NA | NA | 225646.9 | 200.86 | 24.035 | NA | 1 | 1 | 2 | 3 | 0.195316 |
| Soricomorpha | Soricidae | Crocidura | grassei | Crocidura grassei | NA | NA | 225706.5 | 138.9 | 23.983 | NA | NA | NA | NA | NA | 0.01789 |
| Soricomorpha | Soricidae | Myosorex | cafer | Myosorex cafer | 12.3 | 2.99 | 225919.8 | 78.62 | 18.579 | 2 | 4 | 1 | 1 | 2 | 0.020296 |
| Rodentia | Heteromyidae | Microdipodops | megacephalus | Microdipodops megacephalus | 12.3 | 3.94 | 226286.8 | 21.26 | 7.185 | 1 | 2 | 2 | 1 | 2 | 0.019028 |
| Rodentia | Muridae | Ammodillus | imbellis | Ammodillus imbellis | 49.99 | NA | 226420.6 | 14.7 | 25.841 | NA | NA | NA | NA | NA | 0.357018 |
| Rodentia | Cricetidae | Microtus | duodecimcostatus | Microtus duodecimcostatus | 22.71 | 2.51 | 226553.2 | 62.9 | 12.176 | NA | NA | NA | NA | NA | 0.013216 |
| Rodentia | Cricetidae | Neotoma | stephensi | Neotoma stephensi | 149.22 | 1.06 | 227258.8 | 24.91 | 12.018 | NA | 3 | NA | NA | 2 | 0.015637 |
| Chiroptera | Vespertilionidae | Pipistrellus | endoi | Pipistrellus endoi | NA | NA | 227486.1 | 138.47 | 9.948 | NA | NA | 1 | 2 | NA | 1.160029 |
| Rodentia | Cricetidae | Necromys | urichi | Necromys urichi | 44.84 | 5.18 | 229420.4 | 145.69 | 23.428 | 2 | 4 | 1 | 1 | 2 | 0.013492 |
| Didelphimorphia | Didelphidae | Monodelphis | iheringi | Monodelphis iheringi | 112 | NA | 229715.8 | 134.74 | 17.219 | 1 | 1 | 1 | 1 | 3 | 0.386822 |
| Artiodactyla | Bovidae | Kobus | leche | Kobus leche | 88645.04 | 1 | 229752.1 | 84.65 | 21.594 | 2 | 2 | 2 | 1 | 1 | 0.018987 |
| Rodentia | Muridae | Paramelomys | mollis | Paramelomys mollis | 90.05 | 1.69 | 231082.8 | 255.74 | 21.334 | NA | 3 | 2 | 1 | 1 | 0.020235 |
| Chiroptera | Pteropodidae | Harpyionycteris | whiteheadi | Harpyionycteris whiteheadi | 135.48 | 1 | 231112.2 | 200.92 | 24.116 | NA | 1 | 1 | 2 | 1 | 0.018868 |
| Rodentia | Cricetidae | Kunsia | tomentosus | Kunsia tomentosus | 115.6 | NA | 231160.9 | 127.07 | 24.193 | NA | NA | 2 | 1 | NA | 0.015219 |
| Artiodactyla | Bovidae | Cephalophus | ogilbyi | Cephalophus ogilbyi | 18386.42 | 1 | 231511.2 | 190.65 | 25.154 | 3 | NA | NA | NA | NA | 0.012654 |
| Chiroptera | Pteropodidae | Pteropus | pumilus | Pteropus pumilus | 184.05 | NA | 232023.9 | 200.49 | 24.098 | NA | 1 | 1 | 2 | 1 | 0.183805 |
| Rodentia | Muridae | Zyzomys | pedunculatus | Zyzomys pedunculatus | 100 | NA | 233916 | 21.64 | 21.572 | NA | NA | NA | NA | NA | 1.733534 |
| Diprotodontia | Acrobatidae | Distoechurus | pennatus | Distoechurus pennatus | 52.74 | 1.31 | 234473.3 | 286 | 21.356 | 1 | 4 | 1 | 2 | 2 | 0.021751 |
| Rodentia | Sciuridae | Callosciurus | inornatus | Callosciurus inornatus | NA | NA | 235249.2 | 129.13 | 19.819 | NA | NA | NA | NA | NA | 0.020081 |
| Rodentia | Cricetidae | Microtus | lusitanicus | Microtus lusitanicus | NA | 2.09 | 235534.5 | 61.3 | 12.399 | NA | NA | NA | NA | NA | 0.013216 |
| Rodentia | Sciuridae | Sciurotamias | forresti | Sciurotamias forresti | NA | NA | 235812.3 | 115.36 | 11.577 | NA | NA | NA | NA | NA | 0.020428 |
| Rodentia | Dipodidae | Sicista | napaea | Sicista napaea | 10 | 3.49 | 236088.4 | 33.22 | -2.512 | NA | 3 | NA | NA | 2 | 0.02343 |
| Rodentia | Cricetidae | Oecomys | superans | Oecomys superans | 73.4 | NA | 236984.3 | 230.78 | 25.172 | NA | NA | NA | NA | NA | 0.012792 |
| Artiodactyla | Bovidae | Capricornis | sumatraensis | Capricornis sumatraensis | 110942.2 | 1.22 | 237174.9 | 227.32 | 24.668 | NA | 2 | NA | NA | 1 | 0.634642 |
| Chiroptera | Vespertilionidae | Myotis | macrotarsus | Myotis macrotarsus | 12.64 | NA | 237568.8 | 202.61 | 24.104 | NA | NA | 1 | 2 | NA | 0.162084 |
| Chiroptera | Pteropodidae | Micropteropus | intermedius | Micropteropus intermedius | NA | NA | 237569.1 | 129.94 | 23.798 | NA | NA | 1 | 2 | NA | 0.159372 |
| Artiodactyla | Suidae | Sus | philippensis | Sus philippensis | 190792.3 | 6.24 | 237935 | 208.67 | 24.084 | 2 | 6 | NA | NA | 2 | 0.925864 |
| Rodentia | Ctenomyidae | Ctenomys | sericeus | Ctenomys sericeus | 400 | NA | 237955.2 | 11.71 | 9.526 | NA | 3 | 1 | 1 | 1 | 0.270692 |
| Rodentia | Sciuridae | Pteromyscus | pulverulentus | Pteromyscus pulverulentus | 251.3 | 1.28 | 238644.3 | 228.86 | 24.746 | NA | NA | 1 | 2 | NA | 1.120619 |
| Rodentia | Muridae | Rattus | steini | Rattus steini | 151.17 | 3.65 | 238700.4 | 275.17 | 21.096 | NA | 4 | NA | NA | 2 | 0.016589 |
| Rodentia | Heteromyidae | Heteromys | anomalus | Heteromys anomalus | 69.06 | 2.83 | 238957 | 85.6 | 24.384 | 1 | 3 | 1 | 1 | 2 | 0.016114 |
| Chiroptera | Molossidae | Mops | mops | Mops mops | 31.12 | NA | 239427.4 | 237.22 | 24.653 | NA | 1 | 1 | 2 | 3 | 0.173618 |
| Rodentia | Muridae | Rattus | everetti | Rattus everetti | 253.53 | NA | 239437.4 | 208.14 | 24.12 | 2 | 5 | NA | NA | 2 | 0.013963 |
| Chiroptera | Hipposideridae | Hipposideros | muscinus | Hipposideros muscinus | NA | NA | 239509.3 | 268.65 | 22.675 | NA | NA | 1 | 2 | NA | 0.293954 |
| Soricomorpha | Talpidae | Uropsilus | soricipes | Uropsilus soricipes | 16 | NA | 240300.1 | 92.15 | 5.906 | NA | 1 | 1 | 1 | 3 | 0.023961 |
| Chiroptera | Rhinolophidae | Rhinolophus | virgo | Rhinolophus virgo | 6.06 | NA | 240685.2 | 200.83 | 24.211 | NA | 1 | 1 | 2 | 3 | 0.012207 |
| Chiroptera | Hipposideridae | Triaenops | rufus | Triaenops rufus | 9.1 | NA | 241046.3 | 148.85 | 22.069 | 1 | 1 | NA | NA | 3 | 0.019659 |
| Chiroptera | Vespertilionidae | Myotis | schaubi | Myotis schaubi | NA | NA | 241089.1 | 51.92 | 4.834 | NA | NA | 1 | 2 | NA | 0.239978 |
| Rodentia | Muridae | Pseudomys | albocinereus | Pseudomys albocinereus | 30.5 | 3.89 | 241382.5 | 38.5 | 16.506 | NA | NA | NA | NA | NA | 0.021649 |
| Soricomorpha | Soricidae | Crocidura | nana | Crocidura nana | NA | NA | 241737.5 | 46.27 | 20.385 | NA | NA | NA | NA | NA | 0.01789 |
| Chiroptera | Phyllostomidae | Rhinophylla | alethina | Rhinophylla alethina | NA | NA | 241884.1 | 198.14 | 20.423 | NA | 1 | 1 | 2 | 1 | 0.197769 |
| Rodentia | Geomyidae | Geomys | pinetis | Geomys pinetis | 201.15 | 1.68 | 242106.9 | 106.58 | 19.679 | NA | 3 | 2 | 1 | 1 | 0.018155 |
| Chiroptera | Pteropodidae | Haplonycteris | fischeri | Haplonycteris fischeri | 18.25 | 1 | 242390.3 | 202.1 | 24.16 | NA | 3 | 1 | 2 | 1 | 0.017247 |
| Rodentia | Muridae | Pogonomys | loriae | Pogonomys loriae | 95.39 | 2.68 | 242498.1 | 291.84 | 21.522 | 1 | 3 | 3 | 2 | 1 | 0.017655 |
| Rodentia | Echimyidae | Proechimys | quadruplicatus | Proechimys quadruplicatus | 284.99 | NA | 242986.1 | 239.8 | 25.444 | NA | NA | NA | NA | NA | 0.020662 |
| Chiroptera | Phyllostomidae | Lophostoma | evotis | Lophostoma evotis | 20.58 | 1 | 243008.9 | 150.71 | 24.483 | NA | 2 | 1 | 2 | 2 | 0.017784 |
| Chiroptera | Pteropodidae | Rousettus | madagascariensis | Rousettus madagascariensis | 65.72 | 1.22 | 243394.8 | 151.39 | 22.067 | 2 | 2 | 1 | 2 | 1 | 0.102753 |
| Chiroptera | Phyllostomidae | Scleronycteris | ega | Scleronycteris ega | 49.54 | NA | 243504 | 213.31 | 25.167 | NA | NA | 1 | 2 | NA | 0.015604 |
| Rodentia | Muridae | Anisomys | imitator | Anisomys imitator | 510.5 | NA | 243507.6 | 265.82 | 20.743 | NA | 3 | 2 | 2 | 1 | 0.022938 |
| Artiodactyla | Bovidae | Raphicerus | melanotis | Raphicerus melanotis | 10503.14 | NA | 243785.1 | 33.18 | 15.72 | 1 | 2 | 1 | 1 | 1 | 0.016244 |
| Rodentia | Muridae | Meriones | zarudnyi | Meriones zarudnyi | NA | NA | 243895.9 | 18.24 | 13.031 | NA | NA | NA | NA | NA | 0.288256 |
| Rodentia | Muridae | Pseudomys | chapmani | Pseudomys chapmani | 10.95 | NA | 244137.1 | 21.5 | 23.458 | NA | NA | NA | NA | NA | 0.021649 |
| Artiodactyla | Cervidae | Pudu | mephistophiles | Pudu mephistophiles | 9599.96 | 1 | 244304.2 | 151.56 | 20.198 | NA | NA | NA | NA | NA | 0.596515 |
| Primates | Hominidae | Pongo | pygmaeus | Pongo pygmaeus | 53408.29 | 1.07 | 245264 | 258.7 | 23.771 | 3 | 5 | 2 | 2 | 2 | 1.396664 |
| Rodentia | Aplodontiidae | Aplodontia | rufa | Aplodontia rufa | 806.21 | 2.46 | 245595.7 | 80.7 | 6.619 | NA | 2 | 1 | 1 | 1 | 0.028074 |
| Chiroptera | Vespertilionidae | Pipistrellus | angulatus | Pipistrellus angulatus | 3.35 | NA | 245803 | 271.39 | 23.019 | NA | NA | NA | NA | NA | 0.015988 |
| Rodentia | Cricetidae | Eothenomys | chinensis | Eothenomys chinensis | NA | NA | 245944.7 | 135.27 | 7.473 | NA | NA | NA | NA | NA | 0.011119 |
| Rodentia | Cricetidae | Akodon | varius | Akodon varius | 40 | NA | 245945.2 | 74.3 | 18.174 | NA | 1 | 2 | 1 | 3 | 0.146488 |
| Rodentia | Muridae | Cremnomys | cutchicus | Cremnomys cutchicus | NA | 5.23 | 246119.1 | 60.28 | 25.77 | NA | NA | NA | NA | NA | 0.017724 |
| Rodentia | Muridae | Rattus | verecundus | Rattus verecundus | 97.02 | 2.15 | 246219.2 | 286.43 | 21.23 | 3 | 3 | 2 | 1 | 2 | 0.020217 |
| Rodentia | Muridae | Gerbillurus | vallinus | Gerbillurus vallinus | 35.24 | 3.87 | 247919.8 | 9.88 | 17.98 | 1 | NA | 2 | 1 | NA | 0.017972 |
| Afrosoricida | Tenrecidae | Tenrec | ecaudatus | Tenrec ecaudatus | 887.59 | 16.89 | 248061.3 | 123.97 | 22.169 | 2 | 3 | 1 | 1 | 2 | 0.025327 |
| Chiroptera | Pteropodidae | Ptenochirus | jagori | Ptenochirus jagori | 79.18 | 1 | 248720.1 | 199.19 | 24.188 | NA | 1 | 1 | 2 | 1 | 0.015563 |
| Chiroptera | Vespertilionidae | Kerivoula | cuprosa | Kerivoula cuprosa | NA | 0.98 | 249700.7 | 125.85 | 22.765 | NA | NA | 1 | 2 | NA | 0.25096 |
| Chiroptera | Phyllostomidae | Choeroniscus | periosus | Choeroniscus periosus | NA | NA | 250151.5 | 213.36 | 21.697 | NA | NA | 1 | 2 | NA | 0.743836 |
| Rodentia | Sciuridae | Sciurus | nayaritensis | Sciurus nayaritensis | 697 | 2.43 | 250172.2 | 44.73 | 14.854 | 3 | 4 | 2 | 2 | 1 | 0.012612 |
| Rodentia | Cricetidae | Oxymycterus | paramensis | Oxymycterus paramensis | 41.99 | NA | 250661.8 | 73.9 | 12.004 | NA | 1 | 2 | 1 | 3 | 0.014623 |
| Rodentia | Muridae | Mus | saxicola | Mus saxicola | NA | NA | 250732.6 | 90.46 | 25.61 | NA | NA | NA | NA | NA | 0.0104 |
| Chiroptera | Pteropodidae | Nyctimene | cephalotes | Nyctimene cephalotes | 44.92 | 0.98 | 251382.8 | 220.07 | 22.829 | NA | NA | 1 | 2 | NA | 0.016172 |
| Soricomorpha | Soricidae | Scutisorex | somereni | Scutisorex somereni | 91.49 | 1.85 | 252190.8 | 128.58 | 22.268 | NA | NA | NA | NA | NA | 0.020364 |
| Rodentia | Cricetidae | Necromys | amoenus | Necromys amoenus | 27.09 | NA | 252648.9 | 120.69 | 14.746 | NA | NA | NA | NA | NA | 0.012975 |
| Afrosoricida | Chrysochloridae | Chrysochloris | stuhlmanni | Chrysochloris stuhlmanni | 55.76 | 0.96 | 252891.6 | 112.83 | 21.92 | 1 | 1 | 1 | 1 | 3 | 0.022697 |
| Rodentia | Cricetidae | Chinchillula | sahamae | Chinchillula sahamae | 169.74 | NA | 253693.9 | 90.56 | 4.576 | 1 | 3 | 1 | 1 | 2 | 0.019815 |
| Primates | Cebidae | Saguinus | labiatus | Saguinus labiatus | 508.46 | 1.84 | 253794.5 | 168.04 | 25.681 | 3 | NA | 1 | 2 | NA | 0.013615 |
| Rodentia | Cricetidae | Sigmodon | mascotensis | Sigmodon mascotensis | 120 | NA | 253914.5 | 99.85 | 20.518 | NA | NA | NA | NA | NA | 0.014461 |
| Rodentia | Dipodidae | Sicista | strandi | Sicista strandi | NA | NA | 254890.4 | 36.98 | 7.963 | NA | NA | NA | NA | NA | 0.02343 |
| Artiodactyla | Cervidae | Rucervus | duvaucelii | Rucervus duvaucelii | 171223.9 | 1 | 255737.1 | 101.07 | 24.548 | 2 | 2 | 2 | 1 | 1 | 0.824584 |
| Didelphimorphia | Didelphidae | Marmosa | tyleriana | Marmosa tyleriana | 32.4 | 3 | 256123.4 | 187.92 | 23.056 | 1 | NA | NA | NA | NA | 0.399738 |
| Afrosoricida | Chrysochloridae | Amblysomus | hottentotus | Amblysomus hottentotus | 62.6 | 1.86 | 256161.8 | 55.31 | 14.614 | 2 | 2 | 2 | 1 | 2 | 0.023245 |
| Rodentia | Cricetidae | Microryzomys | altissimus | Microryzomys altissimus | 13.49 | NA | 256430.2 | 130.5 | 15.71 | NA | NA | NA | NA | NA | 0.016518 |
| Lagomorpha | Leporidae | Lepus | alleni | Lepus alleni | 3930 | 1.96 | 258607.1 | 31.17 | 19.933 | 2 | 2 | NA | NA | 1 | 0.011923 |
| Primates | Cercopithecidae | Miopithecus | talapoin | Miopithecus talapoin | 1248.86 | 1.01 | 258766.2 | 104.31 | 23.171 | 3 | 3 | 1 | 2 | 2 | 0.019029 |
| Rodentia | Muridae | Gerbillus | watersi | Gerbillus watersi | NA | NA | 259090.5 | 15.75 | 27.191 | NA | NA | NA | NA | NA | 0.022232 |
| Chiroptera | Pteropodidae | Nyctimene | cyclotis | Nyctimene cyclotis | 48.83 | NA | 259281.5 | 290.96 | 21.749 | NA | NA | 1 | 2 | NA | 0.234303 |
| Soricomorpha | Talpidae | Parascaptor | leucura | Parascaptor leucura | NA | NA | 259508.4 | 163.94 | 13.686 | NA | 1 | NA | NA | 3 | 0.024929 |
| Diprotodontia | Phalangeridae | Phalanger | gymnotis | Phalanger gymnotis | 2597.89 | 1.01 | 260036.9 | 267.02 | 22.423 | 1 | 4 | 3 | 2 | 2 | 0.017669 |
| Rodentia | Muridae | Hylomyscus | carillus | Hylomyscus carillus | NA | NA | 260098.8 | 100.9 | 21.294 | NA | NA | NA | NA | NA | 0.015329 |
| Chiroptera | Vespertilionidae | Kerivoula | muscina | Kerivoula muscina | 5.29 | NA | 260157.1 | 300.77 | 22.018 | NA | NA | 1 | 2 | NA | 0.015685 |
| Diprotodontia | Phalangeridae | Phalanger | orientalis | Phalanger orientalis | 2487.5 | 1.71 | 260829.7 | 250.97 | 23.868 | 1 | 2 | 2 | 2 | 1 | 0.016412 |
| Rodentia | Muridae | Myotomys | sloggetti | Myotomys sloggetti | 106.25 | 1.44 | 261475 | 42.83 | 14.844 | NA | NA | NA | NA | NA | 0.019294 |
| Carnivora | Felidae | Catopuma | badia | Catopuma badia | 3429.52 | NA | 261512.2 | 278.55 | 22.789 | NA | NA | 1 | 1 | NA | 1.228595 |
| Afrosoricida | Tenrecidae | Setifer | setosus | Setifer setosus | 263.22 | 3.24 | 261594.5 | 125.02 | 22.117 | 1 | 2 | 1 | 1 | 2 | 0.02357 |
| Chiroptera | Pteropodidae | Acerodon | jubatus | Acerodon jubatus | 1087.04 | 1 | 261660.7 | 202.52 | 24.233 | NA | 2 | 1 | 2 | 1 | 1.146688 |
| Primates | Lorisidae | Nycticebus | pygmaeus | Nycticebus pygmaeus | 342.32 | 1.75 | 262116.8 | 149.9 | 21.09 | NA | NA | NA | NA | NA | 1.139771 |
| Primates | Cercopithecidae | Cercopithecus | campbelli | Cercopithecus campbelli | 3627.73 | 1.02 | 262404.6 | 226.67 | 25.298 | 3 | 3 | NA | NA | 1 | 0.014536 |
| Rodentia | Muridae | Dacnomys | millardi | Dacnomys millardi | NA | NA | 263125.3 | 146.11 | 12.689 | NA | NA | NA | NA | NA | 0.378963 |
| Chiroptera | Vespertilionidae | Rhogeessa | alleni | Rhogeessa alleni | NA | NA | 263308.7 | 85.02 | 18.226 | NA | NA | 1 | 2 | NA | 0.017337 |
| Chiroptera | Vespertilionidae | Myotis | pruinosus | Myotis pruinosus | NA | NA | 264536 | 143.02 | 10.43 | NA | NA | 1 | 2 | NA | 1.090991 |
| Rodentia | Sciuridae | Pteromys | momonga | Pteromys momonga | NA | NA | 264536 | 143.02 | 10.43 | NA | NA | NA | NA | NA | 0.016224 |
| Soricomorpha | Soricidae | Crocidura | thalia | Crocidura thalia | NA | NA | 265080.5 | 101.99 | 16.866 | NA | NA | NA | NA | NA | 0.01789 |
| Chiroptera | Vespertilionidae | Miniopterus | majori | Miniopterus majori | NA | NA | 265692.4 | 137.45 | 20.671 | NA | 1 | NA | NA | 3 | 0.01816 |
| Rodentia | Cricetidae | Microtus | montebelli | Microtus montebelli | 29.49 | 4.3 | 266801.3 | 143.02 | 10.43 | NA | NA | NA | NA | NA | 0.014649 |
| Rodentia | Sciuridae | Funambulus | tristriatus | Funambulus tristriatus | 134.62 | 2.64 | 266838.8 | 122.57 | 25.937 | NA | NA | NA | NA | NA | 0.018691 |
| Primates | Cebidae | Saguinus | inustus | Saguinus inustus | 803 | NA | 266932.8 | 246.37 | 24.939 | 3 | NA | 1 | 2 | NA | 0.016555 |
| Rodentia | Cricetidae | Cansumys | canus | Cansumys canus | NA | NA | 267130.8 | 58.53 | 10.316 | NA | NA | NA | NA | NA | 0.02395 |
| Chiroptera | Rhinolophidae | Rhinolophus | paradoxolophus | Rhinolophus paradoxolophus | 8.17 | NA | 267165.4 | 125.4 | 21.498 | NA | 1 | 1 | 2 | 3 | 0.012207 |
| Rodentia | Cricetidae | Graomys | domorum | Graomys domorum | 102 | NA | 267530.5 | 57.15 | 10.042 | NA | NA | 1 | 1 | NA | 0.014461 |
| Primates | Hylobatidae | Bunopithecus | hoolock | Bunopithecus hoolock | 6699 | 1.01 | 267771.5 | 153.6 | 17.457 | 3 | 5 | 1 | 2 | 2 | 1.244829 |
| Rodentia | Muridae | Praomys | delectorum | Praomys delectorum | 35.99 | 3.88 | 267885 | 73.64 | 21.998 | NA | NA | NA | NA | NA | 0.199529 |
| Chiroptera | Vespertilionidae | Hypsugo | imbricatus | Hypsugo imbricatus | 6.29 | NA | 268035 | 235.68 | 23.094 | NA | 1 | 1 | 2 | 3 | 0.018927 |
| Chiroptera | Molossidae | Eumops | maurus | Eumops maurus | NA | NA | 269128 | 140.51 | 25.808 | NA | NA | 1 | 2 | NA | 0.28728 |
| Rodentia | Sciuridae | Cynomys | leucurus | Cynomys leucurus | 963.76 | 5.4 | 269822.6 | 25.55 | 3.837 | 3 | 1 | 2 | 1 | 1 | 0.010973 |
| Macroscelidea | Macroscelididae | Elephantulus | fuscus | Elephantulus fuscus | NA | 1.39 | 269841.5 | 81.71 | 23.381 | NA | 1 | NA | NA | 3 | 0.297658 |
| Rodentia | Ctenodactylidae | Ctenodactylus | vali | Ctenodactylus vali | 173.6 | 2 | 269951.3 | 12.22 | 18.788 | 3 | 3 | NA | NA | 1 | 0.310779 |
| Chiroptera | Vespertilionidae | Rhogeessa | gracilis | Rhogeessa gracilis | NA | NA | 270246.6 | 97.53 | 20.341 | NA | NA | 1 | 2 | NA | 0.015531 |
| Rodentia | Sciuridae | Ammospermophilus | harrisii | Ammospermophilus harrisii | 126.57 | 6.32 | 270261.1 | 23.55 | 17.911 | 3 | 4 | NA | NA | 1 | 0.011721 |
| Soricomorpha | Soricidae | Chodsigoa | lamula | Chodsigoa lamula | NA | NA | 271133.8 | 132.56 | 3.691 | NA | NA | NA | NA | NA | 0.018741 |
| Lagomorpha | Ochotonidae | Ochotona | rutila | Ochotona rutila | NA | 4.1 | 271593.4 | 22.84 | 1.261 | NA | 2 | 1 | 1 | 1 | 0.016405 |
| Didelphimorphia | Didelphidae | Marmosa | rubra | Marmosa rubra | 62.99 | NA | 272062.1 | 192.7 | 26.174 | 1 | NA | NA | NA | NA | 0.399738 |
| Rodentia | Echimyidae | Isothrix | pagurus | Isothrix pagurus | 210 | NA | 272146.8 | 139.2 | 26.006 | 1 | NA | 1 | 2 | NA | 0.015737 |
| Artiodactyla | Bovidae | Capricornis | thar | Capricornis thar | NA | NA | 272208.2 | 151.07 | 10.444 | NA | NA | NA | NA | NA | 0.126928 |
| Rodentia | Sciuridae | Spermophilus | elegans | Spermophilus elegans | 324.45 | 5.75 | 272232.1 | 25.88 | 3.862 | 3 | 1 | 2 | 1 | 1 | 0.014759 |
| Chiroptera | Hipposideridae | Hipposideros | calcaratus | Hipposideros calcaratus | NA | NA | 272468.5 | 280.94 | 22.73 | NA | NA | 1 | 2 | NA | 0.021035 |
| Chiroptera | Vespertilionidae | Myotis | pequinius | Myotis pequinius | NA | NA | 273300.5 | 54.68 | 11.457 | NA | NA | 1 | 2 | NA | 0.015058 |
| Rodentia | Muridae | Pseudomys | gracilicaudatus | Pseudomys gracilicaudatus | 79.53 | 2.91 | 273612.8 | 89.69 | 19.193 | NA | NA | NA | NA | NA | 0.021649 |
| Rodentia | Cricetidae | Oecomys | speciosus | Oecomys speciosus | 73.4 | NA | 273664.2 | 131.56 | 24.349 | NA | NA | NA | NA | NA | 0.012792 |
| Artiodactyla | Bovidae | Tragelaphus | angasii | Tragelaphus angasii | 87616.76 | 1 | 274074.6 | 65.29 | 22.985 | 2 | 4 | 1 | 1 | 1 | 0.020401 |
| Rodentia | Muridae | Gerbillus | mesopotamiae | Gerbillus mesopotamiae | NA | NA | 274832 | 18.88 | 21.275 | NA | NA | NA | NA | NA | 0.022232 |
| Chiroptera | Vespertilionidae | Pipistrellus | adamsi | Pipistrellus adamsi | NA | NA | 276149.5 | 93.57 | 26.876 | NA | NA | NA | NA | NA | 0.015988 |
| Rodentia | Echimyidae | Echimys | semivillosus | Echimys semivillosus | 282.36 | NA | 276412.1 | 98.27 | 25.46 | 1 | 1 | 1 | 2 | 1 | 0.01783 |
| Rodentia | Echimyidae | Phyllomys | dasythrix | Phyllomys dasythrix | 260 | NA | 276503.5 | 130.13 | 17.546 | 1 | NA | 1 | 2 | NA | 0.01783 |
| Rodentia | Muridae | Conilurus | penicillatus | Conilurus penicillatus | 175.02 | 2 | 278072.3 | 89.82 | 27.104 | 1 | 5 | 2 | 2 | 2 | 0.181524 |
| Chiroptera | Vespertilionidae | Eptesicus | innoxius | Eptesicus innoxius | NA | NA | 278413.9 | 92.32 | 18.765 | NA | NA | 1 | 2 | NA | 0.144001 |
| Soricomorpha | Soricidae | Crocidura | floweri | Crocidura floweri | NA | NA | 279190.9 | 1.33 | 21.105 | NA | NA | NA | NA | NA | 0.286234 |
| Soricomorpha | Talpidae | Talpa | occidentalis | Talpa occidentalis | 49.47 | NA | 279205 | 53.93 | 13.067 | 2 | 1 | 2 | 1 | 3 | 0.023985 |
| Carnivora | Herpestidae | Galerella | flavescens | Galerella flavescens | 750 | NA | 279748.7 | 36.5 | 18.127 | NA | NA | 1 | 1 | NA | 0.020025 |
| Rodentia | Muridae | Mus | shortridgei | Mus shortridgei | NA | NA | 280801.9 | 118.1 | 23.975 | NA | NA | NA | NA | NA | 0.0104 |
| Rodentia | Muridae | Hybomys | lunaris | Hybomys lunaris | NA | NA | 281862.7 | 127 | 22.153 | NA | NA | NA | NA | NA | 0.91084 |
| Rodentia | Muridae | Diomys | crumpi | Diomys crumpi | NA | NA | 283011.2 | 179.24 | 23.81 | NA | NA | NA | NA | NA | 0.378963 |
| Rodentia | Sciuridae | Sciurus | lis | Sciurus lis | 264.3 | NA | 283013.8 | 145.01 | 10.561 | 3 | NA | 2 | 2 | NA | 0.010805 |
| Diprotodontia | Macropodidae | Petrogale | herberti | Petrogale herberti | NA | NA | 283187.1 | 59.62 | 19.715 | NA | NA | NA | NA | NA | 0.014704 |
| Rodentia | Cricetidae | Myodes | smithii | Myodes smithii | 34.94 | 4.1 | 283263.9 | 145.01 | 10.561 | NA | NA | NA | NA | NA | 0.01504 |
| Rodentia | Sciuridae | Aeromys | tephromelas | Aeromys tephromelas | 1187 | 1.07 | 283378.8 | 239.02 | 24.603 | 1 | NA | NA | NA | NA | 0.22395 |
| Rodentia | Cricetidae | Sigmodon | leucotis | Sigmodon leucotis | 131.78 | NA | 283910.8 | 61.27 | 15.577 | NA | NA | 1 | 1 | NA | 0.014461 |
| Soricomorpha | Talpidae | Urotrichus | talpoides | Urotrichus talpoides | 18.2 | 3.49 | 284142.6 | 145.01 | 10.561 | 2 | 2 | 2 | 1 | 2 | 0.023099 |
| Chiroptera | Vespertilionidae | Nyctophilus | microtis | Nyctophilus microtis | 7.29 | NA | 284170 | 300.87 | 22.185 | NA | NA | 1 | 2 | NA | 0.016102 |
| Rodentia | Muridae | Arvicanthis | abyssinicus | Arvicanthis abyssinicus | 73.34 | NA | 284170.4 | 91.02 | 17.794 | NA | NA | NA | NA | NA | 0.018553 |
| Soricomorpha | Soricidae | Sorex | volnuchini | Sorex volnuchini | NA | NA | 284315 | 55.9 | 5.94 | NA | NA | NA | NA | NA | 0.018346 |
| Chiroptera | Vespertilionidae | Glauconycteris | egeria | Glauconycteris egeria | NA | NA | 284431 | 129.53 | 24.195 | NA | NA | 1 | 2 | NA | 0.250694 |
| Lagomorpha | Leporidae | Lepus | brachyurus | Lepus brachyurus | 2519.45 | 2.13 | 285030 | 145.01 | 10.561 | NA | NA | NA | NA | NA | 0.015474 |
| Rodentia | Muridae | Aethomys | stannarius | Aethomys stannarius | NA | NA | 285522.8 | 88.53 | 25.87 | NA | NA | NA | NA | NA | 0.341551 |
| Rodentia | Muridae | Niviventer | niviventer | Niviventer niviventer | 100 | NA | 285657.5 | 103.37 | 6.958 | NA | NA | NA | NA | NA | 0.021458 |
| Chiroptera | Rhinolophidae | Rhinolophus | beddomei | Rhinolophus beddomei | NA | NA | 285941 | 121.02 | 25.744 | NA | NA | NA | NA | NA | 0.009408 |
| Rodentia | Muridae | Desmomys | harringtoni | Desmomys harringtoni | 89.99 | NA | 286640.4 | 93.59 | 18.481 | NA | NA | NA | NA | NA | 0.023685 |
| Artiodactyla | Bovidae | Ammodorcas | clarkei | Ammodorcas clarkei | 28049.81 | 1 | 287056.7 | 17.16 | 25.753 | NA | NA | 1 | 1 | NA | 0.860661 |
| Rodentia | Erethizontidae | Sphiggurus | melanurus | Sphiggurus melanurus | 1900 | NA | 287506.9 | 167.14 | 25.483 | NA | NA | NA | NA | NA | 0.017385 |
| Primates | Cercopithecidae | Mandrillus | sphinx | Mandrillus sphinx | 16685.06 | 1.02 | 287896.7 | 161.18 | 24.383 | 3 | 4 | 2 | 2 | 2 | 0.659091 |
| Chiroptera | Molossidae | Cheiromeles | parvidens | Cheiromeles parvidens | NA | NA | 288626.2 | 203.66 | 22.593 | NA | 1 | NA | NA | 3 | 0.016676 |
| Primates | Pitheciidae | Callicebus | donacophilus | Callicebus donacophilus | 897.67 | 1.02 | 288759 | 108.93 | 24.432 | 3 | NA | 1 | 2 | NA | 0.014113 |
| Rodentia | Muridae | Rattus | praetor | Rattus praetor | 194.43 | 4.37 | 288915.9 | 215.06 | 24.831 | NA | NA | 1 | 1 | NA | 0.016589 |
| Rodentia | Sciuridae | Paraxerus | alexandri | Paraxerus alexandri | 50.73 | 0.97 | 290513.8 | 127.93 | 22.898 | NA | NA | NA | NA | NA | 0.017722 |
| Afrosoricida | Chrysochloridae | Calcochloris | leucorhinus | Calcochloris leucorhinus | NA | NA | 290660.2 | 129.9 | 23.641 | NA | 1 | NA | NA | 3 | 0.384834 |
| Rodentia | Cricetidae | Akodon | dayi | Akodon dayi | 32.49 | NA | 290923.1 | 95.97 | 20.881 | 1 | NA | NA | NA | NA | 0.009155 |
| Soricomorpha | Soricidae | Crocidura | ludia | Crocidura ludia | 5.15 | NA | 291396.1 | 143.79 | 23.993 | NA | NA | NA | NA | NA | 0.01789 |
| Chiroptera | Vespertilionidae | Glauconycteris | humeralis | Glauconycteris humeralis | 5.49 | NA | 292110.5 | 118.09 | 22.394 | NA | NA | NA | NA | NA | 0.233188 |
| Rodentia | Sciuridae | Eupetaurus | cinereus | Eupetaurus cinereus | NA | NA | 292850.3 | 78.86 | 0.402 | NA | 2 | 2 | 2 | 1 | 0.258642 |
| Chiroptera | Hipposideridae | Hipposideros | turpis | Hipposideros turpis | 33.31 | 1 | 293300.8 | 157.37 | 24.642 | NA | 1 | 1 | 2 | 3 | 0.244431 |
| Soricomorpha | Soricidae | Crocidura | denti | Crocidura denti | NA | NA | 293305.5 | 146.74 | 24.22 | NA | NA | NA | NA | NA | 0.01789 |
| Rodentia | Caviidae | Cavia | fulgida | Cavia fulgida | 282.5 | 1.34 | 293803.2 | 125.59 | 21.043 | NA | NA | 1 | 1 | NA | 0.020458 |
| Chiroptera | Vespertilionidae | Glauconycteris | superba | Glauconycteris superba | NA | NA | 294978.9 | 120.11 | 25.552 | NA | NA | 1 | 2 | NA | 0.015668 |
| Chiroptera | Vespertilionidae | Bauerus | dubiaquercus | Bauerus dubiaquercus | 22.22 | 1.71 | 295020.7 | 171.52 | 24.132 | NA | 1 | 1 | 2 | 3 | 0.207099 |
| Rodentia | Muridae | Lenothrix | canus | Lenothrix canus | 150 | 2.91 | 295661.8 | 262.26 | 23.604 | NA | NA | NA | NA | NA | 0.023685 |
| Primates | Cebidae | Saguinus | niger | Saguinus niger | NA | NA | 295763.9 | 173.23 | 24.851 | NA | NA | NA | NA | NA | 0.647197 |
| Rodentia | Sciuridae | Tamias | umbrinus | Tamias umbrinus | 51.75 | 6.8 | 295809.4 | 23.67 | 4.751 | NA | NA | NA | NA | NA | 0.011429 |
| Rodentia | Muridae | Mesembriomys | gouldii | Mesembriomys gouldii | 716.49 | 1.87 | 296274.9 | 99.02 | 25.583 | NA | NA | NA | NA | NA | 0.168196 |
| Rodentia | Sciuridae | Tamias | quadrivittatus | Tamias quadrivittatus | 57.49 | 3.92 | 297454.2 | 26.84 | 6.65 | 3 | 5 | 2 | 2 | 2 | 0.011517 |
| Rodentia | Muridae | Lophuromys | nudicaudus | Lophuromys nudicaudus | 32.59 | 1.55 | 297751.6 | 171.3 | 24.124 | NA | NA | NA | NA | NA | 0.014783 |
| Didelphimorphia | Didelphidae | Monodelphis | kunsi | Monodelphis kunsi | 11.3 | NA | 298062.7 | 109.66 | 22.739 | 1 | 1 | 1 | 1 | 3 | 0.024176 |
| Chiroptera | Phyllostomidae | Artibeus | hirsutus | Artibeus hirsutus | 40.42 | 0.98 | 299331 | 68.43 | 20.379 | NA | 1 | 1 | 2 | 1 | 0.0115 |
| Diprotodontia | Macropodidae | Petrogale | lateralis | Petrogale lateralis | 4567.88 | 1 | 299435.8 | 22.25 | 22.561 | NA | NA | 1 | 1 | NA | 0.145016 |
| Soricomorpha | Soricidae | Sorex | excelsus | Sorex excelsus | NA | NA | 299507.2 | 110.1 | 12.504 | NA | NA | NA | NA | NA | 0.020907 |
| Didelphimorphia | Didelphidae | Micoureus | constantiae | Micoureus constantiae | 92.69 | 8 | 299842.9 | 89.78 | 23.48 | 1 | NA | 1 | 2 | NA | 0.021375 |
| Soricomorpha | Soricidae | Crocidura | douceti | Crocidura douceti | 4.3 | NA | 300718.3 | 128.17 | 25.978 | NA | NA | NA | NA | NA | 0.286234 |
| Chiroptera | Hipposideridae | Aselliscus | tricuspidatus | Aselliscus tricuspidatus | 4.08 | 0.99 | 300956.2 | 292.15 | 23.41 | NA | 1 | 1 | 2 | 3 | 0.025919 |
| Rodentia | Muridae | Praomys | derooi | Praomys derooi | 32 | 3.75 | 301232.7 | 103.4 | 26.825 | NA | NA | NA | NA | NA | 0.020329 |
| Chiroptera | Molossidae | Mormopterus | kalinowskii | Mormopterus kalinowskii | NA | NA | 303093.3 | 37.24 | 7.477 | NA | NA | 1 | 2 | NA | 0.017366 |
| Rodentia | Muridae | Sekeetamys | calurus | Sekeetamys calurus | 56.61 | 2.9 | 303182.7 | 3.26 | 21.401 | NA | NA | NA | NA | NA | 0.021015 |
| Scandentia | Tupaiidae | Dendrogale | murina | Dendrogale murina | 49.99 | NA | 303576.9 | 145.06 | 25.744 | NA | NA | NA | NA | NA | 0.020679 |
| Chiroptera | Rhinolophidae | Rhinolophus | celebensis | Rhinolophus celebensis | NA | NA | 304227.1 | 215.32 | 22.805 | NA | 1 | 1 | 2 | 3 | 0.009896 |
| Rodentia | Sciuridae | Petinomys | setosus | Petinomys setosus | 43.85 | 1.5 | 304861.3 | 221.5 | 24.406 | 1 | 4 | NA | NA | 2 | 0.648495 |
| Rodentia | Muridae | Paramelomys | platyops | Paramelomys platyops | 85.6 | 1.94 | 305085.1 | 272.52 | 22.831 | NA | 3 | 1 | 1 | 1 | 0.020235 |
| Rodentia | Cricetidae | Lemmus | lemmus | Lemmus lemmus | 67.62 | 5.1 | 305721 | 35.35 | -1.539 | NA | NA | NA | NA | NA | 0.017183 |
| Rodentia | Cricetidae | Oryzomys | albigularis | Oryzomys albigularis | 60.5 | 3.4 | 307051.8 | 152.57 | 20.658 | NA | NA | 1 | 1 | NA | 0.012924 |
| Soricomorpha | Soricidae | Chodsigoa | smithii | Chodsigoa smithii | NA | NA | 307218 | 96.39 | 9.579 | NA | NA | NA | NA | NA | 0.204584 |
| Rodentia | Caviidae | Hydrochoerus | isthmius | Hydrochoerus isthmius | 21267.32 | 4.99 | 307442.6 | 165.58 | 24.907 | NA | NA | NA | NA | NA | 0.365023 |
| Rodentia | Cricetidae | Microtus | californicus | Microtus californicus | 57.42 | 4.41 | 307995.5 | 43.86 | 11.1 | NA | NA | NA | NA | NA | 0.014923 |
| Soricomorpha | Soricidae | Sorex | dispar | Sorex dispar | 4.99 | 4.18 | 308261.8 | 86.45 | 9.046 | NA | 1 | 2 | 1 | 3 | 0.018367 |
| Rodentia | Dipodidae | Allactaga | balikunica | Allactaga balikunica | NA | NA | 308292.2 | 12.73 | 3.514 | NA | NA | NA | NA | NA | 0.023124 |
| Diprotodontia | Vombatidae | Vombatus | ursinus | Vombatus ursinus | 26000 | 1 | 308388.6 | 64.51 | 12.965 | 1 | 3 | 2 | 1 | 1 | 0.023641 |
| Rodentia | Heteromyidae | Dipodomys | deserti | Dipodomys deserti | 107.63 | 3.36 | 308413.9 | 16.06 | 15.608 | 1 | 5 | 2 | 1 | 1 | 0.015571 |
| Rodentia | Cricetidae | Auliscomys | pictus | Auliscomys pictus | 48.97 | NA | 309322 | 96.91 | 8.066 | NA | NA | NA | NA | NA | 0.016108 |
| Rodentia | Sciuridae | Cynomys | gunnisoni | Cynomys gunnisoni | 797.93 | 4.48 | 309978.8 | 25.94 | 9.434 | 3 | 5 | 2 | 1 | 2 | 0.011966 |
| Rodentia | Sciuridae | Protoxerus | aubinnii | Protoxerus aubinnii | 427.94 | NA | 310389.7 | 173.32 | 25.491 | NA | NA | NA | NA | NA | 0.282205 |
| Soricomorpha | Soricidae | Soriculus | nigrescens | Soriculus nigrescens | 14.58 | 4.89 | 310621.9 | 143.19 | 6.254 | NA | 1 | 2 | 1 | 3 | 0.019798 |
| Primates | Cercopithecidae | Rhinopithecus | roxellana | Rhinopithecus roxellana | 13456.8 | 1 | 311105.7 | 73.4 | 9.01 | 3 | 2 | 2 | 2 | 1 | 0.963787 |
| Rodentia | Muridae | Mus | setzeri | Mus setzeri | 6.65 | NA | 311593.4 | 49.99 | 21.649 | NA | NA | NA | NA | NA | 0.015632 |
| Rodentia | Sciuridae | Epixerus | ebii | Epixerus ebii | 520.96 | 2 | 312109.1 | 189.6 | 25.315 | 3 | 1 | 2 | 2 | 1 | 0.019384 |
| Lagomorpha | Ochotonidae | Ochotona | forresti | Ochotona forresti | NA | NA | 312212.2 | 174.58 | 6.723 | NA | NA | NA | NA | NA | 0.018122 |
| Carnivora | Mustelidae | Melogale | personata | Melogale personata | 1845.26 | 2.5 | 312496.3 | 160 | 22.812 | 2 | 6 | 1 | 1 | 2 | 0.30684 |
| Primates | Cercopithecidae | Trachypithecus | francoisi | Trachypithecus francoisi | 8139.93 | NA | 313031.7 | 123.18 | 18.752 | 3 | 1 | NA | NA | 1 | 0.854974 |
| Rodentia | Gliridae | Eliomys | melanurus | Eliomys melanurus | 100.36 | 4.99 | 313223.5 | 23.64 | 17.888 | NA | 4 | NA | NA | 2 | 0.021355 |
| Rodentia | Sciuridae | Ammospermophilus | interpres | Ammospermophilus interpres | 112.53 | 9.36 | 313517.4 | 24.76 | 17.702 | 3 | 4 | 2 | 1 | 2 | 0.011721 |
| Chiroptera | Vespertilionidae | Myotis | goudoti | Myotis goudoti | 5.56 | NA | 313769.9 | 130.33 | 22.389 | NA | 1 | 1 | 2 | 3 | 0.012196 |
| Chiroptera | Phyllostomidae | Glossophaga | morenoi | Glossophaga morenoi | 8.54 | 1 | 314356.1 | 109.3 | 20.446 | NA | 3 | 1 | 2 | 2 | 0.015908 |
| Dasyuromorphia | Dasyuridae | Dasyurus | albopunctatus | Dasyurus albopunctatus | 603.48 | 6.19 | 314620.6 | 262.33 | 22.502 | 2 | 5 | 1 | 1 | 2 | 0.119542 |
| Rodentia | Cricetidae | Sigmodontomys | alfari | Sigmodontomys alfari | NA | NA | 315113.8 | 200.32 | 23.982 | NA | NA | NA | NA | NA | 0.016518 |
| Rodentia | Sciuridae | Spermophilus | tereticaudus | Spermophilus tereticaudus | 147.57 | 6.32 | 315363.7 | 17.01 | 18.035 | 3 | 4 | 2 | 1 | 2 | 0.012618 |
| Chiroptera | Hipposideridae | Hipposideros | grandis | Hipposideros grandis | NA | NA | 315496.5 | 112.86 | 21.631 | NA | NA | NA | NA | NA | 0.326167 |
| Rodentia | Cricetidae | Oryzomys | xanthaeolus | Oryzomys xanthaeolus | 79.79 | NA | 317397.1 | 51.73 | 12.008 | NA | NA | NA | NA | NA | 0.01522 |
| Rodentia | Dipodidae | Sicista | tianshanica | Sicista tianshanica | NA | 4.37 | 317896.4 | 21.29 | -0.201 | NA | NA | NA | NA | NA | 0.02343 |
| Rodentia | Cricetidae | Akodon | lutescens | Akodon lutescens | 20.69 | NA | 318909.7 | 88.47 | 11.352 | NA | NA | NA | NA | NA | 0.013492 |
| Chiroptera | Hipposideridae | Hipposideros | maggietaylorae | Hipposideros maggietaylorae | 16.1 | NA | 319262.3 | 296.47 | 22.629 | NA | NA | 1 | 2 | NA | 0.024482 |
| Diprotodontia | Pseudocheiridae | Petropseudes | dahli | Petropseudes dahli | 1877.03 | 1 | 319548.6 | 68.96 | 26.353 | NA | 1 | 1 | 2 | 1 | 0.022401 |
| Dasyuromorphia | Dasyuridae | Antechinus | swainsonii | Antechinus swainsonii | 58.69 | 8.24 | 319669.6 | 70.99 | 13.667 | 2 | 2 | 2 | 1 | 2 | 0.016758 |
| Chiroptera | Phyllostomidae | Glossophaga | leachii | Glossophaga leachii | 10.24 | 0.98 | 319942.7 | 119.14 | 20.86 | NA | 3 | 1 | 2 | 2 | 0.015908 |
| Soricomorpha | Soricidae | Crocidura | attila | Crocidura attila | NA | NA | 320205.4 | 157.36 | 23.995 | NA | NA | NA | NA | NA | 0.01789 |
| Carnivora | Mustelidae | Mustela | kathiah | Mustela kathiah | 263.74 | NA | 320536.5 | 120.3 | 15.481 | NA | 1 | 1 | 1 | 3 | 0.017271 |
| Rodentia | Muridae | Acomys | louisae | Acomys louisae | 20.19 | NA | 320804.6 | 14.54 | 24.835 | NA | NA | NA | NA | NA | 0.018505 |
| Primates | Aotidae | Aotus | lemurinus | Aotus lemurinus | 865.96 | 1.01 | 321845.6 | 190.02 | 24.221 | 1 | 4 | 1 | 2 | 2 | 0.342709 |
| Chiroptera | Vespertilionidae | Scotophilus | robustus | Scotophilus robustus | NA | NA | 322073.1 | 131.72 | 22.413 | 2 | 1 | 1 | 2 | 3 | 0.014147 |
| Artiodactyla | Bovidae | Procapra | gutturosa | Procapra gutturosa | 28274.27 | 1.41 | 322170.9 | 23.87 | 0.627 | 2 | 1 | 1 | 1 | 1 | 0.017447 |
| Rodentia | Cricetidae | Holochilus | chacarius | Holochilus chacarius | 204 | NA | 323383.6 | 90.07 | 22.327 | 2 | 2 | 3 | 2 | 1 | 0.010768 |
| Chiroptera | Rhinolophidae | Rhinolophus | rex | Rhinolophus rex | NA | NA | 323465.2 | 108.96 | 14.447 | NA | 1 | 1 | 2 | 3 | 0.012207 |
| Rodentia | Ctenodactylidae | Ctenodactylus | gundi | Ctenodactylus gundi | 288.41 | 2 | 324162.5 | 15.84 | 17.379 | NA | NA | NA | NA | NA | 0.019424 |
| Soricomorpha | Soricidae | Crocidura | obscurior | Crocidura obscurior | NA | NA | 324332.9 | 194.21 | 25.443 | NA | NA | NA | NA | NA | 0.01789 |
| Soricomorpha | Soricidae | Crocidura | vorax | Crocidura vorax | NA | NA | 325781.4 | 119.4 | 18.57 | NA | NA | NA | NA | NA | 0.015538 |
| Chiroptera | Rhinolophidae | Rhinolophus | arcuatus | Rhinolophus arcuatus | 8.98 | NA | 326282.7 | 247.96 | 22.612 | NA | 1 | 1 | 2 | 3 | 0.012207 |
| Rodentia | Cricetidae | Reithrodontomys | gracilis | Reithrodontomys gracilis | 12.47 | 3.11 | 326863.3 | 130.43 | 24.02 | 1 | NA | 2 | 2 | NA | 0.018435 |
| Rodentia | Spalacidae | Myospalax | myospalax | Myospalax myospalax | 225 | 4.5 | 327057.1 | 30.1 | -0.848 | NA | NA | NA | NA | NA | 0.02021 |
| Soricomorpha | Talpidae | Scapanus | latimanus | Scapanus latimanus | 62.46 | 3.49 | 327590.1 | 43.2 | 10.472 | 2 | 1 | 1 | 1 | 3 | 0.021794 |
| Rodentia | Cricetidae | Oryzomys | russatus | Oryzomys russatus | 60.5 | NA | 327955.5 | 123.76 | 19.846 | 1 | 3 | 1 | 1 | 2 | 0.01522 |
| Chiroptera | Pteropodidae | Eonycteris | major | Eonycteris major | 70.7 | 1 | 328195.7 | 271.55 | 23.209 | NA | 1 | 1 | 2 | 1 | 0.283768 |
| Primates | Cercopithecidae | Cercopithecus | hamlyni | Cercopithecus hamlyni | NA | NA | 328372.6 | 126.88 | 22.621 | NA | NA | NA | NA | NA | 0.877509 |
| Lagomorpha | Leporidae | Sylvilagus | cunicularius | Sylvilagus cunicularius | 2489.54 | 3.71 | 329698 | 91.17 | 19.458 | 2 | 2 | 1 | 1 | 1 | 0.014837 |
| Artiodactyla | Tayassuidae | Catagonus | wagneri | Catagonus wagneri | 35566.4 | 2.47 | 329880.9 | 62.93 | 22.121 | 3 | 6 | 1 | 1 | 2 | 1.669843 |
| Chiroptera | Phyllostomidae | Carollia | subrufa | Carollia subrufa | 15.84 | 0.98 | 330694.8 | 143.06 | 23.226 | NA | 1 | 1 | 2 | 1 | 0.016511 |
| Rodentia | Muridae | Gerbilliscus | guineae | Gerbilliscus guineae | 102.5 | NA | 331990.5 | 128.08 | 26.524 | NA | NA | NA | NA | NA | 0.01967 |
| Rodentia | Cricetidae | Andinomys | edax | Andinomys edax | 69.75 | 2.91 | 332581.8 | 60.47 | 7.982 | NA | NA | 1 | 1 | NA | 0.019815 |
| Rodentia | Sciuridae | Xerus | princeps | Xerus princeps | 624.9 | 2 | 333016.5 | 22.45 | 17.247 | 3 | 3 | 3 | 2 | 1 | 0.020049 |
| Rodentia | Cricetidae | Oryzomys | bolivaris | Oryzomys bolivaris | 60.5 | 3.23 | 333553.4 | 221.88 | 24.098 | 1 | 3 | 1 | 1 | 2 | 0.01522 |
| Rodentia | Cricetidae | Scapteromys | tumidus | Scapteromys tumidus | 146 | 3.63 | 333590.2 | 103.67 | 17.116 | 2 | 1 | 2 | 1 | 3 | 0.015226 |
| Rodentia | Cricetidae | Calomys | hummelincki | Calomys hummelincki | 27 | NA | 333859.8 | 105.43 | 25.83 | 1 | NA | 1 | 1 | NA | 0.809845 |
| Rodentia | Spalacidae | Myospalax | aspalax | Myospalax aspalax | NA | NA | 333938.3 | 28.29 | -1.134 | NA | NA | NA | NA | NA | 0.02021 |
| Cingulata | Dasypodidae | Chaetophractus | nationi | Chaetophractus nationi | 2110.04 | NA | 334227.3 | 47.35 | 4.391 | NA | 1 | 2 | 1 | 3 | 0.983855 |
| Chiroptera | Hipposideridae | Hipposideros | camerunensis | Hipposideros camerunensis | NA | NA | 336169.3 | 148.83 | 23.262 | NA | NA | 1 | 2 | NA | 0.300787 |
| Chiroptera | Vespertilionidae | Nyctalus | montanus | Nyctalus montanus | NA | NA | 336171.5 | 76.03 | 16.447 | NA | NA | NA | NA | NA | 0.015824 |
| Rodentia | Muridae | Rattus | lutreolus | Rattus lutreolus | 106 | 4.45 | 336333.3 | 77 | 14.466 | 2 | 4 | 2 | 1 | 2 | 0.016593 |
| Rodentia | Dipodidae | Allactaga | tetradactyla | Allactaga tetradactyla | 52 | NA | 336436.4 | 5.97 | 20.095 | NA | NA | NA | NA | NA | 1.156213 |
| Chiroptera | Phyllostomidae | Artibeus | fraterculus | Artibeus fraterculus | NA | 0.98 | 336498.7 | 67.93 | 16.507 | NA | 1 | 1 | 2 | 1 | 0.011885 |
| Rodentia | Sciuridae | Spermophilus | beldingi | Spermophilus beldingi | 272.53 | 5.71 | 337927.7 | 30.29 | 6.97 | 3 | 5 | 2 | 1 | 2 | 0.010373 |
| Lagomorpha | Ochotonidae | Ochotona | thomasi | Ochotona thomasi | NA | NA | 340281 | 57.54 | -3.003 | NA | NA | 2 | 1 | NA | 0.01889 |
| Chiroptera | Pteropodidae | Dobsonia | minor | Dobsonia minor | 85.95 | 0.99 | 340450.1 | 283.43 | 23.172 | NA | 1 | 1 | 2 | 1 | 0.011263 |
| Rodentia | Anomaluridae | Anomalurus | pelii | Anomalurus pelii | 1769.99 | 2.5 | 340631 | 167.82 | 25.625 | NA | NA | NA | NA | NA | 0.382951 |
| Didelphimorphia | Didelphidae | Thylamys | pallidior | Thylamys pallidior | 15.34 | NA | 341665.7 | 65.29 | 14.071 | 1 | NA | 2 | 2 | NA | 0.019765 |
| Rodentia | Geomyidae | Geomys | breviceps | Geomys breviceps | 136.47 | 2.53 | 341932.2 | 89.36 | 18.135 | 1 | 3 | 1 | 1 | 1 | 0.015239 |
| Chiroptera | Pteropodidae | Scotonycteris | ophiodon | Scotonycteris ophiodon | 69.42 | 0.98 | 342183.8 | 155.36 | 25.021 | NA | NA | 1 | 2 | NA | 0.693337 |
| Chiroptera | Hipposideridae | Hipposideros | dyacorum | Hipposideros dyacorum | NA | NA | 343384.4 | 275.18 | 23.275 | NA | 1 | 1 | 2 | 3 | 0.024482 |
| Chiroptera | Vespertilionidae | Scoteanax | rueppellii | Scoteanax rueppellii | 26.41 | 1 | 343840.7 | 85.29 | 17.585 | NA | NA | 1 | 2 | NA | 0.022856 |
| Erinaceomorpha | Erinaceidae | Paraechinus | micropus | Paraechinus micropus | 171 | 4 | 344515.3 | 54.37 | 25.743 | 1 | 2 | NA | NA | 3 | 0.01959 |
| Rodentia | Ctenomyidae | Ctenomys | opimus | Ctenomys opimus | 327.05 | 2 | 344662.6 | 39.3 | 1.606 | 3 | 3 | 2 | 1 | 1 | 0.016918 |
| Rodentia | Cricetidae | Microtus | majori | Microtus majori | NA | 3.6 | 345482.8 | 59.98 | 6.231 | NA | NA | NA | NA | NA | 0.014235 |
| Diprotodontia | Petauridae | Petaurus | australis | Petaurus australis | 567.15 | 1.24 | 346584.4 | 76.3 | 16.573 | 1 | 3 | 1 | 2 | 2 | 0.017031 |
| Chiroptera | Pteropodidae | Epomophorus | angolensis | Epomophorus angolensis | NA | 1 | 346769.5 | 73.63 | 20.7 | NA | NA | 1 | 2 | NA | 0.107354 |
| Chiroptera | Vespertilionidae | Miniopterus | minor | Miniopterus minor | NA | NA | 347309.2 | 64.51 | 24.226 | NA | NA | NA | NA | NA | 0.263898 |
| Soricomorpha | Soricidae | Sorex | asper | Sorex asper | NA | NA | 347978.8 | 27.12 | 1.26 | NA | NA | NA | NA | NA | 0.014498 |
| Rodentia | Cricetidae | Sigmodon | arizonae | Sigmodon arizonae | 198 | NA | 348392.3 | 32.38 | 17.781 | NA | NA | NA | NA | NA | 0.014461 |
| Rodentia | Cricetidae | Wilfredomys | oenax | Wilfredomys oenax | 46.8 | NA | 348515.4 | 120.33 | 16.809 | 1 | NA | 1 | 2 | NA | 1.238865 |
| Rodentia | Cricetidae | Loxodontomys | micropus | Loxodontomys micropus | 70.76 | 4.16 | 348628.7 | 69.99 | 6.322 | 1 | 6 | 3 | 2 | 2 | 0.016108 |
| Soricomorpha | Talpidae | Scapanus | orarius | Scapanus orarius | 62.13 | 3.24 | 351196.8 | 69.8 | 7.402 | 2 | 3 | 2 | 1 | 2 | 0.020748 |
| Chiroptera | Pteropodidae | Nyctimene | robinsoni | Nyctimene robinsoni | 48.83 | 0.99 | 351920.4 | 95.91 | 21.802 | NA | 1 | 1 | 2 | 1 | 0.016212 |
| Afrosoricida | Chrysochloridae | Chlorotalpa | sclateri | Chlorotalpa sclateri | 38.3 | 2 | 352293.8 | 38.02 | 15.624 | NA | 1 | NA | NA | 3 | 0.024052 |
| Primates | Cercopithecidae | Colobus | satanas | Colobus satanas | 9055.32 | 0.99 | 353215.3 | 165.46 | 24.126 | 3 | 3 | 1 | 2 | 1 | 0.772265 |
| Rodentia | Cricetidae | Akodon | subfuscus | Akodon subfuscus | 30.39 | NA | 353424 | 100.64 | 10.233 | NA | NA | NA | NA | NA | 0.013492 |
| Artiodactyla | Bovidae | Naemorhedus | goral | Naemorhedus goral | 28796.51 | 1 | 353466.2 | 106.99 | 5.389 | 2 | 2 | 1 | 1 | 1 | 0.128574 |
| Rodentia | Muridae | Notomys | cervinus | Notomys cervinus | 34.79 | 2.95 | 353845.5 | 15.84 | 20.08 | NA | NA | NA | NA | NA | 0.950872 |
| Rodentia | Muridae | Rattus | andamanensis | Rattus andamanensis | NA | 10 | 354404.7 | 167.27 | 17.071 | 1 | 4 | 2 | 1 | 2 | 0.013963 |
| Peramelemorphia | Peramelidae | Isoodon | obesulus | Isoodon obesulus | 824.76 | 2.33 | 354752.8 | 64.32 | 14.218 | 2 | 2 | 2 | 1 | 2 | 0.016838 |
| Rodentia | Muridae | Myotomys | unisulcatus | Myotomys unisulcatus | 102.5 | 1.42 | 356725.2 | 24.74 | 15.841 | NA | 1 | NA | NA | 1 | 0.019294 |
| Dasyuromorphia | Dasyuridae | Pseudantechinus | woolleyae | Pseudantechinus woolleyae | 43.23 | 5.99 | 356927.9 | 22 | 22.583 | 2 | NA | 1 | 1 | NA | 0.014319 |
| Rodentia | Muridae | Acomys | kempi | Acomys kempi | 21.99 | NA | 358017.7 | 42.35 | 27.157 | NA | NA | NA | NA | NA | 0.013851 |
| Chiroptera | Vespertilionidae | Miniopterus | macrocneme | Miniopterus macrocneme | 7.53 | 1 | 358072.3 | 260.02 | 22.117 | NA | NA | NA | NA | NA | 0.263898 |
| Chiroptera | Vespertilionidae | Kerivoula | smithii | Kerivoula smithii | NA | 0.98 | 358126.2 | 166.94 | 23.929 | NA | NA | 1 | 2 | NA | 0.015685 |
| Rodentia | Cricetidae | Eolagurus | luteus | Eolagurus luteus | 26 | 7.5 | 358386.9 | 13.55 | 1.204 | NA | NA | NA | NA | NA | 0.016729 |
| Soricomorpha | Soricidae | Crocidura | cinderella | Crocidura cinderella | NA | NA | 358909.4 | 44.08 | 28.159 | NA | NA | NA | NA | NA | 0.01789 |
| Rodentia | Cricetidae | Thalpomys | cerradensis | Thalpomys cerradensis | 23.99 | NA | 359016.5 | 126.71 | 23.244 | NA | NA | NA | NA | NA | 0.016518 |
| Soricomorpha | Talpidae | Scaptonyx | fusicaudus | Scaptonyx fusicaudus | NA | NA | 359135.9 | 102.21 | 8.169 | NA | NA | NA | NA | NA | 0.026075 |
| Rodentia | Cricetidae | Neodon | sikimensis | Neodon sikimensis | NA | 2.25 | 359265.8 | 155.96 | 3.976 | NA | NA | NA | NA | NA | 0.013667 |
| Chiroptera | Pteropodidae | Pteropus | rufus | Pteropus rufus | NA | NA | 359698.8 | 126.27 | 22.445 | NA | NA | NA | NA | NA | 0.748106 |
| Lagomorpha | Leporidae | Sylvilagus | palustris | Sylvilagus palustris | 1355.23 | 2.96 | 360038.8 | 104.53 | 19.31 | 1 | 2 | 1 | 1 | 1 | 0.015453 |
| Rodentia | Muridae | Apodemus | argenteus | Apodemus argenteus | NA | NA | 361275.2 | 132.24 | 9.059 | NA | NA | NA | NA | NA | 0.018118 |
| Rodentia | Muridae | Apodemus | speciosus | Apodemus speciosus | 43.75 | 4.47 | 361275.2 | 132.24 | 9.059 | NA | NA | NA | NA | NA | 0.018118 |
| Chiroptera | Pteropodidae | Paranyctimene | raptor | Paranyctimene raptor | 24.94 | 0.98 | 361367.1 | 283.61 | 22.693 | NA | 1 | 1 | 2 | 1 | 0.020562 |
| Chiroptera | Pteropodidae | Pteropus | macrotis | Pteropus macrotis | 365.99 | 1 | 361950.1 | 280.61 | 22.813 | NA | NA | 1 | 2 | NA | 0.014679 |
| Soricomorpha | Soricidae | Crocidura | goliath | Crocidura goliath | 87.92 | NA | 362264.1 | 143.28 | 24.273 | NA | NA | NA | NA | NA | 0.01789 |
| Rodentia | Muridae | Hybomys | trivirgatus | Hybomys trivirgatus | 60.08 | 2.72 | 362389.1 | 163.61 | 25.665 | NA | NA | NA | NA | NA | 0.018217 |
| Carnivora | Mustelidae | Mustela | itatsi | Mustela itatsi | NA | NA | 362403.8 | 132.24 | 9.059 | NA | NA | NA | NA | NA | 0.012723 |
| Rodentia | Muridae | Rattus | leucopus | Rattus leucopus | 200.89 | 3.45 | 362571.3 | 223.09 | 23.938 | 1 | 4 | 2 | 1 | 2 | 0.016589 |
| Primates | Cercopithecidae | Cercocebus | torquatus | Cercocebus torquatus | 7293.07 | 1.01 | 362869.6 | 175.62 | 25.243 | 3 | 1 | NA | NA | 1 | 0.707321 |
| Diprotodontia | Macropodidae | Macropus | parryi | Macropus parryi | 12629.83 | 1 | 364427.1 | 78.38 | 19.636 | 1 | 1 | 1 | 1 | 1 | 0.017167 |
| Chiroptera | Vespertilionidae | Neoromicia | melckorum | Neoromicia melckorum | NA | 1.98 | 364896.3 | 42.54 | 18.514 | NA | 1 | 1 | 2 | 3 | 0.28701 |
| Rodentia | Sciuridae | Spermophilus | mollis | Spermophilus mollis | 165.4 | 7.97 | 364932.5 | 24.56 | 7.097 | NA | NA | NA | NA | NA | 0.010373 |
| Rodentia | Sciuridae | Dremomys | lokriah | Dremomys lokriah | 226.99 | 3.68 | 365201.5 | 179.15 | 12.885 | NA | NA | 1 | 2 | NA | 0.018891 |
| Rodentia | Sciuridae | Myosciurus | pumilio | Myosciurus pumilio | 16.44 | 1.97 | 366327 | 180.04 | 24.3 | 3 | 4 | 1 | 2 | 2 | 0.021629 |
| Rodentia | Muridae | Rattus | losea | Rattus losea | 104.63 | NA | 367079.3 | 151.3 | 23.013 | NA | NA | NA | NA | NA | 0.013963 |
| Chiroptera | Rhinolophidae | Rhinolophus | subbadius | Rhinolophus subbadius | NA | NA | 368631.8 | 186.35 | 17.22 | NA | 1 | 1 | 2 | 3 | 0.016361 |
| Artiodactyla | Bovidae | Damaliscus | pygargus | Damaliscus pygargus | 77784.55 | 1.41 | 370656.2 | 49.54 | 15.683 | NA | NA | NA | NA | NA | 0.018601 |
| Rodentia | Cricetidae | Oligoryzomys | delticola | Oligoryzomys delticola | 29.39 | 3.3 | 372583.1 | 111.31 | 17.493 | 1 | 2 | 1 | 2 | 2 | 0.013332 |
| Rodentia | Sciuridae | Sciurus | griseus | Sciurus griseus | 703.85 | 2.65 | 373639.1 | 51.63 | 10.143 | 3 | 4 | 2 | 2 | 1 | 0.010026 |
| Dasyuromorphia | Dasyuridae | Dasykaluta | rosamondae | Dasykaluta rosamondae | 32.17 | 6.64 | 374318.6 | 22.02 | 23.761 | 2 | NA | 1 | 1 | NA | 0.015985 |
| Didelphimorphia | Didelphidae | Gracilinanus | microtarsus | Gracilinanus microtarsus | 29.3 | NA | 375848.1 | 132.51 | 18.246 | 1 | NA | 2 | 2 | NA | 0.329485 |
| Rodentia | Sciuridae | Tamiops | rodolphii | Tamiops rodolphii | NA | NA | 376423.6 | 145.23 | 25.864 | NA | NA | NA | NA | NA | 0.017406 |
| Chiroptera | Phyllostomidae | Leptonycteris | curasoae | Leptonycteris curasoae | 25.27 | 0.99 | 376465.7 | 94.09 | 23.397 | NA | 3 | 1 | 2 | 2 | 0.820554 |
| Chiroptera | Vespertilionidae | Nyctophilus | walkeri | Nyctophilus walkeri | 4.44 | NA | 376698.6 | 80.68 | 26.842 | NA | NA | 1 | 2 | NA | 0.016102 |
| Carnivora | Mustelidae | Martes | melampus | Martes melampus | 1000 | NA | 377250.8 | 132.97 | 10.302 | NA | NA | 1 | 1 | NA | 0.016592 |
| Rodentia | Cricetidae | Peromyscus | polionotus | Peromyscus polionotus | 14.27 | 3.59 | 377395.2 | 105.85 | 18.547 | 1 | NA | NA | NA | NA | 0.017825 |
| Soricomorpha | Soricidae | Sorex | trowbridgii | Sorex trowbridgii | 5.02 | 3.91 | 377970.2 | 69.9 | 8.385 | 2 | 3 | 1 | 1 | 2 | 0.019481 |
| Diprotodontia | Burramyidae | Cercartetus | nanus | Cercartetus nanus | 26.85 | 3.96 | 378748.8 | 70.94 | 13.185 | 1 | 4 | 2 | 2 | 2 | 0.021551 |
| Rodentia | Cricetidae | Oryzomys | rostratus | Oryzomys rostratus | NA | NA | 379484.1 | 111.6 | 24.437 | NA | NA | NA | NA | NA | 0.01522 |
| Chiroptera | Vespertilionidae | Phoniscus | papuensis | Phoniscus papuensis | 6.32 | NA | 379533 | 114.37 | 20.4 | NA | NA | 1 | 2 | NA | 0.016422 |
| Erinaceomorpha | Erinaceidae | Mesechinus | hughi | Mesechinus hughi | NA | NA | 379917.4 | 59.05 | 11.612 | 1 | 2 | NA | NA | 3 | 0.02003 |
| Rodentia | Cricetidae | Oryzomys | talamancae | Oryzomys talamancae | 54.99 | NA | 380181.6 | 173.3 | 24.458 | 1 | 3 | 1 | 1 | 2 | 0.01522 |
| Rodentia | Echimyidae | Lonchothrix | emiliae | Lonchothrix emiliae | 138.2 | NA | 380559.2 | 161.06 | 25.444 | 1 | NA | 1 | 2 | NA | 0.020662 |
| Chiroptera | Phyllostomidae | Sturnira | bidens | Sturnira bidens | 18.05 | 0.99 | 381210.5 | 145.74 | 19.1 | NA | 1 | 1 | 2 | 1 | 0.019109 |
| Rodentia | Abrocomidae | Abrocoma | cinerea | Abrocoma cinerea | 193.64 | 2.19 | 381391 | 48.46 | 3.522 | NA | NA | 3 | 2 | NA | 0.021324 |
| Dasyuromorphia | Dasyuridae | Sminthopsis | virginiae | Sminthopsis virginiae | 35.24 | 4.11 | 381450.2 | 115.54 | 25.859 | 2 | 2 | 1 | 1 | 3 | 0.015591 |
| Soricomorpha | Soricidae | Sorex | mirabilis | Sorex mirabilis | 15 | NA | 382085.7 | 54.83 | 0.528 | NA | 2 | NA | NA | 3 | 0.334514 |
| Rodentia | Muridae | Hylomyscus | denniae | Hylomyscus denniae | 35.99 | 4.61 | 382114.2 | 88.52 | 20.746 | NA | NA | NA | NA | NA | 0.015329 |
| Chiroptera | Molossidae | Molossus | aztecus | Molossus aztecus | 14.86 | NA | 382245.9 | 110.37 | 20.813 | NA | NA | NA | NA | NA | 0.019807 |
| Rodentia | Sciuridae | Heliosciurus | punctatus | Heliosciurus punctatus | 173.85 | NA | 382580.5 | 164.71 | 25.545 | NA | NA | NA | NA | NA | 0.277435 |
| Lagomorpha | Ochotonidae | Ochotona | roylei | Ochotona roylei | 260 | 3 | 382682.3 | 48.02 | -3.612 | NA | NA | 1 | 1 | NA | 0.016375 |
| Peramelemorphia | Peramelidae | Perameles | nasuta | Perameles nasuta | 720.26 | 2.43 | 383336.9 | 80.76 | 16.64 | 2 | 2 | 2 | 1 | 2 | 0.018771 |
| Rodentia | Cricetidae | Caryomys | inez | Caryomys inez | NA | 1.94 | 383954 | 44.02 | 9.909 | NA | NA | NA | NA | NA | 0.013 |
| Chiroptera | Molossidae | Mops | demonstrator | Mops demonstrator | NA | 1 | 384114.4 | 85.38 | 25.716 | NA | NA | 1 | 2 | NA | 0.014867 |
| Chiroptera | Pteropodidae | Pteropus | conspicillatus | Pteropus conspicillatus | 760.71 | 1 | 384685.7 | 162.43 | 24.216 | NA | 3 | 1 | 2 | 1 | 0.01529 |
| Chiroptera | Molossidae | Mops | sarasinorum | Mops sarasinorum | NA | NA | 384859.3 | 217.37 | 22.866 | NA | 1 | 1 | 2 | 3 | 0.277788 |
| Soricomorpha | Soricidae | Episoriculus | leucops | Episoriculus leucops | NA | NA | 385403.7 | 145.68 | 12.279 | NA | NA | NA | NA | NA | 0.020458 |
| Chiroptera | Vespertilionidae | Murina | suilla | Murina suilla | 4 | NA | 385996.4 | 251.05 | 23.682 | NA | NA | 1 | 2 | NA | 0.015749 |
| Rodentia | Muridae | Berylmys | manipulus | Berylmys manipulus | NA | NA | 386054.3 | 186.26 | 14.779 | NA | NA | NA | NA | NA | 0.30752 |
| Rodentia | Muridae | Malacomys | edwardsi | Malacomys edwardsi | 63.78 | 2.46 | 387957.6 | 172.79 | 25.9 | NA | NA | NA | NA | NA | 0.019355 |
| Rodentia | Sciuridae | Ratufa | indica | Ratufa indica | 1060 | NA | 388205.9 | 121.06 | 25.612 | NA | 4 | 2 | 2 | 2 | 0.16842 |
| Rodentia | Muridae | Heimyscus | fumosus | Heimyscus fumosus | 17.2 | NA | 388339.4 | 151.22 | 24.124 | 1 | NA | 1 | 1 | NA | 0.023685 |
| Chiroptera | Pteropodidae | Nyctimene | aello | Nyctimene aello | 85.26 | 0.98 | 390567.6 | 266.74 | 22.72 | NA | NA | 1 | 2 | NA | 0.016279 |
| Chiroptera | Rhinolophidae | Rhinolophus | philippinensis | Rhinolophus philippinensis | 10.91 | NA | 391836.8 | 208.4 | 23.647 | NA | 1 | 1 | 2 | 3 | 0.009896 |
| Chiroptera | Vespertilionidae | Pipistrellus | papuanus | Pipistrellus papuanus | NA | NA | 391922.3 | 282.22 | 23.1 | NA | NA | NA | NA | NA | 0.015988 |
| Rodentia | Cuniculidae | Cuniculus | taczanowskii | Cuniculus taczanowskii | 8999.95 | 1 | 392199.7 | 157.32 | 19.181 | 1 | 4 | 2 | 1 | 1 | 0.226707 |
| Rodentia | Heteromyidae | Dipodomys | microps | Dipodomys microps | 56.26 | 2.37 | 392813.7 | 20.55 | 8.812 | 1 | 3 | 2 | 1 | 2 | 0.013895 |
| Rodentia | Muridae | Stenocephalemys | albipes | Stenocephalemys albipes | NA | 4.41 | 393242.3 | 88.33 | 18.063 | NA | NA | NA | NA | NA | 0.020329 |
| Chiroptera | Vespertilionidae | Kerivoula | whiteheadi | Kerivoula whiteheadi | 3.29 | NA | 393863.4 | 228.44 | 23.916 | NA | NA | 1 | 2 | NA | 0.015685 |
| Lagomorpha | Ochotonidae | Ochotona | erythrotis | Ochotona erythrotis | NA | NA | 394635.9 | 43.71 | -4.859 | NA | NA | 2 | 1 | NA | 0.018852 |
| Rodentia | Cricetidae | Akodon | iniscatus | Akodon iniscatus | 28.7 | NA | 395416.7 | 21.27 | 9.068 | NA | NA | NA | NA | NA | 0.013492 |
| Rodentia | Spalacidae | Tachyoryctes | splendens | Tachyoryctes splendens | 227.13 | 1.41 | 396020.9 | 78.44 | 17.758 | NA | NA | NA | NA | NA | 0.021074 |
| Rodentia | Sciuridae | Sciurus | variegatoides | Sciurus variegatoides | 485 | 4.99 | 396517.5 | 186.7 | 23.257 | 3 | 4 | 2 | 2 | 2 | 0.010982 |
| Rodentia | Octodontidae | Octodontomys | gliroides | Octodontomys gliroides | 155.77 | 2.04 | 396551.4 | 43.03 | 3.99 | 1 | 2 | 2 | 2 | 1 | 0.026397 |
| Rodentia | Heteromyidae | Chaetodipus | formosus | Chaetodipus formosus | 20.02 | 5.28 | 397044.7 | 17.81 | 11.714 | NA | NA | NA | NA | NA | 0.021066 |
| Chiroptera | Vespertilionidae | Myotis | keenii | Myotis keenii | 6.51 | 0.99 | 398644.4 | 78.44 | 1.962 | NA | NA | 1 | 2 | NA | 0.012431 |
| Rodentia | Dipodidae | Allactodipus | bobrinskii | Allactodipus bobrinskii | NA | 5.15 | 399244 | 10.42 | 12.382 | NA | NA | NA | NA | NA | 0.025649 |
| Rodentia | Geomyidae | Orthogeomys | hispidus | Orthogeomys hispidus | 499.99 | 1.97 | 399397.8 | 116.21 | 23.491 | NA | 3 | 2 | 1 | 1 | 0.015066 |
| Rodentia | Cricetidae | Neodon | juldaschi | Neodon juldaschi | NA | 3.52 | 399664.3 | 21.71 | 0.562 | NA | NA | NA | NA | NA | 0.015568 |
| Lagomorpha | Leporidae | Lepus | granatensis | Lepus granatensis | 2323.94 | 1.46 | 401180 | 45.33 | 13.124 | NA | NA | NA | NA | NA | 0.01453 |
| Soricomorpha | Soricidae | Crocidura | dsinezumi | Crocidura dsinezumi | NA | 4.29 | 401721.5 | 134.61 | 9.759 | NA | NA | NA | NA | NA | 0.01789 |
| Artiodactyla | Bovidae | Budorcas | taxicolor | Budorcas taxicolor | 294515.3 | 1.22 | 402044.4 | 129.45 | 3.988 | NA | 3 | 1 | 1 | 1 | 0.887503 |
| Chiroptera | Vespertilionidae | Myotis | longipes | Myotis longipes | NA | NA | 402070.4 | 94.68 | 10.093 | NA | NA | 1 | 2 | NA | 0.232745 |
| Rodentia | Cricetidae | Akodon | toba | Akodon toba | 51.2 | NA | 402084.4 | 77.69 | 23.584 | NA | NA | NA | NA | NA | 0.009155 |
| Rodentia | Cricetidae | Dicrostonyx | richardsoni | Dicrostonyx richardsoni | NA | NA | 403806.7 | 26.6 | -9.519 | NA | NA | NA | NA | NA | 0.014002 |
| Cingulata | Dasypodidae | Chlamyphorus | truncatus | Chlamyphorus truncatus | 83.53 | NA | 404571.1 | 33.98 | 13.629 | 2 | 6 | 2 | 1 | 2 | 0.417669 |
| Rodentia | Echimyidae | Hoplomys | gymnurus | Hoplomys gymnurus | 281.42 | 2.1 | 404762.5 | 225.35 | 23.929 | 1 | 2 | 2 | 1 | 1 | 0.020662 |
| Rodentia | Heteromyidae | Chaetodipus | penicillatus | Chaetodipus penicillatus | 15.57 | 3.4 | 407770.3 | 20.78 | 17.196 | NA | NA | NA | NA | NA | 0.015109 |
| Rodentia | Cricetidae | Reithrodontomys | sumichrasti | Reithrodontomys sumichrasti | 19 | 3.49 | 408113.6 | 117.05 | 20.875 | 1 | NA | 2 | 2 | NA | 0.018428 |
| Rodentia | Erethizontidae | Coendou | nycthemera | Coendou nycthemera | 2999.98 | NA | 409217.1 | 168.73 | 25.557 | NA | NA | NA | NA | NA | 0.228905 |
| Carnivora | Ursidae | Tremarctos | ornatus | Tremarctos ornatus | 123177 | 1.44 | 409517.7 | 125.36 | 17.982 | 2 | 5 | 1 | 1 | 2 | 1.193065 |
| Rodentia | Muridae | Gerbillus | gleadowi | Gerbillus gleadowi | 26.01 | 3 | 409918.8 | 30.76 | 25.297 | NA | NA | NA | NA | NA | 0.222319 |
| Primates | Cercopithecidae | Semnopithecus | dussumieri | Semnopithecus dussumieri | NA | NA | 410033.7 | 106.04 | 25.856 | NA | NA | NA | NA | NA | 0.013347 |
| Rodentia | Chinchillidae | Lagidium | peruanum | Lagidium peruanum | 1220 | 1 | 410167.5 | 95.65 | 9.447 | 3 | 2 | NA | NA | 1 | 0.019855 |
| Chiroptera | Nycteridae | Nycteris | parisii | Nycteris parisii | NA | NA | 410454.8 | 53.51 | 25.152 | NA | NA | NA | NA | NA | 0.278783 |
| Didelphimorphia | Didelphidae | Monodelphis | rubida | Monodelphis rubida | 45.5 | NA | 410687.4 | 128.16 | 21.263 | 1 | 1 | 1 | 1 | 3 | 0.34163 |
| Rodentia | Cricetidae | Blanfordimys | afghanus | Blanfordimys afghanus | NA | 3.4 | 410889 | 22.93 | 9.078 | NA | NA | NA | NA | NA | 0.017983 |
| Soricomorpha | Soricidae | Sorex | alpinus | Sorex alpinus | 8.04 | 5.95 | 411029.8 | 77.43 | 5.928 | NA | NA | NA | NA | NA | 0.223923 |
| Soricomorpha | Soricidae | Crocidura | littoralis | Crocidura littoralis | 18.61 | 2.99 | 411448.8 | 112.85 | 21.926 | NA | NA | NA | NA | NA | 0.01789 |
| Rodentia | Muridae | Hylomyscus | parvus | Hylomyscus parvus | 14.49 | NA | 412234.9 | 150.42 | 24.191 | 1 | NA | 2 | 2 | NA | 0.015329 |
| Chiroptera | Vespertilionidae | Eptesicus | pachyotis | Eptesicus pachyotis | 8.1 | NA | 412871.1 | 141.35 | 20.023 | NA | NA | 1 | 2 | NA | 0.016767 |
| Primates | Hominidae | Pan | paniscus | Pan paniscus | 35119.95 | 1.01 | 412936.1 | 161.12 | 25.033 | 3 | 6 | 2 | 2 | 2 | 1.263397 |
| Rodentia | Muridae | Dephomys | defua | Dephomys defua | 43.05 | 2.75 | 414109.8 | 179.68 | 25.564 | NA | NA | NA | NA | NA | 0.019438 |
| Rodentia | Cricetidae | Thomasomys | aureus | Thomasomys aureus | 87.99 | NA | 414682.3 | 122.64 | 14.616 | NA | 1 | 1 | 2 | 1 | 0.012123 |
| Rodentia | Muridae | Grammomys | cometes | Grammomys cometes | 45 | 2.9 | 415006.2 | 70.25 | 22.538 | NA | NA | NA | NA | NA | 0.020268 |
| Chiroptera | Vespertilionidae | Hypsugo | eisentrauti | Hypsugo eisentrauti | 6.11 | NA | 415632.1 | 82.67 | 22.554 | NA | NA | 1 | 2 | NA | 0.312186 |
| Chiroptera | Phyllostomidae | Artibeus | watsoni | Artibeus watsoni | 11.2 | 1 | 415964.1 | 185.4 | 24.227 | NA | NA | NA | NA | NA | 0.013498 |
| Lagomorpha | Leporidae | Sylvilagus | bachmani | Sylvilagus bachmani | 714.53 | 3.35 | 417293.5 | 45.6 | 12.92 | 1 | 3 | 2 | 1 | 1 | 0.018414 |
| Carnivora | Ursidae | Melursus | ursinus | Melursus ursinus | 99999.99 | 1.54 | 417387.7 | 119.46 | 24.179 | 2 | 6 | 1 | 1 | 2 | 0.99519 |
| Rodentia | Cricetidae | Sigmodon | ochrognathus | Sigmodon ochrognathus | 122 | 3 | 418072.2 | 28.35 | 16.021 | NA | NA | NA | NA | NA | 0.014461 |
| Carnivora | Viverridae | Genetta | abyssinica | Genetta abyssinica | 1405.48 | NA | 418638.2 | 61.18 | 23.259 | NA | NA | 1 | 1 | NA | 0.014667 |
| Chiroptera | Vespertilionidae | Myotis | adversus | Myotis adversus | 10.41 | 1 | 418891.8 | 219.39 | 23.998 | NA | 1 | 1 | 2 | 3 | 0.014221 |
| Rodentia | Sciuridae | Callosciurus | caniceps | Callosciurus caniceps | 264.91 | 2.19 | 420861.7 | 183.7 | 24.901 | NA | NA | 1 | 2 | NA | 0.019853 |
| Lagomorpha | Leporidae | Lepus | othus | Lepus othus | 4837.35 | 6.15 | 421759.6 | 33.89 | -4.281 | NA | 2 | NA | NA | 1 | 0.012503 |
| Rodentia | Cricetidae | Eligmodontia | puerulus | Eligmodontia puerulus | 28.49 | NA | 423135 | 45.53 | 1.7 | NA | NA | NA | NA | NA | 0.014905 |
| Rodentia | Cricetidae | Abrothrix | longipilis | Abrothrix longipilis | 38.86 | 3.7 | 423823.7 | 64.15 | 6.448 | 2 | 7 | 3 | 2 | 2 | 0.012978 |
| Rodentia | Cricetidae | Peromyscus | mexicanus | Peromyscus mexicanus | 42.58 | 2.56 | 424193.4 | 134.83 | 22.164 | 1 | 2 | 1 | 1 | 1 | 0.017825 |
| Soricomorpha | Talpidae | Scapanulus | oweni | Scapanulus oweni | NA | NA | 424699 | 59.33 | 8.647 | NA | 1 | NA | NA | 3 | 0.024431 |
| Rodentia | Cricetidae | Microtus | ilaeus | Microtus ilaeus | NA | NA | 424998 | 17.5 | 3.503 | NA | NA | NA | NA | NA | 0.013117 |
| Chiroptera | Vespertilionidae | Hesperoptenus | tickelli | Hesperoptenus tickelli | 16.3 | 1 | 425021.1 | 129.82 | 23.252 | NA | NA | 1 | 2 | NA | 0.018927 |
| Primates | Hylobatidae | Hylobates | lar | Hylobates lar | 5578.61 | 1.01 | 425651.8 | 172.74 | 22.963 | 3 | 4 | NA | NA | 2 | 1.067786 |
| Rodentia | Cricetidae | Akodon | albiventer | Akodon albiventer | 25.95 | NA | 425856.2 | 43.02 | 3.173 | 3 | 1 | 1 | 1 | 3 | 0.013492 |
| Artiodactyla | Bovidae | Neotragus | pygmaeus | Neotragus pygmaeus | 3913.68 | 1 | 426515.1 | 176.5 | 25.617 | 2 | 4 | NA | NA | 1 | 0.021771 |
| Chiroptera | Pteropodidae | Chironax | melanocephalus | Chironax melanocephalus | 17.7 | 0.98 | 427413.2 | 232.64 | 24.524 | NA | 1 | 1 | 2 | 1 | 0.017299 |
| Rodentia | Petromuridae | Petromus | typicus | Petromus typicus | 224 | 1.5 | 427688.4 | 15.15 | 16.807 | 3 | 3 | 1 | 1 | 1 | 0.02661 |
| Soricomorpha | Soricidae | Crocidura | flavescens | Crocidura flavescens | 31.22 | 3.37 | 428284.7 | 51.51 | 15.304 | 2 | 1 | NA | NA | 3 | 0.01789 |
| Chiroptera | Hipposideridae | Hipposideros | lylei | Hipposideros lylei | 40 | NA | 428641.2 | 161.24 | 23.794 | NA | 1 | 1 | 2 | 3 | 0.021054 |
| Lagomorpha | Leporidae | Lepus | callotis | Lepus callotis | 2608.16 | 2.48 | 430968.1 | 66.16 | 16.799 | 1 | 1 | NA | NA | 1 | 0.118607 |
| Primates | Cebidae | Cebus | capucinus | Cebus capucinus | 3005.99 | 1.01 | 431037.9 | 216.57 | 24.05 | 3 | 4 | 2 | 2 | 2 | 0.014063 |
| Rodentia | Muridae | Pogonomys | macrourus | Pogonomys macrourus | 46.98 | 2.81 | 431652.3 | 256.55 | 22.466 | 1 | 3 | 3 | 2 | 1 | 0.019355 |
| Rodentia | Sciuridae | Callosciurus | albescens | Callosciurus albescens | NA | NA | 432350.7 | 229.28 | 25.245 | NA | NA | NA | NA | NA | 0.31925 |
| Chiroptera | Emballonuridae | Emballonura | raffrayana | Emballonura raffrayana | 5.61 | NA | 432542.2 | 257.66 | 22.255 | 1 | NA | 1 | 2 | NA | 0.023995 |
| Carnivora | Herpestidae | Dologale | dybowskii | Dologale dybowskii | 361.92 | 4 | 433013.2 | 124.43 | 23.433 | NA | NA | 1 | 1 | NA | 0.368131 |
| Soricomorpha | Soricidae | Crocidura | pergrisea | Crocidura pergrisea | NA | NA | 433223 | 25.93 | 0.67 | NA | NA | NA | NA | NA | 0.286234 |
| Rodentia | Muridae | Melomys | burtoni | Melomys burtoni | 71.29 | 2.5 | 433347.8 | 95.87 | 23.749 | NA | NA | NA | NA | NA | 0.016139 |
| Chiroptera | Phyllostomidae | Platyrrhinus | aurarius | Platyrrhinus aurarius | 35.12 | NA | 434351.7 | 212.23 | 24.164 | NA | NA | 1 | 2 | NA | 0.016057 |
| Rodentia | Muridae | Myomyscus | angolensis | Myomyscus angolensis | NA | NA | 434874.8 | 99.42 | 21.548 | NA | NA | NA | NA | NA | 0.020022 |
| Rodentia | Sciuridae | Belomys | pearsonii | Belomys pearsonii | NA | NA | 434941.7 | 145.99 | 17.714 | NA | NA | NA | NA | NA | 0.213134 |
| Chiroptera | Vespertilionidae | Falsistrellus | tasmaniensis | Falsistrellus tasmaniensis | 22.54 | NA | 436078 | 69.83 | 13.654 | NA | 1 | 1 | 2 | 3 | 0.017436 |
| Rodentia | Echimyidae | Makalata | macrura | Makalata macrura | 439 | NA | 437079.6 | 202.16 | 22.528 | NA | NA | NA | NA | NA | 0.01783 |
| Chiroptera | Furipteridae | Amorphochilus | schnablii | Amorphochilus schnablii | NA | 0.99 | 437429.7 | 34.8 | 10.252 | NA | 1 | 1 | 2 | 3 | 1.806395 |
| Rodentia | Cricetidae | Peromyscus | difficilis | Peromyscus difficilis | 27.95 | 3.88 | 437500.4 | 52.87 | 15.913 | 1 | NA | NA | NA | NA | 0.017825 |
| Soricomorpha | Soricidae | Crocidura | macarthuri | Crocidura macarthuri | NA | NA | 437724 | 37.71 | 27.321 | NA | NA | NA | NA | NA | 0.01789 |
| Carnivora | Herpestidae | Crossarchus | obscurus | Crossarchus obscurus | 1395.09 | 4.29 | 438210.8 | 175.02 | 25.638 | 3 | 6 | 1 | 1 | 2 | 0.014793 |
| Chiroptera | Vespertilionidae | Glauconycteris | beatrix | Glauconycteris beatrix | 7.5 | NA | 438945.1 | 139.41 | 25.85 | NA | NA | 1 | 2 | NA | 0.014574 |
| Rodentia | Cricetidae | Alticola | tuvinicus | Alticola tuvinicus | NA | NA | 439592.8 | 25.62 | -7.847 | NA | NA | NA | NA | NA | 0.012109 |
| Rodentia | Muridae | Dipodillus | stigmonyx | Dipodillus stigmonyx | NA | NA | 439932.2 | 25.75 | 25.397 | NA | NA | NA | NA | NA | 0.35571 |
| Chiroptera | Rhinolophidae | Rhinolophus | euryotis | Rhinolophus euryotis | 14.3 | NA | 440225.2 | 260.02 | 22.308 | NA | 1 | 1 | 2 | 3 | 0.012207 |
| Primates | Cercopithecidae | Cercocebus | atys | Cercocebus atys | 6941.24 | 1.01 | 440620.6 | 186.09 | 25.564 | 3 | NA | NA | NA | NA | 0.707321 |
| Didelphimorphia | Didelphidae | Marmosa | lepida | Marmosa lepida | 14 | 4.99 | 441236.5 | 197.72 | 25.608 | 1 | NA | NA | NA | NA | 0.024984 |
| Carnivora | Procyonidae | Bassaricyon | beddardi | Bassaricyon beddardi | 1235.01 | NA | 441326.3 | 186.26 | 23.989 | 1 | NA | 1 | 1 | NA | 0.020106 |
| Rodentia | Heteromyidae | Liomys | pictus | Liomys pictus | 43.28 | 3.67 | 441467 | 82.44 | 20.764 | 1 | 1 | 1 | 1 | 1 | 0.013165 |
| Rodentia | Cricetidae | Oecomys | rutilus | Oecomys rutilus | 73.4 | NA | 442179.9 | 160.63 | 25.244 | NA | NA | NA | NA | NA | 0.012792 |
| Rodentia | Sciuridae | Spermophilus | xanthoprymnus | Spermophilus xanthoprymnus | NA | 4.99 | 442235.8 | 42.85 | 9.096 | NA | NA | NA | NA | NA | 0.106495 |
| Chiroptera | Vespertilionidae | Histiotus | alienus | Histiotus alienus | NA | NA | 442813.1 | 120.65 | 17.133 | NA | 1 | 1 | 2 | 3 | 0.311185 |
| Chiroptera | Pteropodidae | Sphaerias | blanfordi | Sphaerias blanfordi | 28.88 | NA | 444062.2 | 134.32 | 16.571 | NA | NA | 1 | 2 | NA | 0.021568 |
| Soricomorpha | Soricidae | Crocidura | grandiceps | Crocidura grandiceps | 23 | NA | 444066.2 | 143.68 | 25.856 | NA | NA | NA | NA | NA | 0.178896 |
| Chiroptera | Phyllostomidae | Lonchophylla | hesperia | Lonchophylla hesperia | NA | NA | 444717.1 | 106.41 | 18.043 | NA | 3 | 1 | 2 | 2 | 0.881559 |
| Soricomorpha | Soricidae | Chodsigoa | hypsibia | Chodsigoa hypsibia | NA | NA | 446123.8 | 107.32 | 6.887 | NA | NA | NA | NA | NA | 0.020458 |
| Rodentia | Muridae | Taterillus | arenarius | Taterillus arenarius | 47 | NA | 446588 | 19.13 | 28.653 | NA | NA | NA | NA | NA | 0.019791 |
| Rodentia | Muridae | Meriones | vinogradovi | Meriones vinogradovi | 117 | 7.48 | 447673.4 | 40.34 | 10.505 | NA | NA | NA | NA | NA | 0.018016 |
| Chiroptera | Vespertilionidae | Nyctalus | plancyi | Nyctalus plancyi | NA | NA | 448501.7 | 105.67 | 14.943 | NA | NA | NA | NA | NA | 0.015729 |
| Chiroptera | Vespertilionidae | Rhogeessa | parvula | Rhogeessa parvula | 4.37 | 1.98 | 449611.7 | 77.1 | 20.172 | NA | 1 | 1 | 2 | 3 | 0.014252 |
| Primates | Cebidae | Cebus | nigritus | Cebus nigritus | NA | 1 | 449987.6 | 126.58 | 18.975 | NA | 4 | 2 | 2 | 2 | 1.224305 |
| Chiroptera | Phyllostomidae | Anoura | latidens | Anoura latidens | 15.06 | NA | 450083 | 102.16 | 25.458 | NA | 3 | 1 | 2 | 2 | 0.01736 |
| Rodentia | Cricetidae | Cricetulus | sokolovi | Cricetulus sokolovi | NA | NA | 450811.9 | 23.56 | 1.425 | NA | NA | NA | NA | NA | 0.021048 |
| Chiroptera | Phyllostomidae | Sturnira | aratathomasi | Sturnira aratathomasi | 49.67 | 0.99 | 450907.4 | 188.57 | 22.758 | NA | 1 | 1 | 2 | 1 | 0.161768 |
| Primates | Cercopithecidae | Cercopithecus | lhoesti | Cercopithecus lhoesti | 5307.47 | 1 | 451166.2 | 127.13 | 22.556 | 3 | 3 | NA | NA | 2 | 0.529582 |
| Rodentia | Muridae | Rattus | fuscipes | Rattus fuscipes | 124.58 | 4.58 | 451237.9 | 75.52 | 15.748 | 1 | 3 | 2 | 1 | 2 | 0.016593 |
| Carnivora | Herpestidae | Galerella | ochracea | Galerella ochracea | NA | NA | 452479.8 | 23.4 | 25.965 | NA | NA | NA | NA | NA | 0.018079 |
| Rodentia | Cricetidae | Baiomys | musculus | Baiomys musculus | 8.99 | 2.62 | 452712.4 | 109.05 | 20.707 | 2 | 4 | 2 | 1 | 1 | 0.019236 |
| Chiroptera | Hipposideridae | Hipposideros | lamottei | Hipposideros lamottei | NA | NA | 452875.3 | 141.3 | 25.936 | NA | NA | 1 | 2 | NA | 1.998303 |
| Carnivora | Mustelidae | Mustela | strigidorsa | Mustela strigidorsa | 1500 | NA | 453670.1 | 163.3 | 15.957 | NA | NA | 1 | 1 | NA | 0.013937 |
| Soricomorpha | Soricidae | Crocidura | zarudnyi | Crocidura zarudnyi | NA | 5.99 | 453690.2 | 14.55 | 15.7 | NA | NA | NA | NA | NA | 0.01789 |
| Primates | Cercopithecidae | Trachypithecus | pileatus | Trachypithecus pileatus | 11219.93 | NA | 453900.8 | 190.31 | 20.332 | 3 | NA | NA | NA | NA | 0.433419 |
| Rodentia | Cricetidae | Peromyscus | attwateri | Peromyscus attwateri | 27.9 | 3.53 | 454032.5 | 66.18 | 16.77 | 1 | 5 | NA | NA | 2 | 0.017825 |
| Rodentia | Dipodidae | Sicista | concolor | Sicista concolor | NA | NA | 454459 | 57.15 | 1.498 | NA | NA | NA | NA | NA | 0.02343 |
| Chiroptera | Pteropodidae | Pteropus | neohibernicus | Pteropus neohibernicus | 1017.37 | 1 | 455283.1 | 264.97 | 23.986 | NA | NA | 1 | 2 | NA | 0.018381 |
| Rodentia | Echimyidae | Carterodon | sulcidens | Carterodon sulcidens | 113.8 | NA | 456431.7 | 114.85 | 21.078 | NA | NA | 2 | 1 | NA | 0.330599 |
| Rodentia | Muridae | Oenomys | ornatus | Oenomys ornatus | 85.5 | NA | 456581.4 | 178.24 | 25.508 | NA | NA | NA | NA | NA | 0.017863 |
| Chiroptera | Vespertilionidae | Hesperoptenus | blanfordi | Hesperoptenus blanfordi | 6.91 | NA | 457426.6 | 221.88 | 24.983 | NA | NA | 1 | 2 | NA | 0.018927 |
| Soricomorpha | Soricidae | Paracrocidura | schoutedeni | Paracrocidura schoutedeni | 12.18 | NA | 458097.1 | 149.83 | 24.23 | NA | NA | NA | NA | NA | 0.015731 |
| Rodentia | Cricetidae | Oxymycterus | angularis | Oxymycterus angularis | 67.99 | NA | 459009.2 | 105.47 | 25.056 | NA | 1 | NA | NA | 3 | 0.014623 |
| Rodentia | Sciuridae | Funisciurus | isabella | Funisciurus isabella | 109.31 | 0.98 | 459256.7 | 167.03 | 24.178 | 3 | 1 | 2 | 2 | 1 | 0.295697 |
| Rodentia | Sciuridae | Spermophilus | beecheyi | Spermophilus beecheyi | 597.82 | 6.71 | 459262.6 | 49.14 | 10.349 | NA | NA | 1 | 1 | NA | 0.010345 |
| Rodentia | Ctenomyidae | Ctenomys | torquatus | Ctenomys torquatus | 209.49 | 2.5 | 459331.3 | 113.3 | 17.949 | 3 | 3 | 2 | 1 | 1 | 0.016918 |
| Chiroptera | Rhinolophidae | Rhinolophus | pusillus | Rhinolophus pusillus | 5.15 | 1 | 459599.2 | 101.35 | 19.887 | NA | 1 | 1 | 2 | 3 | 0.021161 |
| Rodentia | Muridae | Chiromyscus | chiropus | Chiromyscus chiropus | NA | NA | 460796.2 | 137.31 | 21.188 | NA | NA | NA | NA | NA | 0.023685 |
| Primates | Cebidae | Callimico | goeldii | Callimico goeldii | 558 | 1.05 | 462922.1 | 209.19 | 25.978 | 3 | 2 | 1 | 2 | 2 | 0.933847 |
| Rodentia | Cricetidae | Oxymycterus | inca | Oxymycterus inca | 34.99 | NA | 463452.9 | 135.31 | 20.826 | NA | 1 | NA | NA | 3 | 0.014623 |
| Primates | Galagidae | Galago | gabonensis | Galago gabonensis | NA | NA | 465080.6 | 157.01 | 24.278 | NA | NA | NA | NA | NA | 0.020355 |
| Primates | Pitheciidae | Cacajao | melanocephalus | Cacajao melanocephalus | 3121.58 | NA | 465904.6 | 231.02 | 25.053 | 3 | 3 | 1 | 2 | 2 | 0.014314 |
| Carnivora | Procyonidae | Bassaricyon | alleni | Bassaricyon alleni | NA | NA | 467192.7 | 208.81 | 25.81 | NA | NA | NA | NA | NA | 0.020106 |
| Rodentia | Muridae | Rattus | sordidus | Rattus sordidus | 157.5 | 5.99 | 467956.7 | 132.05 | 23.32 | NA | NA | NA | NA | NA | 0.016593 |
| Chiroptera | Vespertilionidae | Myotis | atacamensis | Myotis atacamensis | NA | NA | 469816.7 | 12.15 | 2.518 | NA | NA | NA | NA | NA | 0.158622 |
| Chiroptera | Vespertilionidae | Myotis | fortidens | Myotis fortidens | 4.37 | 0.98 | 470952 | 90.99 | 21.823 | NA | 1 | 1 | 2 | 3 | 0.01728 |
| Rodentia | Geomyidae | Thomomys | umbrinus | Thomomys umbrinus | 125.56 | 2.2 | 471470.9 | 46.02 | 16.504 | NA | 3 | 2 | 1 | 1 | 0.019594 |
| Diprotodontia | Macropodidae | Petrogale | brachyotis | Petrogale brachyotis | 4012.01 | 1 | 476435.1 | 76.96 | 27.049 | NA | NA | 1 | 1 | NA | 0.012075 |
| Didelphimorphia | Didelphidae | Tlacuatzin | canescens | Tlacuatzin canescens | 47.73 | 8.49 | 476499.6 | 87.55 | 20.614 | 1 | NA | 2 | 2 | NA | 0.024984 |
| Rodentia | Muridae | Lophuromys | chrysopus | Lophuromys chrysopus | NA | NA | 478180.9 | 85.07 | 17.516 | NA | NA | NA | NA | NA | 0.014673 |
| Rodentia | Muridae | Otomys | anchietae | Otomys anchietae | NA | NA | 478210.3 | 116.99 | 22.129 | NA | NA | NA | NA | NA | 0.016305 |
| Rodentia | Cricetidae | Pseudoryzomys | simplex | Pseudoryzomys simplex | 45.2 | NA | 478695.7 | 108.57 | 23.617 | NA | NA | 1 | 1 | NA | 0.013758 |
| Chiroptera | Pteropodidae | Epomops | buettikoferi | Epomops buettikoferi | 135.67 | 0.98 | 479614.2 | 164.99 | 25.677 | NA | 1 | 1 | 2 | 1 | 0.011217 |
| Rodentia | Heteromyidae | Heteromys | desmarestianus | Heteromys desmarestianus | 74 | 3.06 | 479714.7 | 167.46 | 23.165 | 1 | 2 | 1 | 1 | 1 | 0.014035 |
| Rodentia | Cricetidae | Tylomys | nudicaudus | Tylomys nudicaudus | 181.65 | 2.47 | 480905.8 | 139.27 | 22.172 | 3 | NA | 1 | NA | NA | 0.01959 |
| Dasyuromorphia | Dasyuridae | Sminthopsis | dolichura | Sminthopsis dolichura | 14.39 | 7.2 | 481145 | 24.48 | 17.713 | 2 | NA | 1 | 1 | NA | 0.016971 |
| Primates | Lorisidae | Loris | lydekkerianus | Loris lydekkerianus | 254.06 | NA | 481306.5 | 112.22 | 26.396 | NA | NA | NA | NA | NA | 0.022469 |
| Rodentia | Cricetidae | Volemys | millicens | Volemys millicens | NA | NA | 483932.6 | 141.56 | 4.184 | NA | NA | NA | NA | NA | 0.286065 |
| Chiroptera | Vespertilionidae | Arielulus | circumdatus | Arielulus circumdatus | 10.4 | NA | 484035.1 | 166.35 | 15.927 | NA | NA | 1 | 2 | NA | 0.018882 |
| Rodentia | Sciuridae | Spermophilus | citellus | Spermophilus citellus | 395.97 | 6.48 | 486400.5 | 51.36 | 8.695 | 3 | 3 | 2 | 1 | 2 | 0.51788 |
| Chiroptera | Rhinolophidae | Rhinolophus | acuminatus | Rhinolophus acuminatus | 12.1 | 1 | 486496.8 | 195.82 | 25.174 | NA | 1 | 1 | 2 | 3 | 0.012207 |
| Rodentia | Sciuridae | Tamiops | swinhoei | Tamiops swinhoei | NA | 3.4 | 486562.1 | 121.92 | 9.181 | NA | NA | NA | NA | NA | 0.018119 |
| Rodentia | Muridae | Niviventer | andersoni | Niviventer andersoni | NA | NA | 486719.1 | 129.11 | 7.051 | NA | NA | NA | NA | NA | 0.021458 |
| Scandentia | Tupaiidae | Tupaia | dorsalis | Tupaia dorsalis | 168.05 | NA | 486839.8 | 271.94 | 23.91 | 3 | 4 | NA | NA | 2 | 0.37559 |
| Rodentia | Cricetidae | Dicrostonyx | hudsonius | Dicrostonyx hudsonius | 57 | 3.49 | 487889.7 | 43.94 | -6.642 | NA | NA | NA | NA | NA | 0.014002 |
| Rodentia | Dipodidae | Allactaga | hotsoni | Allactaga hotsoni | NA | NA | 489818.5 | 8.58 | 19.639 | NA | NA | NA | NA | NA | 0.023124 |
| Rodentia | Cricetidae | Neacomys | guianae | Neacomys guianae | 15.87 | NA | 490417.7 | 175.32 | 25.312 | NA | 3 | 1 | 1 | 2 | 0.015865 |
| Rodentia | Sciuridae | Petinomys | genibarbis | Petinomys genibarbis | 108.81 | 1.07 | 490763.8 | 238.5 | 24.686 | 1 | 4 | NA | NA | 2 | 0.759408 |
| Rodentia | Nesomyidae | Steatomys | cuppedius | Steatomys cuppedius | NA | NA | 490786.7 | 139.33 | 26.206 | NA | NA | NA | NA | NA | 0.021606 |
| Rodentia | Sciuridae | Tamiasciurus | douglasii | Tamiasciurus douglasii | 225 | 5.49 | 491601.9 | 70.9 | 7.359 | 3 | 5 | 2 | 2 | 2 | 0.013882 |
| Rodentia | Chinchillidae | Chinchilla | chinchilla | Chinchilla chinchilla | 499.99 | 2.5 | 492097.5 | 56.04 | 2.005 | 2 | 6 | 1 | 1 | 1 | 1.847554 |
| Chiroptera | Hipposideridae | Hipposideros | stenotis | Hipposideros stenotis | 12 | NA | 492128.4 | 73.89 | 26.684 | NA | NA | 1 | 2 | NA | 0.018372 |
| Chiroptera | Pteropodidae | Pteropus | hypomelanus | Pteropus hypomelanus | 435.61 | 1 | 492950.1 | 214.4 | 23.608 | NA | 1 | 1 | 2 | 1 | 0.018381 |
| Rodentia | Cricetidae | Neodon | irene | Neodon irene | NA | NA | 494103.4 | 123.25 | 0.012 | NA | NA | NA | NA | NA | 0.012515 |
| Rodentia | Muridae | Apodemus | latronum | Apodemus latronum | NA | NA | 494407 | 145.12 | 5.848 | NA | NA | NA | NA | NA | 0.018118 |
| Soricomorpha | Soricidae | Crocidura | maquassiensis | Crocidura maquassiensis | 5.99 | NA | 494580.6 | 51.25 | 17.774 | NA | 1 | NA | NA | 3 | 0.01789 |
| Rodentia | Cricetidae | Peromyscus | melanotis | Peromyscus melanotis | 39.59 | 3.69 | 495038.6 | 53 | 16.213 | 1 | NA | NA | NA | NA | 0.017825 |
| Chiroptera | Vespertilionidae | Vespadelus | darlingtoni | Vespadelus darlingtoni | 6.06 | NA | 495284 | 65.24 | 13.585 | NA | 1 | 1 | 2 | 3 | 0.017918 |
| Diprotodontia | Phalangeridae | Trichosurus | arnhemensis | Trichosurus arnhemensis | 1540.74 | 1.01 | 495415.7 | 73.91 | 26.859 | 1 | 2 | 1 | 2 | 1 | 0.017605 |
| Chiroptera | Molossidae | Myopterus | daubentonii | Myopterus daubentonii | NA | NA | 495506.9 | 168.61 | 25.546 | NA | NA | 1 | 2 | NA | 0.266813 |
| Rodentia | Cricetidae | Auliscomys | sublimis | Auliscomys sublimis | 38 | NA | 497531.4 | 62.02 | 3.857 | NA | NA | 1 | 1 | NA | 0.016108 |
| Rodentia | Sciuridae | Exilisciurus | exilis | Exilisciurus exilis | 21.75 | 2.46 | 497876.8 | 258.26 | 24.468 | NA | NA | NA | NA | NA | 0.300714 |
| Rodentia | Nesomyidae | Saccostomus | mearnsi | Saccostomus mearnsi | 64.91 | 5.75 | 497929.5 | 55.95 | 23.894 | NA | 3 | NA | NA | 2 | 0.022216 |
| Rodentia | Cricetidae | Alticola | barakshin | Alticola barakshin | NA | NA | 498938.7 | 19.89 | -7.153 | NA | NA | NA | NA | NA | 0.014271 |
| Rodentia | Sciuridae | Marmota | caudata | Marmota caudata | 4350 | 3.65 | 499610.4 | 19.7 | -1.282 | NA | NA | NA | NA | NA | 0.009845 |
| Chiroptera | Phyllostomidae | Chiroderma | doriae | Chiroderma doriae | 19.9 | 1 | 499911.4 | 125.32 | 20.037 | NA | 1 | 1 | 2 | 1 | 0.012751 |
| Primates | Cebidae | Cebus | libidinosus | Cebus libidinosus | NA | NA | 500245.3 | 115.7 | 24.423 | NA | NA | NA | NA | NA | 0.016324 |
| Chiroptera | Nycteridae | Nycteris | aurita | Nycteris aurita | NA | NA | 500257.5 | 72.34 | 22.899 | NA | NA | NA | NA | NA | 0.017134 |
| Soricomorpha | Talpidae | Uropsilus | gracilis | Uropsilus gracilis | NA | NA | 502188.9 | 108.82 | 8.507 | NA | NA | NA | NA | NA | 0.023961 |
| Primates | Cercopithecidae | Colobus | vellerosus | Colobus vellerosus | 7700.18 | 1.02 | 502614.9 | 106.72 | 26.835 | 3 | NA | NA | NA | NA | 0.583965 |
| Scandentia | Tupaiidae | Tupaia | gracilis | Tupaia gracilis | 111.95 | NA | 502657.3 | 272.59 | 24.077 | 3 | 4 | NA | NA | 2 | 0.023474 |
| Rodentia | Cricetidae | Ototylomys | phyllotis | Ototylomys phyllotis | 86.94 | 2.2 | 502791.5 | 146.59 | 23.426 | 1 | 3 | 3 | 2 | 1 | 0.01966 |
| Chiroptera | Vespertilionidae | Myotis | punicus | Myotis punicus | NA | NA | 503620 | 34.26 | 15.418 | NA | NA | NA | NA | NA | 0.12124 |
| Rodentia | Sciuridae | Callosciurus | pygerythrus | Callosciurus pygerythrus | NA | NA | 503627.2 | 170.22 | 21.469 | NA | NA | 1 | 2 | NA | 0.019953 |
| Rodentia | Spalacidae | Spalax | ehrenbergi | Spalax ehrenbergi | 164.35 | NA | 504281.3 | 19.91 | 18.976 | 2 | NA | 1 | 1 | NA | 0.32988 |
| Didelphimorphia | Didelphidae | Monodelphis | adusta | Monodelphis adusta | 36.04 | NA | 505245.7 | 184.96 | 21.345 | 1 | 3 | 1 | 1 | 2 | 0.024176 |
| Rodentia | Cricetidae | Abrothrix | jelskii | Abrothrix jelskii | 34.5 | NA | 506394.7 | 76.81 | 6.822 | 2 | 3 | 1 | 1 | 2 | 0.016105 |
| Lagomorpha | Leporidae | Brachylagus | idahoensis | Brachylagus idahoensis | 431.19 | 5.81 | 506906.1 | 23.12 | 6.898 | 2 | 2 | 2 | 1 | 1 | 0.022054 |
| Rodentia | Muridae | Gerbillus | tarabuli | Gerbillus tarabuli | NA | NA | 507731.8 | 2.83 | 22.471 | NA | NA | NA | NA | NA | 0.022232 |
| Soricomorpha | Soricidae | Nectogale | elegans | Nectogale elegans | 38.82 | NA | 509363.9 | 126.95 | 8.46 | NA | 2 | 3 | 1 | 3 | 0.024152 |
| Primates | Hylobatidae | Hylobates | muelleri | Hylobates muelleri | 5909.81 | 1.02 | 511224.1 | 266.53 | 24.008 | 3 | 4 | NA | NA | 2 | 1.067786 |
| Rodentia | Muridae | Meriones | rex | Meriones rex | NA | NA | 511676.3 | 10.68 | 22.152 | NA | NA | NA | NA | NA | 0.016466 |
| Rodentia | Heteromyidae | Chaetodipus | intermedius | Chaetodipus intermedius | 14.94 | 5.53 | 511920.2 | 24.36 | 16.066 | NA | NA | NA | NA | NA | 0.016501 |
| Primates | Pitheciidae | Callicebus | nigrifrons | Callicebus nigrifrons | NA | NA | 513267.9 | 128.2 | 20.37 | NA | NA | NA | NA | NA | 0.186333 |
| Rodentia | Cricetidae | Peromyscus | melanophrys | Peromyscus melanophrys | 40 | 2.91 | 515026.3 | 68.14 | 17.797 | 1 | NA | NA | NA | NA | 0.017825 |
| Diprotodontia | Macropodidae | Macropus | antilopinus | Macropus antilopinus | 27250.1 | 1 | 515289.1 | 84.75 | 27.02 | 1 | NA | 1 | 1 | NA | 0.015011 |
| Artiodactyla | Bovidae | Eudorcas | thomsonii | Eudorcas thomsonii | 22907.43 | 1 | 515821.1 | 72.57 | 22.728 | 2 | 2 | 1 | 1 | 1 | 0.117733 |
| Chiroptera | Vespertilionidae | Myotis | macrodactylus | Myotis macrodactylus | 7.48 | 1 | 517296.3 | 104.45 | 6.385 | NA | NA | 1 | 2 | NA | 0.013033 |
| Rodentia | Cricetidae | Oecomys | rex | Oecomys rex | 73.4 | NA | 518332 | 176.38 | 25.361 | NA | NA | NA | NA | NA | 0.012792 |
| Rodentia | Heteromyidae | Perognathus | longimembris | Perognathus longimembris | 8.07 | 4.45 | 518799.8 | 20.68 | 11.484 | 1 | 1 | NA | NA | 1 | 0.020442 |
| Soricomorpha | Soricidae | Crocidura | muricauda | Crocidura muricauda | NA | NA | 519293.4 | 178.55 | 25.589 | NA | NA | NA | NA | NA | 0.01789 |
| Rodentia | Muridae | Lemniscomys | griselda | Lemniscomys griselda | 55.87 | 3.68 | 520132.4 | 99.38 | 21.399 | NA | NA | NA | NA | NA | 0.019499 |
| Chiroptera | Vespertilionidae | Chalinolobus | dwyeri | Chalinolobus dwyeri | 8.74 | 1.34 | 521325.9 | 65.83 | 17.614 | NA | NA | 1 | 2 | NA | 0.18045 |
| Chiroptera | Phyllostomidae | Vampyressa | melissa | Vampyressa melissa | 16.58 | 0.98 | 521452.8 | 240.44 | 25.798 | NA | NA | 1 | 2 | NA | 0.745649 |
| Rodentia | Cricetidae | Phenacomys | intermedius | Phenacomys intermedius | 25.2 | 4.66 | 524177.2 | 51.33 | 4.108 | 2 | 4 | NA | NA | 1 | 0.014635 |
| Rodentia | Heteromyidae | Dipodomys | spectabilis | Dipodomys spectabilis | 124.61 | 2.67 | 525470.9 | 26.65 | 15.327 | 1 | 4 | 2 | 1 | 2 | 0.141525 |
| Primates | Cercopithecidae | Colobus | polykomos | Colobus polykomos | 8797.29 | 1 | 526083.9 | 180.76 | 25.722 | 3 | 2 | NA | NA | 1 | 0.583965 |
| Primates | Atelidae | Alouatta | sara | Alouatta sara | 6611.04 | NA | 526742.3 | 109.48 | 24.489 | 3 | NA | NA | NA | NA | 0.014532 |
| Chiroptera | Pteropodidae | Nyctimene | albiventer | Nyctimene albiventer | 29.95 | 0.98 | 527482.6 | 256.72 | 23.476 | NA | 1 | 1 | 2 | 1 | 0.015129 |
| Chiroptera | Phyllostomidae | Lonchophylla | handleyi | Lonchophylla handleyi | NA | NA | 527831 | 138.99 | 18.325 | NA | 3 | 1 | 2 | 2 | 0.017631 |
| Soricomorpha | Soricidae | Sorex | raddei | Sorex raddei | NA | 2.69 | 528211.1 | 53.6 | 6.552 | NA | NA | NA | NA | NA | 0.020907 |
| Rodentia | Muridae | Mus | neavei | Mus neavei | NA | NA | 528293.4 | 68.66 | 21.659 | NA | NA | NA | NA | NA | 0.250107 |
| Rodentia | Muridae | Gerbillus | pusillus | Gerbillus pusillus | 12.55 | NA | 529858.8 | 51.53 | 23.755 | NA | NA | NA | NA | NA | 0.35571 |
| Rodentia | Sciuridae | Funisciurus | leucogenys | Funisciurus leucogenys | 250 | 0.97 | 530067.1 | 156.48 | 24.864 | NA | NA | NA | NA | NA | 0.018481 |
| Rodentia | Cricetidae | Akodon | boliviensis | Akodon boliviensis | 27.5 | 4.86 | 530195.7 | 80.06 | 9.774 | 2 | 3 | 1 | 1 | 2 | 0.011694 |
| Chiroptera | Phyllostomidae | Sturnira | luisi | Sturnira luisi | NA | NA | 530510.1 | 174.37 | 21.048 | NA | 1 | 1 | 2 | 1 | 0.015391 |
| Rodentia | Sciuridae | Spermophilus | columbianus | Spermophilus columbianus | 470.94 | 3.54 | 531930.5 | 39.96 | 2.485 | 3 | 4 | 2 | 1 | 1 | 0.009747 |
| Soricomorpha | Soricidae | Myosorex | varius | Myosorex varius | 11.68 | 2.9 | 532836.8 | 49.41 | 15.753 | 2 | 1 | 2 | 1 | 3 | 0.020296 |
| Primates | Cercopithecidae | Lophocebus | aterrimus | Lophocebus aterrimus | 6510.37 | NA | 535514.8 | 157.81 | 25.079 | 3 | NA | NA | NA | NA | 0.157995 |
| Chiroptera | Vespertilionidae | Eptesicus | matroka | Eptesicus matroka | 6.57 | 1.22 | 536252 | 126.16 | 21.875 | NA | 1 | NA | NA | 3 | 0.015962 |
| Perissodactyla | Equidae | Equus | kiang | Equus kiang | 281138.6 | NA | 538675.5 | 36.44 | -5.088 | NA | NA | 1 | 1 | NA | 0.01774 |
| Soricomorpha | Soricidae | Neomys | teres | Neomys teres | NA | NA | 541334.4 | 52.66 | 6.794 | NA | NA | 3 | 1 | NA | 0.020411 |
| Rodentia | Heteromyidae | Chaetodipus | eremicus | Chaetodipus eremicus | NA | NA | 541954.6 | 26.29 | 17.44 | NA | NA | NA | NA | NA | 0.015109 |
| Rodentia | Sciuridae | Hylopetes | platyurus | Hylopetes platyurus | 56.98 | NA | 542348.2 | 225.04 | 25.221 | NA | NA | NA | NA | NA | 0.211113 |
| Primates | Cebidae | Callithrix | melanura | Callithrix melanura | NA | NA | 543620.6 | 126.64 | 24.335 | NA | NA | NA | NA | NA | 0.010941 |
| Primates | Cercopithecidae | Trachypithecus | germaini | Trachypithecus germaini | NA | NA | 544016.8 | 176.62 | 25.308 | NA | NA | NA | NA | NA | 0.784873 |
| Chiroptera | Molossidae | Tadarida | insignis | Tadarida insignis | NA | NA | 546642.6 | 122.92 | 10.16 | NA | NA | NA | NA | NA | 0.258238 |
| Peramelemorphia | Peramelidae | Echymipera | kalubu | Echymipera kalubu | 825.16 | 2.07 | 546845.2 | 243.64 | 23.728 | 1 | 3 | NA | NA | 2 | 0.019016 |
| Chiroptera | Pteropodidae | Pteropus | poliocephalus | Pteropus poliocephalus | 702.78 | 1 | 547129.9 | 70.95 | 15.814 | NA | 2 | 1 | 2 | 1 | 0.733945 |
| Chiroptera | Molossidae | Mops | leucostigma | Mops leucostigma | 26.79 | NA | 547685.2 | 125.4 | 21.911 | NA | 1 | NA | NA | 3 | 0.014867 |
| Monotremata | Ornithorhynchidae | Ornithorhynchus | anatinus | Ornithorhynchus anatinus | 1484.25 | 2 | 547828.4 | 76.73 | 15.653 | 2 | 1 | 3 | 1 | 3 | 0.03121 |
| Soricomorpha | Soricidae | Crocidura | pullata | Crocidura pullata | NA | NA | 549138.4 | 39.09 | 5.884 | NA | NA | NA | NA | NA | 0.248613 |
| Rodentia | Cricetidae | Sigmodon | fulviventer | Sigmodon fulviventer | 195.94 | 7.5 | 549411.8 | 35.24 | 14.795 | NA | NA | 1 | 1 | NA | 0.014461 |
| Chiroptera | Vespertilionidae | Nyctalus | aviator | Nyctalus aviator | NA | 1.91 | 550221.7 | 105.2 | 9.573 | NA | NA | 1 | 2 | NA | 0.158235 |
| Rodentia | Sciuridae | Spermophilus | pallidicauda | Spermophilus pallidicauda | NA | NA | 552357.3 | 19.35 | -1.649 | NA | NA | NA | NA | NA | 0.008715 |
| Rodentia | Cricetidae | Wiedomys | pyrrhorhinos | Wiedomys pyrrhorhinos | 46.7 | 3.8 | 552523.4 | 87.5 | 24.787 | NA | 2 | 2 | 2 | 2 | 0.019815 |
| Chiroptera | Molossidae | Cynomops | mexicanus | Cynomops mexicanus | NA | NA | 552617.1 | 159.18 | 22.37 | NA | NA | NA | NA | NA | 0.016031 |
| Rodentia | Cricetidae | Caryomys | eva | Caryomys eva | NA | NA | 554001.7 | 73.81 | 11.382 | NA | NA | NA | NA | NA | 0.013 |
| Rodentia | Muridae | Millardia | gleadowi | Millardia gleadowi | NA | 2.3 | 554509.5 | 30.5 | 24.455 | NA | NA | NA | NA | NA | 0.018175 |
| Chiroptera | Vespertilionidae | Cistugo | seabrae | Cistugo seabrae | NA | NA | 556498.4 | 12.74 | 16.377 | NA | NA | 1 | 2 | NA | 0.013729 |
| Rodentia | Muridae | Zelotomys | woosnami | Zelotomys woosnami | 54.1 | 4.99 | 557092.7 | 32.83 | 21.454 | NA | NA | NA | NA | NA | 0.020141 |
| Chiroptera | Phyllostomidae | Glossophaga | longirostris | Glossophaga longirostris | 13.32 | 0.98 | 557238.9 | 105.87 | 25.137 | NA | 3 | 1 | 2 | 2 | 0.254522 |
| Primates | Cebidae | Saguinus | mystax | Saguinus mystax | 555.87 | 1.93 | 557860.6 | 194.85 | 25.643 | 3 | 5 | 1 | 2 | 2 | 0.013615 |
| Chiroptera | Nycteridae | Nycteris | gambiensis | Nycteris gambiensis | 7.13 | NA | 558271.8 | 127.26 | 26.994 | NA | NA | 1 | 2 | NA | 0.017596 |
| Rodentia | Sciuridae | Lariscus | niobe | Lariscus niobe | NA | NA | 559126.8 | 221.26 | 25.082 | NA | NA | NA | NA | NA | 0.286623 |
| Chiroptera | Vespertilionidae | Myotis | annectans | Myotis annectans | 9.75 | NA | 559248.7 | 153.12 | 21.65 | NA | NA | 1 | 2 | NA | 0.014229 |
| Primates | Atelidae | Alouatta | palliata | Alouatta palliata | 6576.99 | 1.02 | 559554.9 | 185.26 | 23.738 | 3 | 2 | 1 | 2 | 1 | 0.015285 |
| Chiroptera | Vespertilionidae | Corynorhinus | mexicanus | Corynorhinus mexicanus | NA | NA | 559891.1 | 62.53 | 18.365 | NA | NA | NA | NA | NA | 0.158693 |
| Rodentia | Cricetidae | Ellobius | fuscocapillus | Ellobius fuscocapillus | 80 | 3.46 | 560273.1 | 36.01 | 11.619 | NA | 1 | NA | NA | 1 | 0.018025 |
| Chiroptera | Rhinolophidae | Rhinolophus | eloquens | Rhinolophus eloquens | 19.15 | NA | 560935.8 | 85.48 | 22.468 | NA | 1 | 1 | 2 | 3 | 0.012207 |
| Chiroptera | Molossidae | Mormopterus | loriae | Mormopterus loriae | 7 | NA | 561315.8 | 79.63 | 25.345 | NA | NA | NA | NA | NA | 0.016808 |
| Chiroptera | Vespertilionidae | Myotis | chiloensis | Myotis chiloensis | NA | 0.98 | 562838.9 | 101.3 | 5.227 | NA | 1 | 1 | 2 | 3 | 0.01728 |
| Rodentia | Muridae | Uromys | caudimaculatus | Uromys caudimaculatus | 644.42 | 1.46 | 563894.1 | 231.39 | 23.495 | 3 | 6 | 2 | 2 | 2 | 0.015497 |
| Rodentia | Muridae | Gerbillus | aquilus | Gerbillus aquilus | NA | NA | 564499.9 | 9.94 | 18.793 | NA | NA | NA | NA | NA | 0.022232 |
| Primates | Cercopithecidae | Cercocebus | chrysogaster | Cercocebus chrysogaster | NA | NA | 564761.7 | 151.44 | 24.859 | NA | NA | NA | NA | NA | 0.220424 |
| Lagomorpha | Ochotonidae | Ochotona | nubrica | Ochotona nubrica | NA | NA | 564920.8 | 71.46 | -3.936 | NA | NA | 2 | 1 | NA | 0.026567 |
| Rodentia | Heteromyidae | Chaetodipus | nelsoni | Chaetodipus nelsoni | 15.53 | 3.46 | 566702.5 | 31.52 | 17.355 | 1 | 3 | 2 | 1 | 2 | 0.016501 |
| Rodentia | Muridae | Berylmys | berdmorei | Berylmys berdmorei | NA | NA | 570245.9 | 158.53 | 24.671 | NA | NA | NA | NA | NA | 0.01922 |
| Chiroptera | Rhinolophidae | Rhinolophus | thomasi | Rhinolophus thomasi | 8.26 | NA | 571234.4 | 139.07 | 22.832 | NA | 1 | 1 | 2 | 3 | 0.012207 |
| Chiroptera | Phyllostomidae | Artibeus | aztecus | Artibeus aztecus | 20.81 | 0.99 | 572771.7 | 99.95 | 20.417 | NA | 1 | 1 | 2 | 1 | 0.015859 |
| Rodentia | Nesomyidae | Petromyscus | collinus | Petromyscus collinus | 20 | 2.46 | 573003.9 | 13.48 | 16.671 | NA | NA | NA | NA | NA | 0.020919 |
| Primates | Cercopithecidae | Nasalis | larvatus | Nasalis larvatus | 12265.65 | 1.02 | 574819.6 | 257.08 | 25.021 | 3 | 4 | 2 | 2 | 1 | 1.054688 |
[truncated: 313,610 more chars]
